# Decoding Neuromuscular Disorders Using Phenotypic Clusters Obtained From Co-Occurrence Networks

**DOI:** 10.3389/fmolb.2021.635074

**Published:** 2021-04-19

**Authors:** Elena Díaz-Santiago, M. Gonzalo Claros, Raquel Yahyaoui, Yolanda de Diego-Otero, Rocío Calvo, Janet Hoenicka, Francesc Palau, Juan A. G. Ranea, James R. Perkins

**Affiliations:** ^1^ Department of Molecular Biology and Biochemistry, Universidad de Málaga, Málaga, Spain; ^2^ CIBER de Enfermedades Raras (CIBERER), Madrid, Spain; ^3^ Institute of Biomedical Research in Malaga (IBIMA), IBIMA-RARE, Málaga, Spain; ^4^ Institute for Mediterranean and Subtropical Horticulture “La Mayora” (IHSM-UMA-CSIC), Málaga, Spain; ^5^ Laboratory of Metabolopathies and Neonatal Screening, Málaga Regional University Hospital, Málaga, Spain; ^6^ Sant Joan de Déu Hospital and Research Institute, Barcelona, Spain; ^7^ Hospital Clínic and University of Barcelona School of Medicine and Health Sciences, Barcelona, Spain

**Keywords:** neuromuscular disorders, rare disease, phenotype, network analysis, cluster, co-occurrence analysis

## Abstract

Neuromuscular disorders (NMDs) represent an important subset of rare diseases associated with elevated morbidity and mortality whose diagnosis can take years. Here we present a novel approach using systems biology to produce functionally-coherent phenotype clusters that provide insight into the cellular functions and phenotypic patterns underlying NMDs, using the Human Phenotype Ontology as a common framework. Gene and phenotype information was obtained for 424 NMDs in OMIM and 126 NMDs in Orphanet, and 335 and 216 phenotypes were identified as typical for NMDs, respectively. ‘Elevated serum creatine kinase’ was the most specific to NMDs, in agreement with the clinical test of elevated serum creatinine kinase that is conducted on NMD patients. The approach to obtain co-occurring NMD phenotypes was validated based on co-mention in PubMed abstracts. A total of 231 (OMIM) and 150 (Orphanet) clusters of highly connected co-occurrent NMD phenotypes were obtained. In parallel, a tripartite network based on phenotypes, diseases and genes was used to associate NMD phenotypes with functions, an approach also validated by literature co-mention, with KEGG pathways showing proportionally higher overlap than Gene Ontology and Reactome. Phenotype-function pairs were crossed with the co-occurrent NMD phenotype clusters to obtain 40 (OMIM) and 72 (Orphanet) functionally coherent phenotype clusters. As expected, many of these overlapped with known diseases and confirmed existing knowledge. Other clusters revealed interesting new findings, indicating informative phenotypes for differential diagnosis, providing deeper knowledge of NMDs, and pointing towards specific cell dysfunction caused by pleiotropic genes. This work is an example of reproducible research that i) can help better understand NMDs and support their diagnosis by providing a new tool that exploits existing information to obtain novel clusters of functionally-related phenotypes, and ii) takes us another step towards personalised medicine for NMDs.

## 1 Introduction

Neuromuscular disorders (NMDs) encompass a range of pathologies affecting muscle function (Roy et al., 2015) that can be caused by problems in spinal motor neurones, peripheral nerves, muscles, and neuromuscular junctions. They affect 6–8 million people worldwide (Scotton et al., 2014) and lead to elevated morbidity and mortality (Mccormack et al., 2013). Many result from genomic mutations (Laing, 2012), although they can also be caused by autoimmune disorders and infections (Kraker and Zivković, 2011). Around half manifest during childhood and classification is often based on the affected area (Turakhia et al., 2013). Updated details of known mutations associated with NMDs are published yearly (Benarroch et al., 2019) (http://www.musclegenetable.fr/index.html). Table 1 illustrates the main types of NMDs and shows their high heterogeneity in terms of clinical manifestation.

**TABLE 1:** Classification of NMDs including some examples.

Type	Description	Examples
Muscular dystrophies (MDs)	Diseases causing weakness and degeneration of the skeletal muscles	Myotonic dystrophy;facioscapulohumeral MD; EDMD; Duchenne MD; Becker MD; LGMDs; congenital MDs
Myopathies	Muscle diseases in which the muscle fibres do not function properly, resulting in muscle hypotonia and weakness	Congenital myopathies; distal myopathies; endocrine myopathies; mitochondrial myopathies; metabolic myopathies
Peripheral nerve diseases	Diseases where motor and sensory nerves that connect the brain and spinal cord to the rest of the body are affected, causing impaired sensations, movements and muscular weakness	Charcot-Marie-Tooth disease; Giant axonal neuropathy
Motor neurone diseases	Diseases where motor neurones progressively lose function, causing the muscles they control to become weak and eventually non-functional	Hereditary spastic paraplegias; spinal muscular atrophy; spinal-bulbar muscular atrophy
Ion channel diseases	Diseases associated with defects in ion channels, typically marked by muscular weakness, absent muscle tone, or episodic muscle paralysis	Andersen-Tawil syndrome; hyperkalemic periodic paralysis; hypokalemic periodic paralysis; myotonia congenita; paramyotonia congenita; potassium-aggravated myotonia
Neuromuscular junction diseases	Neuromuscular junction disorders that result from the destruction, malfunction or absence of one or more key proteins involved in the transmission of signals between muscles and nerves	Congenital myasthenic syndromes; Lambert-Eaton myasthenic syndrome; myasthenia gravis

EDMD: Emery-Dreifuss muscular dystrophy; LGMDs: limb girdle muscular dystrophies.

Although diagnosis of NMDs has been aided in recent years by advances in whole-exome/genome sequencing (Brown and Meloche, 2016), it still requires a high level of medical specialisation, due to the high phenotypic and pathophysiology diversity, and large number of causal genes (McDonald, 2012; Roy et al., 2015). Moreover, given that individual NMDs tend to be rare, it can be hard to find sufficient patients to conduct well-powered studies. As such, a range of diagnostic tests (including electrophysiology, tissue biopsies, and measuring levels of certain enzymes such as elevated serum creatinine kinase), and molecular imaging (Thavorntanaburt et al., 2018) must be used, and diagnosis can be slow (Spuler et al., 2011). There is no cure for most NMDs but rather symptomatic treatments to delay progression. Current research is focused on gene therapies and investigating new medications (Scoto et al., 2018). However, efforts are hampered by their etiological heterogeneity and phenotype diversity.

Further work is needed to better understand how NMDs are related in terms of phenotypic overlap and underlying genes and mechanisms, in order to facilitate diagnosis and improve treatment. Although not specific to NMDs, previous studies have compared phenotypic profiles between different diseases to build clusters of related phenotypes (Sirota et al., 2009; Bagley et al., 2016); others have focused on phenotype-similarity based on co-morbidity across multiple diseases (Rzhetsky et al., 2007; Hidalgo et al., 2009). Such studies enable us to identify patterns between groups of diseases and phenotypes by showing how they tend to co-occur. This has multiple potential uses for disease classification and diagnosis. Further studies have investigated the connection between clinical manifestations in disease by integrating gene-disease and protein-protein interaction data (Yang et al., 2011; Hwang et al., 2012; Zhou et al., 2014), as well as connecting proteins with phenotypes through the use of phenotypic clusters based on similarity and by predicting proteins associated with the phenotypes through machine learning (Ren et al., 2020). These studies lead the way towards explaining the co-occurrence of phenotypic patterns across diseases through common underlying mechanisms.

There are several resources that map known diseases to their pathological phenotypes and associated genes. For example, MENDELIAN (https://www.mendelian.co/es/) allows the symptom-guided search of rare diseases. OMIM (Online Mendelian Inheritance in Man) gathers data obtained *via* curation of the biomedical literature (Hamosh, 2004; Amberger et al., 2019) to provide a clinical synopsis of all known Mendelian traits and disorders, describing genes, allelic variants and pathological phenotypes. Orphanet (http://www.orphadata.org and https://www.orpha.net/) is based on expert knowledge, gathering information about rare diseases specifically, with the aim of collecting and unifying the scarce knowledge available for such disorders, including NMDs of genetic origin. There are several tools that exploit these databases to associate disease-related phenotypes with genes, including *Phen2Gene* (Zhao et al., 2020), *AMELIE* (Birgmeier et al., 2020), *Phevor* (Singleton et al., 2014), *Phenolizer* (Yang et al., 2015) and *Phenomizer* (Köhler et al., 2009). Such studies require the use of common phenotype terminology in such a way that makes the information amenable to computational analysis (Hoehndorf et al., 2015). The Human Phenotype Ontology (HPO, https://hpo.jax.org/), provides such a standardised vocabulary to describe phenotypic abnormalities associated with more than 7 800 diseases (Köhler et al., 2021).

However, few studies have combined phenotype and gene information for groups of heterogeneous diseases to look for related phenotypes shared across multiple disorders with common underlying mechanisms. Such an approach was recently conducted by our group using patients with largely undiagnosed rare disorders (Díaz-Santiago et al., 2020), taking advantage of the rare-disease database DECIPHER (Firth et al., 2009). This work showed that by using biomedical networks and systems medicine approaches our understanding of rare diseases can be improved based on phenotype co-occurrence patterns. It also showed the power of the re-analysis of existing data from public databases to obtain new knowledge, something that is recommended in the research community whenever possible (Kovalevskaya et al., 2016; Tan et al., 2020). In this work, the automated workflow *PhenoClusters* is used to investigate phenotype co-occurrence across NMDs and produce functionally coherent clusters of phenotypes with similar underlying biological functions. This can help differentiate diagnosis (‘elevated serum creatine kinase’ is the most significant NMD phenotype) and provide a better understanding of NMDs (many clusters gather typical phenotypes and functions of NMDs) based on the specific cell functions (unanticipated phenotypes such as ‘macroglossia’ and ‘arthogryposis’ point to cell dysfunctions involved in an NMD), including those affected by pleiotropic/multi-functional genes.

## 2 Materials and Methods

### 2.1 *PhenoClusters* Workflow

**FIGURE 1:**
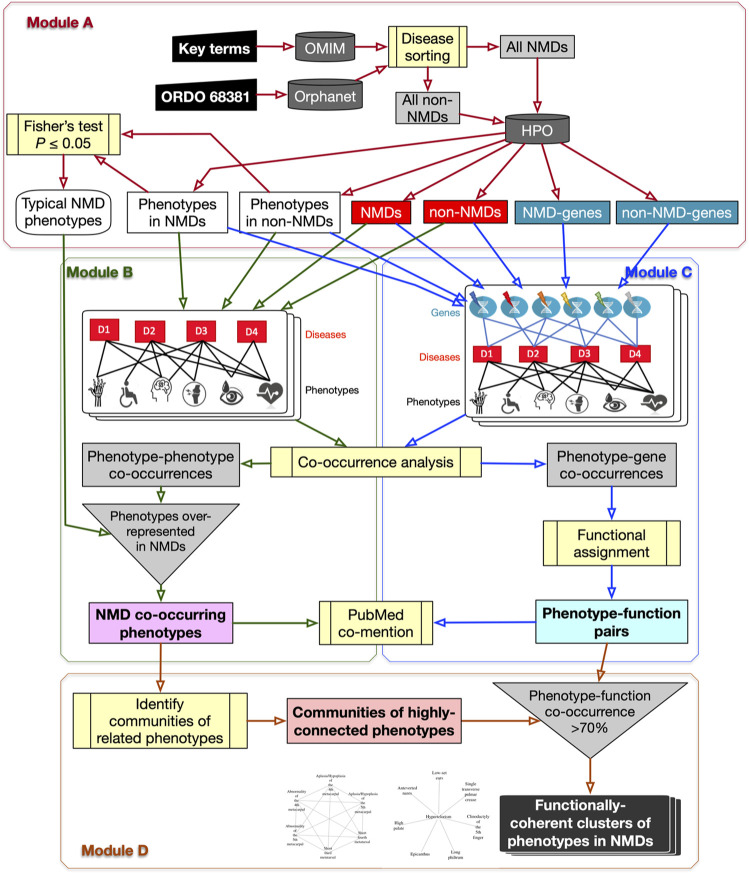
Flowchart of the *PhenoClusters* workflow. Module **(A)**: all diseases in OMIM and Orphanet were classified as “NMDs” or “non-NMDs”. HPO is then queried with those disease, retaining only those diseases (red boxes) for which both a gene (teal boxes) and a phenotype (white boxes) are known. Module **(B)**: a bipartite network was constructed and analysed to obtain the typical “NMD co-occurring phenotypes” that tended to occur in NMDs. Module **(C)**: a tripartite network enabled the obtention of new, significant phenotype-gene pairs that were translated to “phenotype-function pairs”. Module **(D)**: communities of highly related phenotypes were filtered based on shared function to obtain the final set of “functionally coherent clusters of phenotypes in NMDs”. PubMed co-mention was used to assess the reliability of the co-occurrence approach. Dark grey cylinders correspond to databases; yellow boxes are relevant analyses; inverted triangles indicate process merging; light grey rectangles are intermediate sets of results; coloured rectangles with text in bold mark relevant results.

The *PhenoClusters* workflow described in this study is based on OMIM, Orphanet and HPO data and is outlined in Figure 1. The following sections describe its modules in more detail.

#### 2.1.1 Module A: NMD Datasets With Genes, Phenotypes and Typical NMD Phenotypes

The OMIM and Orphanet databases (as of 15th-Nov-2019) were used in parallel since they gather information from different origins and with different goals. OMIM was searched with NMD related keywords (*muscular dystrophy, myopathy, myasthenic, myasthenia, neuropathy, amyotrophic lateral sclerosis, spinal muscular atrophy, spinal and bulbar muscular atrophy, myotonia, periodic paralysis, myotonic dystrophy, mitochondrial cytopathy, necrotizing encephalomyelopathy, mitochondrial DNA depletion*) to obtain a list of NMDs as complete as possible. Since Orphanet organises diseases based on ORDO (Orphanet Rare Disease Ontology) (Vasant et al., 2014), all diseases descendant of the category neuromuscular diseases (Orphanet:68381) were used. Hence, diseases in OMIM and Orphanet were assigned to “All NMDs” or “All non-NMDs” (grey rectangles in Module A of Figure 1 and Table 2). These were used to query HPO (v1.2; 15th-Nov-2019) to retain only those diseases (“Non-NMDs” and “NMDs” in Table 2, corresponding to red rectangles in Module A of Figure 1) for which both phenotype (white rectangles in Module A of Figure 1) and gene (teal rectangles in Module A of Figure 1) information was available.

**TABLE 2:** Summary of diseases found in OMIM and Orphanet databases and the retained diseases, phenotypes and genes after the HPO query.

	OMIM			Orphanet		
		HPO			HPO	
	Diseases	Phenotypes	Genes	Diseases	Phenotypes	Genes
Total	26 943	-	-	3 431	-	-
All non-NMDs	26 387	-	-	3 204	-	-
All NMDs	556	-	-	227	-	-
Complete information in HPO	5189	6385	4015	2020	5430	2730
Non-NMDs	4765	6183	4015	1894	5312	2730
NMDs	424	1314	345	126	1007	222
Common	0	1112	345	0	889	222
Typical in NMDs	-	335	0	-	216	0

“-”: not considered or not relevant for the analysis.

Finally, phenotype frequencies between “NMDs” and “non-NMDs” in Table 2 and Figure 1 were compared using Fisher’s exact test (one-tail) with a threshold of P≤0.05. A list of “Typical NMD phenotypes” (Table 2 and Figure 1) was thus obtained.

#### 2.1.2 Module B: Bipartite Network to Obtain Phenotype Co-Occurrences

All diseases (“NMDs” and “non-NMDs”) as well as their corresponding phenotypes were used to construct a standard bipartite network (Pavlopoulos et al., 2018) of disease-phenotype pairs to find common (co-occurring) phenotypes across diseases. Phenotype co-occurrences (grey rectangle in Module B of Figure 1) were extracted using *NetAnalyzer* (Rojano et al., 2017) (see below). To recover only significant associations for NMDs (pale magenta rectangle of “NMD co-occurring phenotypes” in Module B of Figure 1), all phenotype-phenotype co-occurrences were filtered using the “Typical NMD phenotypes” obtained from Module A.

#### 2.1.3 Module C: Tripartite Network to Obtain Phenotype-Function Pairs

A tripartite network of all diseases (NMDs and non-NMDs), their phenotypes and the affected genes was constructed to link, in first instance, phenotypes with genes, based on their co-occurrence across diseases. The network was constructed as described in (Rojano et al., 2017) and then investigated using *NetAnalyzer* to find significant phenotype-gene co-occurrences (grey rectangle in Module C of Figure 1). Phenotype-gene pairs were converted into phenotype-function pairs based on the biological process sub-ontology from Gene Ontology (GO), KEGG (Kyoto Encyclopaedia of Genes and Genomes) pathways and Reactome pathways. To reveal those enriched functions significantly associated with a given phenotype, all genes associated with that phenotype were obtained and this gene list was used for functional enrichment, based on over-representation analysis (Yu et al., 2012). Association between phenotypes and genes/functions was performed separately for OMIM and Orphanet. This resulted in significant “phenotype-function pairs” (pale cyan rectangle in bold in Module C of Figure 1).

#### 2.1.4 Module D: Functionally-Coherent Clusters of Phenotypes in NMDs

The typical NMD phenotypes obtained in Module B were used to detect communities of related, highly interconnected phenotypes (grey rectangle at bottom centre of Figure 1) using the R package *linkcomm*. Following the rationale proposed in (Díaz-Santiago et al., 2020), phenotype communities for which at least 70% of the constituent phenotypes shared the same functional annotations obtained from the phenotype-function pairs of Module C were retained. These were considered “functionally coherent clusters of phenotypes in NMDs” (final dark rectangle in Module D of Figure 1) and constitute the major result of the workflow. OMIM clusters were tagged with “*”, while Orphanet clusters were tagged with “#”.

### 2.2 PubMed Co-Mention to Validate the Co-Occurrence Approach

Phenotypes co-occurring in NMDs from Module B and phenotype-function pairs from Module C were investigated in terms of co-mention in the scientific literature, by comparing how many PubMed abstracts mention both terms in a given pair, to how many abstracts mention only one of them. To do so, the NCBI Entrez Programming Utilities API was used as previously described (Díaz-Santiago et al., 2020). In brief, the different textual descriptions for a given i) phenotype as described in HPO, ii) GO term from the biological process vocabulary, iii) KEGG pathways, and iv) Reactome pathways, were retrieved. All these terms were queried separately in PubMed (the complete database) to obtain the lists of PMIDs (PubMed identifiers) of abstracts mentioning each given term. PMID lists were then compared for each pair of terms (phenotype-phenotype, phenotype-GO, phenotype-KEGG or phenotype-Reactome) using Fisher’s exact test to detect the significantly (P≤0.05) co-mentioned pairs. The numbers of significant pairs obtained for each set was then compared to the number of significant pairs obtained from random models, by calculating whether the probability of finding as many significant pairs in the random dataset was at least as high as that detected using real data.

### 2.3 Tissue Expression Testing

Genes in phenotype-gene associations were further analysed to see if they were more likely to be expressed in neuronal or muscular tissues, both highly relevant for NMDs. To do so, expression location of genes paired with NMD phenotypes was compared to that of genes paired with non-NMD phenotypes. Expression locations were obtained from the normal tissue expression dataset (*normal_tissue.tsv.zip* file) at The Human Protein Atlas version 20.0 (Uhlén et al., 2015) (http://www.proteinatlas.org). A gene was considered as expressed in neuronal or muscular tissue if it showed medium or high expression in one of the following tissue types: cerebellum, cerebral cortex, hippocampus, caudate neuronal cells, skeletal muscle or heart muscle. The relative proportions of genes from each set that showed expression in neuronal or muscular tissue were compared using Fisher’s exact test.

### 2.4 *PhenoClusters* Architecture and Execution Details

All the above calculations have been implemented as an automated workflow named *PhenoClusters* that is based on *PhenCo* (Díaz-Santiago et al., 2020) and uses additional scripts from (Jabato et al., 2020). The Picasso supercomputer of University of Malaga was used for code implementation and testing. It consists of an OpenSUSE LEAP 12.3 with Slurm queue system and Infiniband network (54/40 Gbps) containing 216 nodes with Intel E5-2670 2.6 GHz cores for a total of 3 456 cores and 22 TB of RAM. The code is available from GitHub at https://github.com/Elenadisa/PhenoClusters. The main requirements are Python 3, Ruby 2.4.1, R 4.0.0 or higher, Bioconductor 3.4 (Huber et al., 2015) or higher, scripts from *sys_bio_lab* (Jabato et al., 2020), and *Anaconda Individual edition* (https://docs.anaconda.com/anaconda/install/) as package and environment manager. The workflow was managed using *AutoFlow* (Seoane et al., 2016).

For co-occurrence analysis, *PhenoClusters* requires *NetAnalyzer* (Rojano et al., 2017). Although many co-occurrence methods are available, previous work suggests that the hypergeometric index (*HyI*) (Fuxman Bass et al., 2013), which can be considered analogous to a contingency table based approach, is the most suitable for co-occurrence studies (Rojano et al., 2017; Bueno et al., 2018). Hence, HyI≥2 was set to calculate associations within the layers of a network and obtain phenotype-phenotype or phenotype-gene associations that were considered significant co-occurrences (Díaz-Santiago et al., 2020).

Functional enrichment was performed using the genes associated with each phenotype. The Bioconductor package *clusterProfiler* 3.18.0 (Yu et al., 2012) was used to extract the biological process subontology from GO and the KEGG pathways and conduct over-representation analysis for these resources. The Bioconductor package *ReactomePA* 1.34.0 (Yu and He, 2016) was used to extract Reactome pathway data and perform over-representation analysis for this resource. Both packages were executed with default parameters (*pvalueCutoff* = *0.05, pAdjustMethod* = “*BH*”*, universe, qvalueCutoff* = *0.2, minGSSize* = *10, maxGSSize* = *500*) and only the assignments with P≤0.05 after Benjamini-Hochberg multiple testing adjustment (Benjamini and Hochberg, 1995) were considered significant.

Highly interconnected phenotype communities were obtained using the R-CRAN package *linkcomm* 1.0.13 (Kalinka and Tomancak, 2011) with default parameters for undirected networks. R-CRAN packages required for graphical representations and data management are *ggplot2* 3.3.2, *RColorBrewer* 1.1.2, *igraph* 1.2.6, *dplyr* 1.0.2 and *VennDiagram* 1.6.20. The final, user-friendly HTML reports were produced using R markdown packages *rmarkdown* 2.6, *knitr* 1.30 and *kableExtra* 1.3.1.

Details about *PhenoClusters* execution can be found at https://github.com/Elenadisa/PhenoClusters. As can be seen, diseases from OMIM and Orphanet were considered separately in different scripts. One run usually takes at least 6 h 45 min without the co-mention verification, since literature analysis takes a very long, heterogeneous amount of time depending on the PubMed server overload. The resulting reports are named *omim_report.html* (Supplementary Material S1) and *orphanet_report.html* (Supplementary Material S2). To simplify cluster inspection, they are also saved as independent files in *omim_clusters_report.html* and *orphanet_clusters_report.html* including the cluster ID, the phenotype graph together with HPO IDs and descriptions, as well as the associated functions and the genes identified within them. Clusters showing functional coherence are given at the top of the file. For the sake of simplicity, “*” is used to tag OMIM clusters, while Orphanet clusters are tagged by “#”, as indicated in Module D.

## 3 Results

### 3.1 Typical NMD Phenotypes Reflect Neuromotor Impairments

**FIGURE 2:**
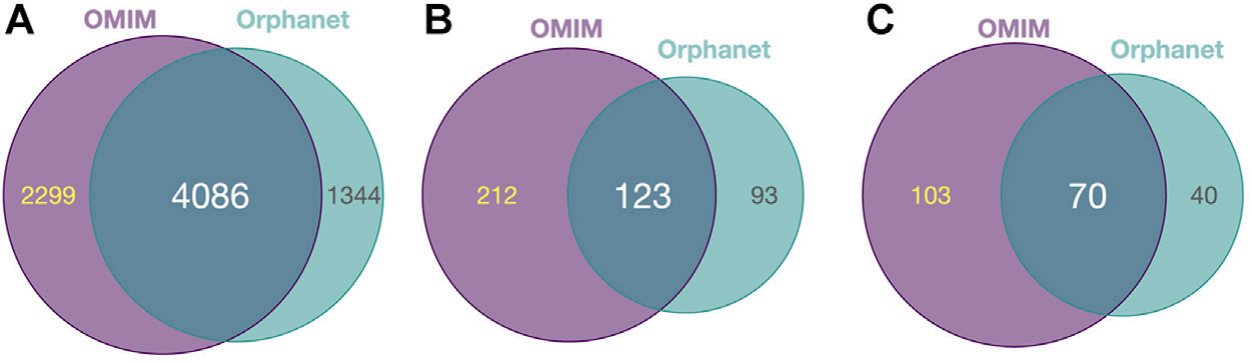
Common and distinct disease-associated HPO-phenotypes in OMIM and Orhpanet described in Table 2. **(A)**: Distribution of the 6 385 HPO phenotypes for OMIM and the 5 430 phenotypes for Orphanet for all NMDs and non-NMDs. **(B)**: Distribution of the typical 335 OMIM and 216 Orphanet phenotypes significantly (P<0.05) associated with NMDs. **(C)**: Same as B, but using the increased significance (P<0.001) NMD-associated phenotypes (172 for OMIM and 110 for Orphanet).

Diseases in OMIM and Orphanet were classified as NMD or non-NMD (Table 2). All downstream analysis treated the resources separately (Supplementary Material S1, S2). The total numbers of diseases in each resource for which genes and pathological phenotype information was available are shown in Table 2; full details can be found in Supplementary Material S3, S4. It can be seen that relatively few diseases were considered NMDs with known genes and phenotypes in HPO (424 and 125 in OMIM and Orphanet, respectively). Many phenotypes were shared by both NMDs and non-NMDs (row “Common” in Table 2). Fisher’s exact test was then performed to compare the relative occurrence of each phenotype between disease groups to find those relatively more common in NMDs. This resulted in 335 and 216 typical NMD phenotypes for OMIM and Orphanet. Overlap between the two is shown in Figure 2, where Panels B and C show that the proportion of common phenotypes in both sets increases with decreasing cutoff *p*-values. This reinforces the suitability of the co-occurrence approach to extract typical NMD phenotypes.

The top over-represented phenotypes are shown in Supplementary Material S5. The most significant in both resources is ‘elevated serum creatine kinase’, which reflects the important role of muscle decay in NMDs, given that the normal location of this enzyme is the cytoplasm and mitochondria, not serum (Moghadam-Kia et al., 2016). This result is reassuring with respect to the approach of *PhenoClusters*, as elevated serum creatine kinase is routinely tested when diagnosing NMDs as a consequence of muscle injury (Thavorntanaburt et al., 2018).

The top phenotypes (Supplementary Material S5) seem to be more indicative of muscular impairment than neuronal dysfunction, pointing to proximal or distal muscular weakness, both very typical in the clinical history of NMD patients (McDonald, 2012; Mukherjee et al., 2019). Other important phenotypes are related to specific diseases, such as Duchenne/Becker muscular dystrophy (‘calf muscle hypertrophy’, ‘Gowers sign’), where muscle hypertrophy can exaggerate postural instability and joint contracture (Kornegay et al., 2012). In contrast, the absence of ‘hypotonia’-related phenotypes in this top ten list is notable. However, it should be made clear that such phenotypes were also significantly over-represented among NMDs, just not included in the top ten (Supplementary Material S3, S4). To conclude, the *PhenoClusters* approach to obtain statistically over-represented phenotypes for NMDs extracts phenotypes characteristic of these disorders.

### 3.2 Typical NMD Phenotypes Tend to Co-Occur Across Diseases

Bipartite networks were created (one for OMIM diseases and another for Orphanet), with nodes representing diseases and phenotypes, and edges representing known relations in OMIM or Orphanet respectively. The network was analysed to identify pairs of typical NMD phenotypes that tend co-occur across diseases (Module B in Figure 1). Around a quarter of all phenotype pairs co-occurring in at least one disease showed significant co-occurrence (27.61% in OMIM and 21.20% in Orphanet). Only a small subset (≤1 %) of all phenotype pairs represent significant co-occurrence between NMD phenotypes. However, this value was far lower for the random sets of non-NMD phenotypes, being almost negligible (Table 3), showing that pairs of NMD phenotypes are far more likely to significantly co-occur across multiple diseases than pairs of randomly selected phenotypes. These results point to the presence of comorbidity between NMD phenotypes.

**TABLE 3:** Numbers of pairs of co-occurring phenotypes for the OMIM and Orphanet databases. Significantly co-occurring pairs defined as having a hypergeometric index value ≥2.

Category	OMIM	Orphanet
Total co-occurring phenotype pairs	455 379	467 899
Significant co-occurring pairs (HyI≥2)	125 720	99 193
Significant co-occurring pairs, NMD phenotypes	4 579	2 314
Significant co-occurring pairs, non-NMD phenotypes*^a^*	655±46.4	173±16.43

^a^ This result is expressed as the average ± SD for 100 sets of 335 randomly chosen OMIM non-NMD phenotypes and 216 random Orphanet non-NMD phenotypes; the non-NMD phenotypes were selected to have a similar prevalence to the NMD phenotypes.

### 3.3 Co-Occurring Phenotypes Tend to be Co-Mentioned in Research Papers

As well as showing that the typical NMD phenotypes tended to co-occur across diseases much more frequently than non-NMD phenotypes, further validation was performed based on co-mention analysis for each phenotype pair within PubMed abstracts. The NMD phenotype pairs were shown to be co-mentioned in the literature much more frequently than equal numbers of pairs of randomly chosen non-NMD phenotypes (Table 3). Fisher’s exact test analysis revealed that 1 173 OMIM pairs and 689 Orphanet pairs (Supplementary Tables S1, S2) were significantly co-mentioned. Conversely, for non-NMDs, the numbers of significant pairs were much lower (333.08±34.63 in OMIM and 110.0±13.47 in Orphanet, Supplementary Tables S1, S2, respectively). These differences were also supported by the distribution of their *p*-values (Supplementary Figures S1, S2) that were clearly more dense at P≤0.05 for NMDs than in non-NMDs. These results demonstrate the validity of the *PhenoClusters* approach to find pairs of NMD-related phenotypes that tend to co-occur together.

### 3.4 Phenotype-Function Associations are Robust

Associations between NMD phenotypes and genes and functions were calculated in order to uncover potential underlying mechanisms leading to the expression of a pathological phenotype. This was done by initially connecting phenotypes with genes using a tripartite network based approach (Module C in Figure 1) by combining all disease-phenotype pairs and all disease-gene pairs in OMIM and Orphanet separately. The networks were then analysed in order to connect genes with phenotypes based on co-occurrence across multiple diseases.

Using a threshold of HyI≥2, 34 139 and 36 218 phenotype-gene associations were obtained for OMIM and Orphanet, respectively; from these, a total of 1 078 and 611 distinct genes were associated with NMD phenotypes, respectively. There was significant overlap between OMIM and Orphanet in terms of genes associated with NMD phenotypes ($P<2.2\times 10^{-16}$, Fisher’s exact test using all phenotype-associated genes as background). Moreover, a significantly greater proportion of the NMD associated genes (84.41% in OMIM, 86.73% in Orphanet) were shown to be expressed in neuronal or muscle tissue than the non-NMD associated genes (70.32% in OMIM. 72.88% in Orphanet), which is a statistically significant difference ($P=2.2\times 10^{-16}$ and $P=6.3\times 10^{-11}$, for OMIM and Orphanet, respectively). The same kind of test was performed for the remaining non-relevant tissues, revealing in this case that there was no significant difference between NMD and non-NMD genes after a Fisher’s test (P>0.9 for both databases).

The genes associated with each phenotype were then used for functional enrichment analysis using *clusterprofiler* and *ReactomePA* to find over-represented (P≤0.05) GO terms (biological process ontology), and KEGG and Reactome pathways (Table 4). As with gene-based analysis, there was significant overlap between OMIM and Orphanet ($P<2.2\times 10^{-16},6.842\times 10^{-8}$, and $2.381\times 10^{-14}$, respectively, using Fisher’s exact test with all phenotype-associated functions used as background). Supplementary Material S6 shows the overlap between the different resources using Venn diagrams. Therefore, genes associated with NMD phenotypes are significantly expressed in neuronal and muscle tissue, the most relevant for NMDs, supporting the robustness of the phenotype-function pairs obtained by *PhenoClusters*.

**TABLE 4:** Numbers of genes and functions associated with phenotypes. Total: all genes in each dataset. “Any”: genes/functions associated with any phenotype. “Only NMD”: genes/functions associated with a typical NMD phenotype.

Feature	OMIM-based				Orphanet-based				OMIM-Orphanet overlap			
	Genes	GOs	KEGG	Reac	Genes	GOs	KEGG	Reac	Genes	GOs	KEGG	Reac
Total	4015	-	-	-	2730	-	-	-	2452	-	-	-
Any	3870	6398	214	1397	2700	6281	209	1356	2374	6260	208	1348
Only NMD	1078	3747	142	821	611	3618	123	631	360	2666	102	451

“-”: not considered or relevant in the analysis.

In spite of the robustness of these novel associations between phenotypes and functions, additional validation was performed based on co-mention analysis using PubMed abstracts as a sign of relevance. Term co-occurrence of a given phenotype alongside its corresponding function was looked for within the same abstract (Supplementary Figures S1, S2). Only a subset of the phenotype-function pairs obtained from the network were significantly co-mentioned (“Confirmed” columns in Table 5). However, this subset was several times larger than the number of co-mentioned pairs within the “random” pairs, and this difference was significant for all comparisons ($P<2.2\times 10^{-16}$) using Fisher’s exact test. Consistent results were found for both OMIM and Orphanet: respectively i) 5% and 4% of phenotype-GO pairs, ii) 10.5% and 9% of KEGG pairs, and iii) 4% and 3% of Reactome pairs were significantly co-mentioned. As such, *PhenoClusters* (Figure 1) appears to produce robust and reliable associations between NMD phenotypes and functions based on phenotype and gene co-occurrence across diseases. Even though only a small fraction could be confirmed in PubMed abstracts, this was several times more than would be expected by chance. The analysis showed much higher co-mention for the KEGG associations as a proportion of the total pairs. This supports our previous work suggesting that information in KEGG is more reliable for functional studies in certain situations (Luque-Baena et al., 2014).

**TABLE 5:** Co-mention validation of phenotype-function pairs. “All” corresponds to all pairs including a function from Table 4. “Confirmed” refers to the number of these pairs that were significantly co-mentioned in PubMed. “Random” refers to the number of pairs in a randomised list based on “All” pairs that were significantly co-mentioned in PubMed; average of 100 random datasets ± SD is shown in this case.

Paired	Phenotypes					
function	OMIM			Orphanet		
	All	Confirmed	Random^a^	All	Confirmed	Random^a^
GO	567 721	26 841	10 814±80.5	535 389	21 863	9 613±93.4
KEGG	17 679	1 858	627±22.1	17 104	1 556	628±21.2
Reactome	82 826	3 278	1001±166.3	78 409	2 402	816±28.7

^a^ The randomised phenotype-function pair set was formed by shuffling the links between the pairs in each list, keeping the total number of links per phenotype/function unchanged. This sampling procedure was repeated to produce 100 different replicas of randomised phenotype-function pairs. These sets were used in the corresponding Fisher’s exact tests.

### 3.5 Clusters Formed Between NMD Phenotypes are Functionally Coherent

The pairs of significantly co-occurring NMD phenotypes (pale magenta rectangle of Module B in Figure 1) were processed to extract communities of highly interconnected phenotypes (Module D in Figure 1). As summarised in Table 6, the number of communities and average number of phenotypes per community is higher using the modules based on NMD phenotypes compared to non-NMDs. This indicates that the co-occurring NMD phenotypes tend to form groups of related phenotypes.

**TABLE 6:** Overview of communities (numbers and average sizes) generated with the co-occurrent phenotype pairs for OMIM and Orphanet. Values shown for communities obtained using pairs of NMD phenotypes and equal numbers of randomly generated pairs of non-NMD phenotypes (average ± SD).

Community	OMIM		Orphanet	
	NMD	non-NMD^a^	NMD	non-NMD^a^
Total number	231	94.98±9.6	150	23.85±4.293
Phenotypes per community	16.75	5.55±0.6	13.63	4.15±0.278
Functionally coherent clusters	40	41.21±7.72	72	14.51±3.189

^a^ ‘non-NMD’ results correspond to the grouping obtained using the 100 random sets described in Table 3.

The phenotype communities were combined with the phenotype-function associations obtained previously (light cyan rectangle of Module C in Figure 1) to evaluate their functional coherence. In total, 40 OMIM communities and 72 Orphanet communities (Table 6) showed coherent function, defined as having shared functional enrichment for at least 70% of their constituent phenotypes; they constituted the “functionally coherent clusters”. The complete list of functionally coherent clusters is provided at the end of the reports given in Supplementary Material S1, S2. In terms of cluster properties, some have high interconnection (*193, *138, *219, #115, #150), others show spoke-hub structure, with a central node connected to a number of other nodes which do not then connect to each other (clusters *58, *1, *3, *4, *91, *26, #144, #2, #3, #21, #60, #22), and others have near linear topology (*146, *85, #97). Compared to their respective random non-NMD models Table 6, OMIM presents a similar number of clusters, in stark contrast with Orphanet, for which 5 times more clusters were found. The coherence threshold must be decreased to 50% to find a substantially different number of coherent clusters in OMIM compared to the random datasets (Supplementary Figure S1), This difference may be due to the OMIM clusters being larger than for Orphanet (16.75 vs. 13.63 phenotypes, respectively) or related to the nature of the diseases considered NMDs in each dataset. These results support the approach presented here in which the information from the different databases is considered separately, with Orphanet arguably providing more confident results with respect to how well the typical NMD phenotypes in these clusters represent realistic and useful groupings of co-occurrent phenotypes that reflect co-morbidity with shared underlying cellular functions.

### 3.6 Clinical Application I: Functionally Coherent Clusters Help Direct Diagnosis

The functionally coherent clusters found by our approach (Supplementary Material S1, S2) were inspected by clinicians with expertise in paediatric neuromuscular diseases, finding clusters with sets of expected phenotypes for NMDs, and others with unexpected phenotypes. Clusters are grouped in Table 7 based on the area in which the constituent phenotypes manifest, followed by further subdivision by group of NMD. Since they represent direct links between NMDs, their symptoms and their underlying mechanisms, they could be used for clinical diagnosis, by suggesting novel phenotypes to test for, should a patient present other phenotypes within a cluster. They also suggest potentially affected genes, useful for directing genetic analysis, and potentially affected functions. Although this analysis necessarily has a subjective aspect, given that clinicians differ in training and experience, it is remarkable that *PhenoClusters* produces functionally coherent clusters easily recognised by clinicians. In summary, there are multiple clusters that fit in with our current knowledge about NMDs as shown in Table 7, which reinforces once again the coherence of the results generated by the presented approach.

**TABLE 7:** Known associations between NMDs, clusters and underlying cell function, sorted by topological and pathophysiological levels.

Topological or pathophysiological level	Group of NMD	Cluster ID: main cell function involved^a^	References
*Peripheral nervous system*			
2^nd^ motor neuron in the anterior horn of the spinal cord	Spinal muscular atrophy	*82: R-HSA-191859 snRNP Assembly	Bäumer et al. (2009), Rossoll and Bassell (2009)
		*82: R-HSA-194441 Metabolism of non-coding RNA	
Peripheral nerve	Peripheral neuropathies	*3: GO:0042552 – myelination	Kamil et al. (2019), Zhou and Notterpek (2016)
		*23: GO:0008366 – axon ensheathment	
		*67: GO:0042552 – myelination	
		*200: GO:0008366 – axon ensheathment	
		*47: GO:0008366 – axon ensheathment	
Neuromuscular junction	Myasthenic syndromes	*97: GO:0007274 – neuromuscular synaptic transmission	Rodríguez Cruz et al. (2018), Souza et al. (2016)
		*166: GO:0007271 – synaptic transmission, cholinergic	
		*166: GO:0007528 – neuromuscular junction development	
Muscle	Congenital or developmental myopathies	*15: GO:0048747 – muscle fiber development	Sarnat (1994), Cassandrini et al. (2017)
		*6: GO:0051146 – striated muscle cell differentiation	
		*6: GO:0048747 – muscle fiber development	
	Myotonic syndromes/dystrophies	*193: GO:0003012 – muscle system process	Meola and Cardani (2015)
	Muscular dystrophies (non-congenital)	#13: GO:0048747 – muscle fiber development	Lovering et al. (2005)
		#13: GO:0007517 – muscle organ development	
		#112: GO:0007517 – muscle organ development	
		#112: GO:0048747 – muscle fiber development	
		#60: hsa05414 – dilated cardiomyopathy	
		#60: hsa05410 – hypertrophic cardiomyopathy	
		#60: R-HSA-390522 – Striated Muscle Contraction	
*Mitochondrial target*			
Mitochondrial genome maintenance	Mitochondrial myopathy or disease	*205: GO:0000002 – mitochondrial genome maintenance	Pfeffer and Chinnery (2013)
Mitochondrial inheritance	Mitochondrial myopathy or disease	*48: GO:0006119 – oxidative phosphorylation	
		*48: GO:0022900 – electron transport chain	
		*48: GO:0045333 – cellular respiration	

^a^ “*” tag for OMIM clusters. “#” tag for Orphanet clusters.

### 3.7 Clinical Application II: Unexpected Phenotypic Relationships in NMDs

**FIGURE 3:**
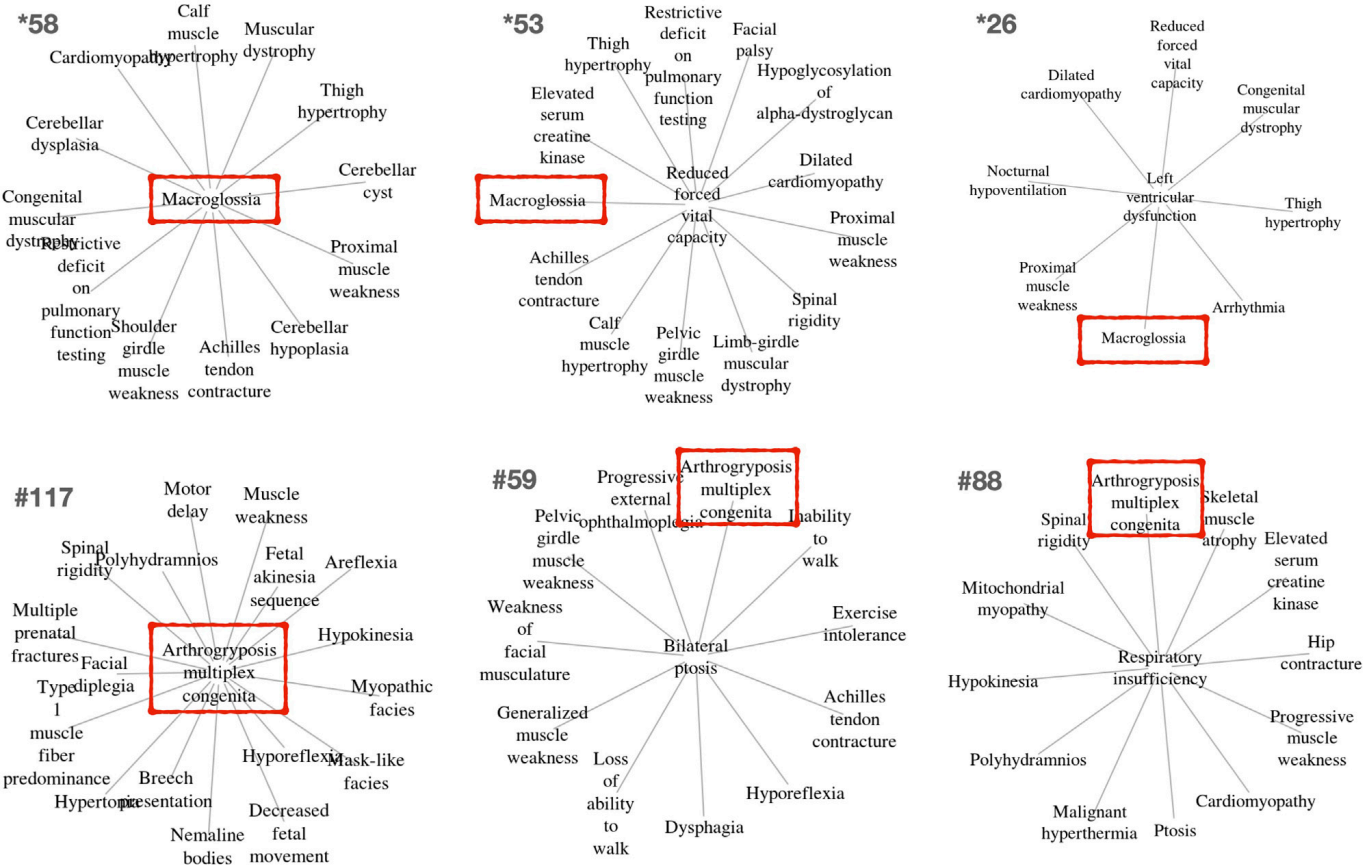
Example clusters including the unexpected but key macroglossia (HP:0000158) and arthrogryposis multiplex congenita (HP:0002804) phenotypes identified in OMIM and Orphanet, respectively. These phenotypes are marked in red boxes. The relevance of these phenotypes for differential diagnosis is further described in the text.

Other clusters provided by *PhenoClusters* contained more unusual groupings of phenotypes. Clinicians were surprised by the presence of ‘macroglossia’ (HP:0000158) and ‘arthogryposis’ (HP:0002804) (Figure 3). Macroglossia is usually associated with non-neuromuscular syndromes and in metabolic disorders such as glycogen storage disease type II (Pompe disease), mucopolysaccharidosis, oligosaccharidosis, mucolipidoses, sphingolipidoses and galactosidosis. However, macroglossia appeared as a hub node in the cluster *58 (Figure 3), strongly associated with highly representative NMD phenotypes, such as ‘congenital muscular dystrophy’ and ‘achilles tendon contracture’. This phenotype also appears in clusters *26, and *53, with macroglossia occupying a peripheral position (Figure 3). In macroglossia-containing clusters, 70%–80% of phenotypes show significant enrichment for the “protein *O*-linked mannosylation” biological process (GO:0035269), suggesting that the clinical observation of macroglossia in patients with NMDs may prompt practitioners to ask for *O*-linked mannosylation tests. This contrasts with cluster *211 (Supplementary Material S1) that also contains congenital muscular dystrophy and ankle flexion contracture, as well as ‘increased endomysial connective tissue’, but not macroglossia, the functions for this cluster being ‘focal adhesion’ (hsa04510) and ‘ECM proteoglycans’ (R-HSA-3000178) but not “protein *O*-linked mannosylation”. Hence, cluster *211 seems to be related to processes involved in physically connecting (‘focal adhesion’) cells to the extracellular matrix (‘ECM proteoglycans’). Therefore, functionally related clusters may help to discriminate disorders presenting with the macroglossia phenotype from other muscular dystrophies caused by the disruption of other cell functions. This finding shows the potential clinical utility of our approach to identify phenotypes whose presence alongside a given set of other phenotypes can indicate distinct underlying processes, with implications for diagnosis and treatment.

‘Arthogryposis’ represents another phenotype of potential use for NMD diagnosis (Figure 3). Arthrogryposis multiplex congenita consists of contractures in multiple body areas (Ambegaonkar et al., 2011). Cluster #117 shows a range of clinical phenotypes with arthrogryposis as the central node (Figure 3), while #59 and #88 are cases where this phenotype appears in a peripheral position. Interestingly, cluster #115 (Supplementary Material S2) presents almost all phenotypes observed in cluster #117, except arthogryposis. Hence, it can be inferred that when arthogryposis is present, the pathways associated with the majority of phenotypes in the cluster are more specific to muscle fibre-related processes including contraction dysfunction. However, when arthogryposis is absent, the cluster phenotypes share additional pathways related to cardio-myopathies and the regulation of pseudopodium assembly, indicating a broader aetiology in the genetic systems involved. As such, the presence or absence of arthogryposis alongside other symptoms may help inform NMD diagnosis and treatment, by indicating distinct underlying processes.

### 3.8 Research and Clinical Application: Clarifying Gene Involvement

The genes underlying the enriched functions were further investigated. It was noted that some of them occur in multiple clusters but are linked to different functions depending on the affected phenotypes. For example, *DAG1* is a pivotal component of the dystrophin-glycoprotein complex and its dysfunction is related to many muscular dystrophies, amongst other diseases (Durbeej et al., 1998; Sciandra et al., 2003). It can be considered a pleiotropic gene due to its role in glycosylation, a process which is involved in myriad cellular functions. For NMDs, it seems that glycosylation pathways are affected when *DAG1* appears in clusters *1, #29 and #113, in agreement with previous studies (Barresi and Campbell, 2006). However, the involvement of *DAG1* in clusters *198, #2, #144, #112, #103, #118, #11, #89 and #63 (Supplementary Material S1, S2), appears to be related to cardiomyopathy (Michele et al., 2009), while it is more focused on ‘ECM proteoglycan components’ in cluster *211. Hence, depending on the cluster in which *DAG1* appears, it seems to be involved in different pathways and functions, showing how *PhenoClusters* facilitates the identification of genes with multiple putative roles in NMDs depending on context.

Other clusters suggest that members of the solute carrier (SLC) family can perform many roles within NMDs. SLCs mediate the transport of a wide range of essential nutrients and metabolites, performing many different functions in cells and tissues (Zhang et al., 2019). SLC proteins were found to be associated with nutrient supply in cluster #93; metabolic transformation in clusters *85, *128, and *218; energy homeostasis in cluster *138; oxidative stress in cluster *205; and neurological regulation in clusters *97 and *166. These findings show how the phenotypic components of the functionally coherent clusters defined using *PhenoClusters* can point to the disruption of different cell functions in relation to NMDs involving genes from the SLC protein family.

## 4 Discussion

### 4.1 PhenoClusters Generates Reproducible Results and can be Extended to Other Diseases

As the use of bioinformatics analysis becomes more routine within biomedicine, it is crucial that published studies are accompanied by workflows that allow the analysis to be recorded and reported in a reproducible manner that can be applied to similar datasets, if applicable. *PhenoClusters* is based on phenotype co-occurrence, incorporating randomisation using matched non-NMD phenotypes to generate control datasets and including literature co-mention for validation of the approach. It also uses known NMD-causing genes to provide functional support to the co-occurring phenotypes. It produces a HTML report that can be easily interpreted, following the proposed Reproducible Research System (Mesirov, 2010; Piccolo and Frampton, 2016). Although there are many articles in the bioinformatics and systems biology fields presenting modular software tools and packages, in many cases code and data are not provided, despite the importance of reproducible research in health sciences (Harris et al., 2018). As it is essential for the present study, the necessary code to conduct the workflow as described in Figure 1 has been provided in full. Since the analysis starts by downloading diseases from OMIM and Orphanet, it can be adapted to study phenotype/gene/function co-occurrences in other diseases. *PhenoClusters* is thus an example of the re-analysis of previously published data using a new approach that produces new results and confirms already known facts. As such, it has achieved the goals of reproducible research (Mesirov, 2010; Piccolo and Frampton, 2016) in the health sciences (Harris et al., 2018) and exploiting already published results (Kovalevskaya et al., 2016).

### 4.2 Network Analyses Should Rely on More Than One Database

*PhenoClusters* did not mix data from OMIM and Orphanet based on two main facts. The first one is that OMIM and Orphanet are populated in different ways. For example, the variability of the phenotypic series of OMIM was high, with PS253600 (muscular dystrophy, limb-girdle, autosomal recessive) presenting 28 entries, while PS118220 (Charcot-Marie-Tooth disease) has 70 entries. The second one is that OMIM was searched with somewhat arbitrary keywords, whilst Orphanet NMDs were obtained based on its ontology structure. As a result, there are more OMIM-specific phenotypes and less Orphanet-specific phenotypes (Figure 2), and fewer functionally coherent clusters in OMIM than in Orphanet (Table 6). Besides these discrepancies, both databases rendered a similar ratio of shared phenotype pairs (Table 3) and the same top specific phenotype for NMDs, ‘elevated serum creatine kinase’ (Supplementary Material S5), in agreement with the importance of the creatine kinase test often used in NMD diagnosis (Thavorntanaburt et al., 2018). Other common phenotypes include muscle weakness, contractures, altered gait, functional difficulties, and respiratory issues, in agreement with recognised phenotypes for these diseases (Norwood et al., 2009). However, the interesting phenotypes ‘macroglossia’ and ‘arthogryposis’ (Figure 3) emerged from database-specific group of phenotypes. In any case, despite the mentioned discrepancies, it is striking that, when the phenotypes were associated with genes and functions, there was high overlap (Table 4, Supplementary Material S6). Therefore, we believe it is preferable to use the information from OMIM and Orphanet separately and to compare to contrast the results.

Co-mention validation in Table 5 revealed that even though GO contains many terms to describe biological functions and Reactome contains more pathway descriptions than KEGG, 2-fold more phenotype-KEGG pathway pairs (percentage-wise) were validated by literature co-mention. Moreover, when comparing functional annotation between OMIM and Orphanet, a proportionally larger overlap was found between the two resources for KEGG pathways (Supplementary Material S6). These findings supports our previous finding that KEGG produces better bioinformatic models in genetic algorithms for clinical diagnosis and prognosis (Luque-Baena et al., 2014), as well as for the outcome of diseases (Urda et al., 2018). Hence, KEGG pathways should always be considered in functional analyses.

### 4.3 Co-Occurrences and Associations Appear Consistent

Due to the etiological heterogeneity of NMDs, each disease is defined by its own set of clinical phenotypes. Phenotypes serve to understand life and disease, but it is not always easy to translate them to molecular mechanisms and vice versa (Yu et al., 2016). Taking advantage of the huge amount of data available in databases, bipartite networks (phenotype-disease, disease-gene) have been used in biomedicine to model factors that influence human diseases, explore their molecular complexity, reveal novel molecular relationships and disease susceptibility genes, uncover the biological significance of disease-associated mutations (Pavlopoulos et al., 2018) and, more recently, discover phenotype/disease clusters that can predict protein-phenotype associations and reveal the underlying mechanisms that link them (Ren et al., 2020). Bipartite gene-disease network analysis was revolutionised with the diseasome and related studies (Goh et al., 2007) which showed that genes associated with similar disease phenotypes have a higher propensity to interact physically with each other, forming distinct disease-specific functional modules, and that disorders tend to form clusters on the basis of similar pathophysiology (Oti and Brunner, 2007). Other studies revealed that phenotypes and gene co-regulation accurately predict unknown disease-gene relations (Deelen et al., 2019) since genes causing a specific disease or disease symptom often have similar molecular functions or are involved in the same biological process or pathway.

With all these facts in mind, the systems biology approach implemented in *PhenoClusters* lies in bipartite and tripartite networks (Modules B and C in Figure 1) to extract co-occurring phenotypes in NMDs (Supplementary Material S3, S4) that were consistent with i) clinical histories (Norwood et al., 2009) (Table 7), ii) creatine kinase tests (Moghadam-Kia et al., 2016; Thavorntanaburt et al., 2018) (Supplementary Material S5), iii) proximal or distal muscular weakness (McDonald, 2012; Mukherjee et al., 2019), and iv) postural instability and joint contracture (Kornegay et al., 2012), as usually found in the clinical history of NMD patients. The genes significantly associated with NMDs by *PhenoClusters* were largely expressed in neuronal and/or muscle-related tissues. For those that were not, it is tempting to speculate that this is due to incomplete information in The Human Protein Atlas, however it may also indicate genes involved in development or other regulatory processes that lead to the manifestation of the NMD phenotypes. There is currently much interest in the relationship between tissue/cell-type specificity and disease progression (Hekselman and Yeger-Lotem, 2020). Functional enrichment of the NMD-associated genes was used to provide phenotype-function associations that were then combined with the co-occurrent phenotype communities to produce functionally coherent clusters. Gene-to-function translation reduced the degrees of freedom of the analysis (several genes were required to assign a significantly enriched function to a phenotype), increasing statistical power and allowing the detection of shared functions between phenotypes, even if the underlying genes differ. The comparison of NMD-specific associations with randomised data in Tables 3, 4, 6 demonstrates that the findings were well-founded, as discussed below.

### 4.4 Co-Mention in Abstracts is a Valuable Approach Validation

There is an absence of gold-standard datasets for phenotypic relationships, particularly for rare diseases. As such, Ren et al. (2020) applied systems biology methods to obtain phenotype/disease clusters to seed machine learning models to predict protein-phenotype associations without any objective validation, assuming that they were relevant since authors can find biological support for some of the clusters. Here, abstract co-mention was proposed as a source of external validation for phenotype-phenotype and phenotype-function pairing approach. There were far fewer co-mentions for randomly paired terms than for specific pairs (Table 5), even though only a small fraction of phenotype-phenotype and phenotype-function pairs (<10 % in the second) showed significant co-mention in the literature. This is likely due to a combination of the following reasons: 1) term relationship existed, although not yet described in literature; 2) the co-mention appeared in the main body, but not in abstracts, of articles; 3) phenotypes were co-mentioned with genes rather than functions; and 4) abstract co-mention existed, but using slightly different terms or natural language synonyms that escape the search. In any case, these issues also apply to the randomised/non-NMD associations. In conclusion, although the abstract co-mention is not perfect, the fact that the amount of significant literature co-mention was much higher for NMD-specific pairs than controls (Table 5) shows it to provide suitable validation of the described approach and that real relationships were found.

### 4.5 Functionally Related Clusters Provide Insight Into NMDs

Many *PhenoClusters* clusters are in clear agreement with current medical knowledge about the relationships between phenotype comorbidity, NMDs, their pathophysiology and genetic causes (Table 7). This serves to further support the bioinformatic approach presented here. Regarding differential diagnosis and treatment, the clusters for which the presence/absence of a specific phenotype (Figure 3) could change the nature of the associated functions are particularly interesting: these have potential applications for differential diagnosis and treatment selection. As such, ‘macroglossia’ can be used to identify *O*-mannosylation alterations in a given NMD dysfunction (Spence et al., 2002; Martin, 2007; Moore and Winder, 2010; Dobson et al., 2013; Goody et al., 2015), while ‘arthogryposis’ (Ambegaonkar et al., 2011) may facilitate NMD diagnosis in other situations, as its presence may indicates that the disease is more specific to muscle fibres and contraction dysfunction.

The advantages of using functions rather than genes only is particularly evident in the findings related to pleiotropic genes and gene families involved in multiple functions. *DAG1* is involved in many diseases because it participates in glycosylation, whose malfunction affects many cellular processes and can produce several NMDs (Durbeej et al., 1998; Sciandra et al., 2003). However, its specific involvement in the proteoglycan components of the ECM seems to be related to cardiomyopathy. Additionally, despite the clear relevance to health and disease of the 190 different SLCs found mutated in human diseases, it is considered an understudied family (César-Razquin et al., 2015). Functionally related clusters from *PhenoClusters* reveal which particular role can be putatively assigned to the present members of the SLC family. Taken together, these results encourage future research lines directed to systematically exploit co-occurrence and clusters of co-occurrent phenotypes and functions in other diseases, as well as further study of a number of presented clusters to obtain more details on the functional implications.

### 4.6 Clinical Implications of Clusters

It has been shown that functionally coherent clusters have potential utility in NMDs in terms of better understanding clinical presentation in these diseases and obtaining clues as to the underlying molecular genetic mechanisms. The former has important implications for diagnosis and patient classification; the latter will help researchers better understand these diseases and search for potential new therapeutic targets.

Their potential for diagnosis is clear: given a patient that presents a number of phenotypes corresponding to one of the clusters, one can make inferences about other clinical phenotypes that the patient may also suffer and that should be tested for. This will help obtain a better clinical profile for the patient, aiding diagnosis. Moreover, genes and functional terms associated with the same cluster can be of use for guiding genetic diagnosis and indicate further pathological examination. For example, multiple clusters in the peripheral neuropathy group in Table 7 contain phenotypes associated with myelination and axon ensheathment related genes (Zhou and Notterpek, 2016; Kamil et al., 2019). As such, given a patient showing phenotypes belonging to one of these clusters, but for whom full diagnosis has not yet been achieved, the clinician could refer the patient for the appropriate assays, such as MRI, to look for demyelination and guide genetic studies to focus on related genes.

A further clinical application is related to differential diagnosis based on the phenotypic profile presented by a patient. The presence/absence of phenotypes ‘macroglossia’ and ‘arthogryposis’ (Figure 3) has been thoroughly explained as examples of phenotypes indicating differing underlying mechanisms that lead to disease manifestation. However, many other examples undoubtedly exist among the clusters presented in Supplementary Material S1, S2.

Nevertheless, there are several limitations in our bioinformatic approach, largely related to the external databases from which the information is acquired. OMIM and Orphanet are both incomplete resources: there are undoubtedly multiple diseases suffered by people around the world whose description and genetic basis have not made their way into these databases. This means that we are likely to miss co-occurrent phenotypes as the diseases in which they co-occur have not yet been added. Similarly, the resources used for functional enrichment analysis, used to ascribe function to the NMD phenotypes in this study, are also incomplete. We do not yet know the function of all genes in the genome, and this has effects on the enrichment procedures. As these resources improve, the potential of our workflow to find clusters of co-occurrent phenotypes and fully characterise their underlying basis will no doubt improve with them. However, regarding the information of clusters, this approach can only extract functions from genes known to be involved in NMDs, and cannot hypothesise about other genes even if they are working in the same pathway. And as already mentioned above, there is a limitation concerning literature co-mention validation (Table 5), as a higher proportion of confirmed pairs it would be desirable.

In conclusion, our approach and the resultant functionally coherent clusters of NMD phenotypes (Supplementary Material S1, S2) can 1) relate phenotype co-occurrence across NMDs to the underlying genes and mechanisms involved in the NMD (or other diseases); 2) provide clinicians with hints about clinical tests to produce a more reliable diagnosis based on the presence or absence of some specific phenotypes that have not yet been reported by the patient or the clinician, in the comorbidity context of the patient; 3) give researchers clues to perform new experiments to discover the underlying biological mechanisms of a disease; and 4) help in selecting optimal treatment. Therefore, *PhenoClusters* can be considered a new tool for more accurate diagnosis and an advance towards personalised medicine for NMDs.

## Data Availability

The original contributions presented in the study are included in the article/Supplementary Material, further inquiries can be directed to the corresponding author.

## Abbreviations

ECM, extracellular matrix; GO, gene ontology; HPO, human phenotype ontology; HyI, hypergeometric index; KEGG, kyoto encyclopedia of genes and genomes; MD, muscular dystrophy; NMD, neuromuscular disease; non-NMD, non-neuromuscular disease; OMIM, online Mendelian inheritance in man; PMID, pubMed identifier; SLC, solute carrier.
