# Supplementary material for: Decoding Neuromuscular Disorders Using Phenotypic Clusters Obtained From Co-Occurrence Networks
Source: Front Mol Biosci. 2021 Apr 19;8:635074. doi: 10.3389/fmolb.2021.635074 (PMC8147726; doi:10.3389/fmolb.2021.635074)

# *Supplementary Material (Díaz-Santiago et al.)*

## SUPPLEMENTARY DATA 1

**Complete HTML report saved by *PhenoCluster* for diseases from OMIM database.** Complete HTML report named *Sup1-omim_report.html* (that has been compressed as `Sup1-omim_report.html.zip`) where one can find

1. the database and the type of disease (in this case, NMDs);
2. the keywords used to retrieve NMDs;
3. the summary of diseases, phenotypes and genes related to NMDs in OMIM and those that have the three data; (disease, phenotype, gene) in HPO (Table 1);
4. the total number of phenotype pairs and only the significant pairs (Table 2);
5. the significant co-occurring phenotype pairs after the network analysis (Fig. 1 and Table 3);
6. the PubMed co-mention validation (Fig. 2 and Table 4);
7. the number of genes, KEGG, GO and Reactome terms before (Table 5) and after (Figs. 3, 5 and 5, and Tables 6, 7 and 8) the tripartite network analysis; and
8. the number of co-occurring phenotype clusters in NMDs and non-NMDs (Fig. 6 and Table 9) and the functionally coherent clusters (Fig. 7 and Table 10) using 70 % and 50 % of coherence.

## SUPPLEMENTARY DATA 2

**Complete HTML report saved by *PhenoCluster* for diseases from Orphanet database.** Complete HTML report named *Sup2-orphanet_report.html* (that has been compressed as `Sup2-orphanet_report.html.`[illegible] where one can find

1. the database and the type of disease (in this case, NMDs);
2. the ORDO term used to retrieve NMDs;
3. the summary of diseases, phenotypes and genes related to NMDs in OMIM and those that have the three data; (disease, phenotype, gene) in HPO (Table 1);
4. the total number of phenotype pairs and only the significant pairs (Table 2);
5. the significant co-occurring phenotype pairs after the network analysis (Fig. 1 and Table 3);
6. the PubMed co-mention validation (Fig. 2 and Table 4);
7. the number of genes, KEGG, GO and Reactome terms before (Table 5) and after (Figs. 3, 5 and 5, and Tables 6, 7 and 8) the tripartite network analysis; and
8. the number of co-occurring phenotype clusters in NMDs and non-NMDs (Fig. 6 and Table 9) and the functionally coherent clusters (Fig. 7 and Table 10) using 70 % and 50 % of coherence.

## SUPPLEMENTARY DATA 3

**Complete list of phenotypes typical for NMDs selected in OMIM database.** The HPO code, its description as well as the counts in NMDs and non-NMDs, together with the corresponding P-value, are given. The file format is tab-separated values (CSV). The file name is `Sup3-OMIM_phenotypes.csv`.

## SUPPLEMENTARY DATA 4

**Complete list of phenotypes typical for NMDs selected in Orphanet database.** The HPO code, its description as well as the counts in NMDs and non-NMDs, together with the corresponding P-value, are given. The file format is tab-separated values (CSV). The file name is `Sup4-Orphanet_phenotypes.csv`.

## Supplementary Data 5

**Top ten typical NMD phenotypes in the OMIM (Suppl. Data 3) and Orphanet (Supp. Data 4) databases.** The occurrence (counts) of each phenotype in NMDs and non-NMDs is given alongside the *P*-value for the one-sided Fisher's exact test. The first phenotype has been highlighted in bold since it is the same for both datasets.

| HPO code | Phenotype | Counts in NMDs | Counts in non-NMDs | *P*-value |
|---|---|---|---|---|
| *From OMIM database* | | | | |
| HP:0003236 | **Elevated serum creatine kinase** | 124 | 59 | 1.841099e-96 |
| HP:0001284 | Areflexia | 110 | 58 | 2.162695e-82 |
| HP:0001265 | Hyporeflexia | 117 | 100 | 4.686633e-74 |
| HP:0002460 | Distal muscle weakness | 81 | 18 | 3.014901e-73 |
| HP:0003701 | Proximal muscle weakness | 73 | 20 | 2.369912e-63 |
| HP:0002936 | Distal sensory impairment | 74 | 29 | 2.761166e-59 |
| HP:0003560 | Muscular dystrophy | 60 | 7 | 5.018812e-59 |
| HP:0003693 | Distal amyotrophy | 72 | 27 | 2.756548e-58 |
| HP:0003677 | Slow progression | 90 | 85 | 8.981719e-54 |
| HP:0010628 | Facial palsy | 63 | 34 | 4.818952e-46 |
| *From Orphanet database* | | | | |
| HP:0003236 | **Elevated serum creatine kinase** | 40 | 23 | 3.047233e-35 |
| HP:0003551 | Difficulty climbing stairs | 20 | 5 | 7.287806e-21 |
| HP:0003457 | EMG abnormality | 25 | 17 | 7.380621e-21 |
| HP:0003458 | EMG: myopathic abnormalities | 25 | 18 | 1.676727e-20 |
| HP:0008981 | Calf muscle hypertrophy | 17 | 5 | 2.290024e-17 |
| HP:0002515 | Waddling gait | 19 | 13 | 5.854754e-16 |
| HP:0003557 | Increased variability in muscle fiber diameter | 17 | 9 | 2.199215e-15 |
| HP:0003198 | Myopathy | 36 | 24 | 7.730904e-15 |
| HP:0003805 | Rimmed vacuoles | 13 | 2 | 1.117866e-14 |
| HP:0003391 | Gowers sign | 13 | 2 | 1.117866e-14 |

## Supplementary Data 6

**Common and distinct functions (GO, KEGG and Reactome) in OMIM and Orphanet described in Table 4 of the main manuscript.**

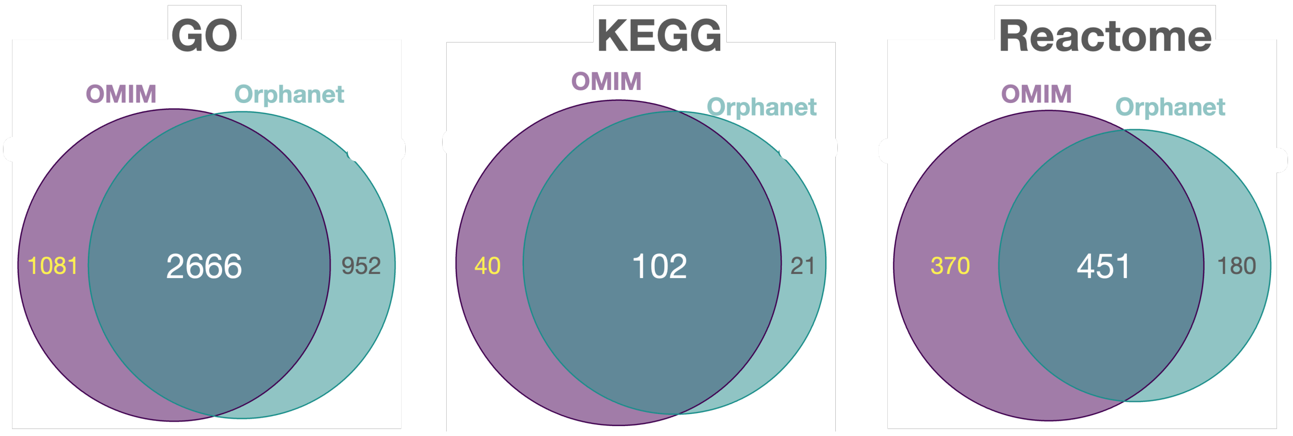

Supplement: Supplementary file 1 [file DataSheet1.zip › SupplMaterial-legends.pdf]
