# Supplementary material for: Decoding Neuromuscular Disorders Using Phenotypic Clusters Obtained From Co-Occurrence Networks
Source: Front Mol Biosci. 2021 Apr 19;8:635074. doi: 10.3389/fmolb.2021.635074 (PMC8147726; doi:10.3389/fmolb.2021.635074)
Supplement: Supplementary file 1 [file DataSheet1.zip › Sup1-omim_report.html]

omim\_report\_template.utf8


# OMIM Neuromuscular Diseases Study

## Step one: Datasets of NMDs, genes, phenotypes and typical NMD phenotypes

OMIM offer a brief description of diseases.  
By conducting a literature review, we can stabilize a number of keywords to filter the OMIM diseases and get a certain number of neuromuscular diseases:  
- MUSCULAR DYSTROPHY.  
- MYOPATHY.  
- MYASTHENIC.  
- MYASTHENIA.  
- NEUROPATHY.  
- AMYOTROPHIC LATERAL SCLEROSIS.  
- SPINAL MUSCULAR ATROPHY.  
- SPINAL AND BULBAR MUSCULAR ATROPHY.  
- MYOTONIA.  
- PERIODIC PARALYSIS.  
- MYOTONIC DYSTROPHY.  
- MITOCHONDRIAL CYTOPATHY.  
- NECROTIZING ENCEPHALOMYELOPATHY.  
- MITOCHONDRIAL DNA DEPLETION.

Once we have the list of neuromuscular diseases, we count the frequency of phenotypes in neuromuscular and non neuromuscular diseases. With these data, we can perform a fisher test analysis to find those phenotypes which are more represented in neuromusculare diseases rather than in non neuromuscular diseases.

**Table 1**: Preprocesing data summary.

| Metric | Value |
| --- | --- |
| Total\_OMIM\_diseases | 26943 |
| Total\_OMIM\_diseases\_in\_HPO | 5189 |
| Total\_OMIM\_phenotypes | 6385 |
| Total\_OMIM\_genes | 4015 |
| OMIM\_NMD | 556 |
| Total\_OMIM\_NMD\_in\_HPO | 424 |
| Total\_OMIM\_neuromuscular\_phenotypes | 335 |

## Step 2: Bipartite network to obtain phenotype-phenotype associations

**Table 2:** Bipartite results general summary.

| Metric | Value |
| --- | --- |
| Total\_phenotype\_pairs | 455379 |
| Significant\_phenotype\_pairs | 125720 |

In the following plot we can see the number of pairs which form the subnetwork with a HyI >= 2.

**Figure 1:** Number of phenotype pairs with a HyI >= 2.

**Table 3:** Number of phenotype pairs with a HyI >= 2.

| Type | Pairs Number |
| --- | --- |
| NMDs | 4579 ± NA |
| non\_NMDs | 654.7 ± 46.437 |

### Validating phenotype-phenotye associations by PubMed co-mention

**Figure 2** Comention pvalue density plot.

**Table 4** Number of co-occurrent pairs.

| Type | Pairs Number |
| --- | --- |
| NMDs | 1173 ± NA |
| non\_NMDs | 333.08 ± 34.633 |

## Step 3: Tripartite network to obtain phenotype-function pairs

In this step we will get phenotype-genes pairs. Through a functional assignment of these genes we can get phenotype-function pairs.

**Table 5** Tripartite results general summary.

| Metric | Value |
| --- | --- |
| Number\_of\_genes | 4015 |
| Number\_of\_phen2gene\_pairs\_HyI\_2 | 34139 |
| Number\_of\_genes\_HyI\_2 | 3870 |
| NMD\_genes\_HyI\_2 | 1078 |
| Total\_kegg\_terms\_0.05 | 214 |
| Total\_go\_terms\_0.05 | 6398 |
| Total\_reactome\_terms\_0.05 | 1397 |
| NMD\_kegg\_0.05 | 142 |
| NMD\_go\_0.05 | 3747 |
| NMD\_reactome\_0.05 | 821 |

### Validating phenotype-function associations by PubMed co-mention

#### KEGG

**Figure 3** Significant phenotype-KEGG pairs

**Table 6** Significant phenotype-KEGG pairs.

| Type | Co-occurrent pairs | No co-occurrent pairs |
| --- | --- | --- |
| phen2kegg\_omim | 1858 ± NA | 15376 ± NA |
| rdm\_phen2kegg\_omim | 626.72 ± 22.148 | 16607.28 ± 22.148 |

#### GO

**Figure 4** Significant phenotype-GO pairs.

**Table 7** Significant phenotype-GO pairs.

| Type | Co-occurrent pairs | No co-occurrent pairs |
| --- | --- | --- |
| phen2go\_omim | 26841 ± NA | 520492 ± NA |
| rdm\_phen2go\_omim | 10814.444 ± 80.538 | 536512.556 ± 82.518 |

##### Reactome

**Figure 5** Significant phenotype-Reactome pairs.

**Table 8** Significant phenotype-Reactome pairs.

| Type | Co-occurrent pairs | No co-occurrent pairs |
| --- | --- | --- |
| phen2reactome\_omim | 3278 ± NA | 74857 ± NA |
| rdm\_phen2reactome\_omim | 1001.206 ± 166.295 | 78589.433 ± 12828.536 |

## Step 4: Cluster Analysis

With linkcomm R package we obtain a number of clusters for each network. In the following plots we show some results of the clustering process. By one hand we have measured the number of clusters obtained and the average number of nodes of each cluster.

**Figure 6** Cluster analysis summary.

**Table 9** Cluster analysis summary.

| Type | Cluster Number | Average Cluster size |
| --- | --- | --- |
| NMDs | 231 ± NA | 16.749 ± NA |
| non\_NMDs | 94.98 ± 9.571 | 5.551 ± 0.573 |

### Functionally-coherent clusters of phenotypes in NMDs

**Figure 7** Functional coherent clusters.

**Table 10** Functional coherent clusters summary.

| Type | Cluster with 70 coherence\_0.05 | Cluster with 50 coherence\_0.05 |
| --- | --- | --- |
| NMDs | 40 ± NA | 131 ± NA |
| non\_NMDs | 41.21 ± 7.718 | 78.43 ± 10.145 |

## Clusters Details

In this section we include OMIM neuromuscular cluster’s details.

---


---


---

# Cluster \*55

| HPO | Cluster | Description |
| --- | --- | --- |
| HP:0003323 | 55 | Progressive muscle weakness |
| HP:0003688 | 55 | Cytochrome C oxidase-negative muscle fibers |
| HP:0001924 | 55 | Sideroblastic anemia |
| HP:0009055 | 55 | Generalized limb muscle atrophy |

| Cluster | Cell function | Description | Phenotype\_coverage | Genes |
| --- | --- | --- | --- | --- |
| 55 | GO:0000959 | mitochondrial RNA metabolic process | 100 | TRNT1, TWNK, YARS2, PUS1 |
| 55 | GO:0140053 | mitochondrial gene expression | 75 | YARS2, LARS2, TWNK, PUS1, TRNT1, FASTKD2 |

---


---


---

# Cluster \*146

| HPO | Cluster | Description |
| --- | --- | --- |
| HP:0003198 | 146 | Myopathy |
| HP:0001427 | 146 | Mitochondrial inheritance |
| HP:0000576 | 146 | Centrocecal scotoma |
| HP:0003752 | 146 | Episodic flaccid weakness |

| Cluster | Cell function | Description | Phenotype\_coverage | Genes |
| --- | --- | --- | --- | --- |
| 146 | hsa05010 | Alzheimer’s disease | 100 | COX1, NDUFS4, SDHA, COX8A, COX6B1, COX3, CACNA1S, CYTB, ATP6, COX2 |
| 146 | hsa04260 | Cardiac muscle contraction | 100 | COX1, COX8A, COX6B1, COX3, CACNA1S, CYTB, COX2 |
| 146 | hsa00190 | Oxidative phosphorylation | 75 | COX10, ND2, ND1, COX1, ND5, SDHA, COX15, NDUFS4, ND4L, COX8A, COX6B1, ND4, COX3, CYTB, ND6, ATP6, COX2 |
| 146 | hsa05016 | Huntington’s disease | 75 | COX1, NDUFS4, SDHA, COX8A, COX6B1, COX3, CYTB, ATP6, COX2 |
| 146 | hsa05012 | Parkinson’s disease | 75 | ND2, ND1, COX1, ND5, SDHA, NDUFS4, ND4L, COX8A, COX6B1, ND4, COX3, CYTB, ND6, ATP6, COX2 |
| 146 | GO:0006979 | response to oxidative stress | 75 | COX1, ND6, ND1, ND5 |
| 146 | GO:0006120 | mitochondrial electron transport, NADH to ubiquinone | 75 | NDUFS4, ND4, ND6, ND4L, ND5, ND2, ND1 |
| 146 | GO:0046677 | response to antibiotic | 75 | ND4, ND6, ND5, CYTB, OPA1 |
| 146 | GO:0006123 | mitochondrial electron transport, cytochrome c to oxygen | 75 | COX10, COX1, COX3, COX8A, COX2, COX15, COX6B1 |
| 146 | GO:0008535 | respiratory chain complex IV assembly | 75 | SCO1, COX10, SURF1, PET100, COX20, COX14, COX15, BCS1L, COX3 |
| 146 | GO:0032981 | mitochondrial respiratory chain complex I assembly | 75 | NDUFS4, ND4, ND6, ND5, ND2, BCS1L, ND1 |
| 146 | GO:0055093 | response to hyperoxia | 75 | ATP6, CYTB |
| 146 | GO:0009205 | purine ribonucleoside triphosphate metabolic process | 75 | NDUFS4, COX6B1, ATP6, COX10, COX1, SURF1, ND1, SDHA, ND4, ND6, COX8A, ND4L, COX15, ND5, COX2, ND2, CYTB, OPA1, COX3 |
| 146 | GO:1902600 | proton transmembrane transport | 75 | COX6B1, COX10, ATP6, COX1, SURF1, ND4, COX8A, COX2, COX15, CYTB, COX3 |
| 146 | GO:0009167 | purine ribonucleoside monophosphate metabolic process | 75 | NDUFS4, COX6B1, ATP6, COX10, COX1, SURF1, ND1, SDHA, ND4, ND6, COX8A, ND4L, COX15, ND5, COX2, ND2, CYTB, COX3 |
| 146 | GO:0035094 | response to nicotine | 75 | ND4, ND6, CHRNB1 |
| 146 | R-HSA-163200 | Respiratory electron transport, ATP synthesis by chemiosmotic coupling, and heat production by uncoupling proteins. | 75 | ATP6, COX1, COX6B1, CYTB, ND5, NDUFS4, SDHA, ND6, ND4, COX14, COX20, COX2, SURF1, COX3, TACO1, ND2, ND1, COX8A, SCO1 |
| 146 | R-HSA-6799198 | Complex I biogenesis | 75 | NDUFS4, ND5, ND6, ND4, ND2, ND1 |
| 146 | R-HSA-1428517 | The citric acid (TCA) cycle and respiratory electron transport | 75 | ATP6, COX1, COX6B1, CYTB, ND5, NDUFS4, SDHA, ND6, ND4, COX14, COX20, COX2, SURF1, COX3, TACO1, ND2, ND1, COX8A, SCO1 |
| 146 | R-HSA-611105 | Respiratory electron transport | 75 | COX1, COX6B1, CYTB, ND5, NDUFS4, SDHA, ND6, ND4, COX14, COX20, COX2, SURF1, COX3, TACO1, ND2, ND1, COX8A, SCO1 |

---


---


---

# Cluster \*97

| HPO | Cluster | Description |
| --- | --- | --- |
| HP:0001612 | 97 | Weak cry |
| HP:0003554 | 97 | Type 2 muscle fiber atrophy |
| HP:0002033 | 97 | Poor suck |
| HP:0002715 | 97 | Abnormality of the immune system |
| HP:0000597 | 97 | Ophthalmoparesis |
| HP:0002872 | 97 | Apneic episodes precipitated by illness, fatigue, stress |
| HP:0002882 | 97 | Sudden episodic apnea |
| HP:0003397 | 97 | Generalized hypotonia due to defect at the neuromuscular junction |
| HP:0003402 | 97 | Decreased miniature endplate potentials |
| HP:0003403 | 97 | EMG: decremental response of compound muscle action potential to repetitive nerve stimulation |
| HP:0003473 | 97 | Fatigable weakness |
| HP:0003436 | 97 | Prolonged miniature endplate currents |
| HP:0003443 | 97 | Decreased size of nerve terminals |

| Cluster | Cell function | Description | Phenotype\_coverage | Genes |
| --- | --- | --- | --- | --- |
| 97 | GO:0007271 | synaptic transmission, cholinergic | 92.30769 | CHRNE, SLC5A7, CHRNA1, COLQ |
| 97 | GO:0007274 | neuromuscular synaptic transmission | 92.30769 | CHAT, CHRNE, SLC5A7, CHRNA1 |
| 97 | GO:0035094 | response to nicotine | 84.61538 | CHRNE, CHRNA1 |
| 97 | R-HSA-112315 | Transmission across Chemical Synapses | 84.61538 | CHRNA1, CHAT, CHRNE, SLC5A7 |
| 97 | R-HSA-622327 | Postsynaptic nicotinic acetylcholine receptors | 84.61538 | CHRNA1, CHRNE |
| 97 | R-HSA-181431 | Acetylcholine binding and downstream events | 84.61538 | CHRNA1, CHRNE |
| 97 | R-HSA-622323 | Presynaptic nicotinic acetylcholine receptors | 84.61538 | CHRNA1, CHRNE |
| 97 | R-HSA-112316 | Neuronal System | 84.61538 | CHRNA1, CHAT, CHRNE, SLC5A7 |
| 97 | R-HSA-264642 | Acetylcholine Neurotransmitter Release Cycle | 76.92308 | CHAT, SLC5A7 |

---


---


---

# Cluster \*3

| HPO | Cluster | Description |
| --- | --- | --- |
| HP:0009830 | 3 | Peripheral neuropathy |
| HP:0001265 | 3 | Hyporeflexia |
| HP:0001761 | 3 | Pes cavus |
| HP:0003693 | 3 | Distal amyotrophy |
| HP:0001178 | 3 | Ulnar claw |
| HP:0001284 | 3 | Areflexia |
| HP:0001765 | 3 | Hammertoe |
| HP:0002460 | 3 | Distal muscle weakness |
| HP:0002936 | 3 | Distal sensory impairment |
| HP:0003376 | 3 | Steppage gait |
| HP:0003380 | 3 | Decreased number of peripheral myelinated nerve fibers |
| HP:0003382 | 3 | Hypertrophic nerve changes |
| HP:0003383 | 3 | Onion bulb formation |
| HP:0003431 | 3 | Decreased motor nerve conduction velocity |
| HP:0003449 | 3 | Cold-induced muscle cramps |
| HP:0004336 | 3 | Myelin outfoldings |
| HP:0009027 | 3 | Foot dorsiflexor weakness |
| HP:0003481 | 3 | Segmental peripheral demyelination/remyelination |
| HP:0003387 | 3 | Decreased number of large peripheral myelinated nerve fibers |
| HP:0003484 | 3 | Upper limb muscle weakness |

| Cluster | Cell function | Description | Phenotype\_coverage | Genes |
| --- | --- | --- | --- | --- |
| 3 | GO:0042552 | myelination | 90 | SBF2, SH3TC2, MPZ, MTMR2, ARHGEF10, EGR2, PSAP, SOX10, FAM126A, NDRG1, PMP22, DHH |

---


---


---

# Cluster \*91

| HPO | Cluster | Description |
| --- | --- | --- |
| HP:0007240 | 91 | Progressive gait ataxia |
| HP:0004389 | 91 | Intestinal pseudo-obstruction |
| HP:0004395 | 91 | Malnutrition |
| HP:0003548 | 91 | Subsarcolemmal accumulations of abnormally shaped mitochondria |
| HP:0003688 | 91 | Cytochrome C oxidase-negative muscle fibers |
| HP:0003713 | 91 | Muscle fiber necrosis |
| HP:0006886 | 91 | Impaired distal vibration sensation |
| HP:0002403 | 91 | Positive Romberg sign |
| HP:0003434 | 91 | Sensory ataxic neuropathy |

| Cluster | Cell function | Description | Phenotype\_coverage | Genes |
| --- | --- | --- | --- | --- |
| 91 | GO:0071333 | cellular response to glucose stimulus | 88.88889 | TWNK, POLG |
| 91 | GO:0032042 | mitochondrial DNA metabolic process | 88.88889 | POLG2, POLG, TWNK |
| 91 | GO:0006264 | mitochondrial DNA replication | 88.88889 | POLG2, POLG, TWNK |
| 91 | GO:0034214 | protein hexamerization | 77.77778 | TWNK |
| 91 | GO:0006268 | DNA unwinding involved in DNA replication | 77.77778 | TWNK |
| 91 | GO:0055093 | response to hyperoxia | 77.77778 | POLG |
| 91 | GO:0006390 | mitochondrial transcription | 77.77778 | TWNK |

---


---


---

# Cluster \*193

| HPO | Cluster | Description |
| --- | --- | --- |
| HP:0003326 | 193 | Myalgia |
| HP:0003712 | 193 | Skeletal muscle hypertrophy |
| HP:0003552 | 193 | Muscle stiffness |
| HP:0003730 | 193 | EMG: myotonic runs |
| HP:0003740 | 193 | Myotonia with warm-up phenomenon |
| HP:0010548 | 193 | Percussion myotonia |
| HP:0012899 | 193 | Handgrip myotonia |
| HP:0025605 | 193 | Lid lag on downgaze |
| HP:0005348 | 193 | Inspiratory stridor |

| Cluster | Cell function | Description | Phenotype\_coverage | Genes |
| --- | --- | --- | --- | --- |
| 193 | GO:0003012 | muscle system process | 88.88889 | MYOT, CLCN1, TNFRSF1A, SCN4A, CCDC78, MSTN, CASQ1 |
| 193 | GO:0019226 | transmission of nerve impulse | 77.77778 | CLCN1, SCN4A |

---


---


---

# Cluster \*82

| HPO | Cluster | Description |
| --- | --- | --- |
| HP:0002398 | 82 | Degeneration of anterior horn cells |
| HP:0002522 | 82 | Areflexia of lower limbs |
| HP:0003445 | 82 | EMG: neuropathic changes |
| HP:0007269 | 82 | Spinal muscular atrophy |
| HP:0001308 | 82 | Tongue fasciculations |
| HP:0002378 | 82 | Hand tremor |
| HP:0003457 | 82 | EMG abnormality |

| Cluster | Cell function | Description | Phenotype\_coverage | Genes |
| --- | --- | --- | --- | --- |
| 82 | R-HSA-194441 | Metabolism of non-coding RNA | 85.71429 | SMN1, SMN2 |
| 82 | R-HSA-191859 | snRNP Assembly | 85.71429 | SMN1, SMN2 |

---


---


---

# Cluster \*138

| HPO | Cluster | Description |
| --- | --- | --- |
| HP:0008955 | 138 | Progressive distal muscular atrophy |
| HP:0003697 | 138 | Scapuloperoneal amyotrophy |
| HP:0007178 | 138 | Motor polyneuropathy |
| HP:0009063 | 138 | Progressive distal muscle weakness |
| HP:0009113 | 138 | Diaphragmatic weakness |
| HP:0010307 | 138 | Stridor |
| HP:0011349 | 138 | Abducens palsy |

| Cluster | Cell function | Description | Phenotype\_coverage | Genes |
| --- | --- | --- | --- | --- |
| 138 | GO:0006884 | cell volume homeostasis | 85.71429 | TRPV4, SLC12A6 |
| 138 | GO:0006971 | hypotonic response | 85.71429 | TRPV4, SLC12A6 |
| 138 | GO:0071472 | cellular response to salt stress | 85.71429 | TRPV4, SLC12A6 |
| 138 | GO:2000778 | positive regulation of interleukin-6 secretion | 71.42857 | TRPV4 |
| 138 | GO:0050891 | multicellular organismal water homeostasis | 71.42857 | TRPV4 |
| 138 | GO:0045599 | negative regulation of fat cell differentiation | 71.42857 | TRPV4 |
| 138 | GO:0043457 | regulation of cellular respiration | 71.42857 | TRPV4 |
| 138 | GO:0070509 | calcium ion import | 71.42857 | TRPV4 |
| 138 | GO:0097009 | energy homeostasis | 71.42857 | TRPV4 |
| 138 | GO:0030865 | cortical cytoskeleton organization | 71.42857 | TRPV4 |
| 138 | GO:0071639 | positive regulation of monocyte chemotactic protein-1 production | 71.42857 | TRPV4 |
| 138 | GO:0043117 | positive regulation of vascular permeability | 71.42857 | TRPV4 |
| 138 | GO:1901881 | positive regulation of protein depolymerization | 71.42857 | TRPV4 |
| 138 | GO:0010759 | positive regulation of macrophage chemotaxis | 71.42857 | TRPV4 |
| 138 | GO:0071609 | chemokine (C-C motif) ligand 5 production | 71.42857 | TRPV4 |
| 138 | GO:0047484 | regulation of response to osmotic stress | 71.42857 | TRPV4 |
| 138 | GO:0042538 | hyperosmotic salinity response | 71.42857 | TRPV4 |
| 138 | GO:0031122 | cytoplasmic microtubule organization | 71.42857 | TRPV4 |
| 138 | GO:0031532 | actin cytoskeleton reorganization | 71.42857 | TRPV4 |
| 138 | GO:0046785 | microtubule polymerization | 71.42857 | TRPV4 |
| 138 | GO:0090335 | regulation of brown fat cell differentiation | 71.42857 | TRPV4 |
| 138 | GO:0002024 | diet induced thermogenesis | 71.42857 | TRPV4 |
| 138 | GO:0031114 | regulation of microtubule depolymerization | 71.42857 | TRPV4 |
| 138 | GO:0060351 | cartilage development involved in endochondral bone morphogenesis | 71.42857 | TRPV4 |
| 138 | GO:0031112 | positive regulation of microtubule polymerization or depolymerization | 71.42857 | TRPV4 |
| 138 | R-HSA-3295583 | TRP channels | 71.42857 | TRPV4 |

---


---


---

# Cluster \*58

| HPO | Cluster | Description |
| --- | --- | --- |
| HP:0001638 | 58 | Cardiomyopathy |
| HP:0000158 | 58 | Macroglossia |
| HP:0003701 | 58 | Proximal muscle weakness |
| HP:0003560 | 58 | Muscular dystrophy |
| HP:0001321 | 58 | Cerebellar hypoplasia |
| HP:0002111 | 58 | Restrictive deficit on pulmonary function testing |
| HP:0003547 | 58 | Shoulder girdle muscle weakness |
| HP:0008981 | 58 | Calf muscle hypertrophy |
| HP:0001771 | 58 | Achilles tendon contracture |
| HP:0003741 | 58 | Congenital muscular dystrophy |
| HP:0007033 | 58 | Cerebellar dysplasia |
| HP:0002350 | 58 | Cerebellar cyst |
| HP:0003733 | 58 | Thigh hypertrophy |

| Cluster | Cell function | Description | Phenotype\_coverage | Genes |
| --- | --- | --- | --- | --- |
| 58 | GO:0035269 | protein O-linked mannosylation | 84.61538 | POMT1, RXYLT1, LARGE1, CRPPA, FKRP, POMGNT2, POMT2, FKTN |

---


---


---

# Cluster \*1

| HPO | Cluster | Description |
| --- | --- | --- |
| HP:0001302 | 1 | Pachygyria |
| HP:0001321 | 1 | Cerebellar hypoplasia |
| HP:0002500 | 1 | Abnormality of the cerebral white matter |
| HP:0002365 | 1 | Hypoplasia of the brainstem |
| HP:0007260 | 1 | Type II lissencephaly |
| HP:0031882 | 1 | Agyria |

| Cluster | Cell function | Description | Phenotype\_coverage | Genes |
| --- | --- | --- | --- | --- |
| 1 | GO:0035269 | protein O-linked mannosylation | 83.33333 | POMT1, TMTC3, LARGE1, RXYLT1, CRPPA, FKRP, B4GAT1, POMGNT2, POMT2, FKTN |
| 1 | GO:0060049 | regulation of protein glycosylation | 83.33333 | POMT2, POMT1, FKTN |
| 1 | R-HSA-5173105 | O-linked glycosylation | 83.33333 | LARGE1, DAG1, B4GAT1, POMT2, B3GALNT2, POMGNT2, POMGNT1, POMT1, POMK |
| 1 | R-HSA-3906995 | Diseases associated with O-glycosylation of proteins | 83.33333 | LARGE1, DAG1, B4GAT1, POMT2, POMGNT1, POMT1 |
| 1 | R-HSA-3781865 | Diseases of glycosylation | 83.33333 | LARGE1, DAG1, B4GAT1, POMT2, POMGNT1, POMT1 |

---


---


---

# Cluster \*89

| HPO | Cluster | Description |
| --- | --- | --- |
| HP:0003470 | 89 | Paralysis |
| HP:0002522 | 89 | Areflexia of lower limbs |
| HP:0002600 | 89 | Hyporeflexia of lower limbs |
| HP:0002601 | 89 | Paresis of extensor muscles of the big toe |
| HP:0003445 | 89 | EMG: neuropathic changes |
| HP:0009053 | 89 | Distal lower limb muscle weakness |

| Cluster | Cell function | Description | Phenotype\_coverage | Genes |
| --- | --- | --- | --- | --- |
| 89 | GO:0006986 | response to unfolded protein | 83.33333 | VAPB, HSPB1, HSPB8, HSPB3 |

---


---


---

# Cluster \*211

| HPO | Cluster | Description |
| --- | --- | --- |
| HP:0006466 | 211 | Ankle flexion contracture |
| HP:0003306 | 211 | Spinal rigidity |
| HP:0003741 | 211 | Congenital muscular dystrophy |
| HP:0007103 | 211 | Hypointensity of cerebral white matter on MRI |
| HP:0100297 | 211 | Increased endomysial connective tissue |
| HP:0003719 | 211 | Muscle mounding |

| Cluster | Cell function | Description | Phenotype\_coverage | Genes |
| --- | --- | --- | --- | --- |
| 211 | hsa04510 | Focal adhesion | 83.33333 | COL6A3, COL6A1, LAMA2, ITGA7, COL6A2, CAV3 |
| 211 | R-HSA-3000178 | ECM proteoglycans | 83.33333 | COL6A1, LAMA2, DAG1, COL6A2, ITGA7, COL6A3 |

---


---


---

# Cluster \*85

| HPO | Cluster | Description |
| --- | --- | --- |
| HP:0001605 | 85 | Vocal cord paralysis |
| HP:0009113 | 85 | Diaphragmatic weakness |
| HP:0009130 | 85 | Hand muscle atrophy |
| HP:0002877 | 85 | Nocturnal hypoventilation |
| HP:0030237 | 85 | Hand muscle weakness |

| Cluster | Cell function | Description | Phenotype\_coverage | Genes |
| --- | --- | --- | --- | --- |
| 85 | hsa04977 | Vitamin digestion and absorption | 80 | SLC52A3 |

---


---


---

# Cluster \*105

| HPO | Cluster | Description |
| --- | --- | --- |
| HP:0003555 | 105 | Muscle fiber splitting |
| HP:0009054 | 105 | Scapuloperoneal myopathy |
| HP:0000759 | 105 | Abnormal peripheral nervous system morphology |
| HP:0003715 | 105 | Myofibrillar myopathy |
| HP:0100303 | 105 | Muscle fiber cytoplasmatic inclusion bodies |

| Cluster | Cell function | Description | Phenotype\_coverage | Genes |
| --- | --- | --- | --- | --- |
| 105 | GO:0048747 | muscle fiber development | 80 | KLHL40, DYSF, TTN, FLNC |

---


---


---

# Cluster \*128

| HPO | Cluster | Description |
| --- | --- | --- |
| HP:0001762 | 128 | Talipes equinovarus |
| HP:0002751 | 128 | Kyphoscoliosis |
| HP:0003803 | 128 | Type 1 muscle fiber predominance |
| HP:0003044 | 128 | Shoulder flexion contracture |
| HP:0002047 | 128 | Malignant hyperthermia |

| Cluster | Cell function | Description | Phenotype\_coverage | Genes |
| --- | --- | --- | --- | --- |
| 128 | R-HSA-3560782 | Diseases associated with glycosaminoglycan metabolism | 80 | B3GALT6, HSPG2, SLC26A2, CHST3 |
| 128 | R-HSA-1793185 | Chondroitin sulfate/dermatan sulfate metabolism | 80 | B3GALT6, HSPG2, CHST3 |
| 128 | R-HSA-4420332 | Defective B3GALT6 causes EDSP2 and SEMDJL1 | 80 | B3GALT6, HSPG2 |
| 128 | R-HSA-1971475 | A tetrasaccharide linker sequence is required for GAG synthesis | 80 | B3GALT6, HSPG2 |

---


---


---

# Cluster \*219

| HPO | Cluster | Description |
| --- | --- | --- |
| HP:0003676 | 219 | Progressive |
| HP:0003737 | 219 | Mitochondrial myopathy |
| HP:0004395 | 219 | Malnutrition |
| HP:0000590 | 219 | Progressive external ophthalmoplegia |
| HP:0003548 | 219 | Subsarcolemmal accumulations of abnormally shaped mitochondria |
| HP:0003688 | 219 | Cytochrome C oxidase-negative muscle fibers |
| HP:0003434 | 219 | Sensory ataxic neuropathy |
| HP:0002579 | 219 | Gastrointestinal dysmotility |
| HP:0007103 | 219 | Hypointensity of cerebral white matter on MRI |
| HP:0004326 | 219 | Cachexia |

| Cluster | Cell function | Description | Phenotype\_coverage | Genes |
| --- | --- | --- | --- | --- |
| 219 | GO:0006264 | mitochondrial DNA replication | 80 | MGME1, POLG, RRM2B, TWNK, DNA2, POLG2 |
| 219 | GO:0032042 | mitochondrial DNA metabolic process | 70 | MGME1, POLG, RRM2B, TWNK, DNA2, TOP3A, POLG2 |
| 219 | R-HSA-73614 | Pyrimidine salvage | 70 | TK2, TYMP |
| 219 | R-HSA-73621 | Pyrimidine catabolism | 70 | TYMP |

---


---


---

# Cluster \*26

| HPO | Cluster | Description |
| --- | --- | --- |
| HP:0000158 | 26 | Macroglossia |
| HP:0011675 | 26 | Arrhythmia |
| HP:0003701 | 26 | Proximal muscle weakness |
| HP:0001644 | 26 | Dilated cardiomyopathy |
| HP:0005162 | 26 | Left ventricular dysfunction |
| HP:0002877 | 26 | Nocturnal hypoventilation |
| HP:0003741 | 26 | Congenital muscular dystrophy |
| HP:0032341 | 26 | Reduced forced vital capacity |
| HP:0003733 | 26 | Thigh hypertrophy |

| Cluster | Cell function | Description | Phenotype\_coverage | Genes |
| --- | --- | --- | --- | --- |
| 26 | GO:0035269 | protein O-linked mannosylation | 77.77778 | POMT1, LARGE1, CRPPA, FKRP, POMT2, FKTN |

---


---


---

# Cluster \*4

| HPO | Cluster | Description |
| --- | --- | --- |
| HP:0009830 | 4 | Peripheral neuropathy |
| HP:0002751 | 4 | Kyphoscoliosis |
| HP:0001171 | 4 | Split hand |
| HP:0001265 | 4 | Hyporeflexia |
| HP:0001761 | 4 | Pes cavus |
| HP:0003693 | 4 | Distal amyotrophy |
| HP:0003690 | 4 | Limb muscle weakness |
| HP:0001178 | 4 | Ulnar claw |
| HP:0001284 | 4 | Areflexia |
| HP:0002936 | 4 | Distal sensory impairment |
| HP:0003376 | 4 | Steppage gait |
| HP:0003380 | 4 | Decreased number of peripheral myelinated nerve fibers |
| HP:0003383 | 4 | Onion bulb formation |
| HP:0003431 | 4 | Decreased motor nerve conduction velocity |
| HP:0003449 | 4 | Cold-induced muscle cramps |
| HP:0004336 | 4 | Myelin outfoldings |
| HP:0009027 | 4 | Foot dorsiflexor weakness |
| HP:0011096 | 4 | Peripheral demyelination |
| HP:0003387 | 4 | Decreased number of large peripheral myelinated nerve fibers |
| HP:0007182 | 4 | Peripheral hypomyelination |

| Cluster | Cell function | Description | Phenotype\_coverage | Genes |
| --- | --- | --- | --- | --- |
| 4 | GO:0042552 | myelination | 75 | SBF2, MPZ, MTMR2, ARHGEF10, FIG4, EGR2, KCNJ10, PSAP, SOX10, FAM126A, NDRG1, GALC, PMP22, DHH |

---


---


---

# Cluster \*6

| HPO | Cluster | Description |
| --- | --- | --- |
| HP:0001561 | 6 | Polyhydramnios |
| HP:0001611 | 6 | Nasal speech |
| HP:0010628 | 6 | Facial palsy |
| HP:0001371 | 6 | Flexion contracture |
| HP:0003324 | 6 | Generalized muscle weakness |
| HP:0003798 | 6 | Nemaline bodies |
| HP:0001533 | 6 | Slender build |
| HP:0003810 | 6 | Late-onset distal muscle weakness |

| Cluster | Cell function | Description | Phenotype\_coverage | Genes |
| --- | --- | --- | --- | --- |
| 6 | GO:0048747 | muscle fiber development | 75 | LMOD3, NEB, ACTA1, KLHL40, SELENON, MYO18B, ZMPSTE24, RYR1 |
| 6 | GO:0051146 | striated muscle cell differentiation | 75 | LMOD3, NEB, MYPN, CFL2, ACTA1, KLHL40, SELENON, MYO18B, LMNA, ZMPSTE24, RYR1, TNNT1, TGFB1 |

---


---


---

# Cluster \*14

| HPO | Cluster | Description |
| --- | --- | --- |
| HP:0002486 | 14 | Myotonia |
| HP:0002411 | 14 | Myokymia |
| HP:0002292 | 14 | Frontal balding |
| HP:0003752 | 14 | Episodic flaccid weakness |

| Cluster | Cell function | Description | Phenotype\_coverage | Genes |
| --- | --- | --- | --- | --- |
| 14 | GO:0014819 | regulation of skeletal muscle contraction | 75 | DMPK, SCN4A |
| 14 | R-HSA-5576891 | Cardiac conduction | 75 | CACNA1S, DMPK, SCN4A |
| 14 | R-HSA-445095 | Interaction between L1 and Ankyrins | 75 | SCN4A, KCNQ2 |
| 14 | R-HSA-397014 | Muscle contraction | 75 | CACNA1S, DMPK, SCN4A |

---


---


---

# Cluster \*15

| HPO | Cluster | Description |
| --- | --- | --- |
| HP:0001270 | 15 | Motor delay |
| HP:0000508 | 15 | Ptosis |
| HP:0003701 | 15 | Proximal muscle weakness |
| HP:0003307 | 15 | Hyperlordosis |
| HP:0010628 | 15 | Facial palsy |
| HP:0001371 | 15 | Flexion contracture |
| HP:0003324 | 15 | Generalized muscle weakness |
| HP:0003803 | 15 | Type 1 muscle fiber predominance |
| HP:0003458 | 15 | EMG: myopathic abnormalities |
| HP:0003557 | 15 | Increased variability in muscle fiber diameter |
| HP:0002515 | 15 | Waddling gait |
| HP:0003691 | 15 | Scapular winging |
| HP:0002747 | 15 | Respiratory insufficiency due to muscle weakness |
| HP:0003547 | 15 | Shoulder girdle muscle weakness |
| HP:0003749 | 15 | Pelvic girdle muscle weakness |
| HP:0003391 | 15 | Gowers sign |
| HP:0003687 | 15 | Centrally nucleated skeletal muscle fibers |
| HP:0003805 | 15 | Rimmed vacuoles |
| HP:0002093 | 15 | Respiratory insufficiency |
| HP:0003306 | 15 | Spinal rigidity |
| HP:0003722 | 15 | Neck flexor weakness |
| HP:0003810 | 15 | Late-onset distal muscle weakness |
| HP:0008988 | 15 | Pelvic girdle muscle atrophy |
| HP:0003731 | 15 | Quadriceps muscle weakness |

| Cluster | Cell function | Description | Phenotype\_coverage | Genes |
| --- | --- | --- | --- | --- |
| 15 | GO:0048747 | muscle fiber development | 75 | SGCB, LMOD3, NEB, TCAP, ACTA1, COMP, OBSL1, SELENON, FLNC, ZMPSTE24, TTN, RYR1 |

---


---


---

# Cluster \*72

| HPO | Cluster | Description |
| --- | --- | --- |
| HP:0003674 | 72 | Onset |
| HP:0007141 | 72 | Sensorimotor neuropathy |
| HP:0003392 | 72 | First dorsal interossei muscle weakness |
| HP:0003426 | 72 | First dorsal interossei muscle atrophy |
| HP:0003427 | 72 | Thenar muscle weakness |
| HP:0003435 | 72 | Cold-induced hand cramps |
| HP:0003484 | 72 | Upper limb muscle weakness |
| HP:0009129 | 72 | Upper limb amyotrophy |

| Cluster | Cell function | Description | Phenotype\_coverage | Genes |
| --- | --- | --- | --- | --- |
| 72 | hsa00970 | Aminoacyl-tRNA biosynthesis | 75 | GARS1, YARS1 |
| 72 | GO:0006418 | tRNA aminoacylation for protein translation | 75 | YARS1, GARS1 |
| 72 | R-HSA-379724 | tRNA Aminoacylation | 75 | YARS1, GARS1 |
| 72 | R-HSA-379726 | Mitochondrial tRNA aminoacylation | 75 | GARS1 |
| 72 | R-HSA-72766 | Translation | 75 | YARS1, GARS1 |
| 72 | R-HSA-379716 | Cytosolic tRNA aminoacylation | 75 | YARS1, GARS1 |

---


---


---

# Cluster \*80

| HPO | Cluster | Description |
| --- | --- | --- |
| HP:0006597 | 80 | Diaphragmatic paralysis |
| HP:0007108 | 80 | Demyelinating peripheral neuropathy |
| HP:0032341 | 80 | Reduced forced vital capacity |
| HP:0003715 | 80 | Myofibrillar myopathy |

| Cluster | Cell function | Description | Phenotype\_coverage | Genes |
| --- | --- | --- | --- | --- |
| 80 | GO:0046716 | muscle cell cellular homeostasis | 75 | BAG3, GAA, CFL2 |

---


---


---

# Cluster \*125

| HPO | Cluster | Description |
| --- | --- | --- |
| HP:0001349 | 125 | Facial diplegia |
| HP:0003554 | 125 | Type 2 muscle fiber atrophy |
| HP:0002292 | 125 | Frontal balding |
| HP:0003722 | 125 | Neck flexor weakness |

| Cluster | Cell function | Description | Phenotype\_coverage | Genes |
| --- | --- | --- | --- | --- |
| 125 | R-HSA-397014 | Muscle contraction | 75 | DMPK, TPM3, TPM2, NEB, ACTA1 |

---


---


---

# Cluster \*131

| HPO | Cluster | Description |
| --- | --- | --- |
| HP:0006958 | 131 | Abnormal auditory evoked potentials |
| HP:0001963 | 131 | Abnormal speech discrimination |
| HP:0004463 | 131 | Absent brainstem auditory responses |
| HP:0008529 | 131 | Absence of acoustic reflex |

| Cluster | Cell function | Description | Phenotype\_coverage | Genes |
| --- | --- | --- | --- | --- |
| 131 | GO:0051258 | protein polymerization | 75 | DIAPH3, OPA1 |

---


---


---

# Cluster \*201

| HPO | Cluster | Description |
| --- | --- | --- |
| HP:0001425 | 201 | Heterogeneous |
| HP:0003324 | 201 | Generalized muscle weakness |
| HP:0003798 | 201 | Nemaline bodies |
| HP:0003803 | 201 | Type 1 muscle fiber predominance |
| HP:0003458 | 201 | EMG: myopathic abnormalities |
| HP:0001427 | 201 | Mitochondrial inheritance |
| HP:0003688 | 201 | Cytochrome C oxidase-negative muscle fibers |
| HP:0003810 | 201 | Late-onset distal muscle weakness |

| Cluster | Cell function | Description | Phenotype\_coverage | Genes |
| --- | --- | --- | --- | --- |
| 201 | GO:0048747 | muscle fiber development | 75 | LMOD3, NEB, ACTA1, KLHL40, SELENON, MYO18B, ZMPSTE24, TTN, RYR1 |
| 201 | GO:0014706 | striated muscle tissue development | 75 | MYH7, LMOD3, NEB, EGR2, MTM1, CHRNA1, CFL2, ACTA1, KLHL40, SELENON, MYO18B, LMNA, ZMPSTE24, TTN, RYR1 |
| 201 | GO:0030049 | muscle filament sliding | 75 | MYH7, NEB, TPM3, ACTA1, TPM2, MYH2, TTN, MYL1, TNNT1 |
| 201 | R-HSA-390522 | Striated Muscle Contraction | 75 | TPM3, TTN, MYL1, TPM2, NEB, TNNT1, ACTA1 |
| 201 | R-HSA-397014 | Muscle contraction | 75 | TPM3, KCNQ1, TTN, MYL1, TPM2, NEB, FXYD2, TNNT1, ACTA1, KCNE1, RYR1 |

---


---


---

# Cluster \*218

| HPO | Cluster | Description |
| --- | --- | --- |
| HP:0003546 | 218 | Exercise intolerance |
| HP:0001992 | 218 | Organic aciduria |
| HP:0012548 | 218 | Fatty replacement of skeletal muscle |
| HP:0008322 | 218 | Abnormal mitochondrial morphology |

| Cluster | Cell function | Description | Phenotype\_coverage | Genes |
| --- | --- | --- | --- | --- |
| 218 | GO:0009165 | nucleotide biosynthetic process | 75 | FLAD1, MPC1, ATP5F1D, LDHA, TAZ, PGAM2, PGM1, PFKM, PGK1, ENO3 |
| 218 | GO:0006732 | coenzyme metabolic process | 75 | FLAD1, MPC1, LDHA, PGAM2, PFKM, BTD, PGM1, COQ8A, MCCC2, SLC25A32, PGK1, HLCS, ENO3 |

---


---


---

# Cluster \*228

| HPO | Cluster | Description |
| --- | --- | --- |
| HP:0001171 | 228 | Split hand |
| HP:0001425 | 228 | Heterogeneous |
| HP:0001265 | 228 | Hyporeflexia |
| HP:0003477 | 228 | Peripheral axonal neuropathy |
| HP:0001761 | 228 | Pes cavus |
| HP:0003693 | 228 | Distal amyotrophy |
| HP:0001178 | 228 | Ulnar claw |
| HP:0001284 | 228 | Areflexia |
| HP:0001765 | 228 | Hammertoe |
| HP:0002460 | 228 | Distal muscle weakness |
| HP:0002936 | 228 | Distal sensory impairment |
| HP:0003376 | 228 | Steppage gait |
| HP:0003380 | 228 | Decreased number of peripheral myelinated nerve fibers |
| HP:0003382 | 228 | Hypertrophic nerve changes |
| HP:0003383 | 228 | Onion bulb formation |
| HP:0003431 | 228 | Decreased motor nerve conduction velocity |
| HP:0003449 | 228 | Cold-induced muscle cramps |
| HP:0003621 | 228 | Juvenile onset |
| HP:0004336 | 228 | Myelin outfoldings |
| HP:0009027 | 228 | Foot dorsiflexor weakness |
| HP:0003378 | 228 | Axonal degeneration/regeneration |
| HP:0003384 | 228 | Peripheral axonal atrophy |
| HP:0003481 | 228 | Segmental peripheral demyelination/remyelination |
| HP:0003484 | 228 | Upper limb muscle weakness |

| Cluster | Cell function | Description | Phenotype\_coverage | Genes |
| --- | --- | --- | --- | --- |
| 228 | GO:0042552 | myelination | 75 | SBF2, SH3TC2, MPZ, MTMR2, ARHGEF10, EGR2, PSAP, SOX10, FAM126A, NDRG1, PMP22, DHH |

---


---


---

# Cluster \*200

| HPO | Cluster | Description |
| --- | --- | --- |
| HP:0001425 | 200 | Heterogeneous |
| HP:0001291 | 200 | Abnormal cranial nerve morphology |
| HP:0040078 | 200 | Axonal degeneration |
| HP:0001761 | 200 | Pes cavus |
| HP:0003693 | 200 | Distal amyotrophy |
| HP:0001178 | 200 | Ulnar claw |
| HP:0001284 | 200 | Areflexia |
| HP:0002460 | 200 | Distal muscle weakness |
| HP:0002936 | 200 | Distal sensory impairment |
| HP:0003380 | 200 | Decreased number of peripheral myelinated nerve fibers |
| HP:0003382 | 200 | Hypertrophic nerve changes |
| HP:0003431 | 200 | Decreased motor nerve conduction velocity |
| HP:0003481 | 200 | Segmental peripheral demyelination/remyelination |
| HP:0001604 | 200 | Vocal cord paresis |
| HP:0003387 | 200 | Decreased number of large peripheral myelinated nerve fibers |
| HP:0003400 | 200 | Basal lamina onion bulb formation |
| HP:0003484 | 200 | Upper limb muscle weakness |
| HP:0007107 | 200 | Segmental peripheral demyelination |
| HP:0000764 | 200 | Peripheral axonal degeneration |

| Cluster | Cell function | Description | Phenotype\_coverage | Genes |
| --- | --- | --- | --- | --- |
| 200 | GO:0008366 | axon ensheathment | 73.68421 | SBF2, SH3TC2, MPZ, MTMR2, EGR2, FAM126A, NDRG1, PMP22, DHH, PRX |

---


---


---

# Cluster \*205

| HPO | Cluster | Description |
| --- | --- | --- |
| HP:0003198 | 205 | Myopathy |
| HP:0003701 | 205 | Proximal muscle weakness |
| HP:0010628 | 205 | Facial palsy |
| HP:0003326 | 205 | Myalgia |
| HP:0003458 | 205 | EMG: myopathic abnormalities |
| HP:0003737 | 205 | Mitochondrial myopathy |
| HP:0003200 | 205 | Ragged-red muscle fibers |
| HP:0003557 | 205 | Increased variability in muscle fiber diameter |
| HP:0001618 | 205 | Dysphonia |
| HP:0003390 | 205 | Sensory axonal neuropathy |
| HP:0000590 | 205 | Progressive external ophthalmoplegia |
| HP:0003323 | 205 | Progressive muscle weakness |
| HP:0003548 | 205 | Subsarcolemmal accumulations of abnormally shaped mitochondria |
| HP:0003688 | 205 | Cytochrome C oxidase-negative muscle fibers |
| HP:0003713 | 205 | Muscle fiber necrosis |
| HP:0006886 | 205 | Impaired distal vibration sensation |
| HP:0002403 | 205 | Positive Romberg sign |
| HP:0003434 | 205 | Sensory ataxic neuropathy |
| HP:0003687 | 205 | Centrally nucleated skeletal muscle fibers |

| Cluster | Cell function | Description | Phenotype\_coverage | Genes |
| --- | --- | --- | --- | --- |
| 205 | GO:0000002 | mitochondrial genome maintenance | 73.68421 | POLG2, MGME1, SLC25A4, POLG, MSTO1, RRM2B, TWNK, DNA2, TOP3A, TYMP |

---


---


---

# Cluster \*226

| HPO | Cluster | Description |
| --- | --- | --- |
| HP:0009771 | 226 | Osteolytic defects of the phalanges of the hand |
| HP:0000970 | 226 | Anhidrosis |
| HP:0002754 | 226 | Osteomyelitis |
| HP:0007460 | 226 | Autoamputation of digits |
| HP:0002661 | 226 | Painless fractures due to injury |
| HP:0007021 | 226 | Pain insensitivity |
| HP:0008000 | 226 | Decreased corneal reflex |
| HP:0000559 | 226 | Corneal scarring |
| HP:0012804 | 226 | Corneal ulceration |
| HP:0000522 | 226 | Alacrima |
| HP:0000224 | 226 | Decreased taste sensation |
| HP:0001069 | 226 | Episodic hyperhidrosis |
| HP:0006121 | 226 | Acral ulceration |
| HP:0002821 | 226 | Neuropathic arthropathy |
| HP:0012211 | 226 | Abnormal renal physiology |

| Cluster | Cell function | Description | Phenotype\_coverage | Genes |
| --- | --- | --- | --- | --- |
| 226 | GO:0019233 | sensory perception of pain | 73.33333 | SCN11A, SCN9A, NTRK1, ZFHX2, PRDM12, RETREG1 |

---


---


---

# Cluster \*48

| HPO | Cluster | Description |
| --- | --- | --- |
| HP:0001324 | 48 | Muscle weakness |
| HP:0000602 | 48 | Ophthalmoplegia |
| HP:0003198 | 48 | Myopathy |
| HP:0001639 | 48 | Hypertrophic cardiomyopathy |
| HP:0003737 | 48 | Mitochondrial myopathy |
| HP:0001427 | 48 | Mitochondrial inheritance |
| HP:0003200 | 48 | Ragged-red muscle fibers |
| HP:0003201 | 48 | Rhabdomyolysis |
| HP:0003546 | 48 | Exercise intolerance |
| HP:0002878 | 48 | Respiratory failure |
| HP:0002490 | 48 | Increased CSF lactate |

| Cluster | Cell function | Description | Phenotype\_coverage | Genes |
| --- | --- | --- | --- | --- |
| 48 | GO:0045333 | cellular respiration | 72.72727 | ATP5F1D, SUCLA2, ND4, COX8A, ND4L, COX2, COX15, PDHA1, COX10, MDH2, NDUFS1, ND5, ND2, FASTKD2, CYTB, NDUFS4, COX1, COX3, ISCU, ND6, NDUFV1, NDUFB8, SURF1, SDHA, TAZ, ND1, COX6B1 |
| 48 | GO:0009167 | purine ribonucleoside monophosphate metabolic process | 72.72727 | ATP5F1D, AMPD1, ND4, PGAM2, COX8A, ND4L, COX2, COX15, PGM1, PFKM, COX10, NDUFS1, LDHA, ND5, ND2, CYTB, ENO3, NDUFS4, COX1, COX3, ISCU, ND6, NDUFV1, ATP6, NDUFB8, SURF1, SDHA, TAZ, PGK1, ND1, COX6B1 |
| 48 | GO:0006119 | oxidative phosphorylation | 72.72727 | ATP5F1D, ND4, COX8A, ND4L, COX2, COX15, COX10, NDUFS1, ND5, ND2, CYTB, NDUFS4, COX1, COX3, ISCU, ND6, NDUFV1, ATP6, NDUFB8, SURF1, SDHA, TAZ, PGK1, ND1, COX6B1 |
| 48 | GO:0009205 | purine ribonucleoside triphosphate metabolic process | 72.72727 | ATP5F1D, ND4, PGAM2, COX8A, ND4L, COX2, COX15, PGM1, PFKM, COX10, NDUFS1, LDHA, ND5, ND2, CYTB, ENO3, NDUFS4, COX1, COX3, ISCU, ND6, NDUFV1, ATP6, NDUFB8, SURF1, SDHA, TAZ, PGK1, ND1, COX6B1 |
| 48 | GO:0022900 | electron transport chain | 72.72727 | FDX2, ND4, COX8A, ND4L, COX2, COX15, COX10, NDUFS1, ND5, ND2, CYTB, NDUFS4, COX1, COX3, ISCU, ND6, NDUFV1, NDUFB8, SURF1, SDHA, TAZ, ND1, COX6B1 |

---


---


---

# Cluster \*115

| HPO | Cluster | Description |
| --- | --- | --- |
| HP:0002650 | 115 | Scoliosis |
| HP:0001283 | 115 | Bulbar palsy |
| HP:0008872 | 115 | Feeding difficulties in infancy |
| HP:0010628 | 115 | Facial palsy |
| HP:0003691 | 115 | Scapular winging |
| HP:0000467 | 115 | Neck muscle weakness |
| HP:0002093 | 115 | Respiratory insufficiency |
| HP:0010307 | 115 | Stridor |
| HP:0003704 | 115 | Scapuloperoneal weakness |
| HP:0003697 | 115 | Scapuloperoneal amyotrophy |
| HP:0009113 | 115 | Diaphragmatic weakness |

| Cluster | Cell function | Description | Phenotype\_coverage | Genes |
| --- | --- | --- | --- | --- |
| 115 | GO:0030049 | muscle filament sliding | 72.72727 | MYH7, NEB, DES, MYH3, TPM3, ACTA1, MYH2, TTN, MYL1 |

---


---


---

# Cluster \*23

| HPO | Cluster | Description |
| --- | --- | --- |
| HP:0009830 | 23 | Peripheral neuropathy |
| HP:0001425 | 23 | Heterogeneous |
| HP:0001265 | 23 | Hyporeflexia |
| HP:0040078 | 23 | Axonal degeneration |
| HP:0001761 | 23 | Pes cavus |
| HP:0003677 | 23 | Slow progression |
| HP:0003693 | 23 | Distal amyotrophy |
| HP:0001284 | 23 | Areflexia |
| HP:0001765 | 23 | Hammertoe |
| HP:0002460 | 23 | Distal muscle weakness |
| HP:0002936 | 23 | Distal sensory impairment |
| HP:0003380 | 23 | Decreased number of peripheral myelinated nerve fibers |
| HP:0003383 | 23 | Onion bulb formation |
| HP:0003431 | 23 | Decreased motor nerve conduction velocity |
| HP:0000764 | 23 | Peripheral axonal degeneration |
| HP:0003387 | 23 | Decreased number of large peripheral myelinated nerve fibers |
| HP:0003400 | 23 | Basal lamina onion bulb formation |
| HP:0007107 | 23 | Segmental peripheral demyelination |

| Cluster | Cell function | Description | Phenotype\_coverage | Genes |
| --- | --- | --- | --- | --- |
| 23 | GO:0008366 | axon ensheathment | 72.22222 | SBF2, SH3TC2, MPZ, MTMR2, ARHGEF10, EGR2, PSAP, FAM126A, NDRG1, PMP22, DHH, PRX |

---


---


---

# Cluster \*67

| HPO | Cluster | Description |
| --- | --- | --- |
| HP:0009830 | 67 | Peripheral neuropathy |
| HP:0002751 | 67 | Kyphoscoliosis |
| HP:0001171 | 67 | Split hand |
| HP:0001265 | 67 | Hyporeflexia |
| HP:0001291 | 67 | Abnormal cranial nerve morphology |
| HP:0040078 | 67 | Axonal degeneration |
| HP:0003693 | 67 | Distal amyotrophy |
| HP:0001284 | 67 | Areflexia |
| HP:0002460 | 67 | Distal muscle weakness |
| HP:0002936 | 67 | Distal sensory impairment |
| HP:0003382 | 67 | Hypertrophic nerve changes |
| HP:0003383 | 67 | Onion bulb formation |
| HP:0003431 | 67 | Decreased motor nerve conduction velocity |
| HP:0004336 | 67 | Myelin outfoldings |
| HP:0007267 | 67 | Chronic axonal neuropathy |
| HP:0003400 | 67 | Basal lamina onion bulb formation |
| HP:0007182 | 67 | Peripheral hypomyelination |
| HP:0003450 | 67 | Axonal regeneration |

| Cluster | Cell function | Description | Phenotype\_coverage | Genes |
| --- | --- | --- | --- | --- |
| 67 | GO:0042552 | myelination | 72.22222 | SBF2, SH3TC2, MPZ, MTMR2, ARHGEF10, FIG4, EGR2, KCNJ10, PSAP, SOX10, FAM126A, NDRG1, PMP22 |

---


---


---

# Cluster \*53

| HPO | Cluster | Description |
| --- | --- | --- |
| HP:0000158 | 53 | Macroglossia |
| HP:0003701 | 53 | Proximal muscle weakness |
| HP:0010628 | 53 | Facial palsy |
| HP:0001644 | 53 | Dilated cardiomyopathy |
| HP:0003236 | 53 | Elevated serum creatine kinase |
| HP:0002111 | 53 | Restrictive deficit on pulmonary function testing |
| HP:0008981 | 53 | Calf muscle hypertrophy |
| HP:0003749 | 53 | Pelvic girdle muscle weakness |
| HP:0003306 | 53 | Spinal rigidity |
| HP:0001771 | 53 | Achilles tendon contracture |
| HP:0006785 | 53 | Limb-girdle muscular dystrophy |
| HP:0032341 | 53 | Reduced forced vital capacity |
| HP:0003733 | 53 | Thigh hypertrophy |
| HP:0030046 | 53 | Hypoglycosylation of alpha-dystroglycan |

| Cluster | Cell function | Description | Phenotype\_coverage | Genes |
| --- | --- | --- | --- | --- |
| 53 | GO:0035269 | protein O-linked mannosylation | 71.42857 | POMT1, LARGE1, CRPPA, FKRP, POMGNT2, POMT2, FKTN |

---


---


---

# Cluster \*184

| HPO | Cluster | Description |
| --- | --- | --- |
| HP:0003710 | 184 | Exercise-induced muscle cramps |
| HP:0003712 | 184 | Skeletal muscle hypertrophy |
| HP:0003552 | 184 | Muscle stiffness |
| HP:0003738 | 184 | Exercise-induced myalgia |
| HP:0008967 | 184 | Exercise-induced muscle stiffness |
| HP:0003457 | 184 | EMG abnormality |
| HP:0003719 | 184 | Muscle mounding |

| Cluster | Cell function | Description | Phenotype\_coverage | Genes |
| --- | --- | --- | --- | --- |
| 184 | GO:0001765 | membrane raft assembly | 71.42857 | CAV3 |
| 184 | GO:0061052 | negative regulation of cell growth involved in cardiac muscle cell development | 71.42857 | CAV3 |
| 184 | GO:0001778 | plasma membrane repair | 71.42857 | CAV3 |
| 184 | GO:0045759 | negative regulation of action potential | 71.42857 | CAV3 |
| 184 | GO:0045792 | negative regulation of cell size | 71.42857 | CAV3 |
| 184 | GO:0046716 | muscle cell cellular homeostasis | 71.42857 | CAV3, PFKM, LAMP2, TRIM32 |
| 184 | GO:0060347 | heart trabecula formation | 71.42857 | CAV3 |
| 184 | GO:0072584 | caveolin-mediated endocytosis | 71.42857 | CAV3 |
| 184 | GO:0051001 | negative regulation of nitric-oxide synthase activity | 71.42857 | CAV3 |
| 184 | GO:2000009 | negative regulation of protein localization to cell surface | 71.42857 | CAV3 |

---


---


---

# Cluster \*198

| HPO | Cluster | Description |
| --- | --- | --- |
| HP:0001638 | 198 | Cardiomyopathy |
| HP:0011675 | 198 | Arrhythmia |
| HP:0003236 | 198 | Elevated serum creatine kinase |
| HP:0003560 | 198 | Muscular dystrophy |
| HP:0002522 | 198 | Areflexia of lower limbs |
| HP:0003115 | 198 | Abnormal EKG |
| HP:0003707 | 198 | Calf muscle pseudohypertrophy |

| Cluster | Cell function | Description | Phenotype\_coverage | Genes |
| --- | --- | --- | --- | --- |
| 198 | hsa05410 | Hypertrophic cardiomyopathy (HCM) | 71.42857 | DAG1, SGCG, LAMA2, PRKAG2, DMD, SGCB, TTN, MYH7 |
| 198 | GO:0046716 | muscle cell cellular homeostasis | 71.42857 | TRIM32, CAPN3, LARGE1, DMD, CAV3, LAMP2 |
| 198 | GO:0007517 | muscle organ development | 71.42857 | SGCB, MYH7, MYLK2, FHL1, LARGE1, CAPN3, FOXC2, TAZ, LAMA2, CRYAB, DMD, TTN, FKTN, SGCG, CAV3 |

---


---


---

# Cluster \*47

| HPO | Cluster | Description |
| --- | --- | --- |
| HP:0001265 | 47 | Hyporeflexia |
| HP:0001761 | 47 | Pes cavus |
| HP:0003677 | 47 | Slow progression |
| HP:0001284 | 47 | Areflexia |
| HP:0002460 | 47 | Distal muscle weakness |
| HP:0002936 | 47 | Distal sensory impairment |
| HP:0003376 | 47 | Steppage gait |
| HP:0009027 | 47 | Foot dorsiflexor weakness |
| HP:0002359 | 47 | Frequent falls |
| HP:0007141 | 47 | Sensorimotor neuropathy |

| Cluster | Cell function | Description | Phenotype\_coverage | Genes |
| --- | --- | --- | --- | --- |
| 47 | GO:0008366 | axon ensheathment | 70 | SBF2, MPZ, EGR2, PSAP, PMP22, PRX |

---


---


---

# Cluster \*166

| HPO | Cluster | Description |
| --- | --- | --- |
| HP:0001270 | 166 | Motor delay |
| HP:0003307 | 166 | Hyperlordosis |
| HP:0003324 | 166 | Generalized muscle weakness |
| HP:0020152 | 166 | Distal joint laxity |
| HP:0003473 | 166 | Fatigable weakness |
| HP:0001612 | 166 | Weak cry |
| HP:0003554 | 166 | Type 2 muscle fiber atrophy |
| HP:0000597 | 166 | Ophthalmoparesis |
| HP:0003436 | 166 | Prolonged miniature endplate currents |
| HP:0003443 | 166 | Decreased size of nerve terminals |

| Cluster | Cell function | Description | Phenotype\_coverage | Genes |
| --- | --- | --- | --- | --- |
| 166 | GO:0007271 | synaptic transmission, cholinergic | 70 | CHRNE, SLC5A7, CHRNA1, COLQ |
| 166 | GO:0007528 | neuromuscular junction development | 70 | AFG3L2, CHRNA1, COLQ |

---


---


---

# Cluster \*2

| HPO | Cluster | Description |
| --- | --- | --- |
| HP:0008000 | 2 | Decreased corneal reflex |
| HP:0003387 | 2 | Decreased number of large peripheral myelinated nerve fibers |
| HP:0000224 | 2 | Decreased taste sensation |
| HP:0001069 | 2 | Episodic hyperhidrosis |
| HP:0002821 | 2 | Neuropathic arthropathy |
| HP:0012211 | 2 | Abnormal renal physiology |

---


---


---

# Cluster \*5

| HPO | Cluster | Description |
| --- | --- | --- |
| HP:0001302 | 5 | Pachygyria |
| HP:0001270 | 5 | Motor delay |
| HP:0003593 | 5 | Infantile onset |
| HP:0008872 | 5 | Feeding difficulties in infancy |

---


---


---

# Cluster \*7

| HPO | Cluster | Description |
| --- | --- | --- |
| HP:0001271 | 7 | Polyneuropathy |
| HP:0000970 | 7 | Anhidrosis |
| HP:0006958 | 7 | Abnormal auditory evoked potentials |
| HP:0000762 | 7 | Decreased nerve conduction velocity |

---


---


---

# Cluster \*8

| HPO | Cluster | Description |
| --- | --- | --- |
| HP:0003828 | 8 | Variable expressivity |
| HP:0003202 | 8 | Skeletal muscle atrophy |
| HP:0003677 | 8 | Slow progression |
| HP:0003325 | 8 | Limb-girdle muscle weakness |
| HP:0003690 | 8 | Limb muscle weakness |
| HP:0003701 | 8 | Proximal muscle weakness |
| HP:0001371 | 8 | Flexion contracture |
| HP:0003326 | 8 | Myalgia |
| HP:0003458 | 8 | EMG: myopathic abnormalities |
| HP:0003546 | 8 | Exercise intolerance |
| HP:0003691 | 8 | Scapular winging |
| HP:0002355 | 8 | Difficulty walking |
| HP:0002747 | 8 | Respiratory insufficiency due to muscle weakness |
| HP:0003391 | 8 | Gowers sign |
| HP:0008180 | 8 | Mildly elevated creatine kinase |
| HP:0003306 | 8 | Spinal rigidity |

---


---


---

# Cluster \*9

| HPO | Cluster | Description |
| --- | --- | --- |
| HP:0001324 | 9 | Muscle weakness |
| HP:0003198 | 9 | Myopathy |
| HP:0003701 | 9 | Proximal muscle weakness |
| HP:0010628 | 9 | Facial palsy |
| HP:0001644 | 9 | Dilated cardiomyopathy |
| HP:0003324 | 9 | Generalized muscle weakness |
| HP:0003803 | 9 | Type 1 muscle fiber predominance |
| HP:0003458 | 9 | EMG: myopathic abnormalities |
| HP:0003560 | 9 | Muscular dystrophy |
| HP:0003200 | 9 | Ragged-red muscle fibers |
| HP:0003201 | 9 | Rhabdomyolysis |
| HP:0003546 | 9 | Exercise intolerance |
| HP:0003557 | 9 | Increased variability in muscle fiber diameter |
| HP:0002515 | 9 | Waddling gait |
| HP:0008981 | 9 | Calf muscle hypertrophy |
| HP:0003687 | 9 | Centrally nucleated skeletal muscle fibers |
| HP:0008180 | 9 | Mildly elevated creatine kinase |
| HP:0003306 | 9 | Spinal rigidity |
| HP:0003741 | 9 | Congenital muscular dystrophy |

---


---


---

# Cluster \*10

| HPO | Cluster | Description |
| --- | --- | --- |
| HP:0001638 | 10 | Cardiomyopathy |
| HP:0003581 | 10 | Adult onset |
| HP:0001324 | 10 | Muscle weakness |
| HP:0003198 | 10 | Myopathy |
| HP:0003701 | 10 | Proximal muscle weakness |
| HP:0001371 | 10 | Flexion contracture |
| HP:0003326 | 10 | Myalgia |
| HP:0003458 | 10 | EMG: myopathic abnormalities |
| HP:0003560 | 10 | Muscular dystrophy |
| HP:0003546 | 10 | Exercise intolerance |
| HP:0000590 | 10 | Progressive external ophthalmoplegia |
| HP:0003548 | 10 | Subsarcolemmal accumulations of abnormally shaped mitochondria |
| HP:0003688 | 10 | Cytochrome C oxidase-negative muscle fibers |
| HP:0006886 | 10 | Impaired distal vibration sensation |

---


---


---

# Cluster \*11

| HPO | Cluster | Description |
| --- | --- | --- |
| HP:0003198 | 11 | Myopathy |
| HP:0003325 | 11 | Limb-girdle muscle weakness |
| HP:0003691 | 11 | Scapular winging |
| HP:0008981 | 11 | Calf muscle hypertrophy |
| HP:0003805 | 11 | Rimmed vacuoles |
| HP:0008180 | 11 | Mildly elevated creatine kinase |
| HP:0006785 | 11 | Limb-girdle muscular dystrophy |
| HP:0030046 | 11 | Hypoglycosylation of alpha-dystroglycan |

---


---


---

# Cluster \*12

| HPO | Cluster | Description |
| --- | --- | --- |
| HP:0001638 | 12 | Cardiomyopathy |
| HP:0003326 | 12 | Myalgia |
| HP:0003458 | 12 | EMG: myopathic abnormalities |
| HP:0003560 | 12 | Muscular dystrophy |
| HP:0003737 | 12 | Mitochondrial myopathy |
| HP:0012378 | 12 | Fatigue |
| HP:0003200 | 12 | Ragged-red muscle fibers |
| HP:0003201 | 12 | Rhabdomyolysis |
| HP:0003546 | 12 | Exercise intolerance |
| HP:0000590 | 12 | Progressive external ophthalmoplegia |
| HP:0003323 | 12 | Progressive muscle weakness |
| HP:0003551 | 12 | Difficulty climbing stairs |

---


---


---

# Cluster \*13

| HPO | Cluster | Description |
| --- | --- | --- |
| HP:0000007 | 13 | Autosomal recessive inheritance |
| HP:0000508 | 13 | Ptosis |
| HP:0002650 | 13 | Scoliosis |
| HP:0003202 | 13 | Skeletal muscle atrophy |
| HP:0003676 | 13 | Progressive |
| HP:0001283 | 13 | Bulbar palsy |
| HP:0002015 | 13 | Dysphagia |
| HP:0003701 | 13 | Proximal muscle weakness |
| HP:0010628 | 13 | Facial palsy |
| HP:0011968 | 13 | Feeding difficulties |
| HP:0001308 | 13 | Tongue fasciculations |
| HP:0000467 | 13 | Neck muscle weakness |
| HP:0002093 | 13 | Respiratory insufficiency |
| HP:0002058 | 13 | Myopathic facies |

---


---


---

# Cluster \*16

| HPO | Cluster | Description |
| --- | --- | --- |
| HP:0009830 | 16 | Peripheral neuropathy |
| HP:0001171 | 16 | Split hand |
| HP:0003581 | 16 | Adult onset |
| HP:0001265 | 16 | Hyporeflexia |
| HP:0003477 | 16 | Peripheral axonal neuropathy |
| HP:0002380 | 16 | Fasciculations |
| HP:0040078 | 16 | Axonal degeneration |
| HP:0001761 | 16 | Pes cavus |
| HP:0003677 | 16 | Slow progression |
| HP:0003693 | 16 | Distal amyotrophy |
| HP:0001284 | 16 | Areflexia |
| HP:0001765 | 16 | Hammertoe |
| HP:0002460 | 16 | Distal muscle weakness |
| HP:0002936 | 16 | Distal sensory impairment |
| HP:0003376 | 16 | Steppage gait |
| HP:0003380 | 16 | Decreased number of peripheral myelinated nerve fibers |
| HP:0003383 | 16 | Onion bulb formation |
| HP:0003431 | 16 | Decreased motor nerve conduction velocity |
| HP:0002355 | 16 | Difficulty walking |
| HP:0003387 | 16 | Decreased number of large peripheral myelinated nerve fibers |

---


---


---

# Cluster \*17

| HPO | Cluster | Description |
| --- | --- | --- |
| HP:0003581 | 17 | Adult onset |
| HP:0003325 | 17 | Limb-girdle muscle weakness |
| HP:0003458 | 17 | EMG: myopathic abnormalities |
| HP:0002747 | 17 | Respiratory insufficiency due to muscle weakness |
| HP:0003547 | 17 | Shoulder girdle muscle weakness |
| HP:0003749 | 17 | Pelvic girdle muscle weakness |
| HP:0003805 | 17 | Rimmed vacuoles |
| HP:0003810 | 17 | Late-onset distal muscle weakness |
| HP:0003736 | 17 | Autophagic vacuoles |
| HP:0003555 | 17 | Muscle fiber splitting |
| HP:0009025 | 17 | Increased connective tissue |
| HP:0003715 | 17 | Myofibrillar myopathy |

---


---


---

# Cluster \*18

| HPO | Cluster | Description |
| --- | --- | --- |
| HP:0009830 | 18 | Peripheral neuropathy |
| HP:0003828 | 18 | Variable expressivity |
| HP:0003202 | 18 | Skeletal muscle atrophy |
| HP:0003676 | 18 | Progressive |
| HP:0003581 | 18 | Adult onset |
| HP:0003477 | 18 | Peripheral axonal neuropathy |
| HP:0001761 | 18 | Pes cavus |
| HP:0003677 | 18 | Slow progression |
| HP:0003693 | 18 | Distal amyotrophy |
| HP:0002936 | 18 | Distal sensory impairment |
| HP:0003390 | 18 | Sensory axonal neuropathy |
| HP:0007340 | 18 | Lower limb muscle weakness |
| HP:0002355 | 18 | Difficulty walking |
| HP:0003484 | 18 | Upper limb muscle weakness |

---


---


---

# Cluster \*19

| HPO | Cluster | Description |
| --- | --- | --- |
| HP:0003202 | 19 | Skeletal muscle atrophy |
| HP:0001324 | 19 | Muscle weakness |
| HP:0003394 | 19 | Muscle spasm |
| HP:0007354 | 19 | Amyotrophic lateral sclerosis |
| HP:0003470 | 19 | Paralysis |
| HP:0002355 | 19 | Difficulty walking |

---


---


---

# Cluster \*20

| HPO | Cluster | Description |
| --- | --- | --- |
| HP:0001762 | 20 | Talipes equinovarus |
| HP:0001270 | 20 | Motor delay |
| HP:0000508 | 20 | Ptosis |
| HP:0000218 | 20 | High palate |
| HP:0002650 | 20 | Scoliosis |
| HP:0001611 | 20 | Nasal speech |
| HP:0001319 | 20 | Neonatal hypotonia |
| HP:0003557 | 20 | Increased variability in muscle fiber diameter |
| HP:0003388 | 20 | Easy fatigability |
| HP:0009046 | 20 | Difficulty running |
| HP:0001558 | 20 | Decreased fetal movement |
| HP:0007126 | 20 | Proximal amyotrophy |
| HP:0003306 | 20 | Spinal rigidity |

---


---


---

# Cluster \*21

| HPO | Cluster | Description |
| --- | --- | --- |
| HP:0001425 | 21 | Heterogeneous |
| HP:0001265 | 21 | Hyporeflexia |
| HP:0003693 | 21 | Distal amyotrophy |
| HP:0002460 | 21 | Distal muscle weakness |
| HP:0002936 | 21 | Distal sensory impairment |
| HP:0003431 | 21 | Decreased motor nerve conduction velocity |
| HP:0003387 | 21 | Decreased number of large peripheral myelinated nerve fibers |
| HP:0003484 | 21 | Upper limb muscle weakness |
| HP:0003450 | 21 | Axonal regeneration |

---


---


---

# Cluster \*22

| HPO | Cluster | Description |
| --- | --- | --- |
| HP:0001270 | 22 | Motor delay |
| HP:0001283 | 22 | Bulbar palsy |
| HP:0003701 | 22 | Proximal muscle weakness |
| HP:0003307 | 22 | Hyperlordosis |
| HP:0010628 | 22 | Facial palsy |
| HP:0001319 | 22 | Neonatal hypotonia |
| HP:0001371 | 22 | Flexion contracture |
| HP:0003324 | 22 | Generalized muscle weakness |
| HP:0003798 | 22 | Nemaline bodies |
| HP:0003803 | 22 | Type 1 muscle fiber predominance |
| HP:0003458 | 22 | EMG: myopathic abnormalities |
| HP:0003557 | 22 | Increased variability in muscle fiber diameter |
| HP:0002515 | 22 | Waddling gait |
| HP:0003749 | 22 | Pelvic girdle muscle weakness |
| HP:0003805 | 22 | Rimmed vacuoles |
| HP:0002058 | 22 | Myopathic facies |
| HP:0003722 | 22 | Neck flexor weakness |
| HP:0003810 | 22 | Late-onset distal muscle weakness |
| HP:0003551 | 22 | Difficulty climbing stairs |
| HP:0000597 | 22 | Ophthalmoparesis |
| HP:0003473 | 22 | Fatigable weakness |

---


---


---

# Cluster \*24

| HPO | Cluster | Description |
| --- | --- | --- |
| HP:0002650 | 24 | Scoliosis |
| HP:0003198 | 24 | Myopathy |
| HP:0003236 | 24 | Elevated serum creatine kinase |
| HP:0003557 | 24 | Increased variability in muscle fiber diameter |
| HP:0003741 | 24 | Congenital muscular dystrophy |
| HP:0012548 | 24 | Fatty replacement of skeletal muscle |

---


---


---

# Cluster \*25

| HPO | Cluster | Description |
| --- | --- | --- |
| HP:0001270 | 25 | Motor delay |
| HP:0001425 | 25 | Heterogeneous |
| HP:0001265 | 25 | Hyporeflexia |
| HP:0001761 | 25 | Pes cavus |
| HP:0003383 | 25 | Onion bulb formation |
| HP:0009027 | 25 | Foot dorsiflexor weakness |
| HP:0008180 | 25 | Mildly elevated creatine kinase |
| HP:0008944 | 25 | Distal lower limb amyotrophy |

---


---


---

# Cluster \*27

| HPO | Cluster | Description |
| --- | --- | --- |
| HP:0001638 | 27 | Cardiomyopathy |
| HP:0000518 | 27 | Cataract |
| HP:0011675 | 27 | Arrhythmia |
| HP:0003200 | 27 | Ragged-red muscle fibers |
| HP:0003548 | 27 | Subsarcolemmal accumulations of abnormally shaped mitochondria |
| HP:0003688 | 27 | Cytochrome C oxidase-negative muscle fibers |

---


---


---

# Cluster \*28

| HPO | Cluster | Description |
| --- | --- | --- |
| HP:0001270 | 28 | Motor delay |
| HP:0000508 | 28 | Ptosis |
| HP:0003202 | 28 | Skeletal muscle atrophy |
| HP:0001324 | 28 | Muscle weakness |
| HP:0001561 | 28 | Polyhydramnios |
| HP:0001371 | 28 | Flexion contracture |
| HP:0002047 | 28 | Malignant hyperthermia |

---


---


---

# Cluster \*29

| HPO | Cluster | Description |
| --- | --- | --- |
| HP:0001270 | 29 | Motor delay |
| HP:0000218 | 29 | High palate |
| HP:0003677 | 29 | Slow progression |
| HP:0003701 | 29 | Proximal muscle weakness |
| HP:0001611 | 29 | Nasal speech |
| HP:0010628 | 29 | Facial palsy |
| HP:0003324 | 29 | Generalized muscle weakness |
| HP:0003798 | 29 | Nemaline bodies |
| HP:0003803 | 29 | Type 1 muscle fiber predominance |
| HP:0003557 | 29 | Increased variability in muscle fiber diameter |
| HP:0002515 | 29 | Waddling gait |
| HP:0003388 | 29 | Easy fatigability |
| HP:0003691 | 29 | Scapular winging |
| HP:0002359 | 29 | Frequent falls |
| HP:0002355 | 29 | Difficulty walking |
| HP:0008981 | 29 | Calf muscle hypertrophy |
| HP:0003391 | 29 | Gowers sign |
| HP:0000467 | 29 | Neck muscle weakness |
| HP:0003805 | 29 | Rimmed vacuoles |
| HP:0003306 | 29 | Spinal rigidity |
| HP:0003700 | 29 | Generalized amyotrophy |
| HP:0020152 | 29 | Distal joint laxity |
| HP:0003327 | 29 | Axial muscle weakness |
| HP:0003787 | 29 | Type 1 and type 2 muscle fiber minicore regions |
| HP:0002792 | 29 | Reduced vital capacity |

---


---


---

# Cluster \*30

| HPO | Cluster | Description |
| --- | --- | --- |
| HP:0000508 | 30 | Ptosis |
| HP:0001638 | 30 | Cardiomyopathy |
| HP:0001324 | 30 | Muscle weakness |
| HP:0011675 | 30 | Arrhythmia |
| HP:0003701 | 30 | Proximal muscle weakness |
| HP:0001644 | 30 | Dilated cardiomyopathy |
| HP:0003560 | 30 | Muscular dystrophy |
| HP:0003128 | 30 | Lactic acidosis |
| HP:0003546 | 30 | Exercise intolerance |
| HP:0000590 | 30 | Progressive external ophthalmoplegia |
| HP:0002093 | 30 | Respiratory insufficiency |

---


---


---

# Cluster \*31

| HPO | Cluster | Description |
| --- | --- | --- |
| HP:0001252 | 31 | Muscular hypotonia |
| HP:0003219 | 31 | Ethylmalonic aciduria |
| HP:0012847 | 31 | Epilepsia partialis continua |
| HP:0001992 | 31 | Organic aciduria |

---


---


---

# Cluster \*32

| HPO | Cluster | Description |
| --- | --- | --- |
| HP:0003394 | 32 | Muscle spasm |
| HP:0003677 | 32 | Slow progression |
| HP:0003701 | 32 | Proximal muscle weakness |
| HP:0002378 | 32 | Hand tremor |
| HP:0003457 | 32 | EMG abnormality |

---


---


---

# Cluster \*33

| HPO | Cluster | Description |
| --- | --- | --- |
| HP:0001762 | 33 | Talipes equinovarus |
| HP:0001270 | 33 | Motor delay |
| HP:0000508 | 33 | Ptosis |
| HP:0000218 | 33 | High palate |
| HP:0002650 | 33 | Scoliosis |
| HP:0001188 | 33 | Hand clenching |
| HP:0002804 | 33 | Arthrogryposis multiplex congenita |
| HP:0001561 | 33 | Polyhydramnios |
| HP:0003577 | 33 | Congenital onset |
| HP:0011968 | 33 | Feeding difficulties |
| HP:0001558 | 33 | Decreased fetal movement |
| HP:0002747 | 33 | Respiratory insufficiency due to muscle weakness |
| HP:0002058 | 33 | Myopathic facies |
| HP:0003810 | 33 | Late-onset distal muscle weakness |

---


---


---

# Cluster \*34

| HPO | Cluster | Description |
| --- | --- | --- |
| HP:0003202 | 34 | Skeletal muscle atrophy |
| HP:0001324 | 34 | Muscle weakness |
| HP:0002380 | 34 | Fasciculations |
| HP:0002486 | 34 | Myotonia |
| HP:0003326 | 34 | Myalgia |
| HP:0003712 | 34 | Skeletal muscle hypertrophy |
| HP:0003552 | 34 | Muscle stiffness |

---


---


---

# Cluster \*35

| HPO | Cluster | Description |
| --- | --- | --- |
| HP:0003581 | 35 | Adult onset |
| HP:0003394 | 35 | Muscle spasm |
| HP:0001761 | 35 | Pes cavus |
| HP:0003677 | 35 | Slow progression |
| HP:0003701 | 35 | Proximal muscle weakness |
| HP:0003376 | 35 | Steppage gait |
| HP:0009027 | 35 | Foot dorsiflexor weakness |
| HP:0003557 | 35 | Increased variability in muscle fiber diameter |
| HP:0002355 | 35 | Difficulty walking |
| HP:0002522 | 35 | Areflexia of lower limbs |
| HP:0009046 | 35 | Difficulty running |
| HP:0007126 | 35 | Proximal amyotrophy |

---


---


---

# Cluster \*36

| HPO | Cluster | Description |
| --- | --- | --- |
| HP:0001283 | 36 | Bulbar palsy |
| HP:0002804 | 36 | Arthrogryposis multiplex congenita |
| HP:0001561 | 36 | Polyhydramnios |
| HP:0003307 | 36 | Hyperlordosis |
| HP:0010628 | 36 | Facial palsy |
| HP:0003324 | 36 | Generalized muscle weakness |
| HP:0003803 | 36 | Type 1 muscle fiber predominance |
| HP:0001612 | 36 | Weak cry |
| HP:0002747 | 36 | Respiratory insufficiency due to muscle weakness |
| HP:0003391 | 36 | Gowers sign |
| HP:0003554 | 36 | Type 2 muscle fiber atrophy |
| HP:0002058 | 36 | Myopathic facies |
| HP:0003722 | 36 | Neck flexor weakness |
| HP:0003810 | 36 | Late-onset distal muscle weakness |
| HP:0002033 | 36 | Poor suck |
| HP:0000597 | 36 | Ophthalmoparesis |
| HP:0003402 | 36 | Decreased miniature endplate potentials |
| HP:0003403 | 36 | EMG: decremental response of compound muscle action potential to repetitive nerve stimulation |
| HP:0003473 | 36 | Fatigable weakness |

---


---


---

# Cluster \*37

| HPO | Cluster | Description |
| --- | --- | --- |
| HP:0001302 | 37 | Pachygyria |
| HP:0001561 | 37 | Polyhydramnios |
| HP:0003198 | 37 | Myopathy |
| HP:0003577 | 37 | Congenital onset |
| HP:0001321 | 37 | Cerebellar hypoplasia |
| HP:0001558 | 37 | Decreased fetal movement |
| HP:0002093 | 37 | Respiratory insufficiency |
| HP:0002058 | 37 | Myopathic facies |
| HP:0002365 | 37 | Hypoplasia of the brainstem |
| HP:0002803 | 37 | Congenital contracture |
| HP:0012110 | 37 | Hypoplasia of the pons |
| HP:0006829 | 37 | Severe muscular hypotonia |
| HP:0007033 | 37 | Cerebellar dysplasia |
| HP:0007260 | 37 | Type II lissencephaly |

---


---


---

# Cluster \*38

| HPO | Cluster | Description |
| --- | --- | --- |
| HP:0001302 | 38 | Pachygyria |
| HP:0001270 | 38 | Motor delay |
| HP:0000218 | 38 | High palate |
| HP:0000518 | 38 | Cataract |
| HP:0001283 | 38 | Bulbar palsy |
| HP:0002804 | 38 | Arthrogryposis multiplex congenita |
| HP:0001561 | 38 | Polyhydramnios |
| HP:0003307 | 38 | Hyperlordosis |
| HP:0010628 | 38 | Facial palsy |
| HP:0003577 | 38 | Congenital onset |
| HP:0001319 | 38 | Neonatal hypotonia |
| HP:0001371 | 38 | Flexion contracture |
| HP:0003324 | 38 | Generalized muscle weakness |
| HP:0003798 | 38 | Nemaline bodies |
| HP:0003803 | 38 | Type 1 muscle fiber predominance |
| HP:0001612 | 38 | Weak cry |
| HP:0003458 | 38 | EMG: myopathic abnormalities |
| HP:0003557 | 38 | Increased variability in muscle fiber diameter |
| HP:0002515 | 38 | Waddling gait |
| HP:0003388 | 38 | Easy fatigability |
| HP:0003691 | 38 | Scapular winging |
| HP:0002747 | 38 | Respiratory insufficiency due to muscle weakness |
| HP:0003391 | 38 | Gowers sign |
| HP:0000467 | 38 | Neck muscle weakness |
| HP:0002058 | 38 | Myopathic facies |
| HP:0003306 | 38 | Spinal rigidity |
| HP:0002033 | 38 | Poor suck |
| HP:0006829 | 38 | Severe muscular hypotonia |
| HP:0003473 | 38 | Fatigable weakness |

---


---


---

# Cluster \*39

| HPO | Cluster | Description |
| --- | --- | --- |
| HP:0001265 | 39 | Hyporeflexia |
| HP:0001284 | 39 | Areflexia |
| HP:0002878 | 39 | Respiratory failure |
| HP:0002490 | 39 | Increased CSF lactate |
| HP:0002421 | 39 | Poor head control |

---


---


---

# Cluster \*40

| HPO | Cluster | Description |
| --- | --- | --- |
| HP:0001283 | 40 | Bulbar palsy |
| HP:0002015 | 40 | Dysphagia |
| HP:0000544 | 40 | External ophthalmoplegia |
| HP:0003557 | 40 | Increased variability in muscle fiber diameter |
| HP:0002747 | 40 | Respiratory insufficiency due to muscle weakness |
| HP:0003547 | 40 | Shoulder girdle muscle weakness |
| HP:0003805 | 40 | Rimmed vacuoles |
| HP:0003736 | 40 | Autophagic vacuoles |
| HP:0008756 | 40 | Bowing of the vocal cords |

---


---


---

# Cluster \*41

| HPO | Cluster | Description |
| --- | --- | --- |
| HP:0000007 | 41 | Autosomal recessive inheritance |
| HP:0001762 | 41 | Talipes equinovarus |
| HP:0001270 | 41 | Motor delay |
| HP:0000508 | 41 | Ptosis |
| HP:0000218 | 41 | High palate |
| HP:0002650 | 41 | Scoliosis |
| HP:0001283 | 41 | Bulbar palsy |
| HP:0001324 | 41 | Muscle weakness |
| HP:0002804 | 41 | Arthrogryposis multiplex congenita |
| HP:0001561 | 41 | Polyhydramnios |
| HP:0003198 | 41 | Myopathy |
| HP:0003690 | 41 | Limb muscle weakness |
| HP:0003701 | 41 | Proximal muscle weakness |
| HP:0003307 | 41 | Hyperlordosis |
| HP:0010628 | 41 | Facial palsy |
| HP:0001319 | 41 | Neonatal hypotonia |
| HP:0001371 | 41 | Flexion contracture |
| HP:0003324 | 41 | Generalized muscle weakness |
| HP:0003798 | 41 | Nemaline bodies |
| HP:0003803 | 41 | Type 1 muscle fiber predominance |
| HP:0001612 | 41 | Weak cry |
| HP:0003557 | 41 | Increased variability in muscle fiber diameter |
| HP:0011968 | 41 | Feeding difficulties |
| HP:0001558 | 41 | Decreased fetal movement |
| HP:0002747 | 41 | Respiratory insufficiency due to muscle weakness |
| HP:0003391 | 41 | Gowers sign |
| HP:0003687 | 41 | Centrally nucleated skeletal muscle fibers |
| HP:0000467 | 41 | Neck muscle weakness |
| HP:0008180 | 41 | Mildly elevated creatine kinase |
| HP:0002093 | 41 | Respiratory insufficiency |
| HP:0002058 | 41 | Myopathic facies |
| HP:0003306 | 41 | Spinal rigidity |
| HP:0003722 | 41 | Neck flexor weakness |
| HP:0003810 | 41 | Late-onset distal muscle weakness |
| HP:0002033 | 41 | Poor suck |
| HP:0003700 | 41 | Generalized amyotrophy |
| HP:0003473 | 41 | Fatigable weakness |

---


---


---

# Cluster \*42

| HPO | Cluster | Description |
| --- | --- | --- |
| HP:0009830 | 42 | Peripheral neuropathy |
| HP:0003676 | 42 | Progressive |
| HP:0001265 | 42 | Hyporeflexia |
| HP:0002380 | 42 | Fasciculations |
| HP:0040078 | 42 | Axonal degeneration |
| HP:0001761 | 42 | Pes cavus |
| HP:0002015 | 42 | Dysphagia |
| HP:0003693 | 42 | Distal amyotrophy |
| HP:0003690 | 42 | Limb muscle weakness |
| HP:0003701 | 42 | Proximal muscle weakness |
| HP:0001284 | 42 | Areflexia |
| HP:0002460 | 42 | Distal muscle weakness |
| HP:0002936 | 42 | Distal sensory impairment |
| HP:0003380 | 42 | Decreased number of peripheral myelinated nerve fibers |
| HP:0003383 | 42 | Onion bulb formation |
| HP:0003431 | 42 | Decreased motor nerve conduction velocity |
| HP:0003378 | 42 | Axonal degeneration/regeneration |
| HP:0000763 | 42 | Sensory neuropathy |

---


---


---

# Cluster \*43

| HPO | Cluster | Description |
| --- | --- | --- |
| HP:0003828 | 43 | Variable expressivity |
| HP:0003690 | 43 | Limb muscle weakness |
| HP:0003701 | 43 | Proximal muscle weakness |
| HP:0010628 | 43 | Facial palsy |
| HP:0003324 | 43 | Generalized muscle weakness |
| HP:0003326 | 43 | Myalgia |
| HP:0003458 | 43 | EMG: myopathic abnormalities |
| HP:0003546 | 43 | Exercise intolerance |
| HP:0003388 | 43 | Easy fatigability |
| HP:0000590 | 43 | Progressive external ophthalmoplegia |
| HP:0003688 | 43 | Cytochrome C oxidase-negative muscle fibers |
| HP:0002355 | 43 | Difficulty walking |
| HP:0002111 | 43 | Restrictive deficit on pulmonary function testing |
| HP:0003547 | 43 | Shoulder girdle muscle weakness |
| HP:0008981 | 43 | Calf muscle hypertrophy |
| HP:0003749 | 43 | Pelvic girdle muscle weakness |
| HP:0003391 | 43 | Gowers sign |
| HP:0003687 | 43 | Centrally nucleated skeletal muscle fibers |
| HP:0001771 | 43 | Achilles tendon contracture |
| HP:0040083 | 43 | Toe walking |
| HP:0003733 | 43 | Thigh hypertrophy |

---


---


---

# Cluster \*44

| HPO | Cluster | Description |
| --- | --- | --- |
| HP:0001270 | 44 | Motor delay |
| HP:0003677 | 44 | Slow progression |
| HP:0003701 | 44 | Proximal muscle weakness |
| HP:0003307 | 44 | Hyperlordosis |
| HP:0003560 | 44 | Muscular dystrophy |
| HP:0003712 | 44 | Skeletal muscle hypertrophy |
| HP:0003552 | 44 | Muscle stiffness |
| HP:0003391 | 44 | Gowers sign |

---


---


---

# Cluster \*45

| HPO | Cluster | Description |
| --- | --- | --- |
| HP:0003828 | 45 | Variable expressivity |
| HP:0003324 | 45 | Generalized muscle weakness |
| HP:0008981 | 45 | Calf muscle hypertrophy |
| HP:0003306 | 45 | Spinal rigidity |
| HP:0002350 | 45 | Cerebellar cyst |
| HP:0001321 | 45 | Cerebellar hypoplasia |
| HP:0003733 | 45 | Thigh hypertrophy |

---


---


---

# Cluster \*46

| HPO | Cluster | Description |
| --- | --- | --- |
| HP:0001270 | 46 | Motor delay |
| HP:0000218 | 46 | High palate |
| HP:0002650 | 46 | Scoliosis |
| HP:0003676 | 46 | Progressive |
| HP:0002015 | 46 | Dysphagia |
| HP:0003701 | 46 | Proximal muscle weakness |
| HP:0001611 | 46 | Nasal speech |
| HP:0010628 | 46 | Facial palsy |
| HP:0001319 | 46 | Neonatal hypotonia |
| HP:0001371 | 46 | Flexion contracture |
| HP:0003458 | 46 | EMG: myopathic abnormalities |
| HP:0003200 | 46 | Ragged-red muscle fibers |
| HP:0003546 | 46 | Exercise intolerance |
| HP:0003557 | 46 | Increased variability in muscle fiber diameter |
| HP:0001618 | 46 | Dysphonia |
| HP:0003388 | 46 | Easy fatigability |
| HP:0000590 | 46 | Progressive external ophthalmoplegia |
| HP:0003713 | 46 | Muscle fiber necrosis |
| HP:0002747 | 46 | Respiratory insufficiency due to muscle weakness |
| HP:0003391 | 46 | Gowers sign |
| HP:0000467 | 46 | Neck muscle weakness |
| HP:0008180 | 46 | Mildly elevated creatine kinase |
| HP:0002058 | 46 | Myopathic facies |
| HP:0003306 | 46 | Spinal rigidity |
| HP:0003700 | 46 | Generalized amyotrophy |
| HP:0003741 | 46 | Congenital muscular dystrophy |
| HP:0020152 | 46 | Distal joint laxity |
| HP:0003327 | 46 | Axial muscle weakness |
| HP:0003787 | 46 | Type 1 and type 2 muscle fiber minicore regions |

---


---


---

# Cluster \*49

| HPO | Cluster | Description |
| --- | --- | --- |
| HP:0002398 | 49 | Degeneration of anterior horn cells |
| HP:0007269 | 49 | Spinal muscular atrophy |
| HP:0001308 | 49 | Tongue fasciculations |
| HP:0008955 | 49 | Progressive distal muscular atrophy |
| HP:0010307 | 49 | Stridor |

---


---


---

# Cluster \*50

| HPO | Cluster | Description |
| --- | --- | --- |
| HP:0001270 | 50 | Motor delay |
| HP:0000158 | 50 | Macroglossia |
| HP:0003701 | 50 | Proximal muscle weakness |
| HP:0003307 | 50 | Hyperlordosis |
| HP:0010628 | 50 | Facial palsy |
| HP:0001371 | 50 | Flexion contracture |
| HP:0003324 | 50 | Generalized muscle weakness |
| HP:0003798 | 50 | Nemaline bodies |
| HP:0003803 | 50 | Type 1 muscle fiber predominance |
| HP:0003326 | 50 | Myalgia |
| HP:0003458 | 50 | EMG: myopathic abnormalities |
| HP:0002111 | 50 | Restrictive deficit on pulmonary function testing |
| HP:0008981 | 50 | Calf muscle hypertrophy |
| HP:0003306 | 50 | Spinal rigidity |
| HP:0003722 | 50 | Neck flexor weakness |
| HP:0003810 | 50 | Late-onset distal muscle weakness |
| HP:0003551 | 50 | Difficulty climbing stairs |
| HP:0040083 | 50 | Toe walking |
| HP:0003733 | 50 | Thigh hypertrophy |

---


---


---

# Cluster \*51

| HPO | Cluster | Description |
| --- | --- | --- |
| HP:0001270 | 51 | Motor delay |
| HP:0003828 | 51 | Variable expressivity |
| HP:0001265 | 51 | Hyporeflexia |
| HP:0001761 | 51 | Pes cavus |
| HP:0003677 | 51 | Slow progression |
| HP:0003701 | 51 | Proximal muscle weakness |
| HP:0010628 | 51 | Facial palsy |
| HP:0001319 | 51 | Neonatal hypotonia |
| HP:0001371 | 51 | Flexion contracture |
| HP:0003798 | 51 | Nemaline bodies |
| HP:0001284 | 51 | Areflexia |
| HP:0003458 | 51 | EMG: myopathic abnormalities |
| HP:0003557 | 51 | Increased variability in muscle fiber diameter |
| HP:0003691 | 51 | Scapular winging |
| HP:0002359 | 51 | Frequent falls |
| HP:0002355 | 51 | Difficulty walking |
| HP:0009046 | 51 | Difficulty running |
| HP:0008981 | 51 | Calf muscle hypertrophy |
| HP:0003391 | 51 | Gowers sign |
| HP:0000467 | 51 | Neck muscle weakness |
| HP:0003805 | 51 | Rimmed vacuoles |
| HP:0002093 | 51 | Respiratory insufficiency |
| HP:0003306 | 51 | Spinal rigidity |
| HP:0003551 | 51 | Difficulty climbing stairs |
| HP:0009025 | 51 | Increased connective tissue |

---


---


---

# Cluster \*52

| HPO | Cluster | Description |
| --- | --- | --- |
| HP:0001425 | 52 | Heterogeneous |
| HP:0001761 | 52 | Pes cavus |
| HP:0003677 | 52 | Slow progression |
| HP:0003693 | 52 | Distal amyotrophy |
| HP:0003690 | 52 | Limb muscle weakness |
| HP:0003701 | 52 | Proximal muscle weakness |
| HP:0003803 | 52 | Type 1 muscle fiber predominance |
| HP:0002460 | 52 | Distal muscle weakness |
| HP:0011463 | 52 | Childhood onset |
| HP:0003236 | 52 | Elevated serum creatine kinase |
| HP:0003560 | 52 | Muscular dystrophy |
| HP:0002515 | 52 | Waddling gait |
| HP:0003445 | 52 | EMG: neuropathic changes |
| HP:0000467 | 52 | Neck muscle weakness |

---


---


---

# Cluster \*54

| HPO | Cluster | Description |
| --- | --- | --- |
| HP:0001265 | 54 | Hyporeflexia |
| HP:0001761 | 54 | Pes cavus |
| HP:0003677 | 54 | Slow progression |
| HP:0003693 | 54 | Distal amyotrophy |
| HP:0003701 | 54 | Proximal muscle weakness |
| HP:0003307 | 54 | Hyperlordosis |
| HP:0010628 | 54 | Facial palsy |
| HP:0003458 | 54 | EMG: myopathic abnormalities |
| HP:0002515 | 54 | Waddling gait |
| HP:0003691 | 54 | Scapular winging |
| HP:0002359 | 54 | Frequent falls |
| HP:0002355 | 54 | Difficulty walking |
| HP:0003547 | 54 | Shoulder girdle muscle weakness |
| HP:0008981 | 54 | Calf muscle hypertrophy |
| HP:0003306 | 54 | Spinal rigidity |
| HP:0003551 | 54 | Difficulty climbing stairs |
| HP:0001771 | 54 | Achilles tendon contracture |

---


---


---

# Cluster \*56

| HPO | Cluster | Description |
| --- | --- | --- |
| HP:0002380 | 56 | Fasciculations |
| HP:0002398 | 56 | Degeneration of anterior horn cells |
| HP:0001284 | 56 | Areflexia |
| HP:0003394 | 56 | Muscle spasm |
| HP:0007354 | 56 | Amyotrophic lateral sclerosis |
| HP:0040078 | 56 | Axonal degeneration |
| HP:0003677 | 56 | Slow progression |
| HP:0003701 | 56 | Proximal muscle weakness |
| HP:0002355 | 56 | Difficulty walking |
| HP:0001558 | 56 | Decreased fetal movement |
| HP:0002747 | 56 | Respiratory insufficiency due to muscle weakness |
| HP:0000764 | 56 | Peripheral axonal degeneration |
| HP:0001308 | 56 | Tongue fasciculations |

---


---


---

# Cluster \*57

| HPO | Cluster | Description |
| --- | --- | --- |
| HP:0001265 | 57 | Hyporeflexia |
| HP:0001284 | 57 | Areflexia |
| HP:0003380 | 57 | Decreased number of peripheral myelinated nerve fibers |
| HP:0007460 | 57 | Autoamputation of digits |
| HP:0002661 | 57 | Painless fractures due to injury |
| HP:0007021 | 57 | Pain insensitivity |
| HP:0008000 | 57 | Decreased corneal reflex |
| HP:0000522 | 57 | Alacrima |
| HP:0000224 | 57 | Decreased taste sensation |
| HP:0001069 | 57 | Episodic hyperhidrosis |
| HP:0006121 | 57 | Acral ulceration |
| HP:0012211 | 57 | Abnormal renal physiology |

---


---


---

# Cluster \*59

| HPO | Cluster | Description |
| --- | --- | --- |
| HP:0001270 | 59 | Motor delay |
| HP:0000508 | 59 | Ptosis |
| HP:0000218 | 59 | High palate |
| HP:0002650 | 59 | Scoliosis |
| HP:0001283 | 59 | Bulbar palsy |
| HP:0002804 | 59 | Arthrogryposis multiplex congenita |
| HP:0001561 | 59 | Polyhydramnios |
| HP:0003701 | 59 | Proximal muscle weakness |
| HP:0010628 | 59 | Facial palsy |
| HP:0001644 | 59 | Dilated cardiomyopathy |
| HP:0001319 | 59 | Neonatal hypotonia |
| HP:0001371 | 59 | Flexion contracture |
| HP:0003324 | 59 | Generalized muscle weakness |
| HP:0003798 | 59 | Nemaline bodies |
| HP:0001612 | 59 | Weak cry |
| HP:0000467 | 59 | Neck muscle weakness |
| HP:0002093 | 59 | Respiratory insufficiency |
| HP:0007126 | 59 | Proximal amyotrophy |
| HP:0002058 | 59 | Myopathic facies |
| HP:0003306 | 59 | Spinal rigidity |
| HP:0032341 | 59 | Reduced forced vital capacity |

---


---


---

# Cluster \*60

| HPO | Cluster | Description |
| --- | --- | --- |
| HP:0001761 | 60 | Pes cavus |
| HP:0003677 | 60 | Slow progression |
| HP:0003693 | 60 | Distal amyotrophy |
| HP:0001765 | 60 | Hammertoe |
| HP:0003392 | 60 | First dorsal interossei muscle weakness |
| HP:0003426 | 60 | First dorsal interossei muscle atrophy |
| HP:0003427 | 60 | Thenar muscle weakness |
| HP:0003435 | 60 | Cold-induced hand cramps |

---


---


---

# Cluster \*61

| HPO | Cluster | Description |
| --- | --- | --- |
| HP:0003676 | 61 | Progressive |
| HP:0001283 | 61 | Bulbar palsy |
| HP:0003581 | 61 | Adult onset |
| HP:0001265 | 61 | Hyporeflexia |
| HP:0002380 | 61 | Fasciculations |
| HP:0003394 | 61 | Muscle spasm |
| HP:0001761 | 61 | Pes cavus |
| HP:0002015 | 61 | Dysphagia |
| HP:0003677 | 61 | Slow progression |
| HP:0003693 | 61 | Distal amyotrophy |
| HP:0003701 | 61 | Proximal muscle weakness |
| HP:0001284 | 61 | Areflexia |
| HP:0002460 | 61 | Distal muscle weakness |
| HP:0002936 | 61 | Distal sensory impairment |
| HP:0003431 | 61 | Decreased motor nerve conduction velocity |
| HP:0009027 | 61 | Foot dorsiflexor weakness |
| HP:0002355 | 61 | Difficulty walking |
| HP:0009046 | 61 | Difficulty running |
| HP:0002747 | 61 | Respiratory insufficiency due to muscle weakness |
| HP:0008981 | 61 | Calf muscle hypertrophy |
| HP:0007126 | 61 | Proximal amyotrophy |

---


---


---

# Cluster \*62

| HPO | Cluster | Description |
| --- | --- | --- |
| HP:0001270 | 62 | Motor delay |
| HP:0000508 | 62 | Ptosis |
| HP:0003828 | 62 | Variable expressivity |
| HP:0003307 | 62 | Hyperlordosis |
| HP:0010628 | 62 | Facial palsy |
| HP:0001319 | 62 | Neonatal hypotonia |
| HP:0001371 | 62 | Flexion contracture |
| HP:0003798 | 62 | Nemaline bodies |
| HP:0001612 | 62 | Weak cry |
| HP:0002515 | 62 | Waddling gait |
| HP:0002355 | 62 | Difficulty walking |
| HP:0002747 | 62 | Respiratory insufficiency due to muscle weakness |
| HP:0003547 | 62 | Shoulder girdle muscle weakness |
| HP:0008981 | 62 | Calf muscle hypertrophy |
| HP:0003749 | 62 | Pelvic girdle muscle weakness |
| HP:0003391 | 62 | Gowers sign |
| HP:0000467 | 62 | Neck muscle weakness |
| HP:0002093 | 62 | Respiratory insufficiency |
| HP:0003554 | 62 | Type 2 muscle fiber atrophy |
| HP:0003551 | 62 | Difficulty climbing stairs |
| HP:0002715 | 62 | Abnormality of the immune system |
| HP:0000597 | 62 | Ophthalmoparesis |
| HP:0003403 | 62 | EMG: decremental response of compound muscle action potential to repetitive nerve stimulation |
| HP:0003473 | 62 | Fatigable weakness |

---


---


---

# Cluster \*63

| HPO | Cluster | Description |
| --- | --- | --- |
| HP:0001270 | 63 | Motor delay |
| HP:0000508 | 63 | Ptosis |
| HP:0000218 | 63 | High palate |
| HP:0003202 | 63 | Skeletal muscle atrophy |
| HP:0003676 | 63 | Progressive |
| HP:0001265 | 63 | Hyporeflexia |
| HP:0003477 | 63 | Peripheral axonal neuropathy |
| HP:0001188 | 63 | Hand clenching |
| HP:0002804 | 63 | Arthrogryposis multiplex congenita |
| HP:0000602 | 63 | Ophthalmoplegia |
| HP:0001561 | 63 | Polyhydramnios |
| HP:0003690 | 63 | Limb muscle weakness |
| HP:0003798 | 63 | Nemaline bodies |
| HP:0001284 | 63 | Areflexia |
| HP:0003431 | 63 | Decreased motor nerve conduction velocity |
| HP:0001349 | 63 | Facial diplegia |
| HP:0003458 | 63 | EMG: myopathic abnormalities |
| HP:0001558 | 63 | Decreased fetal movement |
| HP:0002747 | 63 | Respiratory insufficiency due to muscle weakness |
| HP:0003391 | 63 | Gowers sign |
| HP:0000467 | 63 | Neck muscle weakness |
| HP:0002058 | 63 | Myopathic facies |

---


---


---

# Cluster \*64

| HPO | Cluster | Description |
| --- | --- | --- |
| HP:0001270 | 64 | Motor delay |
| HP:0000218 | 64 | High palate |
| HP:0002650 | 64 | Scoliosis |
| HP:0001761 | 64 | Pes cavus |
| HP:0003677 | 64 | Slow progression |
| HP:0003325 | 64 | Limb-girdle muscle weakness |
| HP:0001319 | 64 | Neonatal hypotonia |
| HP:0001371 | 64 | Flexion contracture |
| HP:0003803 | 64 | Type 1 muscle fiber predominance |
| HP:0003691 | 64 | Scapular winging |
| HP:0003391 | 64 | Gowers sign |
| HP:0008180 | 64 | Mildly elevated creatine kinase |
| HP:0003306 | 64 | Spinal rigidity |
| HP:0020152 | 64 | Distal joint laxity |

---


---


---

# Cluster \*65

| HPO | Cluster | Description |
| --- | --- | --- |
| HP:0000508 | 65 | Ptosis |
| HP:0003676 | 65 | Progressive |
| HP:0001260 | 65 | Dysarthria |
| HP:0003394 | 65 | Muscle spasm |
| HP:0002015 | 65 | Dysphagia |
| HP:0003677 | 65 | Slow progression |
| HP:0000544 | 65 | External ophthalmoplegia |
| HP:0003324 | 65 | Generalized muscle weakness |
| HP:0003701 | 65 | Proximal muscle weakness |
| HP:0010628 | 65 | Facial palsy |
| HP:0001284 | 65 | Areflexia |
| HP:0003458 | 65 | EMG: myopathic abnormalities |
| HP:0003557 | 65 | Increased variability in muscle fiber diameter |
| HP:0003388 | 65 | Easy fatigability |
| HP:0000590 | 65 | Progressive external ophthalmoplegia |
| HP:0009046 | 65 | Difficulty running |
| HP:0000467 | 65 | Neck muscle weakness |
| HP:0002093 | 65 | Respiratory insufficiency |
| HP:0002058 | 65 | Myopathic facies |

---


---


---

# Cluster \*66

| HPO | Cluster | Description |
| --- | --- | --- |
| HP:0009830 | 66 | Peripheral neuropathy |
| HP:0001283 | 66 | Bulbar palsy |
| HP:0001265 | 66 | Hyporeflexia |
| HP:0002380 | 66 | Fasciculations |
| HP:0001761 | 66 | Pes cavus |
| HP:0003677 | 66 | Slow progression |
| HP:0003693 | 66 | Distal amyotrophy |
| HP:0003690 | 66 | Limb muscle weakness |
| HP:0003701 | 66 | Proximal muscle weakness |
| HP:0003307 | 66 | Hyperlordosis |
| HP:0010628 | 66 | Facial palsy |
| HP:0003324 | 66 | Generalized muscle weakness |
| HP:0003798 | 66 | Nemaline bodies |
| HP:0003803 | 66 | Type 1 muscle fiber predominance |
| HP:0001284 | 66 | Areflexia |
| HP:0002460 | 66 | Distal muscle weakness |
| HP:0002936 | 66 | Distal sensory impairment |
| HP:0003376 | 66 | Steppage gait |
| HP:0011096 | 66 | Peripheral demyelination |
| HP:0003458 | 66 | EMG: myopathic abnormalities |
| HP:0003200 | 66 | Ragged-red muscle fibers |
| HP:0001533 | 66 | Slender build |
| HP:0002515 | 66 | Waddling gait |
| HP:0000762 | 66 | Decreased nerve conduction velocity |
| HP:0007340 | 66 | Lower limb muscle weakness |
| HP:0002359 | 66 | Frequent falls |
| HP:0002355 | 66 | Difficulty walking |
| HP:0003445 | 66 | EMG: neuropathic changes |
| HP:0001558 | 66 | Decreased fetal movement |
| HP:0002747 | 66 | Respiratory insufficiency due to muscle weakness |
| HP:0003805 | 66 | Rimmed vacuoles |
| HP:0008180 | 66 | Mildly elevated creatine kinase |
| HP:0007126 | 66 | Proximal amyotrophy |
| HP:0002058 | 66 | Myopathic facies |
| HP:0003306 | 66 | Spinal rigidity |
| HP:0003722 | 66 | Neck flexor weakness |
| HP:0003810 | 66 | Late-onset distal muscle weakness |
| HP:0003555 | 66 | Muscle fiber splitting |

---


---


---

# Cluster \*68

| HPO | Cluster | Description |
| --- | --- | --- |
| HP:0001283 | 68 | Bulbar palsy |
| HP:0003701 | 68 | Proximal muscle weakness |
| HP:0010628 | 68 | Facial palsy |
| HP:0001319 | 68 | Neonatal hypotonia |
| HP:0003324 | 68 | Generalized muscle weakness |
| HP:0003798 | 68 | Nemaline bodies |
| HP:0003803 | 68 | Type 1 muscle fiber predominance |
| HP:0001612 | 68 | Weak cry |
| HP:0003458 | 68 | EMG: myopathic abnormalities |
| HP:0002359 | 68 | Frequent falls |
| HP:0002747 | 68 | Respiratory insufficiency due to muscle weakness |
| HP:0003391 | 68 | Gowers sign |
| HP:0000467 | 68 | Neck muscle weakness |
| HP:0003554 | 68 | Type 2 muscle fiber atrophy |
| HP:0002058 | 68 | Myopathic facies |
| HP:0003306 | 68 | Spinal rigidity |
| HP:0003722 | 68 | Neck flexor weakness |
| HP:0003810 | 68 | Late-onset distal muscle weakness |
| HP:0002033 | 68 | Poor suck |
| HP:0002715 | 68 | Abnormality of the immune system |
| HP:0000597 | 68 | Ophthalmoparesis |
| HP:0002872 | 68 | Apneic episodes precipitated by illness, fatigue, stress |
| HP:0002882 | 68 | Sudden episodic apnea |
| HP:0003397 | 68 | Generalized hypotonia due to defect at the neuromuscular junction |
| HP:0003402 | 68 | Decreased miniature endplate potentials |
| HP:0003403 | 68 | EMG: decremental response of compound muscle action potential to repetitive nerve stimulation |
| HP:0003473 | 68 | Fatigable weakness |

---


---


---

# Cluster \*69

| HPO | Cluster | Description |
| --- | --- | --- |
| HP:0001762 | 69 | Talipes equinovarus |
| HP:0001270 | 69 | Motor delay |
| HP:0000508 | 69 | Ptosis |
| HP:0000218 | 69 | High palate |
| HP:0002751 | 69 | Kyphoscoliosis |
| HP:0003202 | 69 | Skeletal muscle atrophy |
| HP:0001171 | 69 | Split hand |
| HP:0001425 | 69 | Heterogeneous |
| HP:0001265 | 69 | Hyporeflexia |
| HP:0001761 | 69 | Pes cavus |
| HP:0003693 | 69 | Distal amyotrophy |
| HP:0003690 | 69 | Limb muscle weakness |
| HP:0001371 | 69 | Flexion contracture |
| HP:0001178 | 69 | Ulnar claw |
| HP:0001284 | 69 | Areflexia |
| HP:0001765 | 69 | Hammertoe |
| HP:0002460 | 69 | Distal muscle weakness |
| HP:0002936 | 69 | Distal sensory impairment |
| HP:0003376 | 69 | Steppage gait |
| HP:0003380 | 69 | Decreased number of peripheral myelinated nerve fibers |
| HP:0003382 | 69 | Hypertrophic nerve changes |
| HP:0003383 | 69 | Onion bulb formation |
| HP:0003431 | 69 | Decreased motor nerve conduction velocity |
| HP:0003449 | 69 | Cold-induced muscle cramps |
| HP:0003621 | 69 | Juvenile onset |
| HP:0004336 | 69 | Myelin outfoldings |
| HP:0009027 | 69 | Foot dorsiflexor weakness |
| HP:0003481 | 69 | Segmental peripheral demyelination/remyelination |
| HP:0002355 | 69 | Difficulty walking |

---


---


---

# Cluster \*70

| HPO | Cluster | Description |
| --- | --- | --- |
| HP:0001265 | 70 | Hyporeflexia |
| HP:0003677 | 70 | Slow progression |
| HP:0003701 | 70 | Proximal muscle weakness |
| HP:0010628 | 70 | Facial palsy |
| HP:0003324 | 70 | Generalized muscle weakness |
| HP:0003798 | 70 | Nemaline bodies |
| HP:0003803 | 70 | Type 1 muscle fiber predominance |
| HP:0001284 | 70 | Areflexia |
| HP:0009027 | 70 | Foot dorsiflexor weakness |
| HP:0003458 | 70 | EMG: myopathic abnormalities |
| HP:0003546 | 70 | Exercise intolerance |
| HP:0003557 | 70 | Increased variability in muscle fiber diameter |
| HP:0002058 | 70 | Myopathic facies |
| HP:0003722 | 70 | Neck flexor weakness |
| HP:0003810 | 70 | Late-onset distal muscle weakness |

---


---


---

# Cluster \*71

| HPO | Cluster | Description |
| --- | --- | --- |
| HP:0003828 | 71 | Variable expressivity |
| HP:0003676 | 71 | Progressive |
| HP:0001265 | 71 | Hyporeflexia |
| HP:0001288 | 71 | Gait disturbance |
| HP:0001761 | 71 | Pes cavus |
| HP:0002015 | 71 | Dysphagia |
| HP:0003677 | 71 | Slow progression |
| HP:0000544 | 71 | External ophthalmoplegia |
| HP:0003693 | 71 | Distal amyotrophy |
| HP:0003690 | 71 | Limb muscle weakness |
| HP:0003701 | 71 | Proximal muscle weakness |
| HP:0001284 | 71 | Areflexia |
| HP:0002460 | 71 | Distal muscle weakness |
| HP:0002936 | 71 | Distal sensory impairment |
| HP:0003383 | 71 | Onion bulb formation |
| HP:0009027 | 71 | Foot dorsiflexor weakness |
| HP:0003326 | 71 | Myalgia |
| HP:0003546 | 71 | Exercise intolerance |
| HP:0001618 | 71 | Dysphonia |
| HP:0003805 | 71 | Rimmed vacuoles |
| HP:0008180 | 71 | Mildly elevated creatine kinase |
| HP:0007126 | 71 | Proximal amyotrophy |
| HP:0003551 | 71 | Difficulty climbing stairs |

---


---


---

# Cluster \*73

| HPO | Cluster | Description |
| --- | --- | --- |
| HP:0003676 | 73 | Progressive |
| HP:0001260 | 73 | Dysarthria |
| HP:0001265 | 73 | Hyporeflexia |
| HP:0001324 | 73 | Muscle weakness |
| HP:0001761 | 73 | Pes cavus |
| HP:0003693 | 73 | Distal amyotrophy |
| HP:0002936 | 73 | Distal sensory impairment |
| HP:0003621 | 73 | Juvenile onset |
| HP:0003390 | 73 | Sensory axonal neuropathy |
| HP:0007002 | 73 | Motor axonal neuropathy |

---


---


---

# Cluster \*74

| HPO | Cluster | Description |
| --- | --- | --- |
| HP:0003202 | 74 | Skeletal muscle atrophy |
| HP:0003581 | 74 | Adult onset |
| HP:0003678 | 74 | Rapidly progressive |
| HP:0001288 | 74 | Gait disturbance |
| HP:0003307 | 74 | Hyperlordosis |
| HP:0001371 | 74 | Flexion contracture |
| HP:0001284 | 74 | Areflexia |
| HP:0003236 | 74 | Elevated serum creatine kinase |
| HP:0003560 | 74 | Muscular dystrophy |
| HP:0002747 | 74 | Respiratory insufficiency due to muscle weakness |
| HP:0003551 | 74 | Difficulty climbing stairs |

---


---


---

# Cluster \*75

| HPO | Cluster | Description |
| --- | --- | --- |
| HP:0001270 | 75 | Motor delay |
| HP:0003828 | 75 | Variable expressivity |
| HP:0001283 | 75 | Bulbar palsy |
| HP:0001265 | 75 | Hyporeflexia |
| HP:0002804 | 75 | Arthrogryposis multiplex congenita |
| HP:0003677 | 75 | Slow progression |
| HP:0003701 | 75 | Proximal muscle weakness |
| HP:0003307 | 75 | Hyperlordosis |
| HP:0010628 | 75 | Facial palsy |
| HP:0001319 | 75 | Neonatal hypotonia |
| HP:0001371 | 75 | Flexion contracture |
| HP:0003324 | 75 | Generalized muscle weakness |
| HP:0001284 | 75 | Areflexia |
| HP:0001612 | 75 | Weak cry |
| HP:0002515 | 75 | Waddling gait |
| HP:0003388 | 75 | Easy fatigability |
| HP:0002359 | 75 | Frequent falls |
| HP:0002355 | 75 | Difficulty walking |
| HP:0009046 | 75 | Difficulty running |
| HP:0001558 | 75 | Decreased fetal movement |
| HP:0002747 | 75 | Respiratory insufficiency due to muscle weakness |
| HP:0008981 | 75 | Calf muscle hypertrophy |
| HP:0003391 | 75 | Gowers sign |
| HP:0002093 | 75 | Respiratory insufficiency |
| HP:0003554 | 75 | Type 2 muscle fiber atrophy |
| HP:0003306 | 75 | Spinal rigidity |
| HP:0003551 | 75 | Difficulty climbing stairs |
| HP:0002033 | 75 | Poor suck |
| HP:0002715 | 75 | Abnormality of the immune system |
| HP:0000597 | 75 | Ophthalmoparesis |
| HP:0003402 | 75 | Decreased miniature endplate potentials |
| HP:0003403 | 75 | EMG: decremental response of compound muscle action potential to repetitive nerve stimulation |
| HP:0003473 | 75 | Fatigable weakness |
| HP:0003443 | 75 | Decreased size of nerve terminals |

---


---


---

# Cluster \*76

| HPO | Cluster | Description |
| --- | --- | --- |
| HP:0000007 | 76 | Autosomal recessive inheritance |
| HP:0001762 | 76 | Talipes equinovarus |
| HP:0001270 | 76 | Motor delay |
| HP:0000508 | 76 | Ptosis |
| HP:0003828 | 76 | Variable expressivity |
| HP:0002650 | 76 | Scoliosis |
| HP:0003202 | 76 | Skeletal muscle atrophy |
| HP:0001283 | 76 | Bulbar palsy |
| HP:0001265 | 76 | Hyporeflexia |
| HP:0001324 | 76 | Muscle weakness |
| HP:0003477 | 76 | Peripheral axonal neuropathy |
| HP:0003394 | 76 | Muscle spasm |
| HP:0002804 | 76 | Arthrogryposis multiplex congenita |
| HP:0000602 | 76 | Ophthalmoplegia |
| HP:0001761 | 76 | Pes cavus |
| HP:0003677 | 76 | Slow progression |
| HP:0003198 | 76 | Myopathy |
| HP:0003690 | 76 | Limb muscle weakness |
| HP:0003701 | 76 | Proximal muscle weakness |
| HP:0010628 | 76 | Facial palsy |
| HP:0001371 | 76 | Flexion contracture |
| HP:0003798 | 76 | Nemaline bodies |
| HP:0001284 | 76 | Areflexia |
| HP:0002460 | 76 | Distal muscle weakness |
| HP:0002936 | 76 | Distal sensory impairment |
| HP:0003431 | 76 | Decreased motor nerve conduction velocity |
| HP:0009027 | 76 | Foot dorsiflexor weakness |
| HP:0003560 | 76 | Muscular dystrophy |
| HP:0003546 | 76 | Exercise intolerance |
| HP:0001618 | 76 | Dysphonia |
| HP:0003388 | 76 | Easy fatigability |
| HP:0011968 | 76 | Feeding difficulties |
| HP:0003691 | 76 | Scapular winging |
| HP:0000590 | 76 | Progressive external ophthalmoplegia |
| HP:0003323 | 76 | Progressive muscle weakness |
| HP:0003713 | 76 | Muscle fiber necrosis |
| HP:0002355 | 76 | Difficulty walking |
| HP:0001558 | 76 | Decreased fetal movement |
| HP:0002747 | 76 | Respiratory insufficiency due to muscle weakness |
| HP:0008981 | 76 | Calf muscle hypertrophy |
| HP:0003391 | 76 | Gowers sign |
| HP:0003687 | 76 | Centrally nucleated skeletal muscle fibers |
| HP:0002093 | 76 | Respiratory insufficiency |
| HP:0002058 | 76 | Myopathic facies |
| HP:0009025 | 76 | Increased connective tissue |

---


---


---

# Cluster \*77

| HPO | Cluster | Description |
| --- | --- | --- |
| HP:0001638 | 77 | Cardiomyopathy |
| HP:0011675 | 77 | Arrhythmia |
| HP:0003307 | 77 | Hyperlordosis |
| HP:0001644 | 77 | Dilated cardiomyopathy |
| HP:0003458 | 77 | EMG: myopathic abnormalities |
| HP:0002515 | 77 | Waddling gait |
| HP:0003691 | 77 | Scapular winging |
| HP:0003713 | 77 | Muscle fiber necrosis |
| HP:0003724 | 77 | Shoulder girdle muscle atrophy |
| HP:0003391 | 77 | Gowers sign |
| HP:0003687 | 77 | Centrally nucleated skeletal muscle fibers |
| HP:0008988 | 77 | Pelvic girdle muscle atrophy |
| HP:0003704 | 77 | Scapuloperoneal weakness |
| HP:0003697 | 77 | Scapuloperoneal amyotrophy |
| HP:0003707 | 77 | Calf muscle pseudohypertrophy |
| HP:0003731 | 77 | Quadriceps muscle weakness |
| HP:0002792 | 77 | Reduced vital capacity |

---


---


---

# Cluster \*78

| HPO | Cluster | Description |
| --- | --- | --- |
| HP:0001638 | 78 | Cardiomyopathy |
| HP:0001324 | 78 | Muscle weakness |
| HP:0001649 | 78 | Tachycardia |
| HP:0001962 | 78 | Palpitations |
| HP:0003768 | 78 | Periodic paralysis |
| HP:0003201 | 78 | Rhabdomyolysis |
| HP:0003326 | 78 | Myalgia |

---


---


---

# Cluster \*79

| HPO | Cluster | Description |
| --- | --- | --- |
| HP:0003677 | 79 | Slow progression |
| HP:0003701 | 79 | Proximal muscle weakness |
| HP:0003307 | 79 | Hyperlordosis |
| HP:0010628 | 79 | Facial palsy |
| HP:0003798 | 79 | Nemaline bodies |
| HP:0001284 | 79 | Areflexia |
| HP:0002460 | 79 | Distal muscle weakness |
| HP:0009027 | 79 | Foot dorsiflexor weakness |
| HP:0003458 | 79 | EMG: myopathic abnormalities |
| HP:0003557 | 79 | Increased variability in muscle fiber diameter |
| HP:0003691 | 79 | Scapular winging |
| HP:0002747 | 79 | Respiratory insufficiency due to muscle weakness |
| HP:0003547 | 79 | Shoulder girdle muscle weakness |
| HP:0008981 | 79 | Calf muscle hypertrophy |
| HP:0003749 | 79 | Pelvic girdle muscle weakness |
| HP:0003687 | 79 | Centrally nucleated skeletal muscle fibers |
| HP:0003805 | 79 | Rimmed vacuoles |
| HP:0003306 | 79 | Spinal rigidity |
| HP:0003810 | 79 | Late-onset distal muscle weakness |
| HP:0003551 | 79 | Difficulty climbing stairs |
| HP:0001771 | 79 | Achilles tendon contracture |
| HP:0003555 | 79 | Muscle fiber splitting |
| HP:0009025 | 79 | Increased connective tissue |

---


---


---

# Cluster \*81

| HPO | Cluster | Description |
| --- | --- | --- |
| HP:0000007 | 81 | Autosomal recessive inheritance |
| HP:0001762 | 81 | Talipes equinovarus |
| HP:0001270 | 81 | Motor delay |
| HP:0000508 | 81 | Ptosis |
| HP:0000218 | 81 | High palate |
| HP:0002650 | 81 | Scoliosis |
| HP:0003593 | 81 | Infantile onset |
| HP:0008872 | 81 | Feeding difficulties in infancy |
| HP:0001561 | 81 | Polyhydramnios |
| HP:0003690 | 81 | Limb muscle weakness |
| HP:0010628 | 81 | Facial palsy |
| HP:0001319 | 81 | Neonatal hypotonia |
| HP:0001371 | 81 | Flexion contracture |
| HP:0003324 | 81 | Generalized muscle weakness |
| HP:0003798 | 81 | Nemaline bodies |
| HP:0003803 | 81 | Type 1 muscle fiber predominance |
| HP:0001612 | 81 | Weak cry |
| HP:0001349 | 81 | Facial diplegia |
| HP:0003128 | 81 | Lactic acidosis |
| HP:0002747 | 81 | Respiratory insufficiency due to muscle weakness |
| HP:0002093 | 81 | Respiratory insufficiency |
| HP:0002058 | 81 | Myopathic facies |
| HP:0002033 | 81 | Poor suck |
| HP:0008972 | 81 | Decreased activity of mitochondrial respiratory chain |
| HP:0003403 | 81 | EMG: decremental response of compound muscle action potential to repetitive nerve stimulation |
| HP:0003327 | 81 | Axial muscle weakness |

---


---


---

# Cluster \*83

| HPO | Cluster | Description |
| --- | --- | --- |
| HP:0000007 | 83 | Autosomal recessive inheritance |
| HP:0009830 | 83 | Peripheral neuropathy |
| HP:0000508 | 83 | Ptosis |
| HP:0000218 | 83 | High palate |
| HP:0000602 | 83 | Ophthalmoplegia |
| HP:0003198 | 83 | Myopathy |
| HP:0003701 | 83 | Proximal muscle weakness |
| HP:0010628 | 83 | Facial palsy |
| HP:0001319 | 83 | Neonatal hypotonia |
| HP:0001371 | 83 | Flexion contracture |
| HP:0003324 | 83 | Generalized muscle weakness |
| HP:0003200 | 83 | Ragged-red muscle fibers |
| HP:0003388 | 83 | Easy fatigability |
| HP:0003390 | 83 | Sensory axonal neuropathy |
| HP:0011968 | 83 | Feeding difficulties |
| HP:0001558 | 83 | Decreased fetal movement |
| HP:0002747 | 83 | Respiratory insufficiency due to muscle weakness |
| HP:0003687 | 83 | Centrally nucleated skeletal muscle fibers |
| HP:0000467 | 83 | Neck muscle weakness |
| HP:0002093 | 83 | Respiratory insufficiency |
| HP:0006829 | 83 | Severe muscular hypotonia |

---


---


---

# Cluster \*84

| HPO | Cluster | Description |
| --- | --- | --- |
| HP:0001270 | 84 | Motor delay |
| HP:0003828 | 84 | Variable expressivity |
| HP:0002650 | 84 | Scoliosis |
| HP:0003676 | 84 | Progressive |
| HP:0001260 | 84 | Dysarthria |
| HP:0001265 | 84 | Hyporeflexia |
| HP:0002380 | 84 | Fasciculations |
| HP:0040078 | 84 | Axonal degeneration |
| HP:0001761 | 84 | Pes cavus |
| HP:0003693 | 84 | Distal amyotrophy |
| HP:0002460 | 84 | Distal muscle weakness |
| HP:0003431 | 84 | Decreased motor nerve conduction velocity |
| HP:0002359 | 84 | Frequent falls |
| HP:0002355 | 84 | Difficulty walking |
| HP:0003547 | 84 | Shoulder girdle muscle weakness |
| HP:0008981 | 84 | Calf muscle hypertrophy |
| HP:0003391 | 84 | Gowers sign |
| HP:0000764 | 84 | Peripheral axonal degeneration |
| HP:0003551 | 84 | Difficulty climbing stairs |
| HP:0003741 | 84 | Congenital muscular dystrophy |
| HP:0040083 | 84 | Toe walking |

---


---


---

# Cluster \*86

| HPO | Cluster | Description |
| --- | --- | --- |
| HP:0002380 | 86 | Fasciculations |
| HP:0003394 | 86 | Muscle spasm |
| HP:0002936 | 86 | Distal sensory impairment |
| HP:0009027 | 86 | Foot dorsiflexor weakness |
| HP:0002411 | 86 | Myokymia |

---


---


---

# Cluster \*87

| HPO | Cluster | Description |
| --- | --- | --- |
| HP:0000508 | 87 | Ptosis |
| HP:0003677 | 87 | Slow progression |
| HP:0003198 | 87 | Myopathy |
| HP:0003326 | 87 | Myalgia |
| HP:0003690 | 87 | Limb muscle weakness |
| HP:0003701 | 87 | Proximal muscle weakness |
| HP:0010628 | 87 | Facial palsy |
| HP:0001319 | 87 | Neonatal hypotonia |
| HP:0001371 | 87 | Flexion contracture |
| HP:0003324 | 87 | Generalized muscle weakness |
| HP:0003798 | 87 | Nemaline bodies |
| HP:0003737 | 87 | Mitochondrial myopathy |
| HP:0003200 | 87 | Ragged-red muscle fibers |
| HP:0003201 | 87 | Rhabdomyolysis |
| HP:0003546 | 87 | Exercise intolerance |
| HP:0003557 | 87 | Increased variability in muscle fiber diameter |
| HP:0002515 | 87 | Waddling gait |
| HP:0003388 | 87 | Easy fatigability |
| HP:0003691 | 87 | Scapular winging |
| HP:0000590 | 87 | Progressive external ophthalmoplegia |
| HP:0009046 | 87 | Difficulty running |
| HP:0002747 | 87 | Respiratory insufficiency due to muscle weakness |
| HP:0003749 | 87 | Pelvic girdle muscle weakness |
| HP:0003391 | 87 | Gowers sign |
| HP:0003687 | 87 | Centrally nucleated skeletal muscle fibers |
| HP:0003805 | 87 | Rimmed vacuoles |
| HP:0008180 | 87 | Mildly elevated creatine kinase |
| HP:0002093 | 87 | Respiratory insufficiency |
| HP:0003554 | 87 | Type 2 muscle fiber atrophy |
| HP:0007126 | 87 | Proximal amyotrophy |
| HP:0002058 | 87 | Myopathic facies |
| HP:0003722 | 87 | Neck flexor weakness |
| HP:0003551 | 87 | Difficulty climbing stairs |
| HP:0003555 | 87 | Muscle fiber splitting |
| HP:0003403 | 87 | EMG: decremental response of compound muscle action potential to repetitive nerve stimulation |

---


---


---

# Cluster \*88

| HPO | Cluster | Description |
| --- | --- | --- |
| HP:0001270 | 88 | Motor delay |
| HP:0001319 | 88 | Neonatal hypotonia |
| HP:0001171 | 88 | Split hand |
| HP:0001265 | 88 | Hyporeflexia |
| HP:0040078 | 88 | Axonal degeneration |
| HP:0001288 | 88 | Gait disturbance |
| HP:0001761 | 88 | Pes cavus |
| HP:0003677 | 88 | Slow progression |
| HP:0003693 | 88 | Distal amyotrophy |
| HP:0003690 | 88 | Limb muscle weakness |
| HP:0001371 | 88 | Flexion contracture |
| HP:0003803 | 88 | Type 1 muscle fiber predominance |
| HP:0001178 | 88 | Ulnar claw |
| HP:0001284 | 88 | Areflexia |
| HP:0002460 | 88 | Distal muscle weakness |
| HP:0002936 | 88 | Distal sensory impairment |
| HP:0003380 | 88 | Decreased number of peripheral myelinated nerve fibers |
| HP:0003382 | 88 | Hypertrophic nerve changes |
| HP:0003383 | 88 | Onion bulb formation |
| HP:0003431 | 88 | Decreased motor nerve conduction velocity |
| HP:0003481 | 88 | Segmental peripheral demyelination/remyelination |
| HP:0001533 | 88 | Slender build |
| HP:0002359 | 88 | Frequent falls |
| HP:0002355 | 88 | Difficulty walking |
| HP:0002058 | 88 | Myopathic facies |
| HP:0003810 | 88 | Late-onset distal muscle weakness |
| HP:0000764 | 88 | Peripheral axonal degeneration |
| HP:0001771 | 88 | Achilles tendon contracture |
| HP:0003400 | 88 | Basal lamina onion bulb formation |
| HP:0007182 | 88 | Peripheral hypomyelination |
| HP:0007141 | 88 | Sensorimotor neuropathy |
| HP:0003484 | 88 | Upper limb muscle weakness |

---


---


---

# Cluster \*90

| HPO | Cluster | Description |
| --- | --- | --- |
| HP:0001283 | 90 | Bulbar palsy |
| HP:0003581 | 90 | Adult onset |
| HP:0001265 | 90 | Hyporeflexia |
| HP:0003477 | 90 | Peripheral axonal neuropathy |
| HP:0001288 | 90 | Gait disturbance |
| HP:0001761 | 90 | Pes cavus |
| HP:0003677 | 90 | Slow progression |
| HP:0003693 | 90 | Distal amyotrophy |
| HP:0003690 | 90 | Limb muscle weakness |
| HP:0003701 | 90 | Proximal muscle weakness |
| HP:0010628 | 90 | Facial palsy |
| HP:0001284 | 90 | Areflexia |
| HP:0002460 | 90 | Distal muscle weakness |
| HP:0002936 | 90 | Distal sensory impairment |
| HP:0009027 | 90 | Foot dorsiflexor weakness |
| HP:0003326 | 90 | Myalgia |
| HP:0003458 | 90 | EMG: myopathic abnormalities |
| HP:0003200 | 90 | Ragged-red muscle fibers |
| HP:0003546 | 90 | Exercise intolerance |
| HP:0003557 | 90 | Increased variability in muscle fiber diameter |
| HP:0001618 | 90 | Dysphonia |
| HP:0003390 | 90 | Sensory axonal neuropathy |
| HP:0000590 | 90 | Progressive external ophthalmoplegia |
| HP:0003323 | 90 | Progressive muscle weakness |
| HP:0003548 | 90 | Subsarcolemmal accumulations of abnormally shaped mitochondria |
| HP:0003688 | 90 | Cytochrome C oxidase-negative muscle fibers |
| HP:0003713 | 90 | Muscle fiber necrosis |
| HP:0006886 | 90 | Impaired distal vibration sensation |
| HP:0009046 | 90 | Difficulty running |
| HP:0002747 | 90 | Respiratory insufficiency due to muscle weakness |
| HP:0003547 | 90 | Shoulder girdle muscle weakness |
| HP:0003749 | 90 | Pelvic girdle muscle weakness |
| HP:0000467 | 90 | Neck muscle weakness |
| HP:0003805 | 90 | Rimmed vacuoles |
| HP:0008180 | 90 | Mildly elevated creatine kinase |
| HP:0007126 | 90 | Proximal amyotrophy |
| HP:0003551 | 90 | Difficulty climbing stairs |
| HP:0000763 | 90 | Sensory neuropathy |
| HP:0003555 | 90 | Muscle fiber splitting |
| HP:0002403 | 90 | Positive Romberg sign |
| HP:0003434 | 90 | Sensory ataxic neuropathy |
| HP:0003715 | 90 | Myofibrillar myopathy |
| HP:0100303 | 90 | Muscle fiber cytoplasmatic inclusion bodies |

---


---


---

# Cluster \*92

| HPO | Cluster | Description |
| --- | --- | --- |
| HP:0001270 | 92 | Motor delay |
| HP:0001265 | 92 | Hyporeflexia |
| HP:0001761 | 92 | Pes cavus |
| HP:0003701 | 92 | Proximal muscle weakness |
| HP:0010628 | 92 | Facial palsy |
| HP:0001319 | 92 | Neonatal hypotonia |
| HP:0001371 | 92 | Flexion contracture |
| HP:0001284 | 92 | Areflexia |
| HP:0009027 | 92 | Foot dorsiflexor weakness |
| HP:0003200 | 92 | Ragged-red muscle fibers |
| HP:0003557 | 92 | Increased variability in muscle fiber diameter |
| HP:0003390 | 92 | Sensory axonal neuropathy |
| HP:0000590 | 92 | Progressive external ophthalmoplegia |
| HP:0003548 | 92 | Subsarcolemmal accumulations of abnormally shaped mitochondria |
| HP:0003688 | 92 | Cytochrome C oxidase-negative muscle fibers |
| HP:0003713 | 92 | Muscle fiber necrosis |
| HP:0006886 | 92 | Impaired distal vibration sensation |
| HP:0002355 | 92 | Difficulty walking |
| HP:0008981 | 92 | Calf muscle hypertrophy |
| HP:0002058 | 92 | Myopathic facies |
| HP:0003551 | 92 | Difficulty climbing stairs |
| HP:0002403 | 92 | Positive Romberg sign |
| HP:0003434 | 92 | Sensory ataxic neuropathy |

---


---


---

# Cluster \*93

| HPO | Cluster | Description |
| --- | --- | --- |
| HP:0003677 | 93 | Slow progression |
| HP:0003325 | 93 | Limb-girdle muscle weakness |
| HP:0003557 | 93 | Increased variability in muscle fiber diameter |
| HP:0003691 | 93 | Scapular winging |
| HP:0003547 | 93 | Shoulder girdle muscle weakness |
| HP:0003724 | 93 | Shoulder girdle muscle atrophy |
| HP:0008981 | 93 | Calf muscle hypertrophy |
| HP:0003749 | 93 | Pelvic girdle muscle weakness |
| HP:0003805 | 93 | Rimmed vacuoles |
| HP:0003722 | 93 | Neck flexor weakness |
| HP:0008988 | 93 | Pelvic girdle muscle atrophy |
| HP:0003555 | 93 | Muscle fiber splitting |
| HP:0002877 | 93 | Nocturnal hypoventilation |
| HP:0003731 | 93 | Quadriceps muscle weakness |
| HP:0003715 | 93 | Myofibrillar myopathy |
| HP:0002792 | 93 | Reduced vital capacity |

---


---


---

# Cluster \*94

| HPO | Cluster | Description |
| --- | --- | --- |
| HP:0000007 | 94 | Autosomal recessive inheritance |
| HP:0001302 | 94 | Pachygyria |
| HP:0001270 | 94 | Motor delay |
| HP:0000557 | 94 | Buphthalmos |
| HP:0003202 | 94 | Skeletal muscle atrophy |
| HP:0000518 | 94 | Cataract |
| HP:0002804 | 94 | Arthrogryposis multiplex congenita |
| HP:0003198 | 94 | Myopathy |
| HP:0010628 | 94 | Facial palsy |
| HP:0001371 | 94 | Flexion contracture |
| HP:0003324 | 94 | Generalized muscle weakness |
| HP:0003557 | 94 | Increased variability in muscle fiber diameter |
| HP:0011968 | 94 | Feeding difficulties |
| HP:0001558 | 94 | Decreased fetal movement |
| HP:0002747 | 94 | Respiratory insufficiency due to muscle weakness |
| HP:0000467 | 94 | Neck muscle weakness |
| HP:0002093 | 94 | Respiratory insufficiency |
| HP:0002058 | 94 | Myopathic facies |
| HP:0003306 | 94 | Spinal rigidity |
| HP:0002365 | 94 | Hypoplasia of the brainstem |
| HP:0003700 | 94 | Generalized amyotrophy |
| HP:0007973 | 94 | Retinal dysplasia |
| HP:0012110 | 94 | Hypoplasia of the pons |
| HP:0006829 | 94 | Severe muscular hypotonia |
| HP:0003741 | 94 | Congenital muscular dystrophy |
| HP:0007033 | 94 | Cerebellar dysplasia |
| HP:0007260 | 94 | Type II lissencephaly |
| HP:0031882 | 94 | Agyria |
| HP:0002350 | 94 | Cerebellar cyst |

---


---


---

# Cluster \*95

| HPO | Cluster | Description |
| --- | --- | --- |
| HP:0000007 | 95 | Autosomal recessive inheritance |
| HP:0003828 | 95 | Variable expressivity |
| HP:0000218 | 95 | High palate |
| HP:0002650 | 95 | Scoliosis |
| HP:0003202 | 95 | Skeletal muscle atrophy |
| HP:0003593 | 95 | Infantile onset |
| HP:0003676 | 95 | Progressive |
| HP:0003577 | 95 | Congenital onset |
| HP:0001319 | 95 | Neonatal hypotonia |
| HP:0001371 | 95 | Flexion contracture |
| HP:0003560 | 95 | Muscular dystrophy |
| HP:0001321 | 95 | Cerebellar hypoplasia |
| HP:0002878 | 95 | Respiratory failure |
| HP:0011968 | 95 | Feeding difficulties |
| HP:0001558 | 95 | Decreased fetal movement |
| HP:0002093 | 95 | Respiratory insufficiency |
| HP:0002058 | 95 | Myopathic facies |
| HP:0002490 | 95 | Increased CSF lactate |
| HP:0002421 | 95 | Poor head control |
| HP:0031882 | 95 | Agyria |
| HP:0008972 | 95 | Decreased activity of mitochondrial respiratory chain |
| HP:0002350 | 95 | Cerebellar cyst |

---


---


---

# Cluster \*96

| HPO | Cluster | Description |
| --- | --- | --- |
| HP:0001762 | 96 | Talipes equinovarus |
| HP:0001270 | 96 | Motor delay |
| HP:0002938 | 96 | Lumbar hyperlordosis |
| HP:0002650 | 96 | Scoliosis |
| HP:0002751 | 96 | Kyphoscoliosis |
| HP:0003418 | 96 | Back pain |
| HP:0003325 | 96 | Limb-girdle muscle weakness |
| HP:0003701 | 96 | Proximal muscle weakness |
| HP:0010628 | 96 | Facial palsy |
| HP:0002515 | 96 | Waddling gait |
| HP:0003551 | 96 | Difficulty climbing stairs |
| HP:0032341 | 96 | Reduced forced vital capacity |

---


---


---

# Cluster \*98

| HPO | Cluster | Description |
| --- | --- | --- |
| HP:0001270 | 98 | Motor delay |
| HP:0001761 | 98 | Pes cavus |
| HP:0001284 | 98 | Areflexia |
| HP:0003431 | 98 | Decreased motor nerve conduction velocity |
| HP:0007340 | 98 | Lower limb muscle weakness |
| HP:0003448 | 98 | Decreased sensory nerve conduction velocity |
| HP:0000763 | 98 | Sensory neuropathy |
| HP:0007178 | 98 | Motor polyneuropathy |
| HP:0003444 | 98 | EMG: chronic denervation signs |
| HP:0007182 | 98 | Peripheral hypomyelination |

---


---


---

# Cluster \*99

| HPO | Cluster | Description |
| --- | --- | --- |
| HP:0009830 | 99 | Peripheral neuropathy |
| HP:0003581 | 99 | Adult onset |
| HP:0000975 | 99 | Hyperhidrosis |
| HP:0001605 | 99 | Vocal cord paralysis |
| HP:0003390 | 99 | Sensory axonal neuropathy |

---


---


---

# Cluster \*100

| HPO | Cluster | Description |
| --- | --- | --- |
| HP:0001762 | 100 | Talipes equinovarus |
| HP:0002804 | 100 | Arthrogryposis multiplex congenita |
| HP:0002878 | 100 | Respiratory failure |
| HP:0007269 | 100 | Spinal muscular atrophy |
| HP:0001558 | 100 | Decreased fetal movement |
| HP:0009110 | 100 | Diaphragmatic eventration |
| HP:0006597 | 100 | Diaphragmatic paralysis |
| HP:0006829 | 100 | Severe muscular hypotonia |

---


---


---

# Cluster \*101

| HPO | Cluster | Description |
| --- | --- | --- |
| HP:0003477 | 101 | Peripheral axonal neuropathy |
| HP:0001761 | 101 | Pes cavus |
| HP:0003693 | 101 | Distal amyotrophy |
| HP:0002460 | 101 | Distal muscle weakness |
| HP:0002936 | 101 | Distal sensory impairment |
| HP:0001868 | 101 | Autoamputation of foot |
| HP:0001886 | 101 | Foot osteomyelitis |
| HP:0003387 | 101 | Decreased number of large peripheral myelinated nerve fibers |
| HP:0003448 | 101 | Decreased sensory nerve conduction velocity |
| HP:0006984 | 101 | Distal sensory loss of all modalities |
| HP:0007267 | 101 | Chronic axonal neuropathy |

---


---


---

# Cluster \*102

| HPO | Cluster | Description |
| --- | --- | --- |
| HP:0003593 | 102 | Infantile onset |
| HP:0002922 | 102 | Increased CSF protein |
| HP:0000970 | 102 | Anhidrosis |
| HP:0002661 | 102 | Painless fractures due to injury |
| HP:0007021 | 102 | Pain insensitivity |
| HP:0006121 | 102 | Acral ulceration |

---


---


---

# Cluster \*103

| HPO | Cluster | Description |
| --- | --- | --- |
| HP:0001270 | 103 | Motor delay |
| HP:0009830 | 103 | Peripheral neuropathy |
| HP:0003828 | 103 | Variable expressivity |
| HP:0003202 | 103 | Skeletal muscle atrophy |
| HP:0003676 | 103 | Progressive |
| HP:0003581 | 103 | Adult onset |
| HP:0002015 | 103 | Dysphagia |
| HP:0001265 | 103 | Hyporeflexia |
| HP:0003477 | 103 | Peripheral axonal neuropathy |
| HP:0001761 | 103 | Pes cavus |
| HP:0003693 | 103 | Distal amyotrophy |
| HP:0003690 | 103 | Limb muscle weakness |
| HP:0003701 | 103 | Proximal muscle weakness |
| HP:0010628 | 103 | Facial palsy |
| HP:0001371 | 103 | Flexion contracture |
| HP:0001284 | 103 | Areflexia |
| HP:0002460 | 103 | Distal muscle weakness |
| HP:0002936 | 103 | Distal sensory impairment |
| HP:0003458 | 103 | EMG: myopathic abnormalities |
| HP:0003200 | 103 | Ragged-red muscle fibers |
| HP:0001618 | 103 | Dysphonia |
| HP:0003390 | 103 | Sensory axonal neuropathy |
| HP:0000590 | 103 | Progressive external ophthalmoplegia |
| HP:0003323 | 103 | Progressive muscle weakness |
| HP:0003548 | 103 | Subsarcolemmal accumulations of abnormally shaped mitochondria |
| HP:0003688 | 103 | Cytochrome C oxidase-negative muscle fibers |
| HP:0003391 | 103 | Gowers sign |
| HP:0003306 | 103 | Spinal rigidity |
| HP:0003444 | 103 | EMG: chronic denervation signs |
| HP:0009025 | 103 | Increased connective tissue |
| HP:0007141 | 103 | Sensorimotor neuropathy |
| HP:0006937 | 103 | Impaired distal tactile sensation |

---


---


---

# Cluster \*104

| HPO | Cluster | Description |
| --- | --- | --- |
| HP:0003676 | 104 | Progressive |
| HP:0001265 | 104 | Hyporeflexia |
| HP:0003477 | 104 | Peripheral axonal neuropathy |
| HP:0002380 | 104 | Fasciculations |
| HP:0003394 | 104 | Muscle spasm |
| HP:0002804 | 104 | Arthrogryposis multiplex congenita |
| HP:0001761 | 104 | Pes cavus |
| HP:0003677 | 104 | Slow progression |
| HP:0003693 | 104 | Distal amyotrophy |
| HP:0003701 | 104 | Proximal muscle weakness |
| HP:0003307 | 104 | Hyperlordosis |
| HP:0001284 | 104 | Areflexia |
| HP:0002460 | 104 | Distal muscle weakness |
| HP:0002936 | 104 | Distal sensory impairment |
| HP:0009027 | 104 | Foot dorsiflexor weakness |
| HP:0003557 | 104 | Increased variability in muscle fiber diameter |
| HP:0002515 | 104 | Waddling gait |
| HP:0002878 | 104 | Respiratory failure |
| HP:0002355 | 104 | Difficulty walking |
| HP:0007269 | 104 | Spinal muscular atrophy |
| HP:0001308 | 104 | Tongue fasciculations |
| HP:0009046 | 104 | Difficulty running |
| HP:0001558 | 104 | Decreased fetal movement |
| HP:0002747 | 104 | Respiratory insufficiency due to muscle weakness |
| HP:0007126 | 104 | Proximal amyotrophy |
| HP:0006829 | 104 | Severe muscular hypotonia |

---


---


---

# Cluster \*106

| HPO | Cluster | Description |
| --- | --- | --- |
| HP:0001324 | 106 | Muscle weakness |
| HP:0002145 | 106 | Frontotemporal dementia |
| HP:0003687 | 106 | Centrally nucleated skeletal muscle fibers |
| HP:0100295 | 106 | Muscle fiber atrophy |

---


---


---

# Cluster \*107

| HPO | Cluster | Description |
| --- | --- | --- |
| HP:0001270 | 107 | Motor delay |
| HP:0000218 | 107 | High palate |
| HP:0001283 | 107 | Bulbar palsy |
| HP:0001265 | 107 | Hyporeflexia |
| HP:0001761 | 107 | Pes cavus |
| HP:0003693 | 107 | Distal amyotrophy |
| HP:0003690 | 107 | Limb muscle weakness |
| HP:0003701 | 107 | Proximal muscle weakness |
| HP:0003307 | 107 | Hyperlordosis |
| HP:0010628 | 107 | Facial palsy |
| HP:0001319 | 107 | Neonatal hypotonia |
| HP:0003324 | 107 | Generalized muscle weakness |
| HP:0003798 | 107 | Nemaline bodies |
| HP:0003803 | 107 | Type 1 muscle fiber predominance |
| HP:0001284 | 107 | Areflexia |
| HP:0001765 | 107 | Hammertoe |
| HP:0002460 | 107 | Distal muscle weakness |
| HP:0003376 | 107 | Steppage gait |
| HP:0003621 | 107 | Juvenile onset |
| HP:0003458 | 107 | EMG: myopathic abnormalities |
| HP:0003200 | 107 | Ragged-red muscle fibers |
| HP:0003557 | 107 | Increased variability in muscle fiber diameter |
| HP:0001533 | 107 | Slender build |
| HP:0002515 | 107 | Waddling gait |
| HP:0003388 | 107 | Easy fatigability |
| HP:0003390 | 107 | Sensory axonal neuropathy |
| HP:0003691 | 107 | Scapular winging |
| HP:0000590 | 107 | Progressive external ophthalmoplegia |
| HP:0003548 | 107 | Subsarcolemmal accumulations of abnormally shaped mitochondria |
| HP:0003688 | 107 | Cytochrome C oxidase-negative muscle fibers |
| HP:0003713 | 107 | Muscle fiber necrosis |
| HP:0006886 | 107 | Impaired distal vibration sensation |
| HP:0002747 | 107 | Respiratory insufficiency due to muscle weakness |
| HP:0003391 | 107 | Gowers sign |
| HP:0000467 | 107 | Neck muscle weakness |
| HP:0008180 | 107 | Mildly elevated creatine kinase |
| HP:0002058 | 107 | Myopathic facies |
| HP:0003306 | 107 | Spinal rigidity |
| HP:0003722 | 107 | Neck flexor weakness |
| HP:0003810 | 107 | Late-onset distal muscle weakness |
| HP:0000597 | 107 | Ophthalmoparesis |
| HP:0003473 | 107 | Fatigable weakness |
| HP:0002403 | 107 | Positive Romberg sign |
| HP:0003434 | 107 | Sensory ataxic neuropathy |

---


---


---

# Cluster \*108

| HPO | Cluster | Description |
| --- | --- | --- |
| HP:0002650 | 108 | Scoliosis |
| HP:0001265 | 108 | Hyporeflexia |
| HP:0003693 | 108 | Distal amyotrophy |
| HP:0010628 | 108 | Facial palsy |
| HP:0001284 | 108 | Areflexia |
| HP:0002936 | 108 | Distal sensory impairment |
| HP:0004336 | 108 | Myelin outfoldings |
| HP:0000762 | 108 | Decreased nerve conduction velocity |
| HP:0003700 | 108 | Generalized amyotrophy |
| HP:0007182 | 108 | Peripheral hypomyelination |
| HP:0007108 | 108 | Demyelinating peripheral neuropathy |

---


---


---

# Cluster \*109

| HPO | Cluster | Description |
| --- | --- | --- |
| HP:0003394 | 109 | Muscle spasm |
| HP:0003201 | 109 | Rhabdomyolysis |
| HP:0003546 | 109 | Exercise intolerance |
| HP:0003552 | 109 | Muscle stiffness |
| HP:0002913 | 109 | Myoglobinuria |
| HP:0002600 | 109 | Hyporeflexia of lower limbs |
| HP:0009046 | 109 | Difficulty running |
| HP:0003738 | 109 | Exercise-induced myalgia |
| HP:0009005 | 109 | Weakness of the intrinsic hand muscles |
| HP:0009063 | 109 | Progressive distal muscle weakness |
| HP:0100303 | 109 | Muscle fiber cytoplasmatic inclusion bodies |

---


---


---

# Cluster \*110

| HPO | Cluster | Description |
| --- | --- | --- |
| HP:0001270 | 110 | Motor delay |
| HP:0010628 | 110 | Facial palsy |
| HP:0001371 | 110 | Flexion contracture |
| HP:0003324 | 110 | Generalized muscle weakness |
| HP:0002359 | 110 | Frequent falls |
| HP:0002355 | 110 | Difficulty walking |
| HP:0002111 | 110 | Restrictive deficit on pulmonary function testing |
| HP:0003547 | 110 | Shoulder girdle muscle weakness |
| HP:0008981 | 110 | Calf muscle hypertrophy |
| HP:0003749 | 110 | Pelvic girdle muscle weakness |
| HP:0007126 | 110 | Proximal amyotrophy |
| HP:0003551 | 110 | Difficulty climbing stairs |
| HP:0001771 | 110 | Achilles tendon contracture |
| HP:0040083 | 110 | Toe walking |
| HP:0003733 | 110 | Thigh hypertrophy |
| HP:0003327 | 110 | Axial muscle weakness |
| HP:0003787 | 110 | Type 1 and type 2 muscle fiber minicore regions |

---


---


---

# Cluster \*111

| HPO | Cluster | Description |
| --- | --- | --- |
| HP:0000007 | 111 | Autosomal recessive inheritance |
| HP:0000508 | 111 | Ptosis |
| HP:0003593 | 111 | Infantile onset |
| HP:0011675 | 111 | Arrhythmia |
| HP:0001561 | 111 | Polyhydramnios |
| HP:0003198 | 111 | Myopathy |
| HP:0001639 | 111 | Hypertrophic cardiomyopathy |
| HP:0001644 | 111 | Dilated cardiomyopathy |
| HP:0003577 | 111 | Congenital onset |
| HP:0003458 | 111 | EMG: myopathic abnormalities |
| HP:0003200 | 111 | Ragged-red muscle fibers |
| HP:0003546 | 111 | Exercise intolerance |
| HP:0002747 | 111 | Respiratory insufficiency due to muscle weakness |
| HP:0002093 | 111 | Respiratory insufficiency |
| HP:0003704 | 111 | Scapuloperoneal weakness |
| HP:0003700 | 111 | Generalized amyotrophy |
| HP:0002878 | 111 | Respiratory failure |

---


---


---

# Cluster \*112

| HPO | Cluster | Description |
| --- | --- | --- |
| HP:0005348 | 112 | Inspiratory stridor |
| HP:0100295 | 112 | Muscle fiber atrophy |
| HP:0009110 | 112 | Diaphragmatic eventration |
| HP:0006597 | 112 | Diaphragmatic paralysis |

---


---


---

# Cluster \*113

| HPO | Cluster | Description |
| --- | --- | --- |
| HP:0001155 | 113 | Abnormality of the hand |
| HP:0100295 | 113 | Muscle fiber atrophy |
| HP:0003447 | 113 | Axonal loss |
| HP:0007149 | 113 | Distal upper limb amyotrophy |

---


---


---

# Cluster \*114

| HPO | Cluster | Description |
| --- | --- | --- |
| HP:0001270 | 114 | Motor delay |
| HP:0003701 | 114 | Proximal muscle weakness |
| HP:0003307 | 114 | Hyperlordosis |
| HP:0010628 | 114 | Facial palsy |
| HP:0001319 | 114 | Neonatal hypotonia |
| HP:0001371 | 114 | Flexion contracture |
| HP:0003324 | 114 | Generalized muscle weakness |
| HP:0003798 | 114 | Nemaline bodies |
| HP:0003803 | 114 | Type 1 muscle fiber predominance |
| HP:0001612 | 114 | Weak cry |
| HP:0003458 | 114 | EMG: myopathic abnormalities |
| HP:0003200 | 114 | Ragged-red muscle fibers |
| HP:0003557 | 114 | Increased variability in muscle fiber diameter |
| HP:0001618 | 114 | Dysphonia |
| HP:0002515 | 114 | Waddling gait |
| HP:0002359 | 114 | Frequent falls |
| HP:0003323 | 114 | Progressive muscle weakness |
| HP:0003688 | 114 | Cytochrome C oxidase-negative muscle fibers |
| HP:0003713 | 114 | Muscle fiber necrosis |
| HP:0002355 | 114 | Difficulty walking |
| HP:0002747 | 114 | Respiratory insufficiency due to muscle weakness |
| HP:0000467 | 114 | Neck muscle weakness |
| HP:0003554 | 114 | Type 2 muscle fiber atrophy |
| HP:0002058 | 114 | Myopathic facies |
| HP:0003306 | 114 | Spinal rigidity |
| HP:0003722 | 114 | Neck flexor weakness |
| HP:0003810 | 114 | Late-onset distal muscle weakness |
| HP:0002033 | 114 | Poor suck |
| HP:0002715 | 114 | Abnormality of the immune system |
| HP:0003741 | 114 | Congenital muscular dystrophy |
| HP:0000597 | 114 | Ophthalmoparesis |
| HP:0002872 | 114 | Apneic episodes precipitated by illness, fatigue, stress |
| HP:0002882 | 114 | Sudden episodic apnea |
| HP:0003397 | 114 | Generalized hypotonia due to defect at the neuromuscular junction |
| HP:0003402 | 114 | Decreased miniature endplate potentials |
| HP:0003403 | 114 | EMG: decremental response of compound muscle action potential to repetitive nerve stimulation |
| HP:0003473 | 114 | Fatigable weakness |
| HP:0003443 | 114 | Decreased size of nerve terminals |

---


---


---

# Cluster \*116

| HPO | Cluster | Description |
| --- | --- | --- |
| HP:0001270 | 116 | Motor delay |
| HP:0000508 | 116 | Ptosis |
| HP:0000218 | 116 | High palate |
| HP:0002650 | 116 | Scoliosis |
| HP:0001283 | 116 | Bulbar palsy |
| HP:0001265 | 116 | Hyporeflexia |
| HP:0003477 | 116 | Peripheral axonal neuropathy |
| HP:0002804 | 116 | Arthrogryposis multiplex congenita |
| HP:0001561 | 116 | Polyhydramnios |
| HP:0003690 | 116 | Limb muscle weakness |
| HP:0003701 | 116 | Proximal muscle weakness |
| HP:0003307 | 116 | Hyperlordosis |
| HP:0010628 | 116 | Facial palsy |
| HP:0001319 | 116 | Neonatal hypotonia |
| HP:0001371 | 116 | Flexion contracture |
| HP:0003324 | 116 | Generalized muscle weakness |
| HP:0003798 | 116 | Nemaline bodies |
| HP:0001284 | 116 | Areflexia |
| HP:0001612 | 116 | Weak cry |
| HP:0003458 | 116 | EMG: myopathic abnormalities |
| HP:0003737 | 116 | Mitochondrial myopathy |
| HP:0001427 | 116 | Mitochondrial inheritance |
| HP:0003200 | 116 | Ragged-red muscle fibers |
| HP:0003557 | 116 | Increased variability in muscle fiber diameter |
| HP:0001618 | 116 | Dysphonia |
| HP:0003388 | 116 | Easy fatigability |
| HP:0000590 | 116 | Progressive external ophthalmoplegia |
| HP:0003323 | 116 | Progressive muscle weakness |
| HP:0003548 | 116 | Subsarcolemmal accumulations of abnormally shaped mitochondria |
| HP:0003688 | 116 | Cytochrome C oxidase-negative muscle fibers |
| HP:0003713 | 116 | Muscle fiber necrosis |
| HP:0006886 | 116 | Impaired distal vibration sensation |
| HP:0002747 | 116 | Respiratory insufficiency due to muscle weakness |
| HP:0000467 | 116 | Neck muscle weakness |
| HP:0003554 | 116 | Type 2 muscle fiber atrophy |
| HP:0002058 | 116 | Myopathic facies |
| HP:0002033 | 116 | Poor suck |
| HP:0002715 | 116 | Abnormality of the immune system |
| HP:0003700 | 116 | Generalized amyotrophy |
| HP:0002490 | 116 | Increased CSF lactate |
| HP:0000597 | 116 | Ophthalmoparesis |
| HP:0002872 | 116 | Apneic episodes precipitated by illness, fatigue, stress |
| HP:0002882 | 116 | Sudden episodic apnea |
| HP:0003397 | 116 | Generalized hypotonia due to defect at the neuromuscular junction |
| HP:0003402 | 116 | Decreased miniature endplate potentials |
| HP:0003403 | 116 | EMG: decremental response of compound muscle action potential to repetitive nerve stimulation |
| HP:0003473 | 116 | Fatigable weakness |
| HP:0003434 | 116 | Sensory ataxic neuropathy |
| HP:0002579 | 116 | Gastrointestinal dysmotility |
| HP:0004326 | 116 | Cachexia |
| HP:0003436 | 116 | Prolonged miniature endplate currents |
| HP:0003443 | 116 | Decreased size of nerve terminals |

---


---


---

# Cluster \*117

| HPO | Cluster | Description |
| --- | --- | --- |
| HP:0001302 | 117 | Pachygyria |
| HP:0001270 | 117 | Motor delay |
| HP:0000557 | 117 | Buphthalmos |
| HP:0003828 | 117 | Variable expressivity |
| HP:0000518 | 117 | Cataract |
| HP:0003581 | 117 | Adult onset |
| HP:0001265 | 117 | Hyporeflexia |
| HP:0001324 | 117 | Muscle weakness |
| HP:0003677 | 117 | Slow progression |
| HP:0003325 | 117 | Limb-girdle muscle weakness |
| HP:0003701 | 117 | Proximal muscle weakness |
| HP:0003307 | 117 | Hyperlordosis |
| HP:0010628 | 117 | Facial palsy |
| HP:0003577 | 117 | Congenital onset |
| HP:0001371 | 117 | Flexion contracture |
| HP:0003324 | 117 | Generalized muscle weakness |
| HP:0003326 | 117 | Myalgia |
| HP:0003458 | 117 | EMG: myopathic abnormalities |
| HP:0003560 | 117 | Muscular dystrophy |
| HP:0003557 | 117 | Increased variability in muscle fiber diameter |
| HP:0001321 | 117 | Cerebellar hypoplasia |
| HP:0002515 | 117 | Waddling gait |
| HP:0003691 | 117 | Scapular winging |
| HP:0002355 | 117 | Difficulty walking |
| HP:0009046 | 117 | Difficulty running |
| HP:0002747 | 117 | Respiratory insufficiency due to muscle weakness |
| HP:0003547 | 117 | Shoulder girdle muscle weakness |
| HP:0003724 | 117 | Shoulder girdle muscle atrophy |
| HP:0008981 | 117 | Calf muscle hypertrophy |
| HP:0003749 | 117 | Pelvic girdle muscle weakness |
| HP:0003391 | 117 | Gowers sign |
| HP:0003687 | 117 | Centrally nucleated skeletal muscle fibers |
| HP:0000467 | 117 | Neck muscle weakness |
| HP:0003805 | 117 | Rimmed vacuoles |
| HP:0002093 | 117 | Respiratory insufficiency |
| HP:0007126 | 117 | Proximal amyotrophy |
| HP:0003306 | 117 | Spinal rigidity |
| HP:0003736 | 117 | Autophagic vacuoles |
| HP:0003551 | 117 | Difficulty climbing stairs |
| HP:0008988 | 117 | Pelvic girdle muscle atrophy |
| HP:0003555 | 117 | Muscle fiber splitting |
| HP:0002365 | 117 | Hypoplasia of the brainstem |
| HP:0007973 | 117 | Retinal dysplasia |
| HP:0012110 | 117 | Hypoplasia of the pons |
| HP:0009025 | 117 | Increased connective tissue |
| HP:0006829 | 117 | Severe muscular hypotonia |
| HP:0003741 | 117 | Congenital muscular dystrophy |
| HP:0007033 | 117 | Cerebellar dysplasia |
| HP:0007260 | 117 | Type II lissencephaly |
| HP:0031882 | 117 | Agyria |
| HP:0002350 | 117 | Cerebellar cyst |
| HP:0100614 | 117 | Myositis |
| HP:0003707 | 117 | Calf muscle pseudohypertrophy |
| HP:0009072 | 117 | Decreased Achilles reflex |
| HP:0003327 | 117 | Axial muscle weakness |
| HP:0003787 | 117 | Type 1 and type 2 muscle fiber minicore regions |
| HP:0100297 | 117 | Increased endomysial connective tissue |
| HP:0030046 | 117 | Hypoglycosylation of alpha-dystroglycan |

---


---


---

# Cluster \*118

| HPO | Cluster | Description |
| --- | --- | --- |
| HP:0001762 | 118 | Talipes equinovarus |
| HP:0001265 | 118 | Hyporeflexia |
| HP:0001611 | 118 | Nasal speech |
| HP:0010628 | 118 | Facial palsy |
| HP:0001284 | 118 | Areflexia |
| HP:0003236 | 118 | Elevated serum creatine kinase |
| HP:0001558 | 118 | Decreased fetal movement |
| HP:0002093 | 118 | Respiratory insufficiency |
| HP:0000764 | 118 | Peripheral axonal degeneration |
| HP:0006597 | 118 | Diaphragmatic paralysis |

---


---


---

# Cluster \*119

| HPO | Cluster | Description |
| --- | --- | --- |
| HP:0001604 | 119 | Vocal cord paresis |
| HP:0009113 | 119 | Diaphragmatic weakness |
| HP:0010307 | 119 | Stridor |
| HP:0011349 | 119 | Abducens palsy |
| HP:0003444 | 119 | EMG: chronic denervation signs |
| HP:0009130 | 119 | Hand muscle atrophy |
| HP:0012246 | 119 | Oculomotor nerve palsy |
| HP:0007230 | 119 | Decreased distal sensory nerve action potential |

---


---


---

# Cluster \*120

| HPO | Cluster | Description |
| --- | --- | --- |
| HP:0002021 | 120 | Pyloric stenosis |
| HP:0002033 | 120 | Poor suck |
| HP:0000508 | 120 | Ptosis |
| HP:0000218 | 120 | High palate |
| HP:0001611 | 120 | Nasal speech |
| HP:0009588 | 120 | Vestibular Schwannoma |

---


---


---

# Cluster \*121

| HPO | Cluster | Description |
| --- | --- | --- |
| HP:0009830 | 121 | Peripheral neuropathy |
| HP:0003674 | 121 | Onset |
| HP:0002515 | 121 | Waddling gait |
| HP:0001425 | 121 | Heterogeneous |
| HP:0001265 | 121 | Hyporeflexia |
| HP:0001761 | 121 | Pes cavus |
| HP:0003677 | 121 | Slow progression |
| HP:0003693 | 121 | Distal amyotrophy |
| HP:0003701 | 121 | Proximal muscle weakness |
| HP:0003324 | 121 | Generalized muscle weakness |
| HP:0001284 | 121 | Areflexia |
| HP:0001765 | 121 | Hammertoe |
| HP:0002460 | 121 | Distal muscle weakness |
| HP:0002936 | 121 | Distal sensory impairment |
| HP:0003380 | 121 | Decreased number of peripheral myelinated nerve fibers |
| HP:0003383 | 121 | Onion bulb formation |
| HP:0009027 | 121 | Foot dorsiflexor weakness |
| HP:0003378 | 121 | Axonal degeneration/regeneration |
| HP:0003384 | 121 | Peripheral axonal atrophy |
| HP:0003458 | 121 | EMG: myopathic abnormalities |
| HP:0001604 | 121 | Vocal cord paresis |
| HP:0003687 | 121 | Centrally nucleated skeletal muscle fibers |
| HP:0003484 | 121 | Upper limb muscle weakness |

---


---


---

# Cluster \*122

| HPO | Cluster | Description |
| --- | --- | --- |
| HP:0002366 | 122 | Abnormal lower motor neuron morphology |
| HP:0002460 | 122 | Distal muscle weakness |
| HP:0001761 | 122 | Pes cavus |
| HP:0003677 | 122 | Slow progression |
| HP:0003693 | 122 | Distal amyotrophy |
| HP:0008756 | 122 | Bowing of the vocal cords |
| HP:0002483 | 122 | Bulbar signs |

---


---


---

# Cluster \*123

| HPO | Cluster | Description |
| --- | --- | --- |
| HP:0001302 | 123 | Pachygyria |
| HP:0001270 | 123 | Motor delay |
| HP:0003593 | 123 | Infantile onset |
| HP:0008872 | 123 | Feeding difficulties in infancy |
| HP:0003701 | 123 | Proximal muscle weakness |
| HP:0003307 | 123 | Hyperlordosis |
| HP:0010628 | 123 | Facial palsy |
| HP:0001319 | 123 | Neonatal hypotonia |
| HP:0001371 | 123 | Flexion contracture |
| HP:0003324 | 123 | Generalized muscle weakness |
| HP:0003326 | 123 | Myalgia |
| HP:0003458 | 123 | EMG: myopathic abnormalities |
| HP:0003557 | 123 | Increased variability in muscle fiber diameter |
| HP:0001321 | 123 | Cerebellar hypoplasia |
| HP:0002111 | 123 | Restrictive deficit on pulmonary function testing |
| HP:0003547 | 123 | Shoulder girdle muscle weakness |
| HP:0008981 | 123 | Calf muscle hypertrophy |
| HP:0002093 | 123 | Respiratory insufficiency |
| HP:0003306 | 123 | Spinal rigidity |
| HP:0003551 | 123 | Difficulty climbing stairs |
| HP:0001771 | 123 | Achilles tendon contracture |
| HP:0002365 | 123 | Hypoplasia of the brainstem |
| HP:0007973 | 123 | Retinal dysplasia |
| HP:0003741 | 123 | Congenital muscular dystrophy |
| HP:0007033 | 123 | Cerebellar dysplasia |
| HP:0007260 | 123 | Type II lissencephaly |
| HP:0031882 | 123 | Agyria |
| HP:0002350 | 123 | Cerebellar cyst |
| HP:0003733 | 123 | Thigh hypertrophy |

---


---


---

# Cluster \*124

| HPO | Cluster | Description |
| --- | --- | --- |
| HP:0003676 | 124 | Progressive |
| HP:0005010 | 124 | Osteomyelitis leading to amputation due to slow healing fractures |
| HP:0006957 | 124 | Loss of ability to walk |
| HP:0003380 | 124 | Decreased number of peripheral myelinated nerve fibers |
| HP:0007141 | 124 | Sensorimotor neuropathy |

---


---


---

# Cluster \*126

| HPO | Cluster | Description |
| --- | --- | --- |
| HP:0004336 | 126 | Myelin outfoldings |
| HP:0011096 | 126 | Peripheral demyelination |
| HP:0000522 | 126 | Alacrima |
| HP:0007182 | 126 | Peripheral hypomyelination |
| HP:0007108 | 126 | Demyelinating peripheral neuropathy |
| HP:0004463 | 126 | Absent brainstem auditory responses |

---


---


---

# Cluster \*127

| HPO | Cluster | Description |
| --- | --- | --- |
| HP:0000218 | 127 | High palate |
| HP:0002650 | 127 | Scoliosis |
| HP:0001283 | 127 | Bulbar palsy |
| HP:0003701 | 127 | Proximal muscle weakness |
| HP:0003307 | 127 | Hyperlordosis |
| HP:0010628 | 127 | Facial palsy |
| HP:0001319 | 127 | Neonatal hypotonia |
| HP:0003324 | 127 | Generalized muscle weakness |
| HP:0003798 | 127 | Nemaline bodies |
| HP:0003803 | 127 | Type 1 muscle fiber predominance |
| HP:0003458 | 127 | EMG: myopathic abnormalities |
| HP:0003737 | 127 | Mitochondrial myopathy |
| HP:0003546 | 127 | Exercise intolerance |
| HP:0001533 | 127 | Slender build |
| HP:0002515 | 127 | Waddling gait |
| HP:0004395 | 127 | Malnutrition |
| HP:0000590 | 127 | Progressive external ophthalmoplegia |
| HP:0002747 | 127 | Respiratory insufficiency due to muscle weakness |
| HP:0002058 | 127 | Myopathic facies |
| HP:0003306 | 127 | Spinal rigidity |
| HP:0003722 | 127 | Neck flexor weakness |
| HP:0003810 | 127 | Late-onset distal muscle weakness |
| HP:0003700 | 127 | Generalized amyotrophy |
| HP:0003434 | 127 | Sensory ataxic neuropathy |
| HP:0002579 | 127 | Gastrointestinal dysmotility |
| HP:0007103 | 127 | Hypointensity of cerebral white matter on MRI |
| HP:0004326 | 127 | Cachexia |

---


---


---

# Cluster \*129

| HPO | Cluster | Description |
| --- | --- | --- |
| HP:0000975 | 129 | Hyperhidrosis |
| HP:0001649 | 129 | Tachycardia |
| HP:0001962 | 129 | Palpitations |
| HP:0000522 | 129 | Alacrima |
| HP:0001605 | 129 | Vocal cord paralysis |
| HP:0003768 | 129 | Periodic paralysis |

---


---


---

# Cluster \*130

| HPO | Cluster | Description |
| --- | --- | --- |
| HP:0003581 | 130 | Adult onset |
| HP:0003677 | 130 | Slow progression |
| HP:0003325 | 130 | Limb-girdle muscle weakness |
| HP:0002460 | 130 | Distal muscle weakness |
| HP:0007340 | 130 | Lower limb muscle weakness |
| HP:0009005 | 130 | Weakness of the intrinsic hand muscles |
| HP:0003736 | 130 | Autophagic vacuoles |
| HP:0003555 | 130 | Muscle fiber splitting |
| HP:0003444 | 130 | EMG: chronic denervation signs |
| HP:0003791 | 130 | Deposits immunoreactive to beta-amyloid protein |
| HP:0009072 | 130 | Decreased Achilles reflex |

---


---


---

# Cluster \*132

| HPO | Cluster | Description |
| --- | --- | --- |
| HP:0002398 | 132 | Degeneration of anterior horn cells |
| HP:0003677 | 132 | Slow progression |
| HP:0003701 | 132 | Proximal muscle weakness |
| HP:0010628 | 132 | Facial palsy |
| HP:0002515 | 132 | Waddling gait |
| HP:0003388 | 132 | Easy fatigability |
| HP:0003691 | 132 | Scapular winging |
| HP:0002359 | 132 | Frequent falls |
| HP:0002355 | 132 | Difficulty walking |
| HP:0008981 | 132 | Calf muscle hypertrophy |
| HP:0001308 | 132 | Tongue fasciculations |
| HP:0003391 | 132 | Gowers sign |
| HP:0000467 | 132 | Neck muscle weakness |
| HP:0008180 | 132 | Mildly elevated creatine kinase |
| HP:0007126 | 132 | Proximal amyotrophy |
| HP:0003555 | 132 | Muscle fiber splitting |
| HP:0002378 | 132 | Hand tremor |
| HP:0000597 | 132 | Ophthalmoparesis |
| HP:0003473 | 132 | Fatigable weakness |

---


---


---

# Cluster \*133

| HPO | Cluster | Description |
| --- | --- | --- |
| HP:0003724 | 133 | Shoulder girdle muscle atrophy |
| HP:0003749 | 133 | Pelvic girdle muscle weakness |
| HP:0008955 | 133 | Progressive distal muscular atrophy |
| HP:0003722 | 133 | Neck flexor weakness |
| HP:0003555 | 133 | Muscle fiber splitting |
| HP:0009113 | 133 | Diaphragmatic weakness |
| HP:0009130 | 133 | Hand muscle atrophy |
| HP:0003697 | 133 | Scapuloperoneal amyotrophy |
| HP:0011349 | 133 | Abducens palsy |
| HP:0002877 | 133 | Nocturnal hypoventilation |
| HP:0003731 | 133 | Quadriceps muscle weakness |
| HP:0003715 | 133 | Myofibrillar myopathy |
| HP:0002792 | 133 | Reduced vital capacity |

---


---


---

# Cluster \*134

| HPO | Cluster | Description |
| --- | --- | --- |
| HP:0003418 | 134 | Back pain |
| HP:0011675 | 134 | Arrhythmia |
| HP:0001288 | 134 | Gait disturbance |
| HP:0003198 | 134 | Myopathy |
| HP:0003701 | 134 | Proximal muscle weakness |
| HP:0003236 | 134 | Elevated serum creatine kinase |
| HP:0003691 | 134 | Scapular winging |
| HP:0003805 | 134 | Rimmed vacuoles |
| HP:0008988 | 134 | Pelvic girdle muscle atrophy |
| HP:0003704 | 134 | Scapuloperoneal weakness |

---


---


---

# Cluster \*135

| HPO | Cluster | Description |
| --- | --- | --- |
| HP:0001324 | 135 | Muscle weakness |
| HP:0002486 | 135 | Myotonia |
| HP:0002047 | 135 | Malignant hyperthermia |
| HP:0006958 | 135 | Abnormal auditory evoked potentials |
| HP:0003044 | 135 | Shoulder flexion contracture |

---


---


---

# Cluster \*136

| HPO | Cluster | Description |
| --- | --- | --- |
| HP:0003198 | 136 | Myopathy |
| HP:0003701 | 136 | Proximal muscle weakness |
| HP:0003805 | 136 | Rimmed vacuoles |
| HP:0006785 | 136 | Limb-girdle muscular dystrophy |
| HP:0002803 | 136 | Congenital contracture |
| HP:0100299 | 136 | Muscle fiber inclusion bodies |

---


---


---

# Cluster \*137

| HPO | Cluster | Description |
| --- | --- | --- |
| HP:0003828 | 137 | Variable expressivity |
| HP:0001283 | 137 | Bulbar palsy |
| HP:0001260 | 137 | Dysarthria |
| HP:0002936 | 137 | Distal sensory impairment |
| HP:0000762 | 137 | Decreased nerve conduction velocity |
| HP:0000590 | 137 | Progressive external ophthalmoplegia |
| HP:0002500 | 137 | Abnormality of the cerebral white matter |
| HP:0001771 | 137 | Achilles tendon contracture |
| HP:0040083 | 137 | Toe walking |

---


---


---

# Cluster \*139

| HPO | Cluster | Description |
| --- | --- | --- |
| HP:0001638 | 139 | Cardiomyopathy |
| HP:0001265 | 139 | Hyporeflexia |
| HP:0001761 | 139 | Pes cavus |
| HP:0003677 | 139 | Slow progression |
| HP:0001284 | 139 | Areflexia |
| HP:0002460 | 139 | Distal muscle weakness |
| HP:0002936 | 139 | Distal sensory impairment |
| HP:0003376 | 139 | Steppage gait |
| HP:0003390 | 139 | Sensory axonal neuropathy |
| HP:0003200 | 139 | Ragged-red muscle fibers |
| HP:0003546 | 139 | Exercise intolerance |
| HP:0001618 | 139 | Dysphonia |
| HP:0000590 | 139 | Progressive external ophthalmoplegia |
| HP:0003323 | 139 | Progressive muscle weakness |
| HP:0003548 | 139 | Subsarcolemmal accumulations of abnormally shaped mitochondria |
| HP:0003688 | 139 | Cytochrome C oxidase-negative muscle fibers |
| HP:0003713 | 139 | Muscle fiber necrosis |
| HP:0002403 | 139 | Positive Romberg sign |
| HP:0003434 | 139 | Sensory ataxic neuropathy |
| HP:0003484 | 139 | Upper limb muscle weakness |

---


---


---

# Cluster \*140

| HPO | Cluster | Description |
| --- | --- | --- |
| HP:0000007 | 140 | Autosomal recessive inheritance |
| HP:0003202 | 140 | Skeletal muscle atrophy |
| HP:0003593 | 140 | Infantile onset |
| HP:0003676 | 140 | Progressive |
| HP:0001639 | 140 | Hypertrophic cardiomyopathy |
| HP:0003577 | 140 | Congenital onset |
| HP:0003128 | 140 | Lactic acidosis |
| HP:0002878 | 140 | Respiratory failure |
| HP:0011968 | 140 | Feeding difficulties |
| HP:0002033 | 140 | Poor suck |
| HP:0002490 | 140 | Increased CSF lactate |
| HP:0008972 | 140 | Decreased activity of mitochondrial respiratory chain |

---


---


---

# Cluster \*141

| HPO | Cluster | Description |
| --- | --- | --- |
| HP:0000007 | 141 | Autosomal recessive inheritance |
| HP:0001302 | 141 | Pachygyria |
| HP:0001270 | 141 | Motor delay |
| HP:0000518 | 141 | Cataract |
| HP:0003676 | 141 | Progressive |
| HP:0001260 | 141 | Dysarthria |
| HP:0002804 | 141 | Arthrogryposis multiplex congenita |
| HP:0001561 | 141 | Polyhydramnios |
| HP:0001319 | 141 | Neonatal hypotonia |
| HP:0001371 | 141 | Flexion contracture |
| HP:0003324 | 141 | Generalized muscle weakness |
| HP:0003236 | 141 | Elevated serum creatine kinase |
| HP:0003560 | 141 | Muscular dystrophy |
| HP:0003546 | 141 | Exercise intolerance |
| HP:0011968 | 141 | Feeding difficulties |
| HP:0003391 | 141 | Gowers sign |
| HP:0002093 | 141 | Respiratory insufficiency |
| HP:0002365 | 141 | Hypoplasia of the brainstem |
| HP:0002803 | 141 | Congenital contracture |
| HP:0012110 | 141 | Hypoplasia of the pons |
| HP:0003741 | 141 | Congenital muscular dystrophy |
| HP:0007033 | 141 | Cerebellar dysplasia |
| HP:0007260 | 141 | Type II lissencephaly |
| HP:0002350 | 141 | Cerebellar cyst |
| HP:0000597 | 141 | Ophthalmoparesis |
| HP:0003473 | 141 | Fatigable weakness |

---


---


---

# Cluster \*142

| HPO | Cluster | Description |
| --- | --- | --- |
| HP:0001270 | 142 | Motor delay |
| HP:0002650 | 142 | Scoliosis |
| HP:0003198 | 142 | Myopathy |
| HP:0008994 | 142 | Proximal muscle weakness in lower limbs |
| HP:0007941 | 142 | Limited extraocular movements |

---


---


---

# Cluster \*143

| HPO | Cluster | Description |
| --- | --- | --- |
| HP:0000007 | 143 | Autosomal recessive inheritance |
| HP:0003828 | 143 | Variable expressivity |
| HP:0000218 | 143 | High palate |
| HP:0001288 | 143 | Gait disturbance |
| HP:0001761 | 143 | Pes cavus |
| HP:0003677 | 143 | Slow progression |
| HP:0003325 | 143 | Limb-girdle muscle weakness |
| HP:0001284 | 143 | Areflexia |
| HP:0002936 | 143 | Distal sensory impairment |
| HP:0003560 | 143 | Muscular dystrophy |
| HP:0003691 | 143 | Scapular winging |
| HP:0006466 | 143 | Ankle flexion contracture |

---


---


---

# Cluster \*144

| HPO | Cluster | Description |
| --- | --- | --- |
| HP:0001283 | 144 | Bulbar palsy |
| HP:0001265 | 144 | Hyporeflexia |
| HP:0003394 | 144 | Muscle spasm |
| HP:0000158 | 144 | Macroglossia |
| HP:0001761 | 144 | Pes cavus |
| HP:0003677 | 144 | Slow progression |
| HP:0003701 | 144 | Proximal muscle weakness |
| HP:0003307 | 144 | Hyperlordosis |
| HP:0010628 | 144 | Facial palsy |
| HP:0003324 | 144 | Generalized muscle weakness |
| HP:0001284 | 144 | Areflexia |
| HP:0002936 | 144 | Distal sensory impairment |
| HP:0009027 | 144 | Foot dorsiflexor weakness |
| HP:0003326 | 144 | Myalgia |
| HP:0003737 | 144 | Mitochondrial myopathy |
| HP:0003201 | 144 | Rhabdomyolysis |
| HP:0003546 | 144 | Exercise intolerance |
| HP:0002515 | 144 | Waddling gait |
| HP:0003388 | 144 | Easy fatigability |
| HP:0002913 | 144 | Myoglobinuria |
| HP:0002359 | 144 | Frequent falls |
| HP:0002355 | 144 | Difficulty walking |
| HP:0009046 | 144 | Difficulty running |
| HP:0002111 | 144 | Restrictive deficit on pulmonary function testing |
| HP:0003547 | 144 | Shoulder girdle muscle weakness |
| HP:0008981 | 144 | Calf muscle hypertrophy |
| HP:0003391 | 144 | Gowers sign |
| HP:0008180 | 144 | Mildly elevated creatine kinase |
| HP:0003554 | 144 | Type 2 muscle fiber atrophy |
| HP:0007126 | 144 | Proximal amyotrophy |
| HP:0003551 | 144 | Difficulty climbing stairs |
| HP:0002715 | 144 | Abnormality of the immune system |
| HP:0001771 | 144 | Achilles tendon contracture |
| HP:0003741 | 144 | Congenital muscular dystrophy |
| HP:0040083 | 144 | Toe walking |
| HP:0000597 | 144 | Ophthalmoparesis |
| HP:0003403 | 144 | EMG: decremental response of compound muscle action potential to repetitive nerve stimulation |
| HP:0003473 | 144 | Fatigable weakness |
| HP:0003733 | 144 | Thigh hypertrophy |

---


---


---

# Cluster \*145

| HPO | Cluster | Description |
| --- | --- | --- |
| HP:0003445 | 145 | EMG: neuropathic changes |
| HP:0007269 | 145 | Spinal muscular atrophy |
| HP:0008994 | 145 | Proximal muscle weakness in lower limbs |
| HP:0009046 | 145 | Difficulty running |
| HP:0011808 | 145 | Decreased patellar reflex |
| HP:0008956 | 145 | Proximal lower limb amyotrophy |
| HP:0002093 | 145 | Respiratory insufficiency |
| HP:0003551 | 145 | Difficulty climbing stairs |

---


---


---

# Cluster \*147

| HPO | Cluster | Description |
| --- | --- | --- |
| HP:0001270 | 147 | Motor delay |
| HP:0009830 | 147 | Peripheral neuropathy |
| HP:0003828 | 147 | Variable expressivity |
| HP:0002359 | 147 | Frequent falls |
| HP:0003593 | 147 | Infantile onset |
| HP:0001283 | 147 | Bulbar palsy |
| HP:0001265 | 147 | Hyporeflexia |
| HP:0002804 | 147 | Arthrogryposis multiplex congenita |
| HP:0001761 | 147 | Pes cavus |
| HP:0003693 | 147 | Distal amyotrophy |
| HP:0003701 | 147 | Proximal muscle weakness |
| HP:0010628 | 147 | Facial palsy |
| HP:0003577 | 147 | Congenital onset |
| HP:0001371 | 147 | Flexion contracture |
| HP:0001178 | 147 | Ulnar claw |
| HP:0001284 | 147 | Areflexia |
| HP:0001765 | 147 | Hammertoe |
| HP:0002460 | 147 | Distal muscle weakness |
| HP:0002936 | 147 | Distal sensory impairment |
| HP:0003376 | 147 | Steppage gait |
| HP:0003382 | 147 | Hypertrophic nerve changes |
| HP:0003383 | 147 | Onion bulb formation |
| HP:0003431 | 147 | Decreased motor nerve conduction velocity |
| HP:0003449 | 147 | Cold-induced muscle cramps |
| HP:0004336 | 147 | Myelin outfoldings |
| HP:0009027 | 147 | Foot dorsiflexor weakness |
| HP:0003481 | 147 | Segmental peripheral demyelination/remyelination |
| HP:0002355 | 147 | Difficulty walking |
| HP:0001558 | 147 | Decreased fetal movement |
| HP:0002747 | 147 | Respiratory insufficiency due to muscle weakness |
| HP:0003547 | 147 | Shoulder girdle muscle weakness |
| HP:0008981 | 147 | Calf muscle hypertrophy |
| HP:0002093 | 147 | Respiratory insufficiency |
| HP:0003551 | 147 | Difficulty climbing stairs |
| HP:0001771 | 147 | Achilles tendon contracture |
| HP:0003741 | 147 | Congenital muscular dystrophy |
| HP:0003733 | 147 | Thigh hypertrophy |

---


---


---

# Cluster \*148

| HPO | Cluster | Description |
| --- | --- | --- |
| HP:0003202 | 148 | Skeletal muscle atrophy |
| HP:0001265 | 148 | Hyporeflexia |
| HP:0003477 | 148 | Peripheral axonal neuropathy |
| HP:0008872 | 148 | Feeding difficulties in infancy |
| HP:0000602 | 148 | Ophthalmoplegia |
| HP:0003693 | 148 | Distal amyotrophy |
| HP:0002460 | 148 | Distal muscle weakness |
| HP:0002936 | 148 | Distal sensory impairment |
| HP:0003431 | 148 | Decreased motor nerve conduction velocity |
| HP:0001349 | 148 | Facial diplegia |
| HP:0002515 | 148 | Waddling gait |
| HP:0001760 | 148 | Abnormality of the foot |
| HP:0003445 | 148 | EMG: neuropathic changes |
| HP:0002093 | 148 | Respiratory insufficiency |

---


---


---

# Cluster \*149

| HPO | Cluster | Description |
| --- | --- | --- |
| HP:0001762 | 149 | Talipes equinovarus |
| HP:0001252 | 149 | Muscular hypotonia |
| HP:0001270 | 149 | Motor delay |
| HP:0003828 | 149 | Variable expressivity |
| HP:0000218 | 149 | High palate |
| HP:0002650 | 149 | Scoliosis |
| HP:0000158 | 149 | Macroglossia |
| HP:0000518 | 149 | Cataract |
| HP:0001265 | 149 | Hyporeflexia |
| HP:0003477 | 149 | Peripheral axonal neuropathy |
| HP:0002804 | 149 | Arthrogryposis multiplex congenita |
| HP:0001761 | 149 | Pes cavus |
| HP:0001561 | 149 | Polyhydramnios |
| HP:0003693 | 149 | Distal amyotrophy |
| HP:0003198 | 149 | Myopathy |
| HP:0003690 | 149 | Limb muscle weakness |
| HP:0003701 | 149 | Proximal muscle weakness |
| HP:0003307 | 149 | Hyperlordosis |
| HP:0010628 | 149 | Facial palsy |
| HP:0003577 | 149 | Congenital onset |
| HP:0001319 | 149 | Neonatal hypotonia |
| HP:0001371 | 149 | Flexion contracture |
| HP:0003324 | 149 | Generalized muscle weakness |
| HP:0003798 | 149 | Nemaline bodies |
| HP:0003803 | 149 | Type 1 muscle fiber predominance |
| HP:0001284 | 149 | Areflexia |
| HP:0002460 | 149 | Distal muscle weakness |
| HP:0002936 | 149 | Distal sensory impairment |
| HP:0003431 | 149 | Decreased motor nerve conduction velocity |
| HP:0009027 | 149 | Foot dorsiflexor weakness |
| HP:0003458 | 149 | EMG: myopathic abnormalities |
| HP:0003557 | 149 | Increased variability in muscle fiber diameter |
| HP:0002515 | 149 | Waddling gait |
| HP:0007340 | 149 | Lower limb muscle weakness |
| HP:0003691 | 149 | Scapular winging |
| HP:0002359 | 149 | Frequent falls |
| HP:0001604 | 149 | Vocal cord paresis |
| HP:0002355 | 149 | Difficulty walking |
| HP:0002747 | 149 | Respiratory insufficiency due to muscle weakness |
| HP:0002111 | 149 | Restrictive deficit on pulmonary function testing |
| HP:0008981 | 149 | Calf muscle hypertrophy |
| HP:0003391 | 149 | Gowers sign |
| HP:0003687 | 149 | Centrally nucleated skeletal muscle fibers |
| HP:0000467 | 149 | Neck muscle weakness |
| HP:0002058 | 149 | Myopathic facies |
| HP:0003306 | 149 | Spinal rigidity |
| HP:0003387 | 149 | Decreased number of large peripheral myelinated nerve fibers |
| HP:0003448 | 149 | Decreased sensory nerve conduction velocity |
| HP:0002803 | 149 | Congenital contracture |
| HP:0002877 | 149 | Nocturnal hypoventilation |
| HP:0006829 | 149 | Severe muscular hypotonia |
| HP:0003741 | 149 | Congenital muscular dystrophy |
| HP:0002350 | 149 | Cerebellar cyst |
| HP:0032341 | 149 | Reduced forced vital capacity |
| HP:0003733 | 149 | Thigh hypertrophy |

---


---


---

# Cluster \*150

| HPO | Cluster | Description |
| --- | --- | --- |
| HP:0000007 | 150 | Autosomal recessive inheritance |
| HP:0009830 | 150 | Peripheral neuropathy |
| HP:0003828 | 150 | Variable expressivity |
| HP:0003593 | 150 | Infantile onset |
| HP:0003676 | 150 | Progressive |
| HP:0001324 | 150 | Muscle weakness |
| HP:0000602 | 150 | Ophthalmoplegia |
| HP:0003198 | 150 | Myopathy |
| HP:0001639 | 150 | Hypertrophic cardiomyopathy |
| HP:0003577 | 150 | Congenital onset |
| HP:0001319 | 150 | Neonatal hypotonia |
| HP:0003737 | 150 | Mitochondrial myopathy |
| HP:0001414 | 150 | Microvesicular hepatic steatosis |
| HP:0001427 | 150 | Mitochondrial inheritance |
| HP:0003128 | 150 | Lactic acidosis |
| HP:0003200 | 150 | Ragged-red muscle fibers |
| HP:0003201 | 150 | Rhabdomyolysis |
| HP:0003546 | 150 | Exercise intolerance |
| HP:0002878 | 150 | Respiratory failure |
| HP:0011968 | 150 | Feeding difficulties |
| HP:0002913 | 150 | Myoglobinuria |
| HP:0003323 | 150 | Progressive muscle weakness |
| HP:0003548 | 150 | Subsarcolemmal accumulations of abnormally shaped mitochondria |
| HP:0003688 | 150 | Cytochrome C oxidase-negative muscle fibers |
| HP:0002747 | 150 | Respiratory insufficiency due to muscle weakness |
| HP:0002093 | 150 | Respiratory insufficiency |
| HP:0001924 | 150 | Sideroblastic anemia |
| HP:0008972 | 150 | Decreased activity of mitochondrial respiratory chain |
| HP:0008322 | 150 | Abnormal mitochondrial morphology |
| HP:0002134 | 150 | Abnormality of the basal ganglia |
| HP:0004326 | 150 | Cachexia |
| HP:0008945 | 150 | Loss of ability to walk in early childhood |

---


---


---

# Cluster \*151

| HPO | Cluster | Description |
| --- | --- | --- |
| HP:0001265 | 151 | Hyporeflexia |
| HP:0003677 | 151 | Slow progression |
| HP:0008956 | 151 | Proximal lower limb amyotrophy |
| HP:0008994 | 151 | Proximal muscle weakness in lower limbs |
| HP:0006785 | 151 | Limb-girdle muscular dystrophy |
| HP:0002127 | 151 | Abnormal upper motor neuron morphology |
| HP:0000759 | 151 | Abnormal peripheral nervous system morphology |
| HP:0030237 | 151 | Hand muscle weakness |

---


---


---

# Cluster \*152

| HPO | Cluster | Description |
| --- | --- | --- |
| HP:0009830 | 152 | Peripheral neuropathy |
| HP:0003828 | 152 | Variable expressivity |
| HP:0001171 | 152 | Split hand |
| HP:0001425 | 152 | Heterogeneous |
| HP:0001283 | 152 | Bulbar palsy |
| HP:0001265 | 152 | Hyporeflexia |
| HP:0001761 | 152 | Pes cavus |
| HP:0003693 | 152 | Distal amyotrophy |
| HP:0003690 | 152 | Limb muscle weakness |
| HP:0003701 | 152 | Proximal muscle weakness |
| HP:0003307 | 152 | Hyperlordosis |
| HP:0010628 | 152 | Facial palsy |
| HP:0003324 | 152 | Generalized muscle weakness |
| HP:0003798 | 152 | Nemaline bodies |
| HP:0003803 | 152 | Type 1 muscle fiber predominance |
| HP:0001178 | 152 | Ulnar claw |
| HP:0001284 | 152 | Areflexia |
| HP:0001765 | 152 | Hammertoe |
| HP:0002460 | 152 | Distal muscle weakness |
| HP:0002936 | 152 | Distal sensory impairment |
| HP:0003376 | 152 | Steppage gait |
| HP:0003380 | 152 | Decreased number of peripheral myelinated nerve fibers |
| HP:0003382 | 152 | Hypertrophic nerve changes |
| HP:0003383 | 152 | Onion bulb formation |
| HP:0003431 | 152 | Decreased motor nerve conduction velocity |
| HP:0003449 | 152 | Cold-induced muscle cramps |
| HP:0004336 | 152 | Myelin outfoldings |
| HP:0009027 | 152 | Foot dorsiflexor weakness |
| HP:0003378 | 152 | Axonal degeneration/regeneration |
| HP:0003384 | 152 | Peripheral axonal atrophy |
| HP:0001612 | 152 | Weak cry |
| HP:0003458 | 152 | EMG: myopathic abnormalities |
| HP:0003200 | 152 | Ragged-red muscle fibers |
| HP:0003546 | 152 | Exercise intolerance |
| HP:0001533 | 152 | Slender build |
| HP:0002515 | 152 | Waddling gait |
| HP:0003390 | 152 | Sensory axonal neuropathy |
| HP:0002359 | 152 | Frequent falls |
| HP:0000590 | 152 | Progressive external ophthalmoplegia |
| HP:0003323 | 152 | Progressive muscle weakness |
| HP:0003548 | 152 | Subsarcolemmal accumulations of abnormally shaped mitochondria |
| HP:0003688 | 152 | Cytochrome C oxidase-negative muscle fibers |
| HP:0001604 | 152 | Vocal cord paresis |
| HP:0002355 | 152 | Difficulty walking |
| HP:0009046 | 152 | Difficulty running |
| HP:0002747 | 152 | Respiratory insufficiency due to muscle weakness |
| HP:0000467 | 152 | Neck muscle weakness |
| HP:0003554 | 152 | Type 2 muscle fiber atrophy |
| HP:0002058 | 152 | Myopathic facies |
| HP:0003306 | 152 | Spinal rigidity |
| HP:0003722 | 152 | Neck flexor weakness |
| HP:0003810 | 152 | Late-onset distal muscle weakness |
| HP:0003387 | 152 | Decreased number of large peripheral myelinated nerve fibers |
| HP:0003551 | 152 | Difficulty climbing stairs |
| HP:0003700 | 152 | Generalized amyotrophy |
| HP:0000597 | 152 | Ophthalmoparesis |
| HP:0003403 | 152 | EMG: decremental response of compound muscle action potential to repetitive nerve stimulation |
| HP:0003473 | 152 | Fatigable weakness |

---


---


---

# Cluster \*153

| HPO | Cluster | Description |
| --- | --- | --- |
| HP:0003676 | 153 | Progressive |
| HP:0003581 | 153 | Adult onset |
| HP:0001265 | 153 | Hyporeflexia |
| HP:0001761 | 153 | Pes cavus |
| HP:0002015 | 153 | Dysphagia |
| HP:0003693 | 153 | Distal amyotrophy |
| HP:0003701 | 153 | Proximal muscle weakness |
| HP:0003307 | 153 | Hyperlordosis |
| HP:0010628 | 153 | Facial palsy |
| HP:0001319 | 153 | Neonatal hypotonia |
| HP:0003324 | 153 | Generalized muscle weakness |
| HP:0003798 | 153 | Nemaline bodies |
| HP:0003803 | 153 | Type 1 muscle fiber predominance |
| HP:0001284 | 153 | Areflexia |
| HP:0002460 | 153 | Distal muscle weakness |
| HP:0002936 | 153 | Distal sensory impairment |
| HP:0003458 | 153 | EMG: myopathic abnormalities |
| HP:0003557 | 153 | Increased variability in muscle fiber diameter |
| HP:0001533 | 153 | Slender build |
| HP:0002515 | 153 | Waddling gait |
| HP:0002359 | 153 | Frequent falls |
| HP:0003547 | 153 | Shoulder girdle muscle weakness |
| HP:0008981 | 153 | Calf muscle hypertrophy |
| HP:0003749 | 153 | Pelvic girdle muscle weakness |
| HP:0003805 | 153 | Rimmed vacuoles |
| HP:0008180 | 153 | Mildly elevated creatine kinase |
| HP:0003306 | 153 | Spinal rigidity |
| HP:0003722 | 153 | Neck flexor weakness |
| HP:0003810 | 153 | Late-onset distal muscle weakness |
| HP:0003551 | 153 | Difficulty climbing stairs |
| HP:0003555 | 153 | Muscle fiber splitting |
| HP:0003741 | 153 | Congenital muscular dystrophy |

---


---


---

# Cluster \*154

| HPO | Cluster | Description |
| --- | --- | --- |
| HP:0001638 | 154 | Cardiomyopathy |
| HP:0001271 | 154 | Polyneuropathy |
| HP:0001265 | 154 | Hyporeflexia |
| HP:0002922 | 154 | Increased CSF protein |
| HP:0003678 | 154 | Rapidly progressive |
| HP:0003690 | 154 | Limb muscle weakness |
| HP:0001284 | 154 | Areflexia |
| HP:0003376 | 154 | Steppage gait |
| HP:0003383 | 154 | Onion bulb formation |
| HP:0011096 | 154 | Peripheral demyelination |
| HP:0000762 | 154 | Decreased nerve conduction velocity |
| HP:0007141 | 154 | Sensorimotor neuropathy |
| HP:0003434 | 154 | Sensory ataxic neuropathy |

---


---


---

# Cluster \*155

| HPO | Cluster | Description |
| --- | --- | --- |
| HP:0000007 | 155 | Autosomal recessive inheritance |
| HP:0001762 | 155 | Talipes equinovarus |
| HP:0001302 | 155 | Pachygyria |
| HP:0001270 | 155 | Motor delay |
| HP:0000508 | 155 | Ptosis |
| HP:0000518 | 155 | Cataract |
| HP:0002804 | 155 | Arthrogryposis multiplex congenita |
| HP:0001561 | 155 | Polyhydramnios |
| HP:0003577 | 155 | Congenital onset |
| HP:0001319 | 155 | Neonatal hypotonia |
| HP:0001371 | 155 | Flexion contracture |
| HP:0011968 | 155 | Feeding difficulties |
| HP:0001558 | 155 | Decreased fetal movement |
| HP:0002747 | 155 | Respiratory insufficiency due to muscle weakness |
| HP:0003687 | 155 | Centrally nucleated skeletal muscle fibers |
| HP:0002093 | 155 | Respiratory insufficiency |
| HP:0002058 | 155 | Myopathic facies |
| HP:0002033 | 155 | Poor suck |
| HP:0002365 | 155 | Hypoplasia of the brainstem |
| HP:0012110 | 155 | Hypoplasia of the pons |
| HP:0006829 | 155 | Severe muscular hypotonia |
| HP:0003741 | 155 | Congenital muscular dystrophy |
| HP:0007033 | 155 | Cerebellar dysplasia |
| HP:0007260 | 155 | Type II lissencephaly |
| HP:0002350 | 155 | Cerebellar cyst |
| HP:0002872 | 155 | Apneic episodes precipitated by illness, fatigue, stress |
| HP:0002882 | 155 | Sudden episodic apnea |
| HP:0003397 | 155 | Generalized hypotonia due to defect at the neuromuscular junction |
| HP:0003402 | 155 | Decreased miniature endplate potentials |

---


---


---

# Cluster \*156

| HPO | Cluster | Description |
| --- | --- | --- |
| HP:0001260 | 156 | Dysarthria |
| HP:0002385 | 156 | Paraparesis |
| HP:0003678 | 156 | Rapidly progressive |
| HP:0007340 | 156 | Lower limb muscle weakness |
| HP:0002500 | 156 | Abnormality of the cerebral white matter |

---


---


---

# Cluster \*157

| HPO | Cluster | Description |
| --- | --- | --- |
| HP:0003593 | 157 | Infantile onset |
| HP:0001349 | 157 | Facial diplegia |
| HP:0008972 | 157 | Decreased activity of mitochondrial respiratory chain |
| HP:0002134 | 157 | Abnormality of the basal ganglia |
| HP:0008945 | 157 | Loss of ability to walk in early childhood |

---


---


---

# Cluster \*158

| HPO | Cluster | Description |
| --- | --- | --- |
| HP:0001270 | 158 | Motor delay |
| HP:0000508 | 158 | Ptosis |
| HP:0003828 | 158 | Variable expressivity |
| HP:0003202 | 158 | Skeletal muscle atrophy |
| HP:0003674 | 158 | Onset |
| HP:0003676 | 158 | Progressive |
| HP:0001271 | 158 | Polyneuropathy |
| HP:0001283 | 158 | Bulbar palsy |
| HP:0003581 | 158 | Adult onset |
| HP:0001260 | 158 | Dysarthria |
| HP:0001265 | 158 | Hyporeflexia |
| HP:0001324 | 158 | Muscle weakness |
| HP:0003477 | 158 | Peripheral axonal neuropathy |
| HP:0002380 | 158 | Fasciculations |
| HP:0007354 | 158 | Amyotrophic lateral sclerosis |
| HP:0002366 | 158 | Abnormal lower motor neuron morphology |
| HP:0000741 | 158 | Apathy |
| HP:0002145 | 158 | Frontotemporal dementia |
| HP:0003678 | 158 | Rapidly progressive |
| HP:0007240 | 158 | Progressive gait ataxia |
| HP:0001288 | 158 | Gait disturbance |
| HP:0001761 | 158 | Pes cavus |
| HP:0002015 | 158 | Dysphagia |
| HP:0003677 | 158 | Slow progression |
| HP:0003693 | 158 | Distal amyotrophy |
| HP:0003701 | 158 | Proximal muscle weakness |
| HP:0010628 | 158 | Facial palsy |
| HP:0001284 | 158 | Areflexia |
| HP:0002460 | 158 | Distal muscle weakness |
| HP:0002936 | 158 | Distal sensory impairment |
| HP:0003621 | 158 | Juvenile onset |
| HP:0003200 | 158 | Ragged-red muscle fibers |
| HP:0001618 | 158 | Dysphonia |
| HP:0000762 | 158 | Decreased nerve conduction velocity |
| HP:0003390 | 158 | Sensory axonal neuropathy |
| HP:0007340 | 158 | Lower limb muscle weakness |
| HP:0002359 | 158 | Frequent falls |
| HP:0000590 | 158 | Progressive external ophthalmoplegia |
| HP:0003323 | 158 | Progressive muscle weakness |
| HP:0003548 | 158 | Subsarcolemmal accumulations of abnormally shaped mitochondria |
| HP:0003688 | 158 | Cytochrome C oxidase-negative muscle fibers |
| HP:0003713 | 158 | Muscle fiber necrosis |
| HP:0006886 | 158 | Impaired distal vibration sensation |
| HP:0002355 | 158 | Difficulty walking |
| HP:0002747 | 158 | Respiratory insufficiency due to muscle weakness |
| HP:0003554 | 158 | Type 2 muscle fiber atrophy |
| HP:0002058 | 158 | Myopathic facies |
| HP:0003448 | 158 | Decreased sensory nerve conduction velocity |
| HP:0008756 | 158 | Bowing of the vocal cords |
| HP:0000763 | 158 | Sensory neuropathy |
| HP:0002483 | 158 | Bulbar signs |
| HP:0007141 | 158 | Sensorimotor neuropathy |
| HP:0000597 | 158 | Ophthalmoparesis |
| HP:0002403 | 158 | Positive Romberg sign |
| HP:0003436 | 158 | Prolonged miniature endplate currents |
| HP:0003443 | 158 | Decreased size of nerve terminals |

---


---


---

# Cluster \*159

| HPO | Cluster | Description |
| --- | --- | --- |
| HP:0005010 | 159 | Osteomyelitis leading to amputation due to slow healing fractures |
| HP:0001414 | 159 | Microvesicular hepatic steatosis |
| HP:0002661 | 159 | Painless fractures due to injury |
| HP:0007021 | 159 | Pain insensitivity |
| HP:0006121 | 159 | Acral ulceration |

---


---


---

# Cluster \*160

| HPO | Cluster | Description |
| --- | --- | --- |
| HP:0001265 | 160 | Hyporeflexia |
| HP:0007240 | 160 | Progressive gait ataxia |
| HP:0001761 | 160 | Pes cavus |
| HP:0003693 | 160 | Distal amyotrophy |
| HP:0001284 | 160 | Areflexia |
| HP:0002460 | 160 | Distal muscle weakness |
| HP:0003431 | 160 | Decreased motor nerve conduction velocity |
| HP:0003236 | 160 | Elevated serum creatine kinase |
| HP:0003548 | 160 | Subsarcolemmal accumulations of abnormally shaped mitochondria |
| HP:0003688 | 160 | Cytochrome C oxidase-negative muscle fibers |
| HP:0003713 | 160 | Muscle fiber necrosis |
| HP:0006886 | 160 | Impaired distal vibration sensation |
| HP:0003387 | 160 | Decreased number of large peripheral myelinated nerve fibers |
| HP:0003448 | 160 | Decreased sensory nerve conduction velocity |
| HP:0007267 | 160 | Chronic axonal neuropathy |
| HP:0002403 | 160 | Positive Romberg sign |
| HP:0003434 | 160 | Sensory ataxic neuropathy |
| HP:0006937 | 160 | Impaired distal tactile sensation |

---


---


---

# Cluster \*161

| HPO | Cluster | Description |
| --- | --- | --- |
| HP:0000544 | 161 | External ophthalmoplegia |
| HP:0010628 | 161 | Facial palsy |
| HP:0003691 | 161 | Scapular winging |
| HP:0002111 | 161 | Restrictive deficit on pulmonary function testing |
| HP:0003547 | 161 | Shoulder girdle muscle weakness |
| HP:0003724 | 161 | Shoulder girdle muscle atrophy |
| HP:0008970 | 161 | Scapulohumeral muscular dystrophy |
| HP:0008981 | 161 | Calf muscle hypertrophy |
| HP:0030664 | 161 | Beevor’s sign |
| HP:0008988 | 161 | Pelvic girdle muscle atrophy |
| HP:0003749 | 161 | Pelvic girdle muscle weakness |

---


---


---

# Cluster \*162

| HPO | Cluster | Description |
| --- | --- | --- |
| HP:0001265 | 162 | Hyporeflexia |
| HP:0001761 | 162 | Pes cavus |
| HP:0001284 | 162 | Areflexia |
| HP:0002460 | 162 | Distal muscle weakness |
| HP:0003380 | 162 | Decreased number of peripheral myelinated nerve fibers |
| HP:0003378 | 162 | Axonal degeneration/regeneration |
| HP:0003384 | 162 | Peripheral axonal atrophy |
| HP:0001868 | 162 | Autoamputation of foot |
| HP:0001886 | 162 | Foot osteomyelitis |
| HP:0003387 | 162 | Decreased number of large peripheral myelinated nerve fibers |
| HP:0003448 | 162 | Decreased sensory nerve conduction velocity |
| HP:0007267 | 162 | Chronic axonal neuropathy |
| HP:0000763 | 162 | Sensory neuropathy |

---


---


---

# Cluster \*163

| HPO | Cluster | Description |
| --- | --- | --- |
| HP:0002015 | 163 | Dysphagia |
| HP:0010628 | 163 | Facial palsy |
| HP:0002375 | 163 | Hypokinesia |
| HP:0001558 | 163 | Decreased fetal movement |
| HP:0000467 | 163 | Neck muscle weakness |
| HP:0003700 | 163 | Generalized amyotrophy |
| HP:0002490 | 163 | Increased CSF lactate |
| HP:0006829 | 163 | Severe muscular hypotonia |

---


---


---

# Cluster \*164

| HPO | Cluster | Description |
| --- | --- | --- |
| HP:0001762 | 164 | Talipes equinovarus |
| HP:0009830 | 164 | Peripheral neuropathy |
| HP:0003828 | 164 | Variable expressivity |
| HP:0001265 | 164 | Hyporeflexia |
| HP:0003477 | 164 | Peripheral axonal neuropathy |
| HP:0001761 | 164 | Pes cavus |
| HP:0003693 | 164 | Distal amyotrophy |
| HP:0003701 | 164 | Proximal muscle weakness |
| HP:0003307 | 164 | Hyperlordosis |
| HP:0010628 | 164 | Facial palsy |
| HP:0001284 | 164 | Areflexia |
| HP:0002460 | 164 | Distal muscle weakness |
| HP:0002936 | 164 | Distal sensory impairment |
| HP:0003376 | 164 | Steppage gait |
| HP:0003383 | 164 | Onion bulb formation |
| HP:0003431 | 164 | Decreased motor nerve conduction velocity |
| HP:0009027 | 164 | Foot dorsiflexor weakness |
| HP:0002515 | 164 | Waddling gait |
| HP:0001604 | 164 | Vocal cord paresis |
| HP:0002355 | 164 | Difficulty walking |
| HP:0003547 | 164 | Shoulder girdle muscle weakness |
| HP:0008981 | 164 | Calf muscle hypertrophy |
| HP:0003749 | 164 | Pelvic girdle muscle weakness |
| HP:0003805 | 164 | Rimmed vacuoles |
| HP:0002058 | 164 | Myopathic facies |
| HP:0000764 | 164 | Peripheral axonal degeneration |
| HP:0003387 | 164 | Decreased number of large peripheral myelinated nerve fibers |
| HP:0003551 | 164 | Difficulty climbing stairs |
| HP:0003400 | 164 | Basal lamina onion bulb formation |

---


---


---

# Cluster \*165

| HPO | Cluster | Description |
| --- | --- | --- |
| HP:0000007 | 165 | Autosomal recessive inheritance |
| HP:0001762 | 165 | Talipes equinovarus |
| HP:0001270 | 165 | Motor delay |
| HP:0000508 | 165 | Ptosis |
| HP:0000218 | 165 | High palate |
| HP:0002650 | 165 | Scoliosis |
| HP:0001171 | 165 | Split hand |
| HP:0001265 | 165 | Hyporeflexia |
| HP:0040078 | 165 | Axonal degeneration |
| HP:0002804 | 165 | Arthrogryposis multiplex congenita |
| HP:0001761 | 165 | Pes cavus |
| HP:0001561 | 165 | Polyhydramnios |
| HP:0003307 | 165 | Hyperlordosis |
| HP:0010628 | 165 | Facial palsy |
| HP:0001319 | 165 | Neonatal hypotonia |
| HP:0001371 | 165 | Flexion contracture |
| HP:0001284 | 165 | Areflexia |
| HP:0002460 | 165 | Distal muscle weakness |
| HP:0002936 | 165 | Distal sensory impairment |
| HP:0003380 | 165 | Decreased number of peripheral myelinated nerve fibers |
| HP:0002515 | 165 | Waddling gait |
| HP:0002878 | 165 | Respiratory failure |
| HP:0011968 | 165 | Feeding difficulties |
| HP:0003691 | 165 | Scapular winging |
| HP:0003391 | 165 | Gowers sign |

---


---


---

# Cluster \*167

| HPO | Cluster | Description |
| --- | --- | --- |
| HP:0001265 | 167 | Hyporeflexia |
| HP:0001761 | 167 | Pes cavus |
| HP:0002936 | 167 | Distal sensory impairment |
| HP:0003200 | 167 | Ragged-red muscle fibers |
| HP:0003557 | 167 | Increased variability in muscle fiber diameter |
| HP:0003390 | 167 | Sensory axonal neuropathy |
| HP:0003445 | 167 | EMG: neuropathic changes |
| HP:0003805 | 167 | Rimmed vacuoles |
| HP:0003555 | 167 | Muscle fiber splitting |
| HP:0009129 | 167 | Upper limb amyotrophy |

---


---


---

# Cluster \*168

| HPO | Cluster | Description |
| --- | --- | --- |
| HP:0001762 | 168 | Talipes equinovarus |
| HP:0000508 | 168 | Ptosis |
| HP:0001188 | 168 | Hand clenching |
| HP:0001371 | 168 | Flexion contracture |
| HP:0003306 | 168 | Spinal rigidity |
| HP:0001302 | 168 | Pachygyria |
| HP:0000518 | 168 | Cataract |
| HP:0002365 | 168 | Hypoplasia of the brainstem |
| HP:0007260 | 168 | Type II lissencephaly |

---


---


---

# Cluster \*169

| HPO | Cluster | Description |
| --- | --- | --- |
| HP:0001252 | 169 | Muscular hypotonia |
| HP:0001270 | 169 | Motor delay |
| HP:0000218 | 169 | High palate |
| HP:0002650 | 169 | Scoliosis |
| HP:0000602 | 169 | Ophthalmoplegia |
| HP:0000544 | 169 | External ophthalmoplegia |
| HP:0003701 | 169 | Proximal muscle weakness |
| HP:0003307 | 169 | Hyperlordosis |
| HP:0010628 | 169 | Facial palsy |
| HP:0001319 | 169 | Neonatal hypotonia |
| HP:0003324 | 169 | Generalized muscle weakness |
| HP:0003798 | 169 | Nemaline bodies |
| HP:0003557 | 169 | Increased variability in muscle fiber diameter |
| HP:0002515 | 169 | Waddling gait |
| HP:0003691 | 169 | Scapular winging |
| HP:0009046 | 169 | Difficulty running |
| HP:0003391 | 169 | Gowers sign |
| HP:0002093 | 169 | Respiratory insufficiency |
| HP:0003306 | 169 | Spinal rigidity |
| HP:0009025 | 169 | Increased connective tissue |
| HP:0003327 | 169 | Axial muscle weakness |
| HP:0003787 | 169 | Type 1 and type 2 muscle fiber minicore regions |

---


---


---

# Cluster \*170

| HPO | Cluster | Description |
| --- | --- | --- |
| HP:0001283 | 170 | Bulbar palsy |
| HP:0001265 | 170 | Hyporeflexia |
| HP:0003701 | 170 | Proximal muscle weakness |
| HP:0010628 | 170 | Facial palsy |
| HP:0001284 | 170 | Areflexia |
| HP:0002936 | 170 | Distal sensory impairment |
| HP:0009027 | 170 | Foot dorsiflexor weakness |
| HP:0009113 | 170 | Diaphragmatic weakness |
| HP:0011349 | 170 | Abducens palsy |
| HP:0008955 | 170 | Progressive distal muscular atrophy |
| HP:0009063 | 170 | Progressive distal muscle weakness |

---


---


---

# Cluster \*171

| HPO | Cluster | Description |
| --- | --- | --- |
| HP:0003674 | 171 | Onset |
| HP:0001265 | 171 | Hyporeflexia |
| HP:0001761 | 171 | Pes cavus |
| HP:0001178 | 171 | Ulnar claw |
| HP:0001284 | 171 | Areflexia |
| HP:0001765 | 171 | Hammertoe |
| HP:0002460 | 171 | Distal muscle weakness |
| HP:0009027 | 171 | Foot dorsiflexor weakness |
| HP:0001604 | 171 | Vocal cord paresis |
| HP:0008180 | 171 | Mildly elevated creatine kinase |
| HP:0000763 | 171 | Sensory neuropathy |
| HP:0003444 | 171 | EMG: chronic denervation signs |
| HP:0006937 | 171 | Impaired distal tactile sensation |
| HP:0011349 | 171 | Abducens palsy |
| HP:0012246 | 171 | Oculomotor nerve palsy |
| HP:0007230 | 171 | Decreased distal sensory nerve action potential |

---


---


---

# Cluster \*172

| HPO | Cluster | Description |
| --- | --- | --- |
| HP:0002398 | 172 | Degeneration of anterior horn cells |
| HP:0040078 | 172 | Axonal degeneration |
| HP:0000764 | 172 | Peripheral axonal degeneration |
| HP:0005348 | 172 | Inspiratory stridor |
| HP:0006597 | 172 | Diaphragmatic paralysis |

---


---


---

# Cluster \*173

| HPO | Cluster | Description |
| --- | --- | --- |
| HP:0001252 | 173 | Muscular hypotonia |
| HP:0008872 | 173 | Feeding difficulties in infancy |
| HP:0008000 | 173 | Decreased corneal reflex |
| HP:0000224 | 173 | Decreased taste sensation |
| HP:0001069 | 173 | Episodic hyperhidrosis |
| HP:0000975 | 173 | Hyperhidrosis |

---


---


---

# Cluster \*174

| HPO | Cluster | Description |
| --- | --- | --- |
| HP:0001302 | 174 | Pachygyria |
| HP:0001270 | 174 | Motor delay |
| HP:0000508 | 174 | Ptosis |
| HP:0000557 | 174 | Buphthalmos |
| HP:0003828 | 174 | Variable expressivity |
| HP:0002650 | 174 | Scoliosis |
| HP:0001638 | 174 | Cardiomyopathy |
| HP:0003202 | 174 | Skeletal muscle atrophy |
| HP:0000518 | 174 | Cataract |
| HP:0003676 | 174 | Progressive |
| HP:0003581 | 174 | Adult onset |
| HP:0001260 | 174 | Dysarthria |
| HP:0001265 | 174 | Hyporeflexia |
| HP:0001324 | 174 | Muscle weakness |
| HP:0003394 | 174 | Muscle spasm |
| HP:0000158 | 174 | Macroglossia |
| HP:0011675 | 174 | Arrhythmia |
| HP:0002486 | 174 | Myotonia |
| HP:0001761 | 174 | Pes cavus |
| HP:0002015 | 174 | Dysphagia |
| HP:0003677 | 174 | Slow progression |
| HP:0000544 | 174 | External ophthalmoplegia |
| HP:0003693 | 174 | Distal amyotrophy |
| HP:0003198 | 174 | Myopathy |
| HP:0003325 | 174 | Limb-girdle muscle weakness |
| HP:0003690 | 174 | Limb muscle weakness |
| HP:0003701 | 174 | Proximal muscle weakness |
| HP:0001611 | 174 | Nasal speech |
| HP:0003307 | 174 | Hyperlordosis |
| HP:0010628 | 174 | Facial palsy |
| HP:0001644 | 174 | Dilated cardiomyopathy |
| HP:0001319 | 174 | Neonatal hypotonia |
| HP:0001371 | 174 | Flexion contracture |
| HP:0003324 | 174 | Generalized muscle weakness |
| HP:0001284 | 174 | Areflexia |
| HP:0002460 | 174 | Distal muscle weakness |
| HP:0002936 | 174 | Distal sensory impairment |
| HP:0009027 | 174 | Foot dorsiflexor weakness |
| HP:0003326 | 174 | Myalgia |
| HP:0003236 | 174 | Elevated serum creatine kinase |
| HP:0003458 | 174 | EMG: myopathic abnormalities |
| HP:0003560 | 174 | Muscular dystrophy |
| HP:0003710 | 174 | Exercise-induced muscle cramps |
| HP:0003737 | 174 | Mitochondrial myopathy |
| HP:0012378 | 174 | Fatigue |
| HP:0003200 | 174 | Ragged-red muscle fibers |
| HP:0003201 | 174 | Rhabdomyolysis |
| HP:0003546 | 174 | Exercise intolerance |
| HP:0003557 | 174 | Increased variability in muscle fiber diameter |
| HP:0003712 | 174 | Skeletal muscle hypertrophy |
| HP:0001321 | 174 | Cerebellar hypoplasia |
| HP:0002515 | 174 | Waddling gait |
| HP:0003388 | 174 | Easy fatigability |
| HP:0003390 | 174 | Sensory axonal neuropathy |
| HP:0003552 | 174 | Muscle stiffness |
| HP:0002913 | 174 | Myoglobinuria |
| HP:0003691 | 174 | Scapular winging |
| HP:0002359 | 174 | Frequent falls |
| HP:0000590 | 174 | Progressive external ophthalmoplegia |
| HP:0003323 | 174 | Progressive muscle weakness |
| HP:0002355 | 174 | Difficulty walking |
| HP:0002522 | 174 | Areflexia of lower limbs |
| HP:0002600 | 174 | Hyporeflexia of lower limbs |
| HP:0008956 | 174 | Proximal lower limb amyotrophy |
| HP:0008994 | 174 | Proximal muscle weakness in lower limbs |
| HP:0009046 | 174 | Difficulty running |
| HP:0001558 | 174 | Decreased fetal movement |
| HP:0002747 | 174 | Respiratory insufficiency due to muscle weakness |
| HP:0006466 | 174 | Ankle flexion contracture |
| HP:0002111 | 174 | Restrictive deficit on pulmonary function testing |
| HP:0003547 | 174 | Shoulder girdle muscle weakness |
| HP:0003724 | 174 | Shoulder girdle muscle atrophy |
| HP:0008981 | 174 | Calf muscle hypertrophy |
| HP:0003749 | 174 | Pelvic girdle muscle weakness |
| HP:0003391 | 174 | Gowers sign |
| HP:0003687 | 174 | Centrally nucleated skeletal muscle fibers |
| HP:0000467 | 174 | Neck muscle weakness |
| HP:0003805 | 174 | Rimmed vacuoles |
| HP:0002093 | 174 | Respiratory insufficiency |
| HP:0003738 | 174 | Exercise-induced myalgia |
| HP:0007126 | 174 | Proximal amyotrophy |
| HP:0002058 | 174 | Myopathic facies |
| HP:0003306 | 174 | Spinal rigidity |
| HP:0003722 | 174 | Neck flexor weakness |
| HP:0003736 | 174 | Autophagic vacuoles |
| HP:0008756 | 174 | Bowing of the vocal cords |
| HP:0003551 | 174 | Difficulty climbing stairs |
| HP:0008988 | 174 | Pelvic girdle muscle atrophy |
| HP:0001771 | 174 | Achilles tendon contracture |
| HP:0006785 | 174 | Limb-girdle muscular dystrophy |
| HP:0003704 | 174 | Scapuloperoneal weakness |
| HP:0003555 | 174 | Muscle fiber splitting |
| HP:0003697 | 174 | Scapuloperoneal amyotrophy |
| HP:0009063 | 174 | Progressive distal muscle weakness |
| HP:0002365 | 174 | Hypoplasia of the brainstem |
| HP:0002803 | 174 | Congenital contracture |
| HP:0003700 | 174 | Generalized amyotrophy |
| HP:0007973 | 174 | Retinal dysplasia |
| HP:0012110 | 174 | Hypoplasia of the pons |
| HP:0009025 | 174 | Increased connective tissue |
| HP:0006829 | 174 | Severe muscular hypotonia |
| HP:0003741 | 174 | Congenital muscular dystrophy |
| HP:0007033 | 174 | Cerebellar dysplasia |
| HP:0007260 | 174 | Type II lissencephaly |
| HP:0031882 | 174 | Agyria |
| HP:0008967 | 174 | Exercise-induced muscle stiffness |
| HP:0040083 | 174 | Toe walking |
| HP:0002350 | 174 | Cerebellar cyst |
| HP:0100614 | 174 | Myositis |
| HP:0003707 | 174 | Calf muscle pseudohypertrophy |
| HP:0003731 | 174 | Quadriceps muscle weakness |
| HP:0003791 | 174 | Deposits immunoreactive to beta-amyloid protein |
| HP:0009072 | 174 | Decreased Achilles reflex |
| HP:0003715 | 174 | Myofibrillar myopathy |
| HP:0009055 | 174 | Generalized limb muscle atrophy |
| HP:0008948 | 174 | Proximal upper limb amyotrophy |
| HP:0002792 | 174 | Reduced vital capacity |
| HP:0003719 | 174 | Muscle mounding |
| HP:0003733 | 174 | Thigh hypertrophy |
| HP:0100297 | 174 | Increased endomysial connective tissue |
| HP:0100303 | 174 | Muscle fiber cytoplasmatic inclusion bodies |
| HP:0030046 | 174 | Hypoglycosylation of alpha-dystroglycan |

---


---


---

# Cluster \*175

| HPO | Cluster | Description |
| --- | --- | --- |
| HP:0000508 | 175 | Ptosis |
| HP:0000218 | 175 | High palate |
| HP:0002650 | 175 | Scoliosis |
| HP:0003674 | 175 | Onset |
| HP:0003676 | 175 | Progressive |
| HP:0001283 | 175 | Bulbar palsy |
| HP:0001265 | 175 | Hyporeflexia |
| HP:0001324 | 175 | Muscle weakness |
| HP:0002804 | 175 | Arthrogryposis multiplex congenita |
| HP:0000602 | 175 | Ophthalmoplegia |
| HP:0001761 | 175 | Pes cavus |
| HP:0002015 | 175 | Dysphagia |
| HP:0002093 | 175 | Respiratory insufficiency |
| HP:0003677 | 175 | Slow progression |
| HP:0003693 | 175 | Distal amyotrophy |
| HP:0003198 | 175 | Myopathy |
| HP:0003701 | 175 | Proximal muscle weakness |
| HP:0001611 | 175 | Nasal speech |
| HP:0010628 | 175 | Facial palsy |
| HP:0001319 | 175 | Neonatal hypotonia |
| HP:0003324 | 175 | Generalized muscle weakness |
| HP:0003798 | 175 | Nemaline bodies |
| HP:0001284 | 175 | Areflexia |
| HP:0002460 | 175 | Distal muscle weakness |
| HP:0011463 | 175 | Childhood onset |
| HP:0001612 | 175 | Weak cry |
| HP:0003458 | 175 | EMG: myopathic abnormalities |
| HP:0003737 | 175 | Mitochondrial myopathy |
| HP:0003200 | 175 | Ragged-red muscle fibers |
| HP:0003546 | 175 | Exercise intolerance |
| HP:0003557 | 175 | Increased variability in muscle fiber diameter |
| HP:0001618 | 175 | Dysphonia |
| HP:0003388 | 175 | Easy fatigability |
| HP:0011968 | 175 | Feeding difficulties |
| HP:0000590 | 175 | Progressive external ophthalmoplegia |
| HP:0003323 | 175 | Progressive muscle weakness |
| HP:0003548 | 175 | Subsarcolemmal accumulations of abnormally shaped mitochondria |
| HP:0003688 | 175 | Cytochrome C oxidase-negative muscle fibers |
| HP:0006886 | 175 | Impaired distal vibration sensation |
| HP:0001558 | 175 | Decreased fetal movement |
| HP:0002747 | 175 | Respiratory insufficiency due to muscle weakness |
| HP:0003547 | 175 | Shoulder girdle muscle weakness |
| HP:0000467 | 175 | Neck muscle weakness |
| HP:0003554 | 175 | Type 2 muscle fiber atrophy |
| HP:0002058 | 175 | Myopathic facies |
| HP:0003306 | 175 | Spinal rigidity |
| HP:0003722 | 175 | Neck flexor weakness |
| HP:0003810 | 175 | Late-onset distal muscle weakness |
| HP:0002033 | 175 | Poor suck |
| HP:0006829 | 175 | Severe muscular hypotonia |
| HP:0000597 | 175 | Ophthalmoparesis |
| HP:0002872 | 175 | Apneic episodes precipitated by illness, fatigue, stress |
| HP:0002882 | 175 | Sudden episodic apnea |
| HP:0003397 | 175 | Generalized hypotonia due to defect at the neuromuscular junction |
| HP:0003402 | 175 | Decreased miniature endplate potentials |
| HP:0003403 | 175 | EMG: decremental response of compound muscle action potential to repetitive nerve stimulation |
| HP:0003473 | 175 | Fatigable weakness |
| HP:0002403 | 175 | Positive Romberg sign |
| HP:0003436 | 175 | Prolonged miniature endplate currents |
| HP:0003443 | 175 | Decreased size of nerve terminals |

---


---


---

# Cluster \*176

| HPO | Cluster | Description |
| --- | --- | --- |
| HP:0001270 | 176 | Motor delay |
| HP:0001265 | 176 | Hyporeflexia |
| HP:0001761 | 176 | Pes cavus |
| HP:0003693 | 176 | Distal amyotrophy |
| HP:0003701 | 176 | Proximal muscle weakness |
| HP:0010628 | 176 | Facial palsy |
| HP:0001319 | 176 | Neonatal hypotonia |
| HP:0001371 | 176 | Flexion contracture |
| HP:0003803 | 176 | Type 1 muscle fiber predominance |
| HP:0001284 | 176 | Areflexia |
| HP:0002460 | 176 | Distal muscle weakness |
| HP:0002936 | 176 | Distal sensory impairment |
| HP:0003376 | 176 | Steppage gait |
| HP:0003380 | 176 | Decreased number of peripheral myelinated nerve fibers |
| HP:0003431 | 176 | Decreased motor nerve conduction velocity |
| HP:0009027 | 176 | Foot dorsiflexor weakness |
| HP:0003458 | 176 | EMG: myopathic abnormalities |
| HP:0003200 | 176 | Ragged-red muscle fibers |
| HP:0003390 | 176 | Sensory axonal neuropathy |
| HP:0000590 | 176 | Progressive external ophthalmoplegia |
| HP:0003548 | 176 | Subsarcolemmal accumulations of abnormally shaped mitochondria |
| HP:0003688 | 176 | Cytochrome C oxidase-negative muscle fibers |
| HP:0003713 | 176 | Muscle fiber necrosis |
| HP:0006886 | 176 | Impaired distal vibration sensation |
| HP:0008981 | 176 | Calf muscle hypertrophy |
| HP:0002058 | 176 | Myopathic facies |
| HP:0003306 | 176 | Spinal rigidity |
| HP:0003810 | 176 | Late-onset distal muscle weakness |
| HP:0003551 | 176 | Difficulty climbing stairs |
| HP:0002403 | 176 | Positive Romberg sign |
| HP:0003434 | 176 | Sensory ataxic neuropathy |
| HP:0003733 | 176 | Thigh hypertrophy |

---


---


---

# Cluster \*177

| HPO | Cluster | Description |
| --- | --- | --- |
| HP:0000508 | 177 | Ptosis |
| HP:0003236 | 177 | Elevated serum creatine kinase |
| HP:0003200 | 177 | Ragged-red muscle fibers |
| HP:0002747 | 177 | Respiratory insufficiency due to muscle weakness |
| HP:0003700 | 177 | Generalized amyotrophy |
| HP:0008972 | 177 | Decreased activity of mitochondrial respiratory chain |
| HP:0008945 | 177 | Loss of ability to walk in early childhood |
| HP:0002134 | 177 | Abnormality of the basal ganglia |

---


---


---

# Cluster \*178

| HPO | Cluster | Description |
| --- | --- | --- |
| HP:0007240 | 178 | Progressive gait ataxia |
| HP:0003438 | 178 | Absent Achilles reflex |
| HP:0003693 | 178 | Distal amyotrophy |
| HP:0001765 | 178 | Hammertoe |
| HP:0002460 | 178 | Distal muscle weakness |
| HP:0002936 | 178 | Distal sensory impairment |
| HP:0003376 | 178 | Steppage gait |
| HP:0003448 | 178 | Decreased sensory nerve conduction velocity |

---


---


---

# Cluster \*179

| HPO | Cluster | Description |
| --- | --- | --- |
| HP:0000007 | 179 | Autosomal recessive inheritance |
| HP:0001762 | 179 | Talipes equinovarus |
| HP:0001252 | 179 | Muscular hypotonia |
| HP:0001302 | 179 | Pachygyria |
| HP:0001270 | 179 | Motor delay |
| HP:0000508 | 179 | Ptosis |
| HP:0000218 | 179 | High palate |
| HP:0002650 | 179 | Scoliosis |
| HP:0001638 | 179 | Cardiomyopathy |
| HP:0002751 | 179 | Kyphoscoliosis |
| HP:0003593 | 179 | Infantile onset |
| HP:0000518 | 179 | Cataract |
| HP:0001425 | 179 | Heterogeneous |
| HP:0001271 | 179 | Polyneuropathy |
| HP:0001265 | 179 | Hyporeflexia |
| HP:0001324 | 179 | Muscle weakness |
| HP:0002922 | 179 | Increased CSF protein |
| HP:0008872 | 179 | Feeding difficulties in infancy |
| HP:0002015 | 179 | Dysphagia |
| HP:0001561 | 179 | Polyhydramnios |
| HP:0003198 | 179 | Myopathy |
| HP:0001639 | 179 | Hypertrophic cardiomyopathy |
| HP:0001371 | 179 | Flexion contracture |
| HP:0011096 | 179 | Peripheral demyelination |
| HP:0001349 | 179 | Facial diplegia |
| HP:0001427 | 179 | Mitochondrial inheritance |
| HP:0003128 | 179 | Lactic acidosis |
| HP:0001321 | 179 | Cerebellar hypoplasia |
| HP:0002747 | 179 | Respiratory insufficiency due to muscle weakness |
| HP:0002093 | 179 | Respiratory insufficiency |
| HP:0002365 | 179 | Hypoplasia of the brainstem |
| HP:0002490 | 179 | Increased CSF lactate |
| HP:0003741 | 179 | Congenital muscular dystrophy |
| HP:0008972 | 179 | Decreased activity of mitochondrial respiratory chain |
| HP:0008945 | 179 | Loss of ability to walk in early childhood |

---


---


---

# Cluster \*180

| HPO | Cluster | Description |
| --- | --- | --- |
| HP:0000007 | 180 | Autosomal recessive inheritance |
| HP:0000218 | 180 | High palate |
| HP:0001283 | 180 | Bulbar palsy |
| HP:0001265 | 180 | Hyporeflexia |
| HP:0002804 | 180 | Arthrogryposis multiplex congenita |
| HP:0000602 | 180 | Ophthalmoplegia |
| HP:0001761 | 180 | Pes cavus |
| HP:0003307 | 180 | Hyperlordosis |
| HP:0001319 | 180 | Neonatal hypotonia |
| HP:0001371 | 180 | Flexion contracture |
| HP:0003324 | 180 | Generalized muscle weakness |
| HP:0003798 | 180 | Nemaline bodies |
| HP:0003803 | 180 | Type 1 muscle fiber predominance |
| HP:0001284 | 180 | Areflexia |
| HP:0002460 | 180 | Distal muscle weakness |
| HP:0009027 | 180 | Foot dorsiflexor weakness |
| HP:0003458 | 180 | EMG: myopathic abnormalities |
| HP:0002515 | 180 | Waddling gait |
| HP:0001558 | 180 | Decreased fetal movement |
| HP:0002747 | 180 | Respiratory insufficiency due to muscle weakness |
| HP:0003391 | 180 | Gowers sign |
| HP:0002093 | 180 | Respiratory insufficiency |
| HP:0002058 | 180 | Myopathic facies |
| HP:0003306 | 180 | Spinal rigidity |
| HP:0003810 | 180 | Late-onset distal muscle weakness |
| HP:0003700 | 180 | Generalized amyotrophy |
| HP:0001765 | 180 | Hammertoe |
| HP:0003378 | 180 | Axonal degeneration/regeneration |
| HP:0001533 | 180 | Slender build |
| HP:0000467 | 180 | Neck muscle weakness |
| HP:0007973 | 180 | Retinal dysplasia |
| HP:0006829 | 180 | Severe muscular hypotonia |
| HP:0003741 | 180 | Congenital muscular dystrophy |
| HP:0007260 | 180 | Type II lissencephaly |
| HP:0031882 | 180 | Agyria |
| HP:0003327 | 180 | Axial muscle weakness |

---


---


---

# Cluster \*181

| HPO | Cluster | Description |
| --- | --- | --- |
| HP:0000007 | 181 | Autosomal recessive inheritance |
| HP:0001270 | 181 | Motor delay |
| HP:0000508 | 181 | Ptosis |
| HP:0001265 | 181 | Hyporeflexia |
| HP:0001324 | 181 | Muscle weakness |
| HP:0003477 | 181 | Peripheral axonal neuropathy |
| HP:0002380 | 181 | Fasciculations |
| HP:0002398 | 181 | Degeneration of anterior horn cells |
| HP:0003394 | 181 | Muscle spasm |
| HP:0007354 | 181 | Amyotrophic lateral sclerosis |
| HP:0002366 | 181 | Abnormal lower motor neuron morphology |
| HP:0040078 | 181 | Axonal degeneration |
| HP:0001761 | 181 | Pes cavus |
| HP:0003198 | 181 | Myopathy |
| HP:0001639 | 181 | Hypertrophic cardiomyopathy |
| HP:0001371 | 181 | Flexion contracture |
| HP:0001284 | 181 | Areflexia |
| HP:0002936 | 181 | Distal sensory impairment |
| HP:0003326 | 181 | Myalgia |
| HP:0003737 | 181 | Mitochondrial myopathy |
| HP:0012378 | 181 | Fatigue |
| HP:0003200 | 181 | Ragged-red muscle fibers |
| HP:0003201 | 181 | Rhabdomyolysis |
| HP:0003546 | 181 | Exercise intolerance |
| HP:0003557 | 181 | Increased variability in muscle fiber diameter |
| HP:0003712 | 181 | Skeletal muscle hypertrophy |
| HP:0002878 | 181 | Respiratory failure |
| HP:0003552 | 181 | Muscle stiffness |
| HP:0011968 | 181 | Feeding difficulties |
| HP:0002913 | 181 | Myoglobinuria |
| HP:0002355 | 181 | Difficulty walking |
| HP:0007269 | 181 | Spinal muscular atrophy |
| HP:0008981 | 181 | Calf muscle hypertrophy |
| HP:0001308 | 181 | Tongue fasciculations |
| HP:0002093 | 181 | Respiratory insufficiency |
| HP:0003738 | 181 | Exercise-induced myalgia |
| HP:0010548 | 181 | Percussion myotonia |
| HP:0005348 | 181 | Inspiratory stridor |
| HP:0030046 | 181 | Hypoglycosylation of alpha-dystroglycan |

---


---


---

# Cluster \*182

| HPO | Cluster | Description |
| --- | --- | --- |
| HP:0001283 | 182 | Bulbar palsy |
| HP:0003581 | 182 | Adult onset |
| HP:0001265 | 182 | Hyporeflexia |
| HP:0003394 | 182 | Muscle spasm |
| HP:0007354 | 182 | Amyotrophic lateral sclerosis |
| HP:0002145 | 182 | Frontotemporal dementia |
| HP:0002015 | 182 | Dysphagia |
| HP:0003693 | 182 | Distal amyotrophy |
| HP:0002460 | 182 | Distal muscle weakness |
| HP:0002747 | 182 | Respiratory insufficiency due to muscle weakness |
| HP:0003547 | 182 | Shoulder girdle muscle weakness |
| HP:0003805 | 182 | Rimmed vacuoles |

---


---


---

# Cluster \*183

| HPO | Cluster | Description |
| --- | --- | --- |
| HP:0001270 | 183 | Motor delay |
| HP:0001425 | 183 | Heterogeneous |
| HP:0001265 | 183 | Hyporeflexia |
| HP:0001761 | 183 | Pes cavus |
| HP:0003693 | 183 | Distal amyotrophy |
| HP:0001611 | 183 | Nasal speech |
| HP:0010628 | 183 | Facial palsy |
| HP:0001319 | 183 | Neonatal hypotonia |
| HP:0001371 | 183 | Flexion contracture |
| HP:0003803 | 183 | Type 1 muscle fiber predominance |
| HP:0001284 | 183 | Areflexia |
| HP:0002460 | 183 | Distal muscle weakness |
| HP:0003376 | 183 | Steppage gait |
| HP:0003621 | 183 | Juvenile onset |
| HP:0009027 | 183 | Foot dorsiflexor weakness |
| HP:0001612 | 183 | Weak cry |
| HP:0003200 | 183 | Ragged-red muscle fibers |
| HP:0003390 | 183 | Sensory axonal neuropathy |
| HP:0000590 | 183 | Progressive external ophthalmoplegia |
| HP:0003548 | 183 | Subsarcolemmal accumulations of abnormally shaped mitochondria |
| HP:0003688 | 183 | Cytochrome C oxidase-negative muscle fibers |
| HP:0003713 | 183 | Muscle fiber necrosis |
| HP:0006886 | 183 | Impaired distal vibration sensation |
| HP:0002058 | 183 | Myopathic facies |
| HP:0003810 | 183 | Late-onset distal muscle weakness |
| HP:0020152 | 183 | Distal joint laxity |
| HP:0000597 | 183 | Ophthalmoparesis |
| HP:0003403 | 183 | EMG: decremental response of compound muscle action potential to repetitive nerve stimulation |
| HP:0003473 | 183 | Fatigable weakness |

---


---


---

# Cluster \*185

| HPO | Cluster | Description |
| --- | --- | --- |
| HP:0003380 | 185 | Decreased number of peripheral myelinated nerve fibers |
| HP:0003378 | 185 | Axonal degeneration/regeneration |
| HP:0001618 | 185 | Dysphonia |
| HP:0000590 | 185 | Progressive external ophthalmoplegia |
| HP:0001604 | 185 | Vocal cord paresis |
| HP:0007126 | 185 | Proximal amyotrophy |
| HP:0003306 | 185 | Spinal rigidity |
| HP:0003700 | 185 | Generalized amyotrophy |
| HP:0008443 | 185 | Spinal deformities |

---


---


---

# Cluster \*186

| HPO | Cluster | Description |
| --- | --- | --- |
| HP:0000007 | 186 | Autosomal recessive inheritance |
| HP:0001270 | 186 | Motor delay |
| HP:0000508 | 186 | Ptosis |
| HP:0003593 | 186 | Infantile onset |
| HP:0003676 | 186 | Progressive |
| HP:0001260 | 186 | Dysarthria |
| HP:0000602 | 186 | Ophthalmoplegia |
| HP:0002015 | 186 | Dysphagia |
| HP:0010628 | 186 | Facial palsy |
| HP:0001319 | 186 | Neonatal hypotonia |
| HP:0001371 | 186 | Flexion contracture |
| HP:0003324 | 186 | Generalized muscle weakness |
| HP:0003803 | 186 | Type 1 muscle fiber predominance |
| HP:0003431 | 186 | Decreased motor nerve conduction velocity |
| HP:0001612 | 186 | Weak cry |
| HP:0003200 | 186 | Ragged-red muscle fibers |
| HP:0003388 | 186 | Easy fatigability |
| HP:0011968 | 186 | Feeding difficulties |
| HP:0002093 | 186 | Respiratory insufficiency |
| HP:0003554 | 186 | Type 2 muscle fiber atrophy |
| HP:0003306 | 186 | Spinal rigidity |

---


---


---

# Cluster \*187

| HPO | Cluster | Description |
| --- | --- | --- |
| HP:0001270 | 187 | Motor delay |
| HP:0000508 | 187 | Ptosis |
| HP:0003828 | 187 | Variable expressivity |
| HP:0000218 | 187 | High palate |
| HP:0002650 | 187 | Scoliosis |
| HP:0002804 | 187 | Arthrogryposis multiplex congenita |
| HP:0010628 | 187 | Facial palsy |
| HP:0001319 | 187 | Neonatal hypotonia |
| HP:0001371 | 187 | Flexion contracture |
| HP:0001612 | 187 | Weak cry |
| HP:0011968 | 187 | Feeding difficulties |
| HP:0002355 | 187 | Difficulty walking |
| HP:0002093 | 187 | Respiratory insufficiency |
| HP:0003554 | 187 | Type 2 muscle fiber atrophy |
| HP:0002058 | 187 | Myopathic facies |
| HP:0003306 | 187 | Spinal rigidity |
| HP:0012110 | 187 | Hypoplasia of the pons |
| HP:0003443 | 187 | Decreased size of nerve terminals |

---


---


---

# Cluster \*188

| HPO | Cluster | Description |
| --- | --- | --- |
| HP:0000007 | 188 | Autosomal recessive inheritance |
| HP:0003678 | 188 | Rapidly progressive |
| HP:0001188 | 188 | Hand clenching |
| HP:0002804 | 188 | Arthrogryposis multiplex congenita |
| HP:0001561 | 188 | Polyhydramnios |
| HP:0000544 | 188 | External ophthalmoplegia |
| HP:0003577 | 188 | Congenital onset |
| HP:0001319 | 188 | Neonatal hypotonia |
| HP:0001371 | 188 | Flexion contracture |
| HP:0001612 | 188 | Weak cry |
| HP:0002878 | 188 | Respiratory failure |
| HP:0011968 | 188 | Feeding difficulties |
| HP:0001558 | 188 | Decreased fetal movement |
| HP:0002093 | 188 | Respiratory insufficiency |
| HP:0009113 | 188 | Diaphragmatic weakness |
| HP:0006829 | 188 | Severe muscular hypotonia |
| HP:0003715 | 188 | Myofibrillar myopathy |

---


---


---

# Cluster \*189

| HPO | Cluster | Description |
| --- | --- | --- |
| HP:0003557 | 189 | Increased variability in muscle fiber diameter |
| HP:0008956 | 189 | Proximal lower limb amyotrophy |
| HP:0008994 | 189 | Proximal muscle weakness in lower limbs |
| HP:0009046 | 189 | Difficulty running |
| HP:0003547 | 189 | Shoulder girdle muscle weakness |
| HP:0008981 | 189 | Calf muscle hypertrophy |
| HP:0003749 | 189 | Pelvic girdle muscle weakness |
| HP:0003805 | 189 | Rimmed vacuoles |
| HP:0003551 | 189 | Difficulty climbing stairs |
| HP:0006785 | 189 | Limb-girdle muscular dystrophy |
| HP:0009025 | 189 | Increased connective tissue |
| HP:0008948 | 189 | Proximal upper limb amyotrophy |
| HP:0011808 | 189 | Decreased patellar reflex |

---


---


---

# Cluster \*190

| HPO | Cluster | Description |
| --- | --- | --- |
| HP:0003394 | 190 | Muscle spasm |
| HP:0003677 | 190 | Slow progression |
| HP:0000544 | 190 | External ophthalmoplegia |
| HP:0003701 | 190 | Proximal muscle weakness |
| HP:0003557 | 190 | Increased variability in muscle fiber diameter |
| HP:0002522 | 190 | Areflexia of lower limbs |
| HP:0002600 | 190 | Hyporeflexia of lower limbs |
| HP:0007269 | 190 | Spinal muscular atrophy |
| HP:0009046 | 190 | Difficulty running |
| HP:0003554 | 190 | Type 2 muscle fiber atrophy |
| HP:0003738 | 190 | Exercise-induced myalgia |
| HP:0007126 | 190 | Proximal amyotrophy |
| HP:0009005 | 190 | Weakness of the intrinsic hand muscles |
| HP:0003444 | 190 | EMG: chronic denervation signs |

---


---


---

# Cluster \*191

| HPO | Cluster | Description |
| --- | --- | --- |
| HP:0002021 | 191 | Pyloric stenosis |
| HP:0002650 | 191 | Scoliosis |
| HP:0000518 | 191 | Cataract |
| HP:0001561 | 191 | Polyhydramnios |
| HP:0001611 | 191 | Nasal speech |
| HP:0001319 | 191 | Neonatal hypotonia |
| HP:0000467 | 191 | Neck muscle weakness |
| HP:0002033 | 191 | Poor suck |

---


---


---

# Cluster \*192

| HPO | Cluster | Description |
| --- | --- | --- |
| HP:0003693 | 192 | Distal amyotrophy |
| HP:0003325 | 192 | Limb-girdle muscle weakness |
| HP:0002460 | 192 | Distal muscle weakness |
| HP:0003376 | 192 | Steppage gait |
| HP:0009027 | 192 | Foot dorsiflexor weakness |
| HP:0003805 | 192 | Rimmed vacuoles |
| HP:0003551 | 192 | Difficulty climbing stairs |
| HP:0003737 | 192 | Mitochondrial myopathy |

---


---


---

# Cluster \*194

| HPO | Cluster | Description |
| --- | --- | --- |
| HP:0001271 | 194 | Polyneuropathy |
| HP:0001324 | 194 | Muscle weakness |
| HP:0011675 | 194 | Arrhythmia |
| HP:0001288 | 194 | Gait disturbance |
| HP:0003693 | 194 | Distal amyotrophy |
| HP:0002460 | 194 | Distal muscle weakness |
| HP:0006958 | 194 | Abnormal auditory evoked potentials |
| HP:0000762 | 194 | Decreased nerve conduction velocity |

---


---


---

# Cluster \*195

| HPO | Cluster | Description |
| --- | --- | --- |
| HP:0002922 | 195 | Increased CSF protein |
| HP:0003470 | 195 | Paralysis |
| HP:0001414 | 195 | Microvesicular hepatic steatosis |
| HP:0003219 | 195 | Ethylmalonic aciduria |
| HP:0012847 | 195 | Epilepsia partialis continua |
| HP:0006957 | 195 | Loss of ability to walk |

---


---


---

# Cluster \*196

| HPO | Cluster | Description |
| --- | --- | --- |
| HP:0003828 | 196 | Variable expressivity |
| HP:0003701 | 196 | Proximal muscle weakness |
| HP:0010628 | 196 | Facial palsy |
| HP:0003803 | 196 | Type 1 muscle fiber predominance |
| HP:0003557 | 196 | Increased variability in muscle fiber diameter |
| HP:0002359 | 196 | Frequent falls |
| HP:0003713 | 196 | Muscle fiber necrosis |
| HP:0002355 | 196 | Difficulty walking |
| HP:0002111 | 196 | Restrictive deficit on pulmonary function testing |
| HP:0003547 | 196 | Shoulder girdle muscle weakness |
| HP:0008981 | 196 | Calf muscle hypertrophy |
| HP:0003749 | 196 | Pelvic girdle muscle weakness |
| HP:0003306 | 196 | Spinal rigidity |
| HP:0003722 | 196 | Neck flexor weakness |
| HP:0003700 | 196 | Generalized amyotrophy |
| HP:0002877 | 196 | Nocturnal hypoventilation |
| HP:0020152 | 196 | Distal joint laxity |
| HP:0003741 | 196 | Congenital muscular dystrophy |
| HP:0003327 | 196 | Axial muscle weakness |
| HP:0032341 | 196 | Reduced forced vital capacity |
| HP:0003787 | 196 | Type 1 and type 2 muscle fiber minicore regions |
| HP:0002792 | 196 | Reduced vital capacity |
| HP:0003733 | 196 | Thigh hypertrophy |

---


---


---

# Cluster \*197

| HPO | Cluster | Description |
| --- | --- | --- |
| HP:0003202 | 197 | Skeletal muscle atrophy |
| HP:0003676 | 197 | Progressive |
| HP:0001283 | 197 | Bulbar palsy |
| HP:0003581 | 197 | Adult onset |
| HP:0002380 | 197 | Fasciculations |
| HP:0007354 | 197 | Amyotrophic lateral sclerosis |
| HP:0002366 | 197 | Abnormal lower motor neuron morphology |
| HP:0000741 | 197 | Apathy |
| HP:0002145 | 197 | Frontotemporal dementia |
| HP:0002015 | 197 | Dysphagia |
| HP:0002747 | 197 | Respiratory insufficiency due to muscle weakness |
| HP:0002483 | 197 | Bulbar signs |
| HP:0003678 | 197 | Rapidly progressive |
| HP:0002127 | 197 | Abnormal upper motor neuron morphology |

---


---


---

# Cluster \*199

| HPO | Cluster | Description |
| --- | --- | --- |
| HP:0002366 | 199 | Abnormal lower motor neuron morphology |
| HP:0008756 | 199 | Bowing of the vocal cords |
| HP:0002127 | 199 | Abnormal upper motor neuron morphology |
| HP:0009130 | 199 | Hand muscle atrophy |
| HP:0003697 | 199 | Scapuloperoneal amyotrophy |
| HP:0002483 | 199 | Bulbar signs |
| HP:0030237 | 199 | Hand muscle weakness |

---


---


---

# Cluster \*202

| HPO | Cluster | Description |
| --- | --- | --- |
| HP:0001762 | 202 | Talipes equinovarus |
| HP:0002650 | 202 | Scoliosis |
| HP:0003677 | 202 | Slow progression |
| HP:0003307 | 202 | Hyperlordosis |
| HP:0003803 | 202 | Type 1 muscle fiber predominance |
| HP:0001284 | 202 | Areflexia |
| HP:0003458 | 202 | EMG: myopathic abnormalities |
| HP:0002515 | 202 | Waddling gait |
| HP:0003691 | 202 | Scapular winging |
| HP:0003687 | 202 | Centrally nucleated skeletal muscle fibers |
| HP:0003704 | 202 | Scapuloperoneal weakness |
| HP:0003697 | 202 | Scapuloperoneal amyotrophy |
| HP:0003741 | 202 | Congenital muscular dystrophy |
| HP:0003707 | 202 | Calf muscle pseudohypertrophy |
| HP:0009055 | 202 | Generalized limb muscle atrophy |
| HP:0002792 | 202 | Reduced vital capacity |
| HP:0030046 | 202 | Hypoglycosylation of alpha-dystroglycan |

---


---


---

# Cluster \*203

| HPO | Cluster | Description |
| --- | --- | --- |
| HP:0000508 | 203 | Ptosis |
| HP:0003701 | 203 | Proximal muscle weakness |
| HP:0010628 | 203 | Facial palsy |
| HP:0003326 | 203 | Myalgia |
| HP:0003458 | 203 | EMG: myopathic abnormalities |
| HP:0003737 | 203 | Mitochondrial myopathy |
| HP:0003200 | 203 | Ragged-red muscle fibers |
| HP:0003201 | 203 | Rhabdomyolysis |
| HP:0003546 | 203 | Exercise intolerance |
| HP:0000590 | 203 | Progressive external ophthalmoplegia |
| HP:0003548 | 203 | Subsarcolemmal accumulations of abnormally shaped mitochondria |
| HP:0003688 | 203 | Cytochrome C oxidase-negative muscle fibers |
| HP:0003434 | 203 | Sensory ataxic neuropathy |
| HP:0001618 | 203 | Dysphonia |
| HP:0003388 | 203 | Easy fatigability |
| HP:0003390 | 203 | Sensory axonal neuropathy |
| HP:0003323 | 203 | Progressive muscle weakness |
| HP:0003713 | 203 | Muscle fiber necrosis |
| HP:0006886 | 203 | Impaired distal vibration sensation |
| HP:0002093 | 203 | Respiratory insufficiency |
| HP:0003306 | 203 | Spinal rigidity |

---


---


---

# Cluster \*204

| HPO | Cluster | Description |
| --- | --- | --- |
| HP:0009830 | 204 | Peripheral neuropathy |
| HP:0003581 | 204 | Adult onset |
| HP:0001288 | 204 | Gait disturbance |
| HP:0003677 | 204 | Slow progression |
| HP:0003693 | 204 | Distal amyotrophy |
| HP:0003701 | 204 | Proximal muscle weakness |
| HP:0010628 | 204 | Facial palsy |
| HP:0003376 | 204 | Steppage gait |
| HP:0003458 | 204 | EMG: myopathic abnormalities |
| HP:0003390 | 204 | Sensory axonal neuropathy |
| HP:0002355 | 204 | Difficulty walking |
| HP:0002600 | 204 | Hyporeflexia of lower limbs |
| HP:0009053 | 204 | Distal lower limb muscle weakness |
| HP:0003470 | 204 | Paralysis |
| HP:0002522 | 204 | Areflexia of lower limbs |
| HP:0003736 | 204 | Autophagic vacuoles |
| HP:0003555 | 204 | Muscle fiber splitting |
| HP:0009063 | 204 | Progressive distal muscle weakness |
| HP:0003715 | 204 | Myofibrillar myopathy |
| HP:0100303 | 204 | Muscle fiber cytoplasmatic inclusion bodies |
| HP:0003805 | 204 | Rimmed vacuoles |

---


---


---

# Cluster \*206

| HPO | Cluster | Description |
| --- | --- | --- |
| HP:0003581 | 206 | Adult onset |
| HP:0003693 | 206 | Distal amyotrophy |
| HP:0002460 | 206 | Distal muscle weakness |
| HP:0003805 | 206 | Rimmed vacuoles |
| HP:0003551 | 206 | Difficulty climbing stairs |
| HP:0040083 | 206 | Toe walking |
| HP:0003791 | 206 | Deposits immunoreactive to beta-amyloid protein |
| HP:0009072 | 206 | Decreased Achilles reflex |

---


---


---

# Cluster \*207

| HPO | Cluster | Description |
| --- | --- | --- |
| HP:0009830 | 207 | Peripheral neuropathy |
| HP:0003828 | 207 | Variable expressivity |
| HP:0001265 | 207 | Hyporeflexia |
| HP:0003477 | 207 | Peripheral axonal neuropathy |
| HP:0001761 | 207 | Pes cavus |
| HP:0003677 | 207 | Slow progression |
| HP:0001284 | 207 | Areflexia |
| HP:0002460 | 207 | Distal muscle weakness |
| HP:0009027 | 207 | Foot dorsiflexor weakness |
| HP:0003693 | 207 | Distal amyotrophy |
| HP:0003690 | 207 | Limb muscle weakness |
| HP:0003701 | 207 | Proximal muscle weakness |
| HP:0010628 | 207 | Facial palsy |
| HP:0001319 | 207 | Neonatal hypotonia |
| HP:0003798 | 207 | Nemaline bodies |
| HP:0003803 | 207 | Type 1 muscle fiber predominance |
| HP:0001765 | 207 | Hammertoe |
| HP:0002936 | 207 | Distal sensory impairment |
| HP:0003376 | 207 | Steppage gait |
| HP:0003380 | 207 | Decreased number of peripheral myelinated nerve fibers |
| HP:0003382 | 207 | Hypertrophic nerve changes |
| HP:0003383 | 207 | Onion bulb formation |
| HP:0003431 | 207 | Decreased motor nerve conduction velocity |
| HP:0003449 | 207 | Cold-induced muscle cramps |
| HP:0003621 | 207 | Juvenile onset |
| HP:0004336 | 207 | Myelin outfoldings |
| HP:0003378 | 207 | Axonal degeneration/regeneration |
| HP:0003384 | 207 | Peripheral axonal atrophy |
| HP:0003458 | 207 | EMG: myopathic abnormalities |
| HP:0003557 | 207 | Increased variability in muscle fiber diameter |
| HP:0002515 | 207 | Waddling gait |
| HP:0003691 | 207 | Scapular winging |
| HP:0002359 | 207 | Frequent falls |
| HP:0002355 | 207 | Difficulty walking |
| HP:0003547 | 207 | Shoulder girdle muscle weakness |
| HP:0008981 | 207 | Calf muscle hypertrophy |
| HP:0003749 | 207 | Pelvic girdle muscle weakness |
| HP:0003391 | 207 | Gowers sign |
| HP:0003687 | 207 | Centrally nucleated skeletal muscle fibers |
| HP:0000467 | 207 | Neck muscle weakness |
| HP:0003805 | 207 | Rimmed vacuoles |
| HP:0008180 | 207 | Mildly elevated creatine kinase |
| HP:0003306 | 207 | Spinal rigidity |
| HP:0003736 | 207 | Autophagic vacuoles |
| HP:0003551 | 207 | Difficulty climbing stairs |
| HP:0003555 | 207 | Muscle fiber splitting |
| HP:0003715 | 207 | Myofibrillar myopathy |
| HP:0003724 | 207 | Shoulder girdle muscle atrophy |

---


---


---

# Cluster \*208

| HPO | Cluster | Description |
| --- | --- | --- |
| HP:0003676 | 208 | Progressive |
| HP:0001265 | 208 | Hyporeflexia |
| HP:0001324 | 208 | Muscle weakness |
| HP:0003477 | 208 | Peripheral axonal neuropathy |
| HP:0001288 | 208 | Gait disturbance |
| HP:0002015 | 208 | Dysphagia |
| HP:0003693 | 208 | Distal amyotrophy |
| HP:0001284 | 208 | Areflexia |
| HP:0002460 | 208 | Distal muscle weakness |
| HP:0002936 | 208 | Distal sensory impairment |
| HP:0003383 | 208 | Onion bulb formation |
| HP:0011096 | 208 | Peripheral demyelination |
| HP:0003557 | 208 | Increased variability in muscle fiber diameter |
| HP:0003388 | 208 | Easy fatigability |
| HP:0000762 | 208 | Decreased nerve conduction velocity |
| HP:0007269 | 208 | Spinal muscular atrophy |
| HP:0003700 | 208 | Generalized amyotrophy |
| HP:0003447 | 208 | Axonal loss |
| HP:0007182 | 208 | Peripheral hypomyelination |
| HP:0007141 | 208 | Sensorimotor neuropathy |
| HP:0006829 | 208 | Severe muscular hypotonia |

---


---


---

# Cluster \*209

| HPO | Cluster | Description |
| --- | --- | --- |
| HP:0001265 | 209 | Hyporeflexia |
| HP:0003477 | 209 | Peripheral axonal neuropathy |
| HP:0001761 | 209 | Pes cavus |
| HP:0003693 | 209 | Distal amyotrophy |
| HP:0001178 | 209 | Ulnar claw |
| HP:0001284 | 209 | Areflexia |
| HP:0001765 | 209 | Hammertoe |
| HP:0002460 | 209 | Distal muscle weakness |
| HP:0002936 | 209 | Distal sensory impairment |
| HP:0003376 | 209 | Steppage gait |
| HP:0003383 | 209 | Onion bulb formation |
| HP:0003431 | 209 | Decreased motor nerve conduction velocity |
| HP:0006886 | 209 | Impaired distal vibration sensation |
| HP:0007267 | 209 | Chronic axonal neuropathy |
| HP:0003444 | 209 | EMG: chronic denervation signs |
| HP:0006937 | 209 | Impaired distal tactile sensation |

---


---


---

# Cluster \*210

| HPO | Cluster | Description |
| --- | --- | --- |
| HP:0001252 | 210 | Muscular hypotonia |
| HP:0008872 | 210 | Feeding difficulties in infancy |
| HP:0003577 | 210 | Congenital onset |
| HP:0002803 | 210 | Congenital contracture |
| HP:0007941 | 210 | Limited extraocular movements |
| HP:0200147 | 210 | Neuronal loss in basal ganglia |
| HP:0006999 | 210 | Basal ganglia gliosis |

---


---


---

# Cluster \*212

| HPO | Cluster | Description |
| --- | --- | --- |
| HP:0001638 | 212 | Cardiomyopathy |
| HP:0003202 | 212 | Skeletal muscle atrophy |
| HP:0003676 | 212 | Progressive |
| HP:0001271 | 212 | Polyneuropathy |
| HP:0002922 | 212 | Increased CSF protein |
| HP:0001265 | 212 | Hyporeflexia |
| HP:0001324 | 212 | Muscle weakness |
| HP:0003477 | 212 | Peripheral axonal neuropathy |
| HP:0003470 | 212 | Paralysis |
| HP:0011675 | 212 | Arrhythmia |
| HP:0003693 | 212 | Distal amyotrophy |
| HP:0001284 | 212 | Areflexia |
| HP:0002936 | 212 | Distal sensory impairment |
| HP:0003383 | 212 | Onion bulb formation |
| HP:0003431 | 212 | Decreased motor nerve conduction velocity |
| HP:0001349 | 212 | Facial diplegia |
| HP:0000762 | 212 | Decreased nerve conduction velocity |
| HP:0002600 | 212 | Hyporeflexia of lower limbs |
| HP:0003448 | 212 | Decreased sensory nerve conduction velocity |
| HP:0001771 | 212 | Achilles tendon contracture |
| HP:0009063 | 212 | Progressive distal muscle weakness |
| HP:0002490 | 212 | Increased CSF lactate |
| HP:0007141 | 212 | Sensorimotor neuropathy |
| HP:0003715 | 212 | Myofibrillar myopathy |

---


---


---

# Cluster \*213

| HPO | Cluster | Description |
| --- | --- | --- |
| HP:0000970 | 213 | Anhidrosis |
| HP:0007460 | 213 | Autoamputation of digits |
| HP:0002661 | 213 | Painless fractures due to injury |
| HP:0008000 | 213 | Decreased corneal reflex |
| HP:0003448 | 213 | Decreased sensory nerve conduction velocity |
| HP:0000224 | 213 | Decreased taste sensation |
| HP:0001069 | 213 | Episodic hyperhidrosis |
| HP:0006121 | 213 | Acral ulceration |
| HP:0003115 | 213 | Abnormal EKG |

---


---


---

# Cluster \*214

| HPO | Cluster | Description |
| --- | --- | --- |
| HP:0002650 | 214 | Scoliosis |
| HP:0003677 | 214 | Slow progression |
| HP:0003693 | 214 | Distal amyotrophy |
| HP:0010628 | 214 | Facial palsy |
| HP:0001284 | 214 | Areflexia |
| HP:0002460 | 214 | Distal muscle weakness |
| HP:0009027 | 214 | Foot dorsiflexor weakness |
| HP:0003391 | 214 | Gowers sign |
| HP:0008955 | 214 | Progressive distal muscular atrophy |
| HP:0009113 | 214 | Diaphragmatic weakness |
| HP:0011349 | 214 | Abducens palsy |
| HP:0009130 | 214 | Hand muscle atrophy |
| HP:0012246 | 214 | Oculomotor nerve palsy |

---


---


---

# Cluster \*215

| HPO | Cluster | Description |
| --- | --- | --- |
| HP:0000544 | 215 | External ophthalmoplegia |
| HP:0003198 | 215 | Myopathy |
| HP:0003701 | 215 | Proximal muscle weakness |
| HP:0010628 | 215 | Facial palsy |
| HP:0003326 | 215 | Myalgia |
| HP:0003458 | 215 | EMG: myopathic abnormalities |
| HP:0003560 | 215 | Muscular dystrophy |
| HP:0003710 | 215 | Exercise-induced muscle cramps |
| HP:0003201 | 215 | Rhabdomyolysis |
| HP:0003546 | 215 | Exercise intolerance |
| HP:0003557 | 215 | Increased variability in muscle fiber diameter |
| HP:0002913 | 215 | Myoglobinuria |
| HP:0009046 | 215 | Difficulty running |
| HP:0003738 | 215 | Exercise-induced myalgia |
| HP:0008988 | 215 | Pelvic girdle muscle atrophy |
| HP:0003707 | 215 | Calf muscle pseudohypertrophy |
| HP:0003731 | 215 | Quadriceps muscle weakness |
| HP:0003327 | 215 | Axial muscle weakness |
| HP:0003787 | 215 | Type 1 and type 2 muscle fiber minicore regions |

---


---


---

# Cluster \*216

| HPO | Cluster | Description |
| --- | --- | --- |
| HP:0002522 | 216 | Areflexia of lower limbs |
| HP:0009053 | 216 | Distal lower limb muscle weakness |
| HP:0008944 | 216 | Distal lower limb amyotrophy |
| HP:0008948 | 216 | Proximal upper limb amyotrophy |
| HP:0008994 | 216 | Proximal muscle weakness in lower limbs |
| HP:0003791 | 216 | Deposits immunoreactive to beta-amyloid protein |
| HP:0007149 | 216 | Distal upper limb amyotrophy |

---


---


---

# Cluster \*217

| HPO | Cluster | Description |
| --- | --- | --- |
| HP:0003477 | 217 | Peripheral axonal neuropathy |
| HP:0000970 | 217 | Anhidrosis |
| HP:0002754 | 217 | Osteomyelitis |
| HP:0003621 | 217 | Juvenile onset |
| HP:0006984 | 217 | Distal sensory loss of all modalities |
| HP:0007141 | 217 | Sensorimotor neuropathy |
| HP:0006121 | 217 | Acral ulceration |
| HP:0001218 | 217 | Autoamputation |

---


---


---

# Cluster \*220

| HPO | Cluster | Description |
| --- | --- | --- |
| HP:0000007 | 220 | Autosomal recessive inheritance |
| HP:0001762 | 220 | Talipes equinovarus |
| HP:0001302 | 220 | Pachygyria |
| HP:0000557 | 220 | Buphthalmos |
| HP:0002650 | 220 | Scoliosis |
| HP:0000518 | 220 | Cataract |
| HP:0001188 | 220 | Hand clenching |
| HP:0002804 | 220 | Arthrogryposis multiplex congenita |
| HP:0003577 | 220 | Congenital onset |
| HP:0001371 | 220 | Flexion contracture |
| HP:0001321 | 220 | Cerebellar hypoplasia |
| HP:0002365 | 220 | Hypoplasia of the brainstem |
| HP:0007973 | 220 | Retinal dysplasia |
| HP:0012110 | 220 | Hypoplasia of the pons |
| HP:0007033 | 220 | Cerebellar dysplasia |
| HP:0007260 | 220 | Type II lissencephaly |
| HP:0031882 | 220 | Agyria |
| HP:0002350 | 220 | Cerebellar cyst |
| HP:0002803 | 220 | Congenital contracture |
| HP:0011968 | 220 | Feeding difficulties |
| HP:0002093 | 220 | Respiratory insufficiency |
| HP:0006829 | 220 | Severe muscular hypotonia |
| HP:0030046 | 220 | Hypoglycosylation of alpha-dystroglycan |

---


---


---

# Cluster \*221

| HPO | Cluster | Description |
| --- | --- | --- |
| HP:0001270 | 221 | Motor delay |
| HP:0003828 | 221 | Variable expressivity |
| HP:0003701 | 221 | Proximal muscle weakness |
| HP:0010628 | 221 | Facial palsy |
| HP:0001319 | 221 | Neonatal hypotonia |
| HP:0001371 | 221 | Flexion contracture |
| HP:0003324 | 221 | Generalized muscle weakness |
| HP:0003798 | 221 | Nemaline bodies |
| HP:0003803 | 221 | Type 1 muscle fiber predominance |
| HP:0003458 | 221 | EMG: myopathic abnormalities |
| HP:0003557 | 221 | Increased variability in muscle fiber diameter |
| HP:0002515 | 221 | Waddling gait |
| HP:0003388 | 221 | Easy fatigability |
| HP:0003691 | 221 | Scapular winging |
| HP:0002355 | 221 | Difficulty walking |
| HP:0008981 | 221 | Calf muscle hypertrophy |
| HP:0000467 | 221 | Neck muscle weakness |
| HP:0003805 | 221 | Rimmed vacuoles |
| HP:0002058 | 221 | Myopathic facies |
| HP:0003306 | 221 | Spinal rigidity |
| HP:0003551 | 221 | Difficulty climbing stairs |
| HP:0003810 | 221 | Late-onset distal muscle weakness |
| HP:0003201 | 221 | Rhabdomyolysis |
| HP:0003200 | 221 | Ragged-red muscle fibers |
| HP:0003390 | 221 | Sensory axonal neuropathy |
| HP:0000590 | 221 | Progressive external ophthalmoplegia |
| HP:0003548 | 221 | Subsarcolemmal accumulations of abnormally shaped mitochondria |
| HP:0003688 | 221 | Cytochrome C oxidase-negative muscle fibers |
| HP:0003713 | 221 | Muscle fiber necrosis |
| HP:0006886 | 221 | Impaired distal vibration sensation |
| HP:0003391 | 221 | Gowers sign |
| HP:0000597 | 221 | Ophthalmoparesis |
| HP:0003722 | 221 | Neck flexor weakness |

---


---


---

# Cluster \*222

| HPO | Cluster | Description |
| --- | --- | --- |
| HP:0002460 | 222 | Distal muscle weakness |
| HP:0002936 | 222 | Distal sensory impairment |
| HP:0003621 | 222 | Juvenile onset |
| HP:0001760 | 222 | Abnormality of the foot |
| HP:0001155 | 222 | Abnormality of the hand |
| HP:0006984 | 222 | Distal sensory loss of all modalities |
| HP:0003450 | 222 | Axonal regeneration |

---


---


---

# Cluster \*223

| HPO | Cluster | Description |
| --- | --- | --- |
| HP:0000007 | 223 | Autosomal recessive inheritance |
| HP:0001638 | 223 | Cardiomyopathy |
| HP:0003593 | 223 | Infantile onset |
| HP:0001324 | 223 | Muscle weakness |
| HP:0011675 | 223 | Arrhythmia |
| HP:0001639 | 223 | Hypertrophic cardiomyopathy |
| HP:0001414 | 223 | Microvesicular hepatic steatosis |
| HP:0003546 | 223 | Exercise intolerance |
| HP:0003323 | 223 | Progressive muscle weakness |
| HP:0001924 | 223 | Sideroblastic anemia |
| HP:0008322 | 223 | Abnormal mitochondrial morphology |

---


---


---

# Cluster \*224

| HPO | Cluster | Description |
| --- | --- | --- |
| HP:0001638 | 224 | Cardiomyopathy |
| HP:0003677 | 224 | Slow progression |
| HP:0003701 | 224 | Proximal muscle weakness |
| HP:0010628 | 224 | Facial palsy |
| HP:0003798 | 224 | Nemaline bodies |
| HP:0003458 | 224 | EMG: myopathic abnormalities |
| HP:0002515 | 224 | Waddling gait |
| HP:0003323 | 224 | Progressive muscle weakness |
| HP:0003736 | 224 | Autophagic vacuoles |
| HP:0003551 | 224 | Difficulty climbing stairs |
| HP:0001771 | 224 | Achilles tendon contracture |
| HP:0003555 | 224 | Muscle fiber splitting |
| HP:0009063 | 224 | Progressive distal muscle weakness |
| HP:0002877 | 224 | Nocturnal hypoventilation |
| HP:0032341 | 224 | Reduced forced vital capacity |
| HP:0003715 | 224 | Myofibrillar myopathy |
| HP:0100303 | 224 | Muscle fiber cytoplasmatic inclusion bodies |

---


---


---

# Cluster \*225

| HPO | Cluster | Description |
| --- | --- | --- |
| HP:0003394 | 225 | Muscle spasm |
| HP:0003380 | 225 | Decreased number of peripheral myelinated nerve fibers |
| HP:0003621 | 225 | Juvenile onset |
| HP:0002715 | 225 | Abnormality of the immune system |
| HP:0003201 | 225 | Rhabdomyolysis |
| HP:0002913 | 225 | Myoglobinuria |
| HP:0007126 | 225 | Proximal amyotrophy |

---


---


---

# Cluster \*227

| HPO | Cluster | Description |
| --- | --- | --- |
| HP:0003701 | 227 | Proximal muscle weakness |
| HP:0010628 | 227 | Facial palsy |
| HP:0003324 | 227 | Generalized muscle weakness |
| HP:0003621 | 227 | Juvenile onset |
| HP:0003458 | 227 | EMG: myopathic abnormalities |
| HP:0003691 | 227 | Scapular winging |
| HP:0001604 | 227 | Vocal cord paresis |
| HP:0002111 | 227 | Restrictive deficit on pulmonary function testing |
| HP:0003547 | 227 | Shoulder girdle muscle weakness |
| HP:0003724 | 227 | Shoulder girdle muscle atrophy |
| HP:0008981 | 227 | Calf muscle hypertrophy |
| HP:0003749 | 227 | Pelvic girdle muscle weakness |
| HP:0007126 | 227 | Proximal amyotrophy |
| HP:0003551 | 227 | Difficulty climbing stairs |
| HP:0003704 | 227 | Scapuloperoneal weakness |
| HP:0011349 | 227 | Abducens palsy |
| HP:0009130 | 227 | Hand muscle atrophy |
| HP:0012246 | 227 | Oculomotor nerve palsy |
| HP:0007230 | 227 | Decreased distal sensory nerve action potential |
| HP:0003733 | 227 | Thigh hypertrophy |

---


---


---

# Cluster \*229

| HPO | Cluster | Description |
| --- | --- | --- |
| HP:0001283 | 229 | Bulbar palsy |
| HP:0001265 | 229 | Hyporeflexia |
| HP:0003701 | 229 | Proximal muscle weakness |
| HP:0003307 | 229 | Hyperlordosis |
| HP:0010628 | 229 | Facial palsy |
| HP:0001371 | 229 | Flexion contracture |
| HP:0003798 | 229 | Nemaline bodies |
| HP:0003803 | 229 | Type 1 muscle fiber predominance |
| HP:0001284 | 229 | Areflexia |
| HP:0009027 | 229 | Foot dorsiflexor weakness |
| HP:0003458 | 229 | EMG: myopathic abnormalities |
| HP:0003200 | 229 | Ragged-red muscle fibers |
| HP:0003557 | 229 | Increased variability in muscle fiber diameter |
| HP:0002515 | 229 | Waddling gait |
| HP:0003691 | 229 | Scapular winging |
| HP:0002747 | 229 | Respiratory insufficiency due to muscle weakness |
| HP:0003547 | 229 | Shoulder girdle muscle weakness |
| HP:0008981 | 229 | Calf muscle hypertrophy |
| HP:0003749 | 229 | Pelvic girdle muscle weakness |
| HP:0003391 | 229 | Gowers sign |
| HP:0003805 | 229 | Rimmed vacuoles |
| HP:0003551 | 229 | Difficulty climbing stairs |
| HP:0003555 | 229 | Muscle fiber splitting |
| HP:0003306 | 229 | Spinal rigidity |
| HP:0003722 | 229 | Neck flexor weakness |
| HP:0003810 | 229 | Late-onset distal muscle weakness |
| HP:0008180 | 229 | Mildly elevated creatine kinase |

---


---


---

# Cluster \*230

| HPO | Cluster | Description |
| --- | --- | --- |
| HP:0001265 | 230 | Hyporeflexia |
| HP:0001761 | 230 | Pes cavus |
| HP:0003376 | 230 | Steppage gait |
| HP:0003384 | 230 | Peripheral axonal atrophy |
| HP:0011463 | 230 | Childhood onset |
| HP:0003474 | 230 | Sensory impairment |
| HP:0008956 | 230 | Proximal lower limb amyotrophy |
| HP:0011808 | 230 | Decreased patellar reflex |
| HP:0001868 | 230 | Autoamputation of foot |
| HP:0001886 | 230 | Foot osteomyelitis |
| HP:0012211 | 230 | Abnormal renal physiology |
| HP:0007141 | 230 | Sensorimotor neuropathy |
| HP:0030237 | 230 | Hand muscle weakness |

---


---


---

# Cluster \*231

| HPO | Cluster | Description |
| --- | --- | --- |
| HP:0003202 | 231 | Skeletal muscle atrophy |
| HP:0001265 | 231 | Hyporeflexia |
| HP:0001761 | 231 | Pes cavus |
| HP:0003693 | 231 | Distal amyotrophy |
| HP:0001284 | 231 | Areflexia |
| HP:0001765 | 231 | Hammertoe |
| HP:0002460 | 231 | Distal muscle weakness |
| HP:0002936 | 231 | Distal sensory impairment |
| HP:0003431 | 231 | Decreased motor nerve conduction velocity |
| HP:0003378 | 231 | Axonal degeneration/regeneration |
| HP:0003448 | 231 | Decreased sensory nerve conduction velocity |
| HP:0001604 | 231 | Vocal cord paresis |
| HP:0003387 | 231 | Decreased number of large peripheral myelinated nerve fibers |
