# Supplementary material for: Decoding Neuromuscular Disorders Using Phenotypic Clusters Obtained From Co-Occurrence Networks
Source: Front Mol Biosci. 2021 Apr 19;8:635074. doi: 10.3389/fmolb.2021.635074 (PMC8147726; doi:10.3389/fmolb.2021.635074)
Supplement: Supplementary file 1 [file DataSheet1.zip › Sup2-orphanet_report.html]

orphanet\_report\_template.utf8


# Clusters Orphanet Neuromuscular Diseases Study

We performed a phenotypic comorbidity analysis of neuromuscular diseases in Orphanet to elucidate molecular mechanisms underlying these phenotypes.

In the following sections we will be describing each step and the results genereted.

## Step one: Datasets of NMDs, genes, phenotypes and typical NMD phenotypes

Orphanet have an ontology structure. we found an ontology related to neuromuscular diseases, **Orphanet:183497 Genetic neuromuscular** disease. We extract all child terms related to Orphanet:183497 and all of the diseases asociated with this term.

Once we have the list of neuromuscular diseases, we coun the frequency of phenotypes in neuromuscular and non neuromuscular diseases. With these data, we can perform a fisher test analysis to find those phenotypes which are more represented in neuromusculare diseases rather than in non neuromuscular diseases. We have the following results:

**Table 1**: Preprocesing data summary.

| Metric | Value |
| --- | --- |
| Total\_Orphanet\_diseases\_in\_HPO | 2020 |
| Total\_Orphanet\_phenotypes | 5430 |
| Total\_Orphanet\_genes | 2730 |
| Total\_Orphanet\_NMD | 227 |
| Total\_Orphanet\_NMD\_in\_HPO | 126 |
| Total\_Orphanet\_neuromuscular\_phenotypes | 216 |

## Step 2: Bipartite network to obtain phenotype-phenotype associations

**Table 2:** Bipartite results general summary.

| Metric | Value |
| --- | --- |
| Total\_phenotype\_pairs | 467899 |
| Significant\_phenotype\_pairs | 99193 |

In the following plot we can see the number of pairs which form the subnetwork with a HyI >= 2.

**Figure 1:** Number of phenotype pairs with a HyI >= 2.

**Table 3:** Number of phenotype pairs with a HyI >= 2.

| Type | Pairs Number |
| --- | --- |
| NMDs | 2314 ± NA |
| non\_NMDs | 173.23 ± 16.43 |

### Validating phenotype-phenotye associations by PubMed co-mention

**Figure 2** Comention pvalue density plot.

**Table 4** Number of co-occurrent pairs.

| Type | Pairs Number |
| --- | --- |
| NMDs | 689 ± NA |
| non\_NMDs | 110.4 ± 13.469 |

## Step 3: Tripartite network to obtain phenotype-function pairs

In this step we will get phenotype-genes pairs. Through a functional assignment of these genes we can get phenotype-function pairs.

**Table 5** Tripartite results general summary.

| Metric | Value |
| --- | --- |
| Number\_of\_genes | 2730 |
| Number\_of\_phen2gene\_pairs\_HyI\_2 | 36218 |
| Number\_of\_genes\_HyI\_2 | 2700 |
| NMD\_genes\_HyI\_2 | 611 |
| Total\_kegg\_terms\_0.05 | 209 |
| Total\_go\_terms\_0.05 | 6281 |
| Total\_reactome\_terms\_0.05 | 1356 |
| NMD\_kegg\_0.05 | 123 |
| NMD\_go\_0.05 | 3618 |
| NMD\_reactome\_0.05 | 631 |

### Validating phenotype-function associations by PubMed co-mention

#### KEGG

**Figure 3** Significant phenotype-KEGG pairs.

**Table 6** Significant phenotype-KEGG pairs.

| Type | Co-occurrent pairs | No co-occurrent pairs |
| --- | --- | --- |
| phen2kegg\_orpha | 1556 ± NA | 15079 ± NA |
| rdm\_phen2kegg\_orpha | 627.525 ± 21.182 | 16007.475 ± 21.182 |

#### GO

**Figure 4** Significant phenotype-GO pairs.

**Table 7** Significant phenotype-GO pairs.

| Type | Co-occurrent pairs | No co-occurrent pairs |
| --- | --- | --- |
| phen2go\_orpha | 21863 ± NA | 491681 ± NA |
| rdm\_phen2go\_orpha | 9612.99 ± 93.489 | 503921.97 ± 93.366 |

##### Reactome

**Figure 5** Significant phenotype-Reactome pairs.

**Table 8** Significant phenotype-Reactome pairs.

| Type | Co-occurrent pairs | No co-occurrent pairs |
| --- | --- | --- |
| phen2reactome\_orpha | 2402 ± NA | 71659 ± NA |
| rdm\_phen2reactome\_orpha | 816.1 ± 28.712 | 73218.65 ± 29.929 |

## Step 4: Cluster Analysis

With linkcomm R package we obtain a number of clusters for each network. In the following plots we show some results of the clustering process. By one hand we have measured the number of clusters obtained and the average number of nodes of each cluster.

**Figure 6** Cluster analysis summary.

**Table 9** Cluster analysis summary.

| Type | Cluster Number | Average Cluster size |
| --- | --- | --- |
| NMDs | 150 ± NA | 13.627 ± NA |
| non\_NMDs | 23.85 ± 4.293 | 4.146 ± 0.278 |

### Functionally-coherent clusters of phenotypes in NMDs

**Figure 7** Functional coherent clusters summary.

**Table 10** Functional coherent clusters summary.

| Type | Cluster with 70 coherence\_0.05 | Cluster with 50 coherence\_0.05 |
| --- | --- | --- |
| NMDs | 72 ± NA | 132 ± NA |
| non\_NMDs | 14.51 ± 3.189 | 21.99 ± 4.034 |

## Clusters Details

In this section we include Orphanet neuromuscular cluster’s details.

---


---


---

# Cluster #1

| HPO | Cluster | Description |
| --- | --- | --- |
| HP:0030196 | 1 | Fatigable weakness of respiratory muscles |
| HP:0002203 | 1 | Respiratory paralysis |
| HP:0003694 | 1 | Late-onset proximal muscle weakness |
| HP:0003752 | 1 | Episodic flaccid weakness |
| HP:0012726 | 1 | Episodic hypokalemia |

| Cluster | Cell function | Description | Phenotype\_coverage | Genes |
| --- | --- | --- | --- | --- |
| 1 | GO:0071313 | cellular response to caffeine | 100 | PPARGC1A, CACNA1S |
| 1 | GO:0060078 | regulation of postsynaptic membrane potential | 100 | CHRNE, CHRND, CHRNA1, CHRNB1, GABRA3 |
| 1 | GO:0098911 | regulation of ventricular cardiac muscle cell action potential | 80 | KCNE3 |
| 1 | GO:0051932 | synaptic transmission, GABAergic | 80 | GABRA3 |
| 1 | GO:0010107 | potassium ion import | 80 | KCNJ18 |
| 1 | GO:1905031 | regulation of membrane repolarization during cardiac muscle cell action potential | 80 | KCNE3 |
| 1 | GO:0045822 | negative regulation of heart contraction | 80 | KCNE3 |
| 1 | GO:1990573 | potassium ion import across plasma membrane | 80 | KCNJ18 |
| 1 | GO:0086091 | regulation of heart rate by cardiac conduction | 80 | KCNE3 |
| 1 | GO:0045759 | negative regulation of action potential | 80 | KCNE3 |
| 1 | GO:0019226 | transmission of nerve impulse | 80 | SOD1, SCN4A, CHRNA1 |
| 1 | GO:0071435 | potassium ion export | 80 | KCNE3 |
| 1 | GO:0098915 | membrane repolarization during ventricular cardiac muscle cell action potential | 80 | KCNE3 |
| 1 | GO:1901387 | positive regulation of voltage-gated calcium channel activity | 80 | KCNE3 |
| 1 | GO:0003009 | skeletal muscle contraction | 80 | CHRNB1, SCN4A, CHRND, CHRNA1 |
| 1 | GO:0007214 | gamma-aminobutyric acid signaling pathway | 80 | GABRA3 |
| 1 | GO:0097623 | potassium ion export across plasma membrane | 80 | KCNE3 |
| 1 | GO:0070509 | calcium ion import | 80 | CACNA1S |
| 1 | GO:1902476 | chloride transmembrane transport | 80 | GABRA3 |
| 1 | GO:1902260 | negative regulation of delayed rectifier potassium channel activity | 80 | KCNE3 |
| 1 | GO:0060307 | regulation of ventricular cardiac muscle cell membrane repolarization | 80 | KCNE3 |
| 1 | GO:0071804 | cellular potassium ion transport | 80 | KCNE3, KCNJ18 |
| 1 | GO:0045988 | negative regulation of striated muscle contraction | 80 | KCNE3 |
| 1 | R-HSA-5576891 | Cardiac conduction | 80 | CACNA1S, SCN4A, KCNE3 |
| 1 | R-HSA-397014 | Muscle contraction | 80 | CACNA1S, SCN4A, KCNE3 |
| 1 | R-HSA-977443 | GABA receptor activation | 80 | GABRA3 |
| 1 | R-HSA-375165 | NCAM signaling for neurite out-growth | 80 | CACNA1S |
| 1 | R-HSA-5576893 | Phase 2 - plateau phase | 80 | CACNA1S, KCNE3 |
| 1 | R-HSA-5576892 | Phase 0 - rapid depolarisation | 80 | CACNA1S, SCN4A |
| 1 | R-HSA-419037 | NCAM1 interactions | 80 | CACNA1S |

---


---


---

# Cluster #2

| HPO | Cluster | Description |
| --- | --- | --- |
| HP:0002515 | 2 | Waddling gait |
| HP:0008956 | 2 | Proximal lower limb amyotrophy |
| HP:0008994 | 2 | Proximal muscle weakness in lower limbs |
| HP:0003236 | 2 | Elevated serum creatine kinase |
| HP:0001644 | 2 | Dilated cardiomyopathy |
| HP:0003306 | 2 | Spinal rigidity |
| HP:0003691 | 2 | Scapular winging |
| HP:0003458 | 2 | EMG: myopathic abnormalities |
| HP:0008997 | 2 | Proximal muscle weakness in upper limbs |
| HP:0003805 | 2 | Rimmed vacuoles |
| HP:0002987 | 2 | Elbow flexion contracture |
| HP:0011807 | 2 | Type 1 muscle fiber atrophy |
| HP:0006785 | 2 | Limb-girdle muscular dystrophy |
| HP:0008948 | 2 | Proximal upper limb amyotrophy |
| HP:0001771 | 2 | Achilles tendon contracture |
| HP:0004631 | 2 | Decreased cervical spine flexion due to contractures of posterior cervical muscles |
| HP:0001678 | 2 | Atrioventricular block |

| Cluster | Cell function | Description | Phenotype\_coverage | Genes |
| --- | --- | --- | --- | --- |
| 2 | hsa05410 | Hypertrophic cardiomyopathy (HCM) | 100.00000 | SGCD, SGCG, DAG1, MYL2, TTN, TPM3, TPM2, EMD, ITGA7, SGCA, DMD, LMNA, MYH7 |
| 2 | hsa05414 | Dilated cardiomyopathy | 100.00000 | SGCD, SGCG, DAG1, MYL2, TTN, TPM3, TPM2, EMD, ITGA7, SGCA, DMD, LMNA, MYH7 |
| 2 | GO:0007084 | mitotic nuclear envelope reassembly | 82.35294 | EMD, LMNA |
| 2 | hsa05412 | Arrhythmogenic right ventricular cardiomyopathy (ARVC) | 76.47059 | SGCD, SGCG, DAG1, EMD, ITGA7, SGCA, LMNA |
| 2 | GO:0007517 | muscle organ development | 76.47059 | MYF6, KLHL41, NEB, SGCD, CAPN3, SELENON, TTN, EMD, FKTN, FHL1, DMD, CFL2, ITGA7, RYR1, MYH7, SGCA, FOS, ACTA1, MYL2, SGCG, LARGE1, TAZ |
| 2 | R-HSA-2995410 | Nuclear Envelope Reassembly | 76.47059 | LMNA, EMD |
| 2 | R-HSA-4419969 | Depolymerisation of the Nuclear Lamina | 76.47059 | LMNA, EMD |
| 2 | R-HSA-2995383 | Initiation of Nuclear Envelope Reformation | 76.47059 | LMNA, EMD |
| 2 | GO:0003012 | muscle system process | 70.58824 | NEB, SGCD, MYOT, ACTA1, TTN, DMD, MYL2, TPM2, SCN4A, LMNA, EMD, KLHL41, RYR1, MYH7, SGCA, PABPN1, TAZ, TPM3 |
| 2 | GO:0014706 | striated muscle tissue development | 70.58824 | NEB, SGCD, ACVR1, SELENON, ACTA1, CFL2, TTN, MYL2, LMNA, MYF6, SGCG, EMD, KLHL41, RYR1, MYH7, TAZ, FOS |
| 2 | R-HSA-2980766 | Nuclear Envelope Breakdown | 70.58824 | LMNA, EMD |

---


---


---

# Cluster #3

| HPO | Cluster | Description |
| --- | --- | --- |
| HP:0002515 | 3 | Waddling gait |
| HP:0008981 | 3 | Calf muscle hypertrophy |
| HP:0001349 | 3 | Facial diplegia |
| HP:0003691 | 3 | Scapular winging |
| HP:0003803 | 3 | Type 1 muscle fiber predominance |
| HP:0002747 | 3 | Respiratory insufficiency due to muscle weakness |
| HP:0002792 | 3 | Reduced vital capacity |

| Cluster | Cell function | Description | Phenotype\_coverage | Genes |
| --- | --- | --- | --- | --- |
| 3 | R-HSA-445355 | Smooth Muscle Contraction | 100.00000 | TPM3, TPM2, DYSF |
| 3 | hsa05410 | Hypertrophic cardiomyopathy (HCM) | 85.71429 | MYL2, TTN, TPM3, TPM2, EMD, ITGA7, LMNA |
| 3 | hsa05414 | Dilated cardiomyopathy | 85.71429 | MYL2, TTN, TPM3, TPM2, EMD, ITGA7, LMNA |
| 3 | GO:0030049 | muscle filament sliding | 85.71429 | NEB, TNNT1, ACTA1, TTN, MYL2, TPM2, TPM3 |
| 3 | GO:0030239 | myofibril assembly | 85.71429 | NEB, TNNT1, ACTA1, TTN, CFL2, MYL2, LMOD3, KLHL41 |
| 3 | R-HSA-397014 | Muscle contraction | 85.71429 | TTN, TPM2, RYR1, ACTA1, TPM3, TNNT1, NEB, MYL2 |
| 3 | R-HSA-390522 | Striated Muscle Contraction | 85.71429 | TTN, TPM2, ACTA1, TPM3, TNNT1, NEB, MYL2 |
| 3 | hsa04260 | Cardiac muscle contraction | 71.42857 | MYL2, TPM3, TPM2 |
| 3 | GO:0048741 | skeletal muscle fiber development | 71.42857 | SELENON, ACTA1, MYF6, LMOD3, KLHL41, RYR1, KLHL40 |

---


---


---

# Cluster #7

| HPO | Cluster | Description |
| --- | --- | --- |
| HP:0009027 | 7 | Foot dorsiflexor weakness |
| HP:0001290 | 7 | Generalized hypotonia |
| HP:0002515 | 7 | Waddling gait |
| HP:0009046 | 7 | Difficulty running |
| HP:0003557 | 7 | Increased variability in muscle fiber diameter |
| HP:0030200 | 7 | Fatiguable weakness of proximal limb muscles |

| Cluster | Cell function | Description | Phenotype\_coverage | Genes |
| --- | --- | --- | --- | --- |
| 7 | GO:0007517 | muscle organ development | 100.00000 | NEB, SELENON, TTN, ACTA1, SPEG, CFL2, CASQ1, SGCG, KLHL41, LMOD3, RYR1, MYH7 |
| 7 | GO:0048747 | muscle fiber development | 100.00000 | NEB, SELENON, TTN, ACTA1, KLHL41, LMOD3, RYR1, FLNC |
| 7 | GO:0014706 | striated muscle tissue development | 83.33333 | NEB, SELENON, TTN, ACTA1, CFL2, CASQ1, LMNA, KLHL41, LMOD3, RYR1, MYH7 |
| 7 | GO:0006936 | muscle contraction | 83.33333 | NEB, TTN, ACTA1, TPM2, CASQ1, BIN1, KLHL41, LMOD3, RYR1, MYH7, TPM3 |
| 7 | GO:0030239 | myofibril assembly | 83.33333 | NEB, TTN, ACTA1, CFL2, MYPN, CASQ1, KLHL41, LMOD3 |

---


---


---

# Cluster #25

| HPO | Cluster | Description |
| --- | --- | --- |
| HP:0002015 | 25 | Dysphagia |
| HP:0001644 | 25 | Dilated cardiomyopathy |
| HP:0003200 | 25 | Ragged-red muscle fibers |
| HP:0002578 | 25 | Gastroparesis |
| HP:0003737 | 25 | Mitochondrial myopathy |

| Cluster | Cell function | Description | Phenotype\_coverage | Genes |
| --- | --- | --- | --- | --- |
| 25 | GO:0006264 | mitochondrial DNA replication | 100 | TWNK, DNA2, POLG2, RRM2B, MGME1, POLG |
| 25 | GO:0032042 | mitochondrial DNA metabolic process | 100 | TWNK, DNA2, POLG2, RRM2B, MGME1, POLG |
| 25 | GO:0046034 | ATP metabolic process | 80 | COX2, COX3, ND6, NDUFA11, ND3, NDUFV1, NDUFAF1, PINK1, ND2, ATP7A, NDUFS1, NDUFB10, SNCA, NDUFS6, NDUFA6, NDUFS3, ND1, NDUFB3, NDUFS7, NDUFS4, MYH7, NDUFV2, ATP8, NDUFB11, ND4, PARK7, NDUFS8, NDUFB9, TAZ, ND5, COX1, NDUFA1 |
| 25 | GO:0009167 | purine ribonucleoside monophosphate metabolic process | 80 | COX2, COX3, ND6, NDUFA11, ND3, NDUFV1, NDUFAF1, PINK1, ND2, ATP7A, NDUFS1, NDUFB10, SNCA, NDUFS6, NDUFA6, NDUFS3, ND1, NDUFB3, NDUFS7, NDUFS4, MYH7, NDUFV2, ATP8, NDUFB11, ND4, PARK7, NDUFS8, NDUFB9, TAZ, ND5, COX1, NDUFA1 |
| 25 | GO:0045333 | cellular respiration | 80 | COX2, COX3, ND6, NDUFA11, ND3, NDUFV1, NDUFAF1, PINK1, ND2, NDUFS1, PRDM16, NDUFB10, SNCA, NDUFS6, NDUFA6, NDUFS3, ND1, POLG2, NDUFAF2, NDUFB3, NDUFS7, NDUFS4, NDUFV2, NDUFB11, ND4, PARK7, NDUFS8, NDUFB9, TAZ, ND5, COX1, NDUFA1 |
| 25 | GO:0009205 | purine ribonucleoside triphosphate metabolic process | 80 | COX2, COX3, ND6, NDUFA11, ND3, NDUFV1, NDUFAF1, PINK1, ND2, ATP7A, NDUFS1, NDUFB10, SNCA, NDUFS6, NDUFA6, NDUFS3, ND1, NDUFB3, NDUFS7, NDUFS4, MYH7, NDUFV2, ATP8, NDUFB11, LRRK2, ND4, PARK7, NDUFS8, NDUFB9, TAZ, ND5, COX1, NDUFA1 |

---


---


---

# Cluster #29

| HPO | Cluster | Description |
| --- | --- | --- |
| HP:0008981 | 29 | Calf muscle hypertrophy |
| HP:0003236 | 29 | Elevated serum creatine kinase |
| HP:0025169 | 29 | Left ventricular systolic dysfunction |
| HP:0030099 | 29 | Reduced muscle fiber alpha dystroglycan |
| HP:0030197 | 29 | Fatigable weakness of skeletal muscles |

| Cluster | Cell function | Description | Phenotype\_coverage | Genes |
| --- | --- | --- | --- | --- |
| 29 | hsa00514 | Other types of O-glycan biosynthesis | 100 | POMT1, POMT2, POMGNT1 |
| 29 | GO:0060049 | regulation of protein glycosylation | 100 | FKTN, POMT1, POMT2 |
| 29 | GO:0035269 | protein O-linked mannosylation | 100 | POMT1, FKRP, CRPPA, LARGE1, FKTN, POMT2 |
| 29 | GO:0071712 | ER-associated misfolded protein catabolic process | 100 | POMT2 |
| 29 | GO:0010560 | positive regulation of glycoprotein biosynthetic process | 100 | POMT1, POMT2 |
| 29 | R-HSA-3781865 | Diseases of glycosylation | 100 | POMT2, POMT1, DAG1, LARGE1, POMGNT1 |
| 29 | R-HSA-3906995 | Diseases associated with O-glycosylation of proteins | 100 | POMT2, POMT1, DAG1, LARGE1, POMGNT1 |
| 29 | R-HSA-5173105 | O-linked glycosylation | 100 | POMT2, POMT1, DAG1, LARGE1, POMGNT1, POMK |

---


---


---

# Cluster #41

| HPO | Cluster | Description |
| --- | --- | --- |
| HP:0004756 | 41 | Ventricular tachycardia |
| HP:0008331 | 41 | Elevated creatine kinase after exercise |
| HP:0008978 | 41 | Necrotizing myopathy |
| HP:0009045 | 41 | Exercise-induced rhabdomyolysis |

| Cluster | Cell function | Description | Phenotype\_coverage | Genes |
| --- | --- | --- | --- | --- |
| 41 | GO:0071313 | cellular response to caffeine | 100 | CASQ2, CACNA1S, RYR2 |
| 41 | GO:0033539 | fatty acid beta-oxidation using acyl-CoA dehydrogenase | 75 | ACADVL, ACADL |
| 41 | GO:0061337 | cardiac conduction | 75 | CALM1, CASQ2, CACNA1S, RYR2, CALM3, CALM2, TRDN |
| 41 | GO:0045717 | negative regulation of fatty acid biosynthetic process | 75 | ACADVL, ACADL |
| 41 | GO:0046322 | negative regulation of fatty acid oxidation | 75 | ACADVL, ACADL |
| 41 | R-HSA-5576891 | Cardiac conduction | 75 | CACNA1S, CASQ2, CALM1, CALM2, CALM3, RYR2, TRDN |
| 41 | R-HSA-5576893 | Phase 2 - plateau phase | 75 | CACNA1S |
| 41 | R-HSA-5576892 | Phase 0 - rapid depolarisation | 75 | CALM3, CACNA1S, CALM1, CALM2 |
| 41 | R-HSA-419037 | NCAM1 interactions | 75 | CACNA1S |

---


---


---

# Cluster #60

| HPO | Cluster | Description |
| --- | --- | --- |
| HP:0001270 | 60 | Motor delay |
| HP:0002515 | 60 | Waddling gait |
| HP:0003323 | 60 | Progressive muscle weakness |
| HP:0003273 | 60 | Hip contracture |
| HP:0003803 | 60 | Type 1 muscle fiber predominance |
| HP:0002747 | 60 | Respiratory insufficiency due to muscle weakness |
| HP:0003458 | 60 | EMG: myopathic abnormalities |

| Cluster | Cell function | Description | Phenotype\_coverage | Genes |
| --- | --- | --- | --- | --- |
| 60 | hsa05410 | Hypertrophic cardiomyopathy (HCM) | 100.00000 | MYL2, DES, TTN, TPM3, TPM2, EMD, ITGA7, LMNA, MYH7 |
| 60 | hsa05414 | Dilated cardiomyopathy | 100.00000 | MYL2, DES, TTN, TPM3, TPM2, EMD, ITGA7, LMNA, MYH7 |
| 60 | GO:0030049 | muscle filament sliding | 100.00000 | NEB, TNNT1, ACTA1, DES, TTN, MYL2, TPM2, MYH7, TPM3 |
| 60 | GO:0043501 | skeletal muscle adaptation | 100.00000 | MYH7, TNNT1, ACTA1 |
| 60 | GO:0030239 | myofibril assembly | 100.00000 | NEB, TNNT1, ACTA1, TTN, CFL2, MYL2, MYPN, KLHL41, LMOD3 |
| 60 | R-HSA-397014 | Muscle contraction | 100.00000 | DES, TTN, TPM2, RYR1, ACTA1, TPM3, SCN4A, TNNT1, NEB, MYL2 |
| 60 | R-HSA-390522 | Striated Muscle Contraction | 100.00000 | DES, TTN, TPM2, ACTA1, TPM3, TNNT1, NEB, MYL2 |
| 60 | GO:0048741 | skeletal muscle fiber development | 85.71429 | SELENON, ACTA1, MYF6, KLHL41, LMOD3, RYR1, KLHL40 |
| 60 | R-HSA-445355 | Smooth Muscle Contraction | 71.42857 | TPM3, TPM2 |

---


---


---

# Cluster #74

| HPO | Cluster | Description |
| --- | --- | --- |
| HP:0008800 | 74 | Limited hip movement |
| HP:0003327 | 74 | Axial muscle weakness |
| HP:0002747 | 74 | Respiratory insufficiency due to muscle weakness |
| HP:0008963 | 74 | Tibialis muscle weakness |
| HP:0002111 | 74 | Restrictive deficit on pulmonary function testing |
| HP:0003555 | 74 | Muscle fiber splitting |
| HP:0008978 | 74 | Necrotizing myopathy |
| HP:0031237 | 74 | Internally nucleated skeletal muscle fibers |

| Cluster | Cell function | Description | Phenotype\_coverage | Genes |
| --- | --- | --- | --- | --- |
| 74 | GO:0048747 | muscle fiber development | 100.0 | NEB, ACTA1, SELENON, TTN, MYL2, DYSF, MYF6, LMOD3, KLHL41, RYR1, KLHL40 |
| 74 | R-HSA-397014 | Muscle contraction | 87.5 | CACNA1S, TTN, TPM2, RYR1, ACTA1, TPM3, DYSF, TNNT1, NEB, MYL2 |
| 74 | GO:0007517 | muscle organ development | 75.0 | NEB, ACTA1, SELENON, CFL2, FHL1, MYL2, TTN, ITGA7, MYF6, EMD, LMOD3, KLHL41, RYR1, MYH7, KLHL40 |
| 74 | GO:0030049 | muscle filament sliding | 75.0 | NEB, TNNT1, ACTA1, TTN, MYL2, TPM2, MYH7, TPM3 |
| 74 | GO:0014706 | striated muscle tissue development | 75.0 | NEB, ACTA1, SELENON, CFL2, MYL2, TTN, MYF6, EMD, LMOD3, KLHL41, RYR1, MYH7, KLHL40 |
| 74 | GO:0030239 | myofibril assembly | 75.0 | NEB, TNNT1, ACTA1, TTN, CFL2, MYL2, LMOD3, KLHL41 |
| 74 | R-HSA-390522 | Striated Muscle Contraction | 75.0 | TTN, TPM2, ACTA1, TPM3, TNNT1, NEB, MYL2 |

---


---


---

# Cluster #115

| HPO | Cluster | Description |
| --- | --- | --- |
| HP:0001270 | 115 | Motor delay |
| HP:0002375 | 115 | Hypokinesia |
| HP:0001561 | 115 | Polyhydramnios |
| HP:0001558 | 115 | Decreased fetal movement |
| HP:0001349 | 115 | Facial diplegia |
| HP:0003803 | 115 | Type 1 muscle fiber predominance |
| HP:0001623 | 115 | Breech presentation |
| HP:0003798 | 115 | Nemaline bodies |
| HP:0005855 | 115 | Multiple prenatal fractures |
| HP:0009025 | 115 | Increased connective tissue |
| HP:0001989 | 115 | Fetal akinesia sequence |

| Cluster | Cell function | Description | Phenotype\_coverage | Genes |
| --- | --- | --- | --- | --- |
| 115 | GO:0030049 | muscle filament sliding | 100.00000 | NEB, TNNT1, ACTA1, TTN, TPM2, TPM3 |
| 115 | R-HSA-390522 | Striated Muscle Contraction | 100.00000 | TTN, TPM2, ACTA1, TPM3, TNNT1, NEB |
| 115 | GO:0030240 | skeletal muscle thin filament assembly | 90.90909 | LMOD3, ACTA1, TTN |
| 115 | hsa05410 | Hypertrophic cardiomyopathy (HCM) | 81.81818 | TTN, LAMA2, ITGB4, TPM3, TPM2 |
| 115 | hsa05414 | Dilated cardiomyopathy | 81.81818 | TTN, LAMA2, ITGB4, TPM3, TPM2 |
| 115 | GO:0031272 | regulation of pseudopodium assembly | 81.81818 | KLHL41 |
| 115 | GO:0048742 | regulation of skeletal muscle fiber development | 81.81818 | MYOD1, LMOD3, MYF6 |
| 115 | GO:2000291 | regulation of myoblast proliferation | 81.81818 | MYOD1, KLHL41 |
| 115 | GO:0045214 | sarcomere organization | 72.72727 | TNNT1, TTN, CFL2, MYPN, KLHL41 |
| 115 | GO:0035914 | skeletal muscle cell differentiation | 72.72727 | MYOD1, KLHL41, KLHL40, MYF6 |
| 115 | GO:0030832 | regulation of actin filament length | 72.72727 | NEB, BIN1, LMOD3, CFL2 |
| 115 | R-HSA-397014 | Muscle contraction | 72.72727 | TTN, TPM2, RYR1, ACTA1, TPM3, TNNT1, NEB |

---


---


---

# Cluster #122

| HPO | Cluster | Description |
| --- | --- | --- |
| HP:0001663 | 122 | Ventricular fibrillation |
| HP:0011712 | 122 | Right bundle branch block |
| HP:0004756 | 122 | Ventricular tachycardia |
| HP:0001678 | 122 | Atrioventricular block |

| Cluster | Cell function | Description | Phenotype\_coverage | Genes |
| --- | --- | --- | --- | --- |
| 122 | hsa05410 | Hypertrophic cardiomyopathy (HCM) | 100 | CACNA1S, CACNB2, CACNA2D1, LMNA, EMD, RYR2, CACNA1C |
| 122 | hsa05412 | Arrhythmogenic right ventricular cardiomyopathy (ARVC) | 100 | PKP2, CACNA1S, CACNB2, CACNA2D1, LMNA, EMD, RYR2, CACNA1C |
| 122 | hsa05414 | Dilated cardiomyopathy | 100 | CACNA1S, CACNB2, CACNA2D1, LMNA, EMD, RYR2, CACNA1C |
| 122 | hsa04260 | Cardiac muscle contraction | 75 | CACNA1S, CACNB2, CACNA2D1, RYR2, CACNA1C |
| 122 | GO:0098911 | regulation of ventricular cardiac muscle cell action potential | 75 | RYR2, KCNE3, PKP2, CACNA1C |
| 122 | GO:0086070 | SA node cell to atrial cardiac muscle cell communication | 75 | RYR2, HCN4, SCN3B |
| 122 | GO:0043268 | positive regulation of potassium ion transport | 75 | KCNE1, FHL1, KCNE5, KCNJ2, AKAP9, KCNH2, KCNQ1 |
| 122 | GO:1901841 | regulation of high voltage-gated calcium channel activity | 75 | CACNB2, CALM3, CACNA2D1 |
| 122 | GO:0086014 | atrial cardiac muscle cell action potential | 75 | CACNA1C, KCNE5, RYR2, CACNB2, KCNQ1, SCN3B |
| 122 | GO:2001259 | positive regulation of cation channel activity | 75 | CALM1, CACNA2D1, CACNB2, KCNE3, CALM3, CALM2, TRDN |
| 122 | GO:0086016 | AV node cell action potential | 75 | RYR2, CACNB2, CACNA1C |
| 122 | GO:1901021 | positive regulation of calcium ion transmembrane transporter activity | 75 | CALM1, CACNA2D1, RYR2, CACNB2, KCNE3, CALM3, CALM2, TRDN |
| 122 | GO:0086064 | cell communication by electrical coupling involved in cardiac conduction | 75 | CALM1, PKP2, CACNA1C, RYR2, CALM3, CALM2, TRDN |
| 122 | GO:2001258 | negative regulation of cation channel activity | 75 | CALM1, KCNE1, CASQ2, KCNE5, KCNE3, CALM3, CALM2, TRDN |
| 122 | GO:0002027 | regulation of heart rate | 75 | KCNE1, KCNE5, AKAP9, KCNH2, SCN3B, KCND3, CASQ2, SCN2B, RYR2, CACNB2, GPD1L, CACNA1C, KCNE3, CALM3, KCNQ1, CALM1, RANGRF, PKP2, HCN4, CACNA2D1, KCNJ2, CALM2 |
| 122 | GO:1901017 | negative regulation of potassium ion transmembrane transporter activity | 75 | CASQ2, KCNE1, KCNE5, KCNE3 |
| 122 | GO:0060306 | regulation of membrane repolarization | 75 | KCNE1, CASQ2, KCNE5, CACNA2D1, KCNJ2, AKAP9, KCNE3, KCNH2, KCNQ1 |
| 122 | GO:0014074 | response to purine-containing compound | 75 | KCNE1, CASQ2, CACNA1S, HCN4, RYR2, AKAP9, KCNQ1 |
| 122 | GO:0051899 | membrane depolarization | 75 | RANGRF, SLMAP, CACNA1C, FHL1, HCN4, CACNA2D1, SCN2B, KCNJ2, CACNB2, GPD1L, KCNH2, SCN3B |
| 122 | GO:0007204 | positive regulation of cytosolic calcium ion concentration | 75 | CALM1, CACNA1C, CASQ2, CACNA2D1, RYR2, CACNB2, CALM3, CALM2, TRDN |
| 122 | GO:0055117 | regulation of cardiac muscle contraction | 75 | CALM1, RANGRF, PKP2, CACNA1C, CASQ2, HCN4, KCNJ2, RYR2, AKAP9, KCNE3, CALM3, KCNQ1, CALM2 |
| 122 | GO:0097553 | calcium ion transmembrane import into cytosol | 75 | CALM1, CACNA1C, CASQ2, CACNA2D1, RYR2, CALM3, CALM2, TRDN |
| 122 | R-HSA-112316 | Neuronal System | 75 | HCN4, KCNH2, KCND3, GABRA3, CALM1, CALM2, CALM3, KCNJ8, KCNQ1, KCNJ2, CACNA2D1, CACNB2 |
| 122 | R-HSA-397014 | Muscle contraction | 75 | SCN2B, KCNE3, RYR2, AKAP9, CACNB2, CACNA1C, CACNA1S, RANGRF, KCNE5, KCNQ1, CACNA2D1, TRDN, KCND3, CALM3, KCNJ2, KCNH2, KCNE1, CASQ2, SCN3B, CALM1, CALM2 |
| 122 | R-HSA-5576891 | Cardiac conduction | 75 | SCN2B, KCNE3, RYR2, AKAP9, CACNB2, CACNA1C, CACNA1S, RANGRF, KCNE5, KCNQ1, CACNA2D1, TRDN, KCND3, CALM3, KCNJ2, KCNH2, KCNE1, CASQ2, SCN3B, CALM1, CALM2 |
| 122 | R-HSA-5576893 | Phase 2 - plateau phase | 75 | CACNA1C, KCNE1, CACNA1S, KCNE3, AKAP9, KCNQ1, CACNA2D1, CACNB2, KCNE5 |
| 122 | R-HSA-5576892 | Phase 0 - rapid depolarisation | 75 | CACNA1C, CACNA1S, SCN2B, SCN3B, RANGRF, CALM1, CALM2, CALM3, CACNA2D1, CACNB2 |
| 122 | R-HSA-419037 | NCAM1 interactions | 75 | CACNA1C, CACNA1S, CACNB2 |

---


---


---

# Cluster #150

| HPO | Cluster | Description |
| --- | --- | --- |
| HP:0009020 | 150 | Exercise-induced muscle fatigue |
| HP:0003457 | 150 | EMG abnormality |
| HP:0011998 | 150 | Postprandial hyperglycemia |
| HP:0012240 | 150 | Increased intramyocellular lipid droplets |
| HP:0002203 | 150 | Respiratory paralysis |
| HP:0003694 | 150 | Late-onset proximal muscle weakness |
| HP:0003752 | 150 | Episodic flaccid weakness |
| HP:0008153 | 150 | Periodic hypokalemic paresis |
| HP:0012726 | 150 | Episodic hypokalemia |

| Cluster | Cell function | Description | Phenotype\_coverage | Genes |
| --- | --- | --- | --- | --- |
| 150 | R-HSA-5576891 | Cardiac conduction | 100.00000 | CACNA1S, SCN4A, KCNE3 |
| 150 | R-HSA-397014 | Muscle contraction | 100.00000 | CACNA1S, SCN4A, KCNE3 |
| 150 | R-HSA-5576893 | Phase 2 - plateau phase | 100.00000 | CACNA1S, KCNE3 |
| 150 | GO:0071313 | cellular response to caffeine | 88.88889 | CACNA1S |
| 150 | GO:0098911 | regulation of ventricular cardiac muscle cell action potential | 77.77778 | KCNE3 |
| 150 | GO:0051932 | synaptic transmission, GABAergic | 77.77778 | GABRA3 |
| 150 | GO:0010107 | potassium ion import | 77.77778 | KCNJ18 |
| 150 | GO:1905031 | regulation of membrane repolarization during cardiac muscle cell action potential | 77.77778 | KCNE3 |
| 150 | GO:0045822 | negative regulation of heart contraction | 77.77778 | KCNE3 |
| 150 | GO:1990573 | potassium ion import across plasma membrane | 77.77778 | KCNJ18 |
| 150 | GO:0086091 | regulation of heart rate by cardiac conduction | 77.77778 | KCNE3 |
| 150 | GO:0045759 | negative regulation of action potential | 77.77778 | KCNE3 |
| 150 | GO:0071435 | potassium ion export | 77.77778 | KCNE3 |
| 150 | GO:0098915 | membrane repolarization during ventricular cardiac muscle cell action potential | 77.77778 | KCNE3 |
| 150 | GO:1901387 | positive regulation of voltage-gated calcium channel activity | 77.77778 | KCNE3 |
| 150 | GO:0007214 | gamma-aminobutyric acid signaling pathway | 77.77778 | GABRA3 |
| 150 | GO:0097623 | potassium ion export across plasma membrane | 77.77778 | KCNE3 |
| 150 | GO:1902260 | negative regulation of delayed rectifier potassium channel activity | 77.77778 | KCNE3 |
| 150 | GO:0060307 | regulation of ventricular cardiac muscle cell membrane repolarization | 77.77778 | KCNE3 |
| 150 | GO:0071804 | cellular potassium ion transport | 77.77778 | KCNJ18, KCNE3 |
| 150 | GO:0045988 | negative regulation of striated muscle contraction | 77.77778 | KCNE3 |
| 150 | R-HSA-5576892 | Phase 0 - rapid depolarisation | 77.77778 | CACNA1S, SCN4A |
| 150 | R-HSA-419037 | NCAM1 interactions | 77.77778 | CACNA1S |
| 150 | R-HSA-375165 | NCAM signaling for neurite out-growth | 77.77778 | CACNA1S |

---


---


---

# Cluster #22

| HPO | Cluster | Description |
| --- | --- | --- |
| HP:0040083 | 22 | Toe walking |
| HP:0008956 | 22 | Proximal lower limb amyotrophy |
| HP:0008994 | 22 | Proximal muscle weakness in lower limbs |
| HP:0003236 | 22 | Elevated serum creatine kinase |
| HP:0001315 | 22 | Reduced tendon reflexes |
| HP:0003307 | 22 | Hyperlordosis |
| HP:0003306 | 22 | Spinal rigidity |
| HP:0003691 | 22 | Scapular winging |
| HP:0002747 | 22 | Respiratory insufficiency due to muscle weakness |
| HP:0003458 | 22 | EMG: myopathic abnormalities |
| HP:0008997 | 22 | Proximal muscle weakness in upper limbs |
| HP:0002792 | 22 | Reduced vital capacity |
| HP:0003805 | 22 | Rimmed vacuoles |
| HP:0002987 | 22 | Elbow flexion contracture |
| HP:0011807 | 22 | Type 1 muscle fiber atrophy |
| HP:0008948 | 22 | Proximal upper limb amyotrophy |
| HP:0004631 | 22 | Decreased cervical spine flexion due to contractures of posterior cervical muscles |
| HP:0002486 | 22 | Myotonia |

| Cluster | Cell function | Description | Phenotype\_coverage | Genes |
| --- | --- | --- | --- | --- |
| 22 | hsa05410 | Hypertrophic cardiomyopathy (HCM) | 94.44444 | SGCD, MYL2, TTN, TPM3, TPM2, EMD, ITGA7, SGCA, DMD, LMNA, MYH7 |
| 22 | hsa05414 | Dilated cardiomyopathy | 94.44444 | SGCD, MYL2, TTN, TPM3, TPM2, EMD, ITGA7, SGCA, DMD, LMNA, MYH7 |
| 22 | GO:0007517 | muscle organ development | 77.77778 | MYF6, KLHL41, NEB, CAPN3, SGCD, TTN, SELENON, CHRNA1, EMD, FKTN, FHL1, DMD, CFL2, ITGA7, RYR1, MYH7, SGCA, FOS, ACTA1, CHRND, MYL2, LARGE1 |
| 22 | GO:0006998 | nuclear envelope organization | 72.22222 | EMD, TMEM43, LMNA, DMPK |
| 22 | GO:0006936 | muscle contraction | 72.22222 | CHRNE, TNNT1, KLHL41, CLCN1, NEB, SGCD, TTN, CHRNA1, DMPK, TPM2, EMD, DMD, SCN4A, RYR1, MYH7, MYOT, ACTA1, CHRND, MYL2, CHRNB1, PABPN1, TPM3 |

---


---


---

# Cluster #144

| HPO | Cluster | Description |
| --- | --- | --- |
| HP:0040083 | 144 | Toe walking |
| HP:0002515 | 144 | Waddling gait |
| HP:0008956 | 144 | Proximal lower limb amyotrophy |
| HP:0008994 | 144 | Proximal muscle weakness in lower limbs |
| HP:0003236 | 144 | Elevated serum creatine kinase |
| HP:0003307 | 144 | Hyperlordosis |
| HP:0003306 | 144 | Spinal rigidity |
| HP:0003691 | 144 | Scapular winging |
| HP:0008997 | 144 | Proximal muscle weakness in upper limbs |
| HP:0002987 | 144 | Elbow flexion contracture |
| HP:0011807 | 144 | Type 1 muscle fiber atrophy |
| HP:0006785 | 144 | Limb-girdle muscular dystrophy |
| HP:0008948 | 144 | Proximal upper limb amyotrophy |
| HP:0001771 | 144 | Achilles tendon contracture |
| HP:0004631 | 144 | Decreased cervical spine flexion due to contractures of posterior cervical muscles |
| HP:0001678 | 144 | Atrioventricular block |
| HP:0002486 | 144 | Myotonia |

| Cluster | Cell function | Description | Phenotype\_coverage | Genes |
| --- | --- | --- | --- | --- |
| 144 | hsa05410 | Hypertrophic cardiomyopathy (HCM) | 94.11765 | SGCD, SGCG, DAG1, MYL2, TTN, TPM3, TPM2, EMD, ITGA7, SGCA, DMD, LMNA, MYH7 |
| 144 | hsa05414 | Dilated cardiomyopathy | 94.11765 | SGCD, SGCG, DAG1, MYL2, TTN, TPM3, TPM2, EMD, ITGA7, SGCA, DMD, LMNA, MYH7 |
| 144 | GO:0007084 | mitotic nuclear envelope reassembly | 88.23529 | EMD, LMNA |
| 144 | R-HSA-2995410 | Nuclear Envelope Reassembly | 88.23529 | LMNA, EMD |
| 144 | R-HSA-2995383 | Initiation of Nuclear Envelope Reformation | 88.23529 | LMNA, EMD |
| 144 | R-HSA-4419969 | Depolymerisation of the Nuclear Lamina | 88.23529 | LMNA, EMD |
| 144 | hsa05412 | Arrhythmogenic right ventricular cardiomyopathy (ARVC) | 82.35294 | SGCD, SGCG, DAG1, EMD, ITGA7, SGCA, LMNA |
| 144 | R-HSA-2980766 | Nuclear Envelope Breakdown | 82.35294 | LMNA, EMD |
| 144 | R-HSA-1221632 | Meiotic synapsis | 76.47059 | LMNA, SYNE1, SYNE2 |
| 144 | GO:0007517 | muscle organ development | 70.58824 | MYF6, KLHL41, NEB, CAPN3, SGCD, SELENON, TTN, EMD, FKTN, FHL1, DMD, CFL2, ITGA7, RYR1, MYH7, SGCA, FOS, ACTA1, MYL2, SGCG, LARGE1 |
| 144 | R-HSA-1500620 | Meiosis | 70.58824 | LMNA, SYNE1, SYNE2 |

---


---


---

# Cluster #112

| HPO | Cluster | Description |
| --- | --- | --- |
| HP:0001270 | 112 | Motor delay |
| HP:0040083 | 112 | Toe walking |
| HP:0002515 | 112 | Waddling gait |
| HP:0008994 | 112 | Proximal muscle weakness in lower limbs |
| HP:0003236 | 112 | Elevated serum creatine kinase |
| HP:0003391 | 112 | Gowers sign |
| HP:0003306 | 112 | Spinal rigidity |
| HP:0003691 | 112 | Scapular winging |
| HP:0003803 | 112 | Type 1 muscle fiber predominance |
| HP:0003307 | 112 | Hyperlordosis |
| HP:0001558 | 112 | Decreased fetal movement |
| HP:0003805 | 112 | Rimmed vacuoles |
| HP:0006785 | 112 | Limb-girdle muscular dystrophy |
| HP:0001771 | 112 | Achilles tendon contracture |

| Cluster | Cell function | Description | Phenotype\_coverage | Genes |
| --- | --- | --- | --- | --- |
| 112 | GO:0007517 | muscle organ development | 92.85714 | MYF6, KLHL41, LMOD3, NEB, CAPN3, SELENON, TTN, CHRNA1, EMD, SGCB, FKTN, KLHL40, SPEG, FHL1, DMD, CFL2, RYR1, MYH7, SGCA, FOS, ACTA1, CHRND, SGCG, LARGE1 |
| 112 | hsa05410 | Hypertrophic cardiomyopathy (HCM) | 85.71429 | SGCB, SGCG, DAG1, TTN, TPM3, TPM2, EMD, SGCA, DMD, LMNA, MYH7 |
| 112 | hsa05414 | Dilated cardiomyopathy | 85.71429 | SGCB, SGCG, DAG1, TTN, TPM3, TPM2, EMD, SGCA, DMD, LMNA, MYH7 |
| 112 | GO:0014706 | striated muscle tissue development | 78.57143 | NEB, ACVR1, SELENON, ACTA1, CFL2, TTN, CHRNA1, CHRND, LMNA, MYF6, SGCG, EMD, KLHL41, LMOD3, RYR1, SGCB, KLHL40, FOS |
| 112 | GO:0003012 | muscle system process | 71.42857 | CHRNE, TNNT1, LMNA, KLHL41, LMOD3, NEB, TTN, CHRNA1, TPM2, EMD, DMD, RYR1, MYH7, SGCA, MYOT, ACTA1, CHRND, BIN1, CHRNB1, PABPN1, TPM3 |
| 112 | GO:0048747 | muscle fiber development | 71.42857 | NEB, SELENON, ACTA1, TTN, DMD, MYF6, CHRNB1, KLHL41, LMOD3, RYR1, SGCB, FLNC, KLHL40 |

---


---


---

# Cluster #13

| HPO | Cluster | Description |
| --- | --- | --- |
| HP:0001290 | 13 | Generalized hypotonia |
| HP:0002515 | 13 | Waddling gait |
| HP:0009046 | 13 | Difficulty running |
| HP:0003236 | 13 | Elevated serum creatine kinase |
| HP:0003391 | 13 | Gowers sign |
| HP:0003557 | 13 | Increased variability in muscle fiber diameter |
| HP:0003700 | 13 | Generalized amyotrophy |
| HP:0003306 | 13 | Spinal rigidity |
| HP:0003551 | 13 | Difficulty climbing stairs |
| HP:0003687 | 13 | Centrally nucleated skeletal muscle fibers |
| HP:0003803 | 13 | Type 1 muscle fiber predominance |

| Cluster | Cell function | Description | Phenotype\_coverage | Genes |
| --- | --- | --- | --- | --- |
| 13 | GO:0048747 | muscle fiber development | 90.90909 | NEB, SELENON, ACTA1, TTN, DMD, DYSF, MYF6, CHRNB1, KLHL41, LMOD3, RYR1, SGCB, FLNC, KLHL40 |
| 13 | GO:0007517 | muscle organ development | 81.81818 | MYF6, KLHL41, LMOD3, NEB, CAPN3, SELENON, TTN, CHRNA1, CASQ1, EMD, SGCB, FKTN, KLHL40, SPEG, DMD, CFL2, FHL1, RYR1, MYH7, SGCA, FOS, ACTA1, CHRND, SGCG, LARGE1 |
| 13 | GO:0042692 | muscle cell differentiation | 72.72727 | TNNT1, MYPN, LMNA, MYF6, KLHL41, LMOD3, FLNC, NEB, CAPN3, SELENON, TTN, CASQ1, SGCB, KLHL40, ACVR1, SPEG, CFL2, RYR1, ACTA1, BIN1, CHRNB1 |

---


---


---

# Cluster #56

| HPO | Cluster | Description |
| --- | --- | --- |
| HP:0009020 | 56 | Exercise-induced muscle fatigue |
| HP:0003200 | 56 | Ragged-red muscle fibers |
| HP:0003457 | 56 | EMG abnormality |
| HP:0008180 | 56 | Mildly elevated creatine kinase |
| HP:0011998 | 56 | Postprandial hyperglycemia |
| HP:0012240 | 56 | Increased intramyocellular lipid droplets |
| HP:0002203 | 56 | Respiratory paralysis |
| HP:0003694 | 56 | Late-onset proximal muscle weakness |
| HP:0003752 | 56 | Episodic flaccid weakness |
| HP:0008153 | 56 | Periodic hypokalemic paresis |
| HP:0012726 | 56 | Episodic hypokalemia |

| Cluster | Cell function | Description | Phenotype\_coverage | Genes |
| --- | --- | --- | --- | --- |
| 56 | R-HSA-397014 | Muscle contraction | 90.90909 | CACNA1S, TPM2, KCNE3, ACTA1, TPM3, SCN4A |
| 56 | R-HSA-5576893 | Phase 2 - plateau phase | 90.90909 | CACNA1S, KCNE3 |
| 56 | GO:0034765 | regulation of ion transmembrane transport | 81.81818 | SCN4A, CACNA1S, KCNE3, CHCHD10, KCNJ18 |
| 56 | GO:0071313 | cellular response to caffeine | 81.81818 | CACNA1S |
| 56 | GO:0006936 | muscle contraction | 81.81818 | ACTA1, TPM2, SCN4A, CACNA1S, KCNE3, TPM3 |
| 56 | R-HSA-5576891 | Cardiac conduction | 81.81818 | CACNA1S, SCN4A, KCNE3 |
| 56 | GO:1903522 | regulation of blood circulation | 72.72727 | KCNE3, DBH, CACNA1S |
| 56 | GO:0061337 | cardiac conduction | 72.72727 | KCNE3, CACNA1S |
| 56 | R-HSA-5576892 | Phase 0 - rapid depolarisation | 72.72727 | CACNA1S, SCN4A |
| 56 | R-HSA-419037 | NCAM1 interactions | 72.72727 | CACNA1S |
| 56 | R-HSA-375165 | NCAM signaling for neurite out-growth | 72.72727 | CACNA1S |

---


---


---

# Cluster #124

| HPO | Cluster | Description |
| --- | --- | --- |
| HP:0001270 | 124 | Motor delay |
| HP:0002359 | 124 | Frequent falls |
| HP:0002515 | 124 | Waddling gait |
| HP:0008994 | 124 | Proximal muscle weakness in lower limbs |
| HP:0009046 | 124 | Difficulty running |
| HP:0040083 | 124 | Toe walking |
| HP:0009027 | 124 | Foot dorsiflexor weakness |
| HP:0001265 | 124 | Hyporeflexia |
| HP:0001284 | 124 | Areflexia |
| HP:0003236 | 124 | Elevated serum creatine kinase |
| HP:0003391 | 124 | Gowers sign |
| HP:0002421 | 124 | Poor head control |
| HP:0001349 | 124 | Facial diplegia |
| HP:0003691 | 124 | Scapular winging |
| HP:0003458 | 124 | EMG: myopathic abnormalities |
| HP:0001771 | 124 | Achilles tendon contracture |
| HP:0003547 | 124 | Shoulder girdle muscle weakness |
| HP:0003327 | 124 | Axial muscle weakness |
| HP:0003803 | 124 | Type 1 muscle fiber predominance |
| HP:0008997 | 124 | Proximal muscle weakness in upper limbs |

| Cluster | Cell function | Description | Phenotype\_coverage | Genes |
| --- | --- | --- | --- | --- |
| 124 | GO:0007517 | muscle organ development | 90 | MYF6, KLHL41, LMOD3, NEB, CAPN3, SELENON, TTN, CHRNA1, EMD, SGCB, FKTN, KLHL40, SPEG, FHL1, DMD, CFL2, RYR1, MYH7, SGCA, ACTA1, CHRND, SGCG, LARGE1 |
| 124 | GO:0014706 | striated muscle tissue development | 75 | NEB, SELENON, ACTA1, TTN, CFL2, CHRND, CHRNA1, LMNA, MYF6, SGCG, EMD, KLHL41, LMOD3, RYR1, SGCB, MYH7, KLHL40 |
| 124 | GO:0048747 | muscle fiber development | 75 | NEB, SELENON, ACTA1, TTN, DMD, MYF6, CHRNB1, KLHL41, LMOD3, RYR1, SGCB, FLNC, KLHL40 |
| 124 | GO:0042692 | muscle cell differentiation | 70 | NEB, TNNT1, ACTA1, SELENON, SPEG, TTN, CFL2, MYPN, LMNA, BIN1, MYF6, CHRNB1, KLHL41, LMOD3, RYR1, SGCB, FLNC, KLHL40 |

---


---


---

# Cluster #131

| HPO | Cluster | Description |
| --- | --- | --- |
| HP:0030200 | 131 | Fatiguable weakness of proximal limb muscles |
| HP:0003738 | 131 | Exercise-induced myalgia |
| HP:0003325 | 131 | Limb-girdle muscle weakness |
| HP:0008954 | 131 | Intrinsic hand muscle atrophy |
| HP:0008959 | 131 | Distal upper limb muscle weakness |
| HP:0008963 | 131 | Tibialis muscle weakness |
| HP:0009005 | 131 | Weakness of the intrinsic hand muscles |
| HP:0031177 | 131 | Finger flexor weakness |
| HP:0030198 | 131 | Fatigable weakness of distal limb muscles |
| HP:0012515 | 131 | Hip flexor weakness |

| Cluster | Cell function | Description | Phenotype\_coverage | Genes |
| --- | --- | --- | --- | --- |
| 131 | GO:0055002 | striated muscle cell development | 90 | NEB, CFL2, DYSF, MYPN, CASQ1, LDB3, CAV3, MYF6, CHRNB1, LMOD3, KLHL41, FLNC |

---


---


---

# Cluster #48

| HPO | Cluster | Description |
| --- | --- | --- |
| HP:0001270 | 48 | Motor delay |
| HP:0002355 | 48 | Difficulty walking |
| HP:0001436 | 48 | Abnormality of the foot musculature |
| HP:0001290 | 48 | Generalized hypotonia |
| HP:0002515 | 48 | Waddling gait |
| HP:0009046 | 48 | Difficulty running |
| HP:0008981 | 48 | Calf muscle hypertrophy |
| HP:0000602 | 48 | Ophthalmoplegia |
| HP:0002058 | 48 | Myopathic facies |
| HP:0003391 | 48 | Gowers sign |
| HP:0003557 | 48 | Increased variability in muscle fiber diameter |
| HP:0003306 | 48 | Spinal rigidity |
| HP:0003551 | 48 | Difficulty climbing stairs |
| HP:0003687 | 48 | Centrally nucleated skeletal muscle fibers |
| HP:0003691 | 48 | Scapular winging |
| HP:0003803 | 48 | Type 1 muscle fiber predominance |
| HP:0002747 | 48 | Respiratory insufficiency due to muscle weakness |
| HP:0003458 | 48 | EMG: myopathic abnormalities |
| HP:0031108 | 48 | Triceps weakness |

| Cluster | Cell function | Description | Phenotype\_coverage | Genes |
| --- | --- | --- | --- | --- |
| 48 | GO:0048747 | muscle fiber development | 89.47368 | NEB, SELENON, ACTA1, TTN, MYL2, DYSF, MYF6, CHRNB1, STAC3, KLHL41, LMOD3, RYR1, SGCB, FLNC, KLHL40 |
| 48 | GO:0042692 | muscle cell differentiation | 78.94737 | TNNT1, MYPN, LMNA, MYF6, STAC3, KLHL41, LMOD3, FLNC, NEB, CAPN3, SELENON, TTN, SYNE1, CASQ1, SGCB, KLHL40, ACVR1, SPEG, CFL2, RYR1, ACTA1, MYL2, BIN1, CHRNB1 |
| 48 | GO:0007517 | muscle organ development | 78.94737 | MYF6, STAC3, KLHL41, LMOD3, NEB, CAPN3, SELENON, TTN, CHRNA1, CASQ1, EMD, SGCB, KLHL40, SPEG, CFL2, FHL1, ITGA7, LAMA2, RYR1, MYH7, SGCA, FOS, ACTA1, CHRND, MYL2, SGCG |
| 48 | GO:0014706 | striated muscle tissue development | 73.68421 | LMNA, MYF6, STAC3, KLHL41, LMOD3, NEB, SELENON, TTN, CHRNA1, CASQ1, EMD, SGCB, KLHL40, ACVR1, CFL2, RYR1, MYH7, FOS, ACTA1, CHRND, MYL2, SGCG |
| 48 | GO:0006936 | muscle contraction | 73.68421 | CHRNE, TNNT1, STAC3, KLHL41, LMOD3, NEB, TTN, CHRNA1, TPM2, CASQ1, EMD, SCN4A, RYR1, MYH7, MYOT, ACTA1, CHRND, MYL2, DYSF, BIN1, CHRNB1, TPM3 |

---


---


---

# Cluster #21

| HPO | Cluster | Description |
| --- | --- | --- |
| HP:0001436 | 21 | Abnormality of the foot musculature |
| HP:0009027 | 21 | Foot dorsiflexor weakness |
| HP:0031374 | 21 | Ankle weakness |
| HP:0003458 | 21 | EMG: myopathic abnormalities |
| HP:0030198 | 21 | Fatigable weakness of distal limb muscles |
| HP:0009005 | 21 | Weakness of the intrinsic hand muscles |
| HP:0003805 | 21 | Rimmed vacuoles |
| HP:0003547 | 21 | Shoulder girdle muscle weakness |
| HP:0009077 | 21 | Weakness of long finger extensor muscles |

| Cluster | Cell function | Description | Phenotype\_coverage | Genes |
| --- | --- | --- | --- | --- |
| 21 | GO:0006941 | striated muscle contraction | 88.88889 | TTN, CHRND, CHRNA1, SCN4A, CAV3, BIN1, CHRNB1, KLHL41, LMOD3, MYH7 |
| 21 | GO:0014706 | striated muscle tissue development | 77.77778 | NEB, ACTA1, TTN, CFL2, CHRND, CHRNA1, CAV3, LMNA, MYF6, EMD, KLHL41, LMOD3, MYH7 |
| 21 | GO:0007517 | muscle organ development | 77.77778 | NEB, ACTA1, TTN, CFL2, CHRND, CHRNA1, FHL1, CAV3, MYF6, EMD, KLHL41, LMOD3, MYH7 |

---


---


---

# Cluster #47

| HPO | Cluster | Description |
| --- | --- | --- |
| HP:0001270 | 47 | Motor delay |
| HP:0001436 | 47 | Abnormality of the foot musculature |
| HP:0009027 | 47 | Foot dorsiflexor weakness |
| HP:0002515 | 47 | Waddling gait |
| HP:0001265 | 47 | Hyporeflexia |
| HP:0009046 | 47 | Difficulty running |
| HP:0001284 | 47 | Areflexia |
| HP:0003236 | 47 | Elevated serum creatine kinase |
| HP:0002375 | 47 | Hypokinesia |
| HP:0002058 | 47 | Myopathic facies |
| HP:0003391 | 47 | Gowers sign |
| HP:0003557 | 47 | Increased variability in muscle fiber diameter |
| HP:0002938 | 47 | Lumbar hyperlordosis |
| HP:0003306 | 47 | Spinal rigidity |
| HP:0001349 | 47 | Facial diplegia |
| HP:0003691 | 47 | Scapular winging |
| HP:0003803 | 47 | Type 1 muscle fiber predominance |
| HP:0003458 | 47 | EMG: myopathic abnormalities |
| HP:0003798 | 47 | Nemaline bodies |
| HP:0009025 | 47 | Increased connective tissue |
| HP:0002792 | 47 | Reduced vital capacity |
| HP:0008963 | 47 | Tibialis muscle weakness |
| HP:0040081 | 47 | Abnormal circulating creatine kinase concentration |
| HP:0003555 | 47 | Muscle fiber splitting |
| HP:0003805 | 47 | Rimmed vacuoles |
| HP:0003730 | 47 | EMG: myotonic runs |
| HP:0012548 | 47 | Fatty replacement of skeletal muscle |
| HP:0100297 | 47 | Increased endomysial connective tissue |
| HP:0003547 | 47 | Shoulder girdle muscle weakness |
| HP:0003707 | 47 | Calf muscle pseudohypertrophy |
| HP:0030007 | 47 | EMG: positive sharp waves |
| HP:0100284 | 47 | EMG: myotonic discharges |
| HP:0009063 | 47 | Progressive distal muscle weakness |
| HP:0006467 | 47 | Limited shoulder movement |

| Cluster | Cell function | Description | Phenotype\_coverage | Genes |
| --- | --- | --- | --- | --- |
| 47 | GO:0007517 | muscle organ development | 88.23529 | MYOD1, MYF6, STAC3, KLHL41, LMOD3, NEB, CAPN3, TTN, SELENON, CHRNA1, EMD, SGCB, FKTN, KLHL40, CRYAB, SPEG, DMD, CFL2, FHL1, MYH14, CAV3, ITGA7, LAMA2, RYR1, COL6A3, MYH7, SGCA, FOS, ACTA1, CHRND, MYL2, SGCG, LARGE1 |
| 47 | GO:0014706 | striated muscle tissue development | 73.52941 | MYOD1, LMNA, MYF6, STAC3, KLHL41, LMOD3, NEB, TTN, SELENON, CHRNA1, EMD, SGCB, KLHL40, ACVR1, CFL2, CAV3, RYR1, MYH7, FOS, ACTA1, CHRND, MYL2, SGCG |
| 47 | GO:0006936 | muscle contraction | 73.52941 | CHRNE, TNNT1, STAC3, KLHL41, LMOD3, NEB, TTN, CHRNA1, TPM2, EMD, DMD, MYH14, SCN4A, CAV3, RYR1, MYH7, SGCA, MYOT, ACTA1, CHRND, MYL2, BIN1, CHRNB1, CRYAB, PABPN1, TPM3 |

---


---


---

# Cluster #113

| HPO | Cluster | Description |
| --- | --- | --- |
| HP:0007126 | 113 | Proximal amyotrophy |
| HP:0030099 | 113 | Reduced muscle fiber alpha dystroglycan |
| HP:0030197 | 113 | Fatigable weakness of skeletal muscles |
| HP:0003741 | 113 | Congenital muscular dystrophy |
| HP:0030234 | 113 | Highly elevated creatine kinase |
| HP:0003797 | 113 | Limb-girdle muscle atrophy |
| HP:0030046 | 113 | Hypoglycosylation of alpha-dystroglycan |
| HP:0002350 | 113 | Cerebellar cyst |

| Cluster | Cell function | Description | Phenotype\_coverage | Genes |
| --- | --- | --- | --- | --- |
| 113 | GO:0035269 | protein O-linked mannosylation | 87.5 | POMT1, FKTN, CRPPA, LARGE1, FKRP, POMT2 |
| 113 | hsa00514 | Other types of O-glycan biosynthesis | 75.0 | POMT1, POMT2, POMGNT1 |
| 113 | GO:0060049 | regulation of protein glycosylation | 75.0 | FKTN, POMT1, POMT2 |
| 113 | GO:0010560 | positive regulation of glycoprotein biosynthetic process | 75.0 | POMT1, POMT2 |
| 113 | R-HSA-3781865 | Diseases of glycosylation | 75.0 | POMT2, POMT1, DAG1, LARGE1, POMGNT1 |
| 113 | R-HSA-3906995 | Diseases associated with O-glycosylation of proteins | 75.0 | POMT2, POMT1, DAG1, LARGE1, POMGNT1 |
| 113 | R-HSA-5173105 | O-linked glycosylation | 75.0 | POMT2, POMT1, DAG1, LARGE1, POMGNT1, POMK |

---


---


---

# Cluster #128

| HPO | Cluster | Description |
| --- | --- | --- |
| HP:0008800 | 128 | Limited hip movement |
| HP:0008981 | 128 | Calf muscle hypertrophy |
| HP:0003236 | 128 | Elevated serum creatine kinase |
| HP:0011712 | 128 | Right bundle branch block |
| HP:0003557 | 128 | Increased variability in muscle fiber diameter |
| HP:0003691 | 128 | Scapular winging |
| HP:0003458 | 128 | EMG: myopathic abnormalities |
| HP:0003551 | 128 | Difficulty climbing stairs |

| Cluster | Cell function | Description | Phenotype\_coverage | Genes |
| --- | --- | --- | --- | --- |
| 128 | GO:0048747 | muscle fiber development | 87.5 | NEB, TTN, ACTA1, DMD, DYSF, KLHL41, RYR1 |
| 128 | R-HSA-445355 | Smooth Muscle Contraction | 75.0 | TPM3, TPM2, DYSF |

---


---


---

# Cluster #137

| HPO | Cluster | Description |
| --- | --- | --- |
| HP:0001270 | 137 | Motor delay |
| HP:0002359 | 137 | Frequent falls |
| HP:0001290 | 137 | Generalized hypotonia |
| HP:0002515 | 137 | Waddling gait |
| HP:0001265 | 137 | Hyporeflexia |
| HP:0009046 | 137 | Difficulty running |
| HP:0001284 | 137 | Areflexia |
| HP:0003236 | 137 | Elevated serum creatine kinase |
| HP:0003391 | 137 | Gowers sign |
| HP:0003306 | 137 | Spinal rigidity |
| HP:0003551 | 137 | Difficulty climbing stairs |
| HP:0003691 | 137 | Scapular winging |
| HP:0003803 | 137 | Type 1 muscle fiber predominance |
| HP:0003458 | 137 | EMG: myopathic abnormalities |
| HP:0003388 | 137 | Easy fatigability |
| HP:0001349 | 137 | Facial diplegia |

| Cluster | Cell function | Description | Phenotype\_coverage | Genes |
| --- | --- | --- | --- | --- |
| 137 | GO:0048747 | muscle fiber development | 87.50 | NEB, SELENON, ACTA1, TTN, DMD, DYSF, MYF6, CHRNB1, KLHL41, LMOD3, RYR1, SGCB, FLNC, KLHL40 |
| 137 | GO:0007517 | muscle organ development | 81.25 | MYF6, KLHL41, LMOD3, NEB, CAPN3, SELENON, TTN, CHRNA1, EMD, SGCB, FKTN, KLHL40, SPEG, DMD, CFL2, FHL1, RYR1, MYH7, SGCA, FOS, ACTA1, CHRND, SGCG, LARGE1 |
| 137 | GO:0042692 | muscle cell differentiation | 75.00 | NEB, CAPN3, ACVR1, TNNT1, ACTA1, SELENON, SPEG, CFL2, TTN, MYPN, LMNA, BIN1, MYF6, CHRNB1, KLHL41, LMOD3, RYR1, SGCB, FLNC, KLHL40 |
| 137 | GO:0006936 | muscle contraction | 75.00 | NEB, MYOT, CHRNE, TNNT1, ACTA1, TTN, DMD, CHRNA1, TPM2, CHRND, SCN4A, BIN1, CHRNB1, EMD, KLHL41, LMOD3, RYR1, MYH7, TPM3 |

---


---


---

# Cluster #93

| HPO | Cluster | Description |
| --- | --- | --- |
| HP:0001663 | 93 | Ventricular fibrillation |
| HP:0009020 | 93 | Exercise-induced muscle fatigue |
| HP:0003201 | 93 | Rhabdomyolysis |
| HP:0003694 | 93 | Late-onset proximal muscle weakness |
| HP:0012726 | 93 | Episodic hypokalemia |
| HP:0002203 | 93 | Respiratory paralysis |
| HP:0003752 | 93 | Episodic flaccid weakness |

| Cluster | Cell function | Description | Phenotype\_coverage | Genes |
| --- | --- | --- | --- | --- |
| 93 | GO:0010107 | potassium ion import | 85.71429 | SLC12A3, HCN4, KCNJ2, KCNJ18, KCNJ8, KCNH2 |
| 93 | GO:1990573 | potassium ion import across plasma membrane | 85.71429 | SLC12A3, HCN4, KCNJ2, KCNJ18, KCNJ8, KCNH2 |
| 93 | GO:1902476 | chloride transmembrane transport | 85.71429 | CLCNKB, SLC12A3, GABRA3 |
| 93 | GO:0071804 | cellular potassium ion transport | 85.71429 | KCNE1, KCND3, KCNE5, SLC12A3, HCN4, KCNJ2, AKAP9, KCNE3, KCNJ18, KCNJ8, KCNH2, KCNQ1 |
| 93 | R-HSA-5576891 | Cardiac conduction | 85.71429 | KCNH2, CACNA1C, KCNE1, CACNA1S, KCND3, SCN2B, SCN3B, RANGRF, KCNE3, SCN4A, AKAP9, KCNQ1, KCNJ2, CACNA2D1, CACNB2, KCNE5 |
| 93 | R-HSA-397014 | Muscle contraction | 85.71429 | KCNH2, CACNA1C, KCNE1, CACNA1S, KCND3, SCN2B, SCN3B, RANGRF, KCNE3, SCN4A, AKAP9, KCNQ1, KCNJ2, CACNA2D1, CACNB2, KCNE5 |
| 93 | R-HSA-5576893 | Phase 2 - plateau phase | 85.71429 | CACNA1C, KCNE1, CACNA1S, KCNE3, AKAP9, KCNQ1, CACNA2D1, CACNB2, KCNE5 |
| 93 | GO:0098911 | regulation of ventricular cardiac muscle cell action potential | 71.42857 | KCNE3, PKP2, CACNA1C |
| 93 | GO:1905031 | regulation of membrane repolarization during cardiac muscle cell action potential | 71.42857 | KCNE3 |
| 93 | GO:0086091 | regulation of heart rate by cardiac conduction | 71.42857 | KCNE1, KCND3, PKP2, CACNA1C, KCNE5, HCN4, CACNA2D1, SCN2B, KCNJ2, CACNB2, AKAP9, KCNE3, KCNH2, KCNQ1, SCN3B |
| 93 | GO:0071435 | potassium ion export | 71.42857 | KCNE1, KCND3, KCNE5, KCNE3, KCNH2, KCNQ1 |
| 93 | GO:0071313 | cellular response to caffeine | 71.42857 | CACNA1S |
| 93 | GO:0098915 | membrane repolarization during ventricular cardiac muscle cell action potential | 71.42857 | KCNE1, KCND3, KCNE5, KCNE3, KCNJ8, KCNH2, KCNQ1 |
| 93 | GO:1901387 | positive regulation of voltage-gated calcium channel activity | 71.42857 | CACNB2, KCNE3, CACNA2D1 |
| 93 | GO:0097623 | potassium ion export across plasma membrane | 71.42857 | KCNE1, KCND3, KCNE5, KCNE3, KCNH2, KCNQ1 |
| 93 | GO:0006936 | muscle contraction | 71.42857 | KCNE1, KCND3, SLMAP, CACNA1C, PKP2, KCNE5, SCN4A, HCN4, CACNA1S, CACNA2D1, SCN2B, KCNJ2, CACNB2, KCNE3, SCN3B, KCNJ8, KCNH2, KCNQ1, GPD1L |
| 93 | GO:0070509 | calcium ion import | 71.42857 | CACNB2, CACNA1S, CACNA1C, CACNA2D1 |
| 93 | GO:1902260 | negative regulation of delayed rectifier potassium channel activity | 71.42857 | KCNE1, KCNE3, KCNE5 |
| 93 | GO:0060307 | regulation of ventricular cardiac muscle cell membrane repolarization | 71.42857 | KCNE1, KCNE5, CACNA2D1, AKAP9, KCNE3, KCNH2, KCNQ1 |
| 93 | GO:0045988 | negative regulation of striated muscle contraction | 71.42857 | KCNE3 |
| 93 | R-HSA-977443 | GABA receptor activation | 71.42857 | KCNJ2, GABRA3 |
| 93 | R-HSA-5576892 | Phase 0 - rapid depolarisation | 71.42857 | CACNA1C, CACNA1S, SCN2B, SCN3B, RANGRF, SCN4A, CACNA2D1, CACNB2 |
| 93 | R-HSA-419037 | NCAM1 interactions | 71.42857 | CACNA1C, CACNA1S, CACNB2 |
| 93 | R-HSA-375165 | NCAM signaling for neurite out-growth | 71.42857 | CACNA1C, CACNA1S, CACNB2 |

---


---


---

# Cluster #141

| HPO | Cluster | Description |
| --- | --- | --- |
| HP:0003201 | 141 | Rhabdomyolysis |
| HP:0008331 | 141 | Elevated creatine kinase after exercise |
| HP:0002153 | 141 | Hyperkalemia |
| HP:0003552 | 141 | Muscle stiffness |
| HP:0009045 | 141 | Exercise-induced rhabdomyolysis |
| HP:0012899 | 141 | Handgrip myotonia |
| HP:0008153 | 141 | Periodic hypokalemic paresis |

| Cluster | Cell function | Description | Phenotype\_coverage | Genes |
| --- | --- | --- | --- | --- |
| 141 | GO:0071313 | cellular response to caffeine | 85.71429 | CACNA1S, RYR1 |

---


---


---

# Cluster #8

| HPO | Cluster | Description |
| --- | --- | --- |
| HP:0003198 | 8 | Myopathy |
| HP:0001270 | 8 | Motor delay |
| HP:0001290 | 8 | Generalized hypotonia |
| HP:0002515 | 8 | Waddling gait |
| HP:0003391 | 8 | Gowers sign |
| HP:0003307 | 8 | Hyperlordosis |
| HP:0002421 | 8 | Poor head control |
| HP:0003306 | 8 | Spinal rigidity |
| HP:0003273 | 8 | Hip contracture |
| HP:0003691 | 8 | Scapular winging |
| HP:0003803 | 8 | Type 1 muscle fiber predominance |
| HP:0002747 | 8 | Respiratory insufficiency due to muscle weakness |
| HP:0002987 | 8 | Elbow flexion contracture |

| Cluster | Cell function | Description | Phenotype\_coverage | Genes |
| --- | --- | --- | --- | --- |
| 8 | GO:0048741 | skeletal muscle fiber development | 84.61538 | SELENON, ACTA1, MYF6, KLHL41, LMOD3, RYR1, KLHL40 |
| 8 | GO:0003012 | muscle system process | 76.92308 | NEB, TNNT1, CHRNE, ACTA1, TTN, CHRNA1, MYL2, TPM2, CHRND, LMNA, BIN1, CHRNB1, EMD, KLHL41, LMOD3, RYR1, TPM3 |

---


---


---

# Cluster #103

| HPO | Cluster | Description |
| --- | --- | --- |
| HP:0001436 | 103 | Abnormality of the foot musculature |
| HP:0008956 | 103 | Proximal lower limb amyotrophy |
| HP:0003236 | 103 | Elevated serum creatine kinase |
| HP:0003307 | 103 | Hyperlordosis |
| HP:0003691 | 103 | Scapular winging |
| HP:0008963 | 103 | Tibialis muscle weakness |
| HP:0003555 | 103 | Muscle fiber splitting |
| HP:0003730 | 103 | EMG: myotonic runs |
| HP:0006467 | 103 | Limited shoulder movement |
| HP:0030007 | 103 | EMG: positive sharp waves |
| HP:0100284 | 103 | EMG: myotonic discharges |
| HP:0003805 | 103 | Rimmed vacuoles |
| HP:0006785 | 103 | Limb-girdle muscular dystrophy |

| Cluster | Cell function | Description | Phenotype\_coverage | Genes |
| --- | --- | --- | --- | --- |
| 103 | GO:0007517 | muscle organ development | 84.61538 | SGCD, TTN, SELENON, FHL1, DMD, ACTA1, MYF6, SGCG, EMD, RYR1, LARGE1, MYH7, SGCA, FKTN |
| 103 | hsa05410 | Hypertrophic cardiomyopathy (HCM) | 76.92308 | SGCD, SGCG, DAG1, TTN, TPM3, TPM2, EMD, SGCA, DMD, LMNA, MYH7 |
| 103 | hsa05414 | Dilated cardiomyopathy | 76.92308 | SGCD, SGCG, DAG1, TTN, TPM3, TPM2, EMD, SGCA, DMD, LMNA, MYH7 |

---


---


---

# Cluster #130

| HPO | Cluster | Description |
| --- | --- | --- |
| HP:0031374 | 130 | Ankle weakness |
| HP:0001290 | 130 | Generalized hypotonia |
| HP:0001265 | 130 | Hyporeflexia |
| HP:0003557 | 130 | Increased variability in muscle fiber diameter |
| HP:0003306 | 130 | Spinal rigidity |
| HP:0003458 | 130 | EMG: myopathic abnormalities |
| HP:0008180 | 130 | Mildly elevated creatine kinase |
| HP:0001623 | 130 | Breech presentation |
| HP:0001989 | 130 | Fetal akinesia sequence |
| HP:0009058 | 130 | Increased muscle lipid content |
| HP:0030192 | 130 | Fatigable weakness of bulbar muscles |
| HP:0002111 | 130 | Restrictive deficit on pulmonary function testing |
| HP:0003731 | 130 | Quadriceps muscle weakness |

| Cluster | Cell function | Description | Phenotype\_coverage | Genes |
| --- | --- | --- | --- | --- |
| 130 | GO:0030239 | myofibril assembly | 84.61538 | NEB, CAPN3, ACTA1, TTN, CFL2, MYL2, MYPN, KLHL41, LMOD3 |
| 130 | GO:0048741 | skeletal muscle fiber development | 84.61538 | MYOD1, SELENON, ACTA1, LMOD3, KLHL41, RYR1, KLHL40 |
| 130 | GO:0014706 | striated muscle tissue development | 76.92308 | NEB, ACVR1, MYOD1, SELENON, ACTA1, TTN, CFL2, MYL2, LMNA, EMD, KLHL41, LMOD3, RYR1, MYH7, KLHL40, FOS |
| 130 | GO:0007517 | muscle organ development | 76.92308 | NEB, CAPN3, MYOD1, SELENON, ACTA1, TTN, CFL2, FHL1, MYL2, ITGA7, EMD, KLHL41, LMOD3, RYR1, MYH7, KLHL40, FOS |
| 130 | GO:0030049 | muscle filament sliding | 76.92308 | NEB, TTN, ACTA1, DES, MYL2, TPM2, MYH7, TPM3 |
| 130 | R-HSA-390522 | Striated Muscle Contraction | 76.92308 | DES, TTN, TPM2, ACTA1, TPM3, NEB, MYL2 |

---


---


---

# Cluster #23

| HPO | Cluster | Description |
| --- | --- | --- |
| HP:0001270 | 23 | Motor delay |
| HP:0002359 | 23 | Frequent falls |
| HP:0002355 | 23 | Difficulty walking |
| HP:0001290 | 23 | Generalized hypotonia |
| HP:0002515 | 23 | Waddling gait |
| HP:0001265 | 23 | Hyporeflexia |
| HP:0009046 | 23 | Difficulty running |
| HP:0001284 | 23 | Areflexia |
| HP:0002375 | 23 | Hypokinesia |
| HP:0002058 | 23 | Myopathic facies |
| HP:0003391 | 23 | Gowers sign |
| HP:0003557 | 23 | Increased variability in muscle fiber diameter |
| HP:0001558 | 23 | Decreased fetal movement |
| HP:0003306 | 23 | Spinal rigidity |
| HP:0000467 | 23 | Neck muscle weakness |
| HP:0001349 | 23 | Facial diplegia |
| HP:0003551 | 23 | Difficulty climbing stairs |
| HP:0003803 | 23 | Type 1 muscle fiber predominance |
| HP:0003458 | 23 | EMG: myopathic abnormalities |

| Cluster | Cell function | Description | Phenotype\_coverage | Genes |
| --- | --- | --- | --- | --- |
| 23 | GO:0048747 | muscle fiber development | 84.21053 | NEB, MYOD1, SELENON, ACTA1, TTN, DYSF, MYF6, CHRNB1, STAC3, KLHL41, LMOD3, RYR1, SGCB, FLNC, KLHL40 |
| 23 | GO:0042692 | muscle cell differentiation | 78.94737 | TNNT1, MYOD1, MYPN, LMNA, MYF6, STAC3, KLHL41, LMOD3, FLNC, NEB, CAPN3, SELENON, TTN, SGCB, KLHL40, ACVR1, SPEG, CFL2, RYR1, ACTA1, BIN1, CHRNB1 |
| 23 | GO:0007517 | muscle organ development | 78.94737 | MYOD1, MYF6, STAC3, KLHL41, LMOD3, NEB, CAPN3, SELENON, TTN, CHRNA1, EMD, SGCB, KLHL40, SPEG, CFL2, FHL1, LAMA2, RYR1, MYH7, SGCA, FOS, ACTA1, CHRND, SGCG |
| 23 | GO:0014706 | striated muscle tissue development | 73.68421 | MYOD1, LMNA, MYF6, STAC3, KLHL41, LMOD3, NEB, SELENON, TTN, CHRNA1, EMD, SGCB, KLHL40, ACVR1, CFL2, RYR1, MYH7, FOS, ACTA1, CHRND, SGCG |

---


---


---

# Cluster #134

| HPO | Cluster | Description |
| --- | --- | --- |
| HP:0001270 | 134 | Motor delay |
| HP:0002355 | 134 | Difficulty walking |
| HP:0002515 | 134 | Waddling gait |
| HP:0001265 | 134 | Hyporeflexia |
| HP:0001284 | 134 | Areflexia |
| HP:0003391 | 134 | Gowers sign |
| HP:0003557 | 134 | Increased variability in muscle fiber diameter |
| HP:0003306 | 134 | Spinal rigidity |
| HP:0000467 | 134 | Neck muscle weakness |
| HP:0003236 | 134 | Elevated serum creatine kinase |
| HP:0002375 | 134 | Hypokinesia |
| HP:0001558 | 134 | Decreased fetal movement |
| HP:0001349 | 134 | Facial diplegia |
| HP:0003803 | 134 | Type 1 muscle fiber predominance |
| HP:0003798 | 134 | Nemaline bodies |
| HP:0003403 | 134 | EMG: decremental response of compound muscle action potential to repetitive nerve stimulation |
| HP:0003458 | 134 | EMG: myopathic abnormalities |
| HP:0003388 | 134 | Easy fatigability |
| HP:0003805 | 134 | Rimmed vacuoles |

| Cluster | Cell function | Description | Phenotype\_coverage | Genes |
| --- | --- | --- | --- | --- |
| 134 | GO:0006936 | muscle contraction | 84.21053 | NEB, MYOT, CHRNE, TNNT1, ACTA1, TTN, DMD, CHRNA1, TPM2, CHRND, SCN4A, BIN1, CHRNB1, EMD, KLHL41, LMOD3, RYR1, MYH7, PABPN1, TPM3 |
| 134 | GO:0007517 | muscle organ development | 78.94737 | MYOD1, MYF6, KLHL41, LMOD3, NEB, CAPN3, SELENON, TTN, CHRNA1, EMD, SGCB, FKTN, KLHL40, SPEG, CFL2, FHL1, DMD, LAMA2, RYR1, MYH7, SGCA, FOS, ACTA1, CHRND, SGCG, LARGE1 |
| 134 | GO:0014706 | striated muscle tissue development | 73.68421 | NEB, ACVR1, MYOD1, SELENON, ACTA1, TTN, CFL2, CHRNA1, CHRND, LMNA, MYF6, SGCG, EMD, KLHL41, LMOD3, RYR1, SGCB, MYH7, KLHL40, FOS |
| 134 | GO:0048747 | muscle fiber development | 73.68421 | NEB, MYOD1, SELENON, ACTA1, TTN, DMD, MYF6, CHRNB1, KLHL41, LMOD3, RYR1, SGCB, KLHL40 |

---


---


---

# Cluster #97

| HPO | Cluster | Description |
| --- | --- | --- |
| HP:0003236 | 97 | Elevated serum creatine kinase |
| HP:0003325 | 97 | Limb-girdle muscle weakness |
| HP:0030234 | 97 | Highly elevated creatine kinase |
| HP:0030046 | 97 | Hypoglycosylation of alpha-dystroglycan |
| HP:0003707 | 97 | Calf muscle pseudohypertrophy |
| HP:0002350 | 97 | Cerebellar cyst |

| Cluster | Cell function | Description | Phenotype\_coverage | Genes |
| --- | --- | --- | --- | --- |
| 97 | GO:0097502 | mannosylation | 83.33333 | POMT1, ALG2, FKRP, CRPPA, LARGE1, FKTN, POMT2 |

---


---


---

# Cluster #117

| HPO | Cluster | Description |
| --- | --- | --- |
| HP:0001270 | 117 | Motor delay |
| HP:0001265 | 117 | Hyporeflexia |
| HP:0001324 | 117 | Muscle weakness |
| HP:0001284 | 117 | Areflexia |
| HP:0002375 | 117 | Hypokinesia |
| HP:0001276 | 117 | Hypertonia |
| HP:0001561 | 117 | Polyhydramnios |
| HP:0000298 | 117 | Mask-like facies |
| HP:0002804 | 117 | Arthrogryposis multiplex congenita |
| HP:0002058 | 117 | Myopathic facies |
| HP:0001558 | 117 | Decreased fetal movement |
| HP:0003306 | 117 | Spinal rigidity |
| HP:0001349 | 117 | Facial diplegia |
| HP:0003803 | 117 | Type 1 muscle fiber predominance |
| HP:0001623 | 117 | Breech presentation |
| HP:0003798 | 117 | Nemaline bodies |
| HP:0005855 | 117 | Multiple prenatal fractures |
| HP:0001989 | 117 | Fetal akinesia sequence |

| Cluster | Cell function | Description | Phenotype\_coverage | Genes |
| --- | --- | --- | --- | --- |
| 117 | GO:0048741 | skeletal muscle fiber development | 83.33333 | MYOD1, ACTA1, SELENON, MYF6, STAC3, LMOD3, KLHL41, RYR1, KLHL40 |
| 117 | GO:0030239 | myofibril assembly | 77.77778 | NEB, CAPN3, TNNT1, ACTA1, TTN, CFL2, MYPN, KLHL41, LMOD3 |
| 117 | GO:0030049 | muscle filament sliding | 77.77778 | NEB, TNNT1, ACTA1, TTN, TPM2, TPM3 |
| 117 | R-HSA-390522 | Striated Muscle Contraction | 77.77778 | TTN, TPM2, ACTA1, TPM3, TNNT1, NEB |
| 117 | GO:0007015 | actin filament organization | 72.22222 | NEB, ACTA1, TTN, CFL2, TPM2, BIN1, LMOD3, TPM3 |

---


---


---

# Cluster #120

| HPO | Cluster | Description |
| --- | --- | --- |
| HP:0001270 | 120 | Motor delay |
| HP:0002515 | 120 | Waddling gait |
| HP:0001561 | 120 | Polyhydramnios |
| HP:0001638 | 120 | Cardiomyopathy |
| HP:0003306 | 120 | Spinal rigidity |
| HP:0003327 | 120 | Axial muscle weakness |
| HP:0000467 | 120 | Neck muscle weakness |
| HP:0003403 | 120 | EMG: decremental response of compound muscle action potential to repetitive nerve stimulation |
| HP:0003691 | 120 | Scapular winging |
| HP:0002747 | 120 | Respiratory insufficiency due to muscle weakness |
| HP:0003458 | 120 | EMG: myopathic abnormalities |
| HP:0008180 | 120 | Mildly elevated creatine kinase |
| HP:0001623 | 120 | Breech presentation |
| HP:0001989 | 120 | Fetal akinesia sequence |
| HP:0002792 | 120 | Reduced vital capacity |
| HP:0030192 | 120 | Fatigable weakness of bulbar muscles |
| HP:0011807 | 120 | Type 1 muscle fiber atrophy |
| HP:0012785 | 120 | Flexion contracture of finger |

| Cluster | Cell function | Description | Phenotype\_coverage | Genes |
| --- | --- | --- | --- | --- |
| 120 | GO:0030049 | muscle filament sliding | 83.33333 | NEB, TNNT1, ACTA1, TTN, DES, MYL2, TPM2, MYH7, TPM3 |
| 120 | GO:0048747 | muscle fiber development | 83.33333 | NEB, MYOD1, SELENON, ACTA1, TTN, MYL2, MYF6, CHRNB1, KLHL41, LMOD3, RYR1, KLHL40 |
| 120 | R-HSA-390522 | Striated Muscle Contraction | 83.33333 | DES, TTN, TPM2, ACTA1, TPM3, TNNT1, NEB, MYL2 |
| 120 | hsa05410 | Hypertrophic cardiomyopathy (HCM) | 77.77778 | CACNA1S, MYL2, DES, TTN, ITGB4, TPM3, TPM2, EMD, ITGA7, LMNA, MYH7 |
| 120 | hsa05414 | Dilated cardiomyopathy | 77.77778 | CACNA1S, MYL2, DES, TTN, ITGB4, TPM3, TPM2, EMD, ITGA7, LMNA, MYH7 |
| 120 | hsa04260 | Cardiac muscle contraction | 72.22222 | CACNA1S, MYL2, TPM3, TPM2, MYH7 |
| 120 | GO:0007517 | muscle organ development | 72.22222 | MYOD1, MYF6, KLHL41, LMOD3, NEB, CAPN3, SELENON, TTN, CHRNA1, EMD, FLNB, KLHL40, CFL2, FHL1, ITGA7, RYR1, MYH7, FOS, ACTA1, CHRND, MYL2 |
| 120 | GO:0014706 | striated muscle tissue development | 72.22222 | NEB, ACVR1, MYOD1, SELENON, ACTA1, CFL2, MYL2, TTN, CHRND, CHRNA1, LMNA, MYF6, EMD, KLHL41, LMOD3, RYR1, FLNB, MYH7, KLHL40, FOS |
| 120 | GO:0030239 | myofibril assembly | 72.22222 | NEB, CAPN3, TNNT1, ACTA1, TTN, CFL2, MYL2, MYPN, LMOD3, KLHL41 |

---


---


---

# Cluster #148

| HPO | Cluster | Description |
| --- | --- | --- |
| HP:0002515 | 148 | Waddling gait |
| HP:0003236 | 148 | Elevated serum creatine kinase |
| HP:0001561 | 148 | Polyhydramnios |
| HP:0003307 | 148 | Hyperlordosis |
| HP:0001558 | 148 | Decreased fetal movement |
| HP:0003306 | 148 | Spinal rigidity |
| HP:0001349 | 148 | Facial diplegia |
| HP:0003798 | 148 | Nemaline bodies |
| HP:0011807 | 148 | Type 1 muscle fiber atrophy |
| HP:0001239 | 148 | Wrist flexion contracture |
| HP:0003691 | 148 | Scapular winging |
| HP:0012548 | 148 | Fatty replacement of skeletal muscle |

| Cluster | Cell function | Description | Phenotype\_coverage | Genes |
| --- | --- | --- | --- | --- |
| 148 | GO:0007517 | muscle organ development | 83.33333 | KLHL41, LMOD3, NEB, CAPN3, SELENON, TTN, EMD, FLNB, FKTN, KLHL40, SPEG, DMD, CFL2, FHL1, ITGA7, RYR1, MYH7, FOS, ACTA1, MYL2, LARGE1 |
| 148 | GO:0030049 | muscle filament sliding | 83.33333 | NEB, ACTA1, TTN, DMD, MYL2, TPM2, MYH7, TPM3 |
| 148 | GO:0048747 | muscle fiber development | 83.33333 | NEB, SELENON, TTN, ACTA1, DMD, MYL2, KLHL41, LMOD3, RYR1, KLHL40 |
| 148 | R-HSA-390522 | Striated Muscle Contraction | 83.33333 | DMD, TTN, TPM2, ACTA1, TPM3, NEB, MYL2 |
| 148 | GO:0014706 | striated muscle tissue development | 75.00000 | NEB, ACVR1, SELENON, ACTA1, CFL2, MYL2, LMNA, EMD, KLHL41, LMOD3, RYR1, FLNB, KLHL40, FOS |

---


---


---

# Cluster #15

| HPO | Cluster | Description |
| --- | --- | --- |
| HP:0003198 | 15 | Myopathy |
| HP:0001270 | 15 | Motor delay |
| HP:0002355 | 15 | Difficulty walking |
| HP:0001284 | 15 | Areflexia |
| HP:0000602 | 15 | Ophthalmoplegia |
| HP:0002804 | 15 | Arthrogryposis multiplex congenita |
| HP:0002058 | 15 | Myopathic facies |
| HP:0003701 | 15 | Proximal muscle weakness |
| HP:0001558 | 15 | Decreased fetal movement |
| HP:0001349 | 15 | Facial diplegia |
| HP:0003691 | 15 | Scapular winging |
| HP:0003803 | 15 | Type 1 muscle fiber predominance |
| HP:0002747 | 15 | Respiratory insufficiency due to muscle weakness |
| HP:0003458 | 15 | EMG: myopathic abnormalities |
| HP:0003798 | 15 | Nemaline bodies |
| HP:0005855 | 15 | Multiple prenatal fractures |
| HP:0003388 | 15 | Easy fatigability |

| Cluster | Cell function | Description | Phenotype\_coverage | Genes |
| --- | --- | --- | --- | --- |
| 15 | GO:0048741 | skeletal muscle fiber development | 82.35294 | ACTA1, MYF6, STAC3, LMOD3, KLHL41, RYR1, KLHL40 |
| 15 | GO:0030239 | myofibril assembly | 70.58824 | NEB, TNNT1, ACTA1, TTN, CFL2, MYL2, MYPN, LMOD3, KLHL41 |
| 15 | GO:0030049 | muscle filament sliding | 70.58824 | NEB, TNNT1, ACTA1, TTN, MYL2, TPM2, MYH7, TPM3 |
| 15 | R-HSA-397014 | Muscle contraction | 70.58824 | TTN, TPM2, RYR1, ACTA1, TPM3, SCN4A, TNNT1, NEB, MYL2, KCNJ11 |
| 15 | R-HSA-390522 | Striated Muscle Contraction | 70.58824 | TTN, TPM2, ACTA1, TPM3, TNNT1, NEB, MYL2 |

---


---


---

# Cluster #82

| HPO | Cluster | Description |
| --- | --- | --- |
| HP:0001270 | 82 | Motor delay |
| HP:0008981 | 82 | Calf muscle hypertrophy |
| HP:0003236 | 82 | Elevated serum creatine kinase |
| HP:0002058 | 82 | Myopathic facies |
| HP:0003557 | 82 | Increased variability in muscle fiber diameter |
| HP:0003701 | 82 | Proximal muscle weakness |
| HP:0003803 | 82 | Type 1 muscle fiber predominance |
| HP:0003458 | 82 | EMG: myopathic abnormalities |
| HP:0009025 | 82 | Increased connective tissue |
| HP:0031237 | 82 | Internally nucleated skeletal muscle fibers |
| HP:0011807 | 82 | Type 1 muscle fiber atrophy |

| Cluster | Cell function | Description | Phenotype\_coverage | Genes |
| --- | --- | --- | --- | --- |
| 82 | hsa05410 | Hypertrophic cardiomyopathy (HCM) | 81.81818 | SGCB, MYL2, TTN, LAMA2, TPM3, TPM2, EMD, ITGA7, DMD, LMNA, MYH7 |
| 82 | hsa05414 | Dilated cardiomyopathy | 81.81818 | SGCB, MYL2, TTN, LAMA2, TPM3, TPM2, EMD, ITGA7, DMD, LMNA, MYH7 |
| 82 | GO:0007517 | muscle organ development | 81.81818 | MYF6, STAC3, KLHL41, LMOD3, NEB, TTN, CHRNA1, SELENON, EMD, SGCB, FKTN, KLHL40, SPEG, DMD, CFL2, FHL1, ITGA7, LAMA2, RYR1, MYH7, ACTA1, CHRND, MYL2, LARGE1 |
| 82 | GO:0030049 | muscle filament sliding | 72.72727 | NEB, TNNT1, ACTA1, TTN, DMD, MYL2, TPM2, MYH7, TPM3 |
| 82 | GO:0048741 | skeletal muscle fiber development | 72.72727 | ACTA1, MYF6, STAC3, KLHL41, LMOD3, RYR1, KLHL40 |
| 82 | GO:0014706 | striated muscle tissue development | 72.72727 | NEB, ACTA1, TTN, CHRNA1, CFL2, CHRND, SELENON, MYL2, LMNA, MYF6, STAC3, EMD, KLHL41, LMOD3, SGCB, RYR1, MYH7, KLHL40 |
| 82 | R-HSA-390522 | Striated Muscle Contraction | 72.72727 | DMD, TTN, TPM2, ACTA1, TPM3, TNNT1, NEB, MYL2 |

---


---


---

# Cluster #33

| HPO | Cluster | Description |
| --- | --- | --- |
| HP:0001270 | 33 | Motor delay |
| HP:0002355 | 33 | Difficulty walking |
| HP:0003236 | 33 | Elevated serum creatine kinase |
| HP:0002375 | 33 | Hypokinesia |
| HP:0001561 | 33 | Polyhydramnios |
| HP:0002058 | 33 | Myopathic facies |
| HP:0003701 | 33 | Proximal muscle weakness |
| HP:0001558 | 33 | Decreased fetal movement |
| HP:0001349 | 33 | Facial diplegia |
| HP:0003691 | 33 | Scapular winging |
| HP:0003803 | 33 | Type 1 muscle fiber predominance |
| HP:0003458 | 33 | EMG: myopathic abnormalities |
| HP:0003798 | 33 | Nemaline bodies |
| HP:0005855 | 33 | Multiple prenatal fractures |
| HP:0009025 | 33 | Increased connective tissue |
| HP:0003547 | 33 | Shoulder girdle muscle weakness |

| Cluster | Cell function | Description | Phenotype\_coverage | Genes |
| --- | --- | --- | --- | --- |
| 33 | GO:0048741 | skeletal muscle fiber development | 81.25 | MYOD1, ACTA1, MYF6, STAC3, LMOD3, KLHL41, RYR1, KLHL40 |
| 33 | GO:0030049 | muscle filament sliding | 75.00 | NEB, TNNT1, ACTA1, TTN, DMD, TPM2, MYH7, TPM3 |
| 33 | GO:0030240 | skeletal muscle thin filament assembly | 75.00 | LMOD3, ACTA1, TTN |
| 33 | R-HSA-390522 | Striated Muscle Contraction | 75.00 | DMD, TTN, TPM2, ACTA1, TPM3, TNNT1, NEB |

---


---


---

# Cluster #45

| HPO | Cluster | Description |
| --- | --- | --- |
| HP:0001270 | 45 | Motor delay |
| HP:0002355 | 45 | Difficulty walking |
| HP:0001436 | 45 | Abnormality of the foot musculature |
| HP:0009027 | 45 | Foot dorsiflexor weakness |
| HP:0031374 | 45 | Ankle weakness |
| HP:0001290 | 45 | Generalized hypotonia |
| HP:0001265 | 45 | Hyporeflexia |
| HP:0008994 | 45 | Proximal muscle weakness in lower limbs |
| HP:0001644 | 45 | Dilated cardiomyopathy |
| HP:0003557 | 45 | Increased variability in muscle fiber diameter |
| HP:0001558 | 45 | Decreased fetal movement |
| HP:0000467 | 45 | Neck muscle weakness |
| HP:0001349 | 45 | Facial diplegia |
| HP:0003687 | 45 | Centrally nucleated skeletal muscle fibers |
| HP:0003691 | 45 | Scapular winging |
| HP:0003803 | 45 | Type 1 muscle fiber predominance |
| HP:0002747 | 45 | Respiratory insufficiency due to muscle weakness |
| HP:0003458 | 45 | EMG: myopathic abnormalities |
| HP:0008180 | 45 | Mildly elevated creatine kinase |
| HP:0008997 | 45 | Proximal muscle weakness in upper limbs |
| HP:0002792 | 45 | Reduced vital capacity |
| HP:0008954 | 45 | Intrinsic hand muscle atrophy |
| HP:0003805 | 45 | Rimmed vacuoles |
| HP:0012548 | 45 | Fatty replacement of skeletal muscle |
| HP:0003547 | 45 | Shoulder girdle muscle weakness |
| HP:0009077 | 45 | Weakness of long finger extensor muscles |

| Cluster | Cell function | Description | Phenotype\_coverage | Genes |
| --- | --- | --- | --- | --- |
| 45 | GO:0006936 | muscle contraction | 80.76923 | CHRNE, TNNT1, CACNA1S, KLHL41, LMOD3, NEB, TTN, CHRNA1, TPM2, CASQ1, EMD, MYH14, SCN4A, RYR1, MYH7, MYOT, ACTA1, CHRND, MYL2, BIN1, CHRNB1, TAZ, PABPN1, TPM3 |

---


---


---

# Cluster #118

| HPO | Cluster | Description |
| --- | --- | --- |
| HP:0001270 | 118 | Motor delay |
| HP:0002355 | 118 | Difficulty walking |
| HP:0001290 | 118 | Generalized hypotonia |
| HP:0002515 | 118 | Waddling gait |
| HP:0001265 | 118 | Hyporeflexia |
| HP:0008956 | 118 | Proximal lower limb amyotrophy |
| HP:0008994 | 118 | Proximal muscle weakness in lower limbs |
| HP:0003236 | 118 | Elevated serum creatine kinase |
| HP:0001644 | 118 | Dilated cardiomyopathy |
| HP:0003557 | 118 | Increased variability in muscle fiber diameter |
| HP:0001558 | 118 | Decreased fetal movement |
| HP:0003306 | 118 | Spinal rigidity |
| HP:0000467 | 118 | Neck muscle weakness |
| HP:0001349 | 118 | Facial diplegia |
| HP:0003691 | 118 | Scapular winging |
| HP:0003803 | 118 | Type 1 muscle fiber predominance |
| HP:0002747 | 118 | Respiratory insufficiency due to muscle weakness |
| HP:0003458 | 118 | EMG: myopathic abnormalities |
| HP:0008997 | 118 | Proximal muscle weakness in upper limbs |
| HP:0003798 | 118 | Nemaline bodies |
| HP:0001989 | 118 | Fetal akinesia sequence |
| HP:0003388 | 118 | Easy fatigability |
| HP:0003805 | 118 | Rimmed vacuoles |
| HP:0002987 | 118 | Elbow flexion contracture |
| HP:0011807 | 118 | Type 1 muscle fiber atrophy |
| HP:0006785 | 118 | Limb-girdle muscular dystrophy |
| HP:0008948 | 118 | Proximal upper limb amyotrophy |
| HP:0001771 | 118 | Achilles tendon contracture |
| HP:0004631 | 118 | Decreased cervical spine flexion due to contractures of posterior cervical muscles |
| HP:0001678 | 118 | Atrioventricular block |
| HP:0002486 | 118 | Myotonia |

| Cluster | Cell function | Description | Phenotype\_coverage | Genes |
| --- | --- | --- | --- | --- |
| 118 | hsa05410 | Hypertrophic cardiomyopathy (HCM) | 80.64516 | SGCD, SGCG, DAG1, MYL2, TTN, TPM3, TPM2, EMD, ITGA7, SGCA, DMD, LMNA, MYH7 |
| 118 | hsa05414 | Dilated cardiomyopathy | 80.64516 | SGCD, SGCG, DAG1, MYL2, TTN, TPM3, TPM2, EMD, ITGA7, SGCA, DMD, LMNA, MYH7 |
| 118 | GO:0007517 | muscle organ development | 74.19355 | MYOD1, MYF6, KLHL41, LMOD3, NEB, SGCD, CAPN3, SELENON, TTN, CHRNA1, EMD, FKTN, KLHL40, SPEG, FHL1, DMD, CFL2, ITGA7, RYR1, MYH7, SGCA, FOS, ACTA1, CHRND, MYL2, SGCG, LARGE1, TAZ |
| 118 | GO:0006936 | muscle contraction | 74.19355 | CHRNE, TNNT1, KLHL41, LMOD3, CLCN1, NEB, SGCD, TTN, CHRNA1, DMPK, TPM2, EMD, DMD, SCN4A, RYR1, MYH7, MYOT, ACTA1, CHRND, MYL2, BIN1, CHRNB1, TAZ, PABPN1, TPM3 |
| 118 | GO:0014706 | striated muscle tissue development | 70.96774 | MYOD1, LMNA, MYF6, KLHL41, LMOD3, NEB, SGCD, SELENON, TTN, CHRNA1, EMD, KLHL40, ACVR1, CFL2, RYR1, MYH7, FOS, ACTA1, CHRND, MYL2, SGCG, TAZ |

---


---


---

# Cluster #5

| HPO | Cluster | Description |
| --- | --- | --- |
| HP:0001270 | 5 | Motor delay |
| HP:0001290 | 5 | Generalized hypotonia |
| HP:0009046 | 5 | Difficulty running |
| HP:0008981 | 5 | Calf muscle hypertrophy |
| HP:0003236 | 5 | Elevated serum creatine kinase |
| HP:0003391 | 5 | Gowers sign |
| HP:0001349 | 5 | Facial diplegia |
| HP:0003803 | 5 | Type 1 muscle fiber predominance |
| HP:0003388 | 5 | Easy fatigability |
| HP:0003547 | 5 | Shoulder girdle muscle weakness |

| Cluster | Cell function | Description | Phenotype\_coverage | Genes |
| --- | --- | --- | --- | --- |
| 5 | GO:0007517 | muscle organ development | 80 | NEB, SELENON, ACTA1, SPEG, TTN, DMD, CFL2, CHRNA1, CHRND, MYF6, SGCG, KLHL41, LMOD3, RYR1, SGCB, LARGE1, MYH7, SGCA, FKTN, KLHL40 |
| 5 | GO:0048747 | muscle fiber development | 80 | NEB, SELENON, ACTA1, TTN, DMD, DYSF, MYF6, CHRNB1, KLHL41, LMOD3, RYR1, SGCB, FLNC, KLHL40 |
| 5 | GO:0006936 | muscle contraction | 70 | NEB, MYOT, CHRNE, TNNT1, ACTA1, TTN, DMD, CHRNA1, TPM2, CHRND, BIN1, CHRNB1, KLHL41, LMOD3, RYR1, MYH7, TPM3 |

---


---


---

# Cluster #11

| HPO | Cluster | Description |
| --- | --- | --- |
| HP:0001270 | 11 | Motor delay |
| HP:0002359 | 11 | Frequent falls |
| HP:0040083 | 11 | Toe walking |
| HP:0009027 | 11 | Foot dorsiflexor weakness |
| HP:0002515 | 11 | Waddling gait |
| HP:0009046 | 11 | Difficulty running |
| HP:0003236 | 11 | Elevated serum creatine kinase |
| HP:0003391 | 11 | Gowers sign |
| HP:0003557 | 11 | Increased variability in muscle fiber diameter |
| HP:0003307 | 11 | Hyperlordosis |
| HP:0003306 | 11 | Spinal rigidity |
| HP:0001349 | 11 | Facial diplegia |
| HP:0003551 | 11 | Difficulty climbing stairs |
| HP:0003691 | 11 | Scapular winging |
| HP:0003803 | 11 | Type 1 muscle fiber predominance |
| HP:0003458 | 11 | EMG: myopathic abnormalities |
| HP:0006785 | 11 | Limb-girdle muscular dystrophy |
| HP:0012548 | 11 | Fatty replacement of skeletal muscle |
| HP:0001771 | 11 | Achilles tendon contracture |
| HP:0003547 | 11 | Shoulder girdle muscle weakness |

| Cluster | Cell function | Description | Phenotype\_coverage | Genes |
| --- | --- | --- | --- | --- |
| 11 | GO:0007517 | muscle organ development | 80 | MYF6, KLHL41, LMOD3, NEB, CAPN3, TTN, SELENON, CHRNA1, EMD, SGCB, FKTN, KLHL40, SPEG, FHL1, DMD, CFL2, RYR1, MYH7, SGCA, FOS, ACTA1, CHRND, SGCG, LARGE1 |
| 11 | hsa05410 | Hypertrophic cardiomyopathy (HCM) | 70 | SGCB, SGCG, DAG1, TTN, TPM3, TPM2, EMD, SGCA, DMD, LMNA, MYH7 |
| 11 | hsa05414 | Dilated cardiomyopathy | 70 | SGCB, SGCG, DAG1, TTN, TPM3, TPM2, EMD, SGCA, DMD, LMNA, MYH7 |
| 11 | GO:0048747 | muscle fiber development | 70 | NEB, TTN, ACTA1, SELENON, DMD, DYSF, MYF6, CHRNB1, KLHL41, LMOD3, RYR1, SGCB, FLNC, KLHL40 |

---


---


---

# Cluster #17

| HPO | Cluster | Description |
| --- | --- | --- |
| HP:0001290 | 17 | Generalized hypotonia |
| HP:0001284 | 17 | Areflexia |
| HP:0010628 | 17 | Facial palsy |
| HP:0003557 | 17 | Increased variability in muscle fiber diameter |
| HP:0003306 | 17 | Spinal rigidity |
| HP:0000467 | 17 | Neck muscle weakness |
| HP:0002747 | 17 | Respiratory insufficiency due to muscle weakness |
| HP:0003458 | 17 | EMG: myopathic abnormalities |
| HP:0008180 | 17 | Mildly elevated creatine kinase |
| HP:0003690 | 17 | Limb muscle weakness |

| Cluster | Cell function | Description | Phenotype\_coverage | Genes |
| --- | --- | --- | --- | --- |
| 17 | GO:0048741 | skeletal muscle fiber development | 80 | SELENON, SIX1, ACTA1, MYF6, KLHL41, RYR1 |
| 17 | GO:0014706 | striated muscle tissue development | 70 | NEB, ACVR1, SELENON, SIX1, EYA1, TTN, ACTA1, CFL2, MYL2, LMNA, MYF6, EMD, KLHL41, RYR1, MYH7, FOS |
| 17 | GO:0030239 | myofibril assembly | 70 | NEB, CAPN3, TNNT1, TTN, ACTA1, CFL2, MYL2, MYPN, KLHL41 |
| 17 | GO:0006936 | muscle contraction | 70 | NEB, TNNT1, MYOT, TTN, ACTA1, MYL2, TPM2, SCN4A, CACNA1S, BIN1, EMD, KLHL41, RYR1, MYH7, TPM3 |

---


---


---

# Cluster #38

| HPO | Cluster | Description |
| --- | --- | --- |
| HP:0001270 | 38 | Motor delay |
| HP:0002515 | 38 | Waddling gait |
| HP:0001265 | 38 | Hyporeflexia |
| HP:0001644 | 38 | Dilated cardiomyopathy |
| HP:0001315 | 38 | Reduced tendon reflexes |
| HP:0000602 | 38 | Ophthalmoplegia |
| HP:0002058 | 38 | Myopathic facies |
| HP:0003324 | 38 | Generalized muscle weakness |
| HP:0003701 | 38 | Proximal muscle weakness |
| HP:0002421 | 38 | Poor head control |
| HP:0001558 | 38 | Decreased fetal movement |
| HP:0003306 | 38 | Spinal rigidity |
| HP:0000467 | 38 | Neck muscle weakness |
| HP:0003687 | 38 | Centrally nucleated skeletal muscle fibers |
| HP:0003691 | 38 | Scapular winging |
| HP:0002747 | 38 | Respiratory insufficiency due to muscle weakness |
| HP:0003458 | 38 | EMG: myopathic abnormalities |
| HP:0008180 | 38 | Mildly elevated creatine kinase |
| HP:0003388 | 38 | Easy fatigability |
| HP:0001612 | 38 | Weak cry |

| Cluster | Cell function | Description | Phenotype\_coverage | Genes |
| --- | --- | --- | --- | --- |
| 38 | GO:0006936 | muscle contraction | 80 | CHRNE, TNNT1, CACNA1S, STAC3, KLHL41, NEB, TTN, CHRNA1, TPM2, CASQ1, EMD, SCN4A, RYR1, MYH7, MYOT, ACTA1, CHRND, MYL2, BIN1, CHRNB1, TAZ, TPM3 |
| 38 | GO:0048741 | skeletal muscle fiber development | 75 | SELENON, ACTA1, MYF6, STAC3, KLHL41, RYR1 |

---


---


---

# Cluster #67

| HPO | Cluster | Description |
| --- | --- | --- |
| HP:0010508 | 67 | Metatarsus valgus |
| HP:0000600 | 67 | Abnormality of the pharynx |
| HP:0009743 | 67 | Distichiasis |
| HP:0001621 | 67 | Weak voice |
| HP:0003044 | 67 | Shoulder flexion contracture |

| Cluster | Cell function | Description | Phenotype\_coverage | Genes |
| --- | --- | --- | --- | --- |
| 67 | R-HSA-3000157 | Laminin interactions | 80 | HSPG2, COL4A1 |

---


---


---

# Cluster #78

| HPO | Cluster | Description |
| --- | --- | --- |
| HP:0002359 | 78 | Frequent falls |
| HP:0001436 | 78 | Abnormality of the foot musculature |
| HP:0003236 | 78 | Elevated serum creatine kinase |
| HP:0003551 | 78 | Difficulty climbing stairs |
| HP:0003691 | 78 | Scapular winging |
| HP:0003555 | 78 | Muscle fiber splitting |
| HP:0031237 | 78 | Internally nucleated skeletal muscle fibers |
| HP:0002987 | 78 | Elbow flexion contracture |
| HP:0003730 | 78 | EMG: myotonic runs |
| HP:0012548 | 78 | Fatty replacement of skeletal muscle |
| HP:0100297 | 78 | Increased endomysial connective tissue |
| HP:0003547 | 78 | Shoulder girdle muscle weakness |
| HP:0030007 | 78 | EMG: positive sharp waves |
| HP:0100284 | 78 | EMG: myotonic discharges |
| HP:0006467 | 78 | Limited shoulder movement |

| Cluster | Cell function | Description | Phenotype\_coverage | Genes |
| --- | --- | --- | --- | --- |
| 78 | GO:0007517 | muscle organ development | 80 | CAPN3, TTN, ACTA1, DMD, SELENON, FHL1, MYL2, CHRND, CHRNA1, ITGA7, MYF6, SGCG, EMD, RYR1, COL6A3, LARGE1, MYH7, SGCA, FKTN |

---


---


---

# Cluster #89

| HPO | Cluster | Description |
| --- | --- | --- |
| HP:0003236 | 89 | Elevated serum creatine kinase |
| HP:0003551 | 89 | Difficulty climbing stairs |
| HP:0003691 | 89 | Scapular winging |
| HP:0003730 | 89 | EMG: myotonic runs |
| HP:0006785 | 89 | Limb-girdle muscular dystrophy |
| HP:0100297 | 89 | Increased endomysial connective tissue |
| HP:0025169 | 89 | Left ventricular systolic dysfunction |
| HP:0003707 | 89 | Calf muscle pseudohypertrophy |
| HP:0030007 | 89 | EMG: positive sharp waves |
| HP:0100284 | 89 | EMG: myotonic discharges |

| Cluster | Cell function | Description | Phenotype\_coverage | Genes |
| --- | --- | --- | --- | --- |
| 89 | hsa05410 | Hypertrophic cardiomyopathy (HCM) | 80 | SGCG, DAG1, TTN, TPM3, TPM2, EMD, SGCA, DMD, LMNA, MYH7 |
| 89 | hsa05414 | Dilated cardiomyopathy | 80 | SGCG, DAG1, TTN, TPM3, TPM2, EMD, SGCA, DMD, LMNA, MYH7 |
| 89 | GO:0007517 | muscle organ development | 80 | TTN, ACTA1, DMD, CAV3, SGCG, RYR1, COL6A3, LARGE1, MYH7, SGCA, FKTN |
| 89 | hsa05416 | Viral myocarditis | 70 | SGCG, DAG1, SGCA, DMD, MYH7 |

---


---


---

# Cluster #98

| HPO | Cluster | Description |
| --- | --- | --- |
| HP:0001290 | 98 | Generalized hypotonia |
| HP:0003200 | 98 | Ragged-red muscle fibers |
| HP:0003457 | 98 | EMG abnormality |
| HP:0003128 | 98 | Lactic acidosis |
| HP:0003737 | 98 | Mitochondrial myopathy |

| Cluster | Cell function | Description | Phenotype\_coverage | Genes |
| --- | --- | --- | --- | --- |
| 98 | GO:0032981 | mitochondrial respiratory chain complex I assembly | 80 | ND6, NDUFA11, ND3, TIMMDC1, NDUFV1, NDUFAF1, NUBPL, ND2, NDUFS1, NDUFAF5, NDUFB10, NDUFS6, NDUFA6, NDUFS3, ND1, NDUFAF3, NDUFAF4, NDUFAF2, NDUFB3, NDUFS7, NDUFS4, NDUFV2, FOXRED1, TMEM126B, NDUFB11, ACAD9, ND4, NDUFS8, NDUFB9, ND5, NDUFA1 |
| 98 | GO:0006120 | mitochondrial electron transport, NADH to ubiquinone | 80 | ND6, NDUFA11, ND3, NDUFV1, NDUFAF1, ND2, NDUFS1, NDUFB10, NDUFS6, DLD, NDUFA6, NDUFS3, ND1, NDUFB3, NDUFS7, NDUFS4, NDUFV2, NDUFB11, ND4, NDUFS8, NDUFB9, ND5, NDUFA1 |
| 98 | R-HSA-1428517 | The citric acid (TCA) cycle and respiratory electron transport | 80 | NDUFA1, NDUFB10, NDUFAF4, NDUFAF5, NDUFB3, ND3, NDUFB11, NUBPL, ATP8, NDUFS8, COX1, TIMMDC1, COX3, COX2, NDUFB9, ACAD9, TMEM126B, NDUFV1, NDUFV2, NDUFAF3, NDUFS3, NDUFAF1, DLD, ND2, ND4, NDUFS4, ND1, ND6, ND5, NDUFS6, NDUFA6, NDUFAF2, NDUFS7, NDUFS1, NDUFA11 |
| 98 | R-HSA-6799198 | Complex I biogenesis | 80 | NDUFA1, NDUFB10, NDUFAF4, NDUFAF5, NDUFB3, ND3, NDUFB11, NUBPL, NDUFS8, TIMMDC1, NDUFB9, ACAD9, TMEM126B, NDUFV1, NDUFV2, NDUFAF3, NDUFS3, NDUFAF1, ND2, ND4, NDUFS4, ND1, ND6, ND5, NDUFS6, NDUFA6, NDUFAF2, NDUFS7, NDUFS1, NDUFA11 |
| 98 | R-HSA-163200 | Respiratory electron transport, ATP synthesis by chemiosmotic coupling, and heat production by uncoupling proteins. | 80 | NDUFA1, NDUFB10, NDUFAF4, NDUFAF5, NDUFB3, ND3, NDUFB11, NUBPL, ATP8, NDUFS8, COX1, TIMMDC1, COX3, COX2, NDUFB9, ACAD9, TMEM126B, NDUFV1, NDUFV2, NDUFAF3, NDUFS3, NDUFAF1, ND2, ND4, NDUFS4, ND1, ND6, ND5, NDUFS6, NDUFA6, NDUFAF2, NDUFS7, NDUFS1, NDUFA11 |
| 98 | R-HSA-611105 | Respiratory electron transport | 80 | NDUFA1, NDUFB10, NDUFAF4, NDUFAF5, NDUFB3, ND3, NDUFB11, NUBPL, NDUFS8, COX1, TIMMDC1, COX3, COX2, NDUFB9, ACAD9, TMEM126B, NDUFV1, NDUFV2, NDUFAF3, NDUFS3, NDUFAF1, ND2, ND4, NDUFS4, ND1, ND6, ND5, NDUFS6, NDUFA6, NDUFAF2, NDUFS7, NDUFS1, NDUFA11 |

---


---


---

# Cluster #127

| HPO | Cluster | Description |
| --- | --- | --- |
| HP:0040083 | 127 | Toe walking |
| HP:0003236 | 127 | Elevated serum creatine kinase |
| HP:0003307 | 127 | Hyperlordosis |
| HP:0003306 | 127 | Spinal rigidity |
| HP:0003273 | 127 | Hip contracture |
| HP:0003691 | 127 | Scapular winging |
| HP:0003555 | 127 | Muscle fiber splitting |
| HP:0031237 | 127 | Internally nucleated skeletal muscle fibers |
| HP:0002987 | 127 | Elbow flexion contracture |
| HP:0006466 | 127 | Ankle flexion contracture |
| HP:0012785 | 127 | Flexion contracture of finger |
| HP:0001239 | 127 | Wrist flexion contracture |
| HP:0003089 | 127 | Hamstring contractures |
| HP:0003730 | 127 | EMG: myotonic runs |
| HP:0100297 | 127 | Increased endomysial connective tissue |

| Cluster | Cell function | Description | Phenotype\_coverage | Genes |
| --- | --- | --- | --- | --- |
| 127 | GO:0007517 | muscle organ development | 80 | KLHL41, NEB, CAPN3, TTN, SELENON, EMD, FKTN, SPEG, FHL1, DMD, CFL2, ITGA7, RYR1, COL6A3, MYH7, SGCA, FOS, ACTA1, MYL2, SGCG, LARGE1 |

---


---


---

# Cluster #133

| HPO | Cluster | Description |
| --- | --- | --- |
| HP:0003323 | 133 | Progressive muscle weakness |
| HP:0003273 | 133 | Hip contracture |
| HP:0001239 | 133 | Wrist flexion contracture |
| HP:0007126 | 133 | Proximal amyotrophy |
| HP:0003044 | 133 | Shoulder flexion contracture |

| Cluster | Cell function | Description | Phenotype\_coverage | Genes |
| --- | --- | --- | --- | --- |
| 133 | hsa04512 | ECM-receptor interaction | 80 | ITGA7, COL6A2, COL6A1, HSPG2 |
| 133 | GO:0045932 | negative regulation of muscle contraction | 80 | TNNT1, BIN1 |
| 133 | GO:0030049 | muscle filament sliding | 80 | TNNT1, ACTA1, DES, MYL2 |
| 133 | GO:0043501 | skeletal muscle adaptation | 80 | TNNT1, ACTA1 |
| 133 | GO:0030239 | myofibril assembly | 80 | TNNT1, ACTA1, MYL2 |
| 133 | R-HSA-390522 | Striated Muscle Contraction | 80 | DES, ACTA1, MYL2, TNNT1 |

---


---


---

# Cluster #16

| HPO | Cluster | Description |
| --- | --- | --- |
| HP:0001270 | 16 | Motor delay |
| HP:0002359 | 16 | Frequent falls |
| HP:0040083 | 16 | Toe walking |
| HP:0002355 | 16 | Difficulty walking |
| HP:0009027 | 16 | Foot dorsiflexor weakness |
| HP:0002515 | 16 | Waddling gait |
| HP:0001265 | 16 | Hyporeflexia |
| HP:0008994 | 16 | Proximal muscle weakness in lower limbs |
| HP:0009046 | 16 | Difficulty running |
| HP:0001284 | 16 | Areflexia |
| HP:0003236 | 16 | Elevated serum creatine kinase |
| HP:0003391 | 16 | Gowers sign |
| HP:0003557 | 16 | Increased variability in muscle fiber diameter |
| HP:0001558 | 16 | Decreased fetal movement |
| HP:0003306 | 16 | Spinal rigidity |
| HP:0001349 | 16 | Facial diplegia |
| HP:0003551 | 16 | Difficulty climbing stairs |
| HP:0003691 | 16 | Scapular winging |
| HP:0003803 | 16 | Type 1 muscle fiber predominance |
| HP:0003458 | 16 | EMG: myopathic abnormalities |
| HP:0003805 | 16 | Rimmed vacuoles |
| HP:0006785 | 16 | Limb-girdle muscular dystrophy |
| HP:0012548 | 16 | Fatty replacement of skeletal muscle |
| HP:0003547 | 16 | Shoulder girdle muscle weakness |

| Cluster | Cell function | Description | Phenotype\_coverage | Genes |
| --- | --- | --- | --- | --- |
| 16 | GO:0007517 | muscle organ development | 79.16667 | MYF6, KLHL41, LMOD3, NEB, CAPN3, TTN, SELENON, CHRNA1, EMD, SGCB, FKTN, KLHL40, SPEG, FHL1, DMD, CFL2, RYR1, MYH7, SGCA, FOS, ACTA1, CHRND, SGCG, LARGE1 |
| 16 | GO:0048747 | muscle fiber development | 70.83333 | NEB, TTN, ACTA1, SELENON, DMD, DYSF, MYF6, CHRNB1, KLHL41, LMOD3, RYR1, SGCB, FLNC, KLHL40 |

---


---


---

# Cluster #63

| HPO | Cluster | Description |
| --- | --- | --- |
| HP:0040083 | 63 | Toe walking |
| HP:0009027 | 63 | Foot dorsiflexor weakness |
| HP:0002515 | 63 | Waddling gait |
| HP:0008956 | 63 | Proximal lower limb amyotrophy |
| HP:0008994 | 63 | Proximal muscle weakness in lower limbs |
| HP:0003236 | 63 | Elevated serum creatine kinase |
| HP:0003307 | 63 | Hyperlordosis |
| HP:0003306 | 63 | Spinal rigidity |
| HP:0003691 | 63 | Scapular winging |
| HP:0008997 | 63 | Proximal muscle weakness in upper limbs |
| HP:0002792 | 63 | Reduced vital capacity |
| HP:0008954 | 63 | Intrinsic hand muscle atrophy |
| HP:0003805 | 63 | Rimmed vacuoles |
| HP:0002987 | 63 | Elbow flexion contracture |
| HP:0011807 | 63 | Type 1 muscle fiber atrophy |
| HP:0006785 | 63 | Limb-girdle muscular dystrophy |
| HP:0012548 | 63 | Fatty replacement of skeletal muscle |
| HP:0008948 | 63 | Proximal upper limb amyotrophy |
| HP:0001771 | 63 | Achilles tendon contracture |
| HP:0004631 | 63 | Decreased cervical spine flexion due to contractures of posterior cervical muscles |
| HP:0003547 | 63 | Shoulder girdle muscle weakness |
| HP:0001678 | 63 | Atrioventricular block |
| HP:0002486 | 63 | Myotonia |
| HP:0009077 | 63 | Weakness of long finger extensor muscles |

| Cluster | Cell function | Description | Phenotype\_coverage | Genes |
| --- | --- | --- | --- | --- |
| 63 | hsa05410 | Hypertrophic cardiomyopathy (HCM) | 79.16667 | SGCD, SGCG, DAG1, MYL2, TTN, TPM3, TPM2, EMD, ITGA7, SGCA, DMD, LMNA, MYH7 |
| 63 | hsa05414 | Dilated cardiomyopathy | 79.16667 | SGCD, SGCG, DAG1, MYL2, TTN, TPM3, TPM2, EMD, ITGA7, SGCA, DMD, LMNA, MYH7 |

---


---


---

# Cluster #101

| HPO | Cluster | Description |
| --- | --- | --- |
| HP:0002828 | 101 | Multiple joint contractures |
| HP:0001989 | 101 | Fetal akinesia sequence |
| HP:0002047 | 101 | Malignant hyperthermia |
| HP:0003700 | 101 | Generalized amyotrophy |
| HP:0001558 | 101 | Decreased fetal movement |
| HP:0003327 | 101 | Axial muscle weakness |
| HP:0002747 | 101 | Respiratory insufficiency due to muscle weakness |
| HP:0003325 | 101 | Limb-girdle muscle weakness |
| HP:0003552 | 101 | Muscle stiffness |

| Cluster | Cell function | Description | Phenotype\_coverage | Genes |
| --- | --- | --- | --- | --- |
| 101 | GO:0048741 | skeletal muscle fiber development | 77.77778 | MYOD1, ACTA1, MYF6, STAC3, KLHL41, LMOD3, RYR1, KLHL40 |

---


---


---

# Cluster #111

| HPO | Cluster | Description |
| --- | --- | --- |
| HP:0001265 | 111 | Hyporeflexia |
| HP:0000597 | 111 | Ophthalmoparesis |
| HP:0001324 | 111 | Muscle weakness |
| HP:0003200 | 111 | Ragged-red muscle fibers |
| HP:0001621 | 111 | Weak voice |
| HP:0003737 | 111 | Mitochondrial myopathy |
| HP:0003403 | 111 | EMG: decremental response of compound muscle action potential to repetitive nerve stimulation |
| HP:0003803 | 111 | Type 1 muscle fiber predominance |
| HP:0003388 | 111 | Easy fatigability |

| Cluster | Cell function | Description | Phenotype\_coverage | Genes |
| --- | --- | --- | --- | --- |
| 111 | GO:0035094 | response to nicotine | 77.77778 | CHRNE, CHRND, CHRNA1, ND6, ND4, CHRNB1 |

---


---


---

# Cluster #24

| HPO | Cluster | Description |
| --- | --- | --- |
| HP:0000014 | 24 | Abnormality of the bladder |
| HP:0002127 | 24 | Abnormal upper motor neuron morphology |
| HP:0002193 | 24 | Pseudobulbar behavioral symptoms |
| HP:0007354 | 24 | Amyotrophic lateral sclerosis |

| Cluster | Cell function | Description | Phenotype\_coverage | Genes |
| --- | --- | --- | --- | --- |
| 24 | hsa05014 | Amyotrophic lateral sclerosis (ALS) | 75 | SOD1, ALS2, PRPH, NEFH |

---


---


---

# Cluster #43

| HPO | Cluster | Description |
| --- | --- | --- |
| HP:0001270 | 43 | Motor delay |
| HP:0003236 | 43 | Elevated serum creatine kinase |
| HP:0002375 | 43 | Hypokinesia |
| HP:0001644 | 43 | Dilated cardiomyopathy |
| HP:0002878 | 43 | Respiratory failure |
| HP:0001638 | 43 | Cardiomyopathy |
| HP:0002058 | 43 | Myopathic facies |
| HP:0003557 | 43 | Increased variability in muscle fiber diameter |
| HP:0003546 | 43 | Exercise intolerance |
| HP:0001558 | 43 | Decreased fetal movement |
| HP:0001349 | 43 | Facial diplegia |
| HP:0003803 | 43 | Type 1 muscle fiber predominance |
| HP:0003458 | 43 | EMG: myopathic abnormalities |
| HP:0003798 | 43 | Nemaline bodies |
| HP:0005855 | 43 | Multiple prenatal fractures |
| HP:0009025 | 43 | Increased connective tissue |
| HP:0002792 | 43 | Reduced vital capacity |
| HP:0100297 | 43 | Increased endomysial connective tissue |
| HP:0003547 | 43 | Shoulder girdle muscle weakness |
| HP:0012515 | 43 | Hip flexor weakness |

| Cluster | Cell function | Description | Phenotype\_coverage | Genes |
| --- | --- | --- | --- | --- |
| 43 | GO:0007519 | skeletal muscle tissue development | 75 | MYOD1, ACTA1, CHRND, CFL2, CHRNA1, MYF6, STAC3, KLHL41, LMOD3, RYR1, KLHL40 |
| 43 | GO:0048747 | muscle fiber development | 75 | NEB, MYOD1, TTN, ACTA1, DMD, MYL2, MYF6, CHRNB1, STAC3, KLHL41, LMOD3, RYR1, SGCB, FLNC, KLHL40 |
| 43 | GO:0030049 | muscle filament sliding | 70 | NEB, TNNT1, ACTA1, TTN, DMD, MYL2, TPM2, MYH7, TPM3 |

---


---


---

# Cluster #51

| HPO | Cluster | Description |
| --- | --- | --- |
| HP:0007149 | 51 | Distal upper limb amyotrophy |
| HP:0003738 | 51 | Exercise-induced myalgia |
| HP:0003547 | 51 | Shoulder girdle muscle weakness |
| HP:0003731 | 51 | Quadriceps muscle weakness |

| Cluster | Cell function | Description | Phenotype\_coverage | Genes |
| --- | --- | --- | --- | --- |
| 51 | GO:0046033 | AMP metabolic process | 75 | AK9, AMPD3, AMPD1, ADSS1 |

---


---


---

# Cluster #57

| HPO | Cluster | Description |
| --- | --- | --- |
| HP:0001638 | 57 | Cardiomyopathy |
| HP:0030198 | 57 | Fatigable weakness of distal limb muscles |
| HP:0012722 | 57 | Heart block |
| HP:0011808 | 57 | Decreased patellar reflex |

| Cluster | Cell function | Description | Phenotype\_coverage | Genes |
| --- | --- | --- | --- | --- |
| 57 | hsa04260 | Cardiac muscle contraction | 75 | COX2, COX3, COX1, TPM2, MYH7 |

---


---


---

# Cluster #61

| HPO | Cluster | Description |
| --- | --- | --- |
| HP:0000544 | 61 | External ophthalmoplegia |
| HP:0001621 | 61 | Weak voice |
| HP:0001283 | 61 | Bulbar palsy |
| HP:0008049 | 61 | Abnormality of the extraocular muscles |

| Cluster | Cell function | Description | Phenotype\_coverage | Genes |
| --- | --- | --- | --- | --- |
| 61 | GO:0043097 | pyrimidine nucleoside salvage | 75 | TYMP, TK2 |
| 61 | R-HSA-73614 | Pyrimidine salvage | 75 | TYMP, TK2 |
| 61 | R-HSA-8956321 | Nucleotide salvage | 75 | TYMP, TK2 |

---


---


---

# Cluster #71

| HPO | Cluster | Description |
| --- | --- | --- |
| HP:0001436 | 71 | Abnormality of the foot musculature |
| HP:0009027 | 71 | Foot dorsiflexor weakness |
| HP:0031374 | 71 | Ankle weakness |
| HP:0001638 | 71 | Cardiomyopathy |
| HP:0003557 | 71 | Increased variability in muscle fiber diameter |
| HP:0003403 | 71 | EMG: decremental response of compound muscle action potential to repetitive nerve stimulation |
| HP:0003458 | 71 | EMG: myopathic abnormalities |
| HP:0002792 | 71 | Reduced vital capacity |
| HP:0012548 | 71 | Fatty replacement of skeletal muscle |
| HP:0100297 | 71 | Increased endomysial connective tissue |
| HP:0003547 | 71 | Shoulder girdle muscle weakness |
| HP:0012515 | 71 | Hip flexor weakness |
| HP:0009077 | 71 | Weakness of long finger extensor muscles |
| HP:0030007 | 71 | EMG: positive sharp waves |
| HP:0100284 | 71 | EMG: myotonic discharges |
| HP:0006467 | 71 | Limited shoulder movement |

| Cluster | Cell function | Description | Phenotype\_coverage | Genes |
| --- | --- | --- | --- | --- |
| 71 | GO:0006936 | muscle contraction | 75 | NEB, CHRNE, MYOT, TTN, CHRND, CHRNA1, ACTA1, TPM2, MYL2, SCN4A, MYH14, BIN1, CHRNB1, KLHL41, MYH7, SGCA, TPM3 |

---


---


---

# Cluster #96

| HPO | Cluster | Description |
| --- | --- | --- |
| HP:0012548 | 96 | Fatty replacement of skeletal muscle |
| HP:0012899 | 96 | Handgrip myotonia |
| HP:0003715 | 96 | Myofibrillar myopathy |
| HP:0010548 | 96 | Percussion myotonia |

| Cluster | Cell function | Description | Phenotype\_coverage | Genes |
| --- | --- | --- | --- | --- |
| 96 | GO:0060713 | labyrinthine layer morphogenesis | 75 | DNAJB6 |
| 96 | GO:0006457 | protein folding | 75 | VCP, DNAJB6 |
| 96 | GO:0090084 | negative regulation of inclusion body assembly | 75 | DNAJB6 |
| 96 | GO:0034605 | cellular response to heat | 75 | VCP, DNAJB6 |
| 96 | GO:0060706 | cell differentiation involved in embryonic placenta development | 75 | AKT1, DNAJB6 |
| 96 | GO:0045109 | intermediate filament organization | 75 | DNAJB6 |
| 96 | GO:0043281 | regulation of cysteine-type endopeptidase activity involved in apoptotic process | 75 | VCP, AKT1, DNAJB6 |

---


---


---

# Cluster #125

| HPO | Cluster | Description |
| --- | --- | --- |
| HP:0002093 | 125 | Respiratory insufficiency |
| HP:0001315 | 125 | Reduced tendon reflexes |
| HP:0012722 | 125 | Heart block |
| HP:0002401 | 125 | Stroke-like episode |

| Cluster | Cell function | Description | Phenotype\_coverage | Genes |
| --- | --- | --- | --- | --- |
| 125 | hsa04260 | Cardiac muscle contraction | 75 | COX2, COX3, TPM2, COX1, TPM3 |

---


---


---

# Cluster #138

| HPO | Cluster | Description |
| --- | --- | --- |
| HP:0001270 | 138 | Motor delay |
| HP:0002515 | 138 | Waddling gait |
| HP:0009046 | 138 | Difficulty running |
| HP:0003560 | 138 | Muscular dystrophy |
| HP:0003327 | 138 | Axial muscle weakness |
| HP:0001319 | 138 | Neonatal hypotonia |
| HP:0030200 | 138 | Fatiguable weakness of proximal limb muscles |
| HP:0003551 | 138 | Difficulty climbing stairs |
| HP:0003803 | 138 | Type 1 muscle fiber predominance |
| HP:0003458 | 138 | EMG: myopathic abnormalities |
| HP:0003798 | 138 | Nemaline bodies |
| HP:0007126 | 138 | Proximal amyotrophy |
| HP:0001771 | 138 | Achilles tendon contracture |
| HP:0003741 | 138 | Congenital muscular dystrophy |
| HP:0003797 | 138 | Limb-girdle muscle atrophy |
| HP:0012899 | 138 | Handgrip myotonia |

| Cluster | Cell function | Description | Phenotype\_coverage | Genes |
| --- | --- | --- | --- | --- |
| 138 | GO:0007517 | muscle organ development | 75 | MYF6, KLHL41, LMOD3, NEB, CAPN3, SELENON, TTN, CHRNA1, CASQ1, EMD, FKTN, KLHL40, SPEG, CFL2, FHL1, LAMA2, RYR1, MYH7, SGCA, ACTA1, CHRND, SGCG |

---


---


---

# Cluster #114

| HPO | Cluster | Description |
| --- | --- | --- |
| HP:0008800 | 114 | Limited hip movement |
| HP:0009027 | 114 | Foot dorsiflexor weakness |
| HP:0002913 | 114 | Myoglobinuria |
| HP:0003458 | 114 | EMG: myopathic abnormalities |
| HP:0002792 | 114 | Reduced vital capacity |
| HP:0008963 | 114 | Tibialis muscle weakness |
| HP:0003555 | 114 | Muscle fiber splitting |
| HP:0003805 | 114 | Rimmed vacuoles |
| HP:0008978 | 114 | Necrotizing myopathy |
| HP:0009045 | 114 | Exercise-induced rhabdomyolysis |
| HP:0031237 | 114 | Internally nucleated skeletal muscle fibers |

| Cluster | Cell function | Description | Phenotype\_coverage | Genes |
| --- | --- | --- | --- | --- |
| 114 | hsa05410 | Hypertrophic cardiomyopathy (HCM) | 72.72727 | SGCB, CACNA1S, MYL2, TTN, TPM3, TPM2, EMD, ITGA7, LMNA, MYH7 |
| 114 | hsa05414 | Dilated cardiomyopathy | 72.72727 | SGCB, CACNA1S, MYL2, TTN, TPM3, TPM2, EMD, ITGA7, LMNA, MYH7 |
| 114 | GO:0048747 | muscle fiber development | 72.72727 | NEB, TTN, ACTA1, MYL2, DYSF, CHRNB1, KLHL41, RYR1 |

---


---


---

# Cluster #14

| HPO | Cluster | Description |
| --- | --- | --- |
| HP:0001270 | 14 | Motor delay |
| HP:0002355 | 14 | Difficulty walking |
| HP:0002515 | 14 | Waddling gait |
| HP:0001265 | 14 | Hyporeflexia |
| HP:0009046 | 14 | Difficulty running |
| HP:0001284 | 14 | Areflexia |
| HP:0008981 | 14 | Calf muscle hypertrophy |
| HP:0003236 | 14 | Elevated serum creatine kinase |
| HP:0003557 | 14 | Increased variability in muscle fiber diameter |
| HP:0003701 | 14 | Proximal muscle weakness |
| HP:0003551 | 14 | Difficulty climbing stairs |
| HP:0003691 | 14 | Scapular winging |
| HP:0003803 | 14 | Type 1 muscle fiber predominance |
| HP:0003458 | 14 | EMG: myopathic abnormalities |
| HP:0002792 | 14 | Reduced vital capacity |
| HP:0003388 | 14 | Easy fatigability |
| HP:0012548 | 14 | Fatty replacement of skeletal muscle |
| HP:0003547 | 14 | Shoulder girdle muscle weakness |

| Cluster | Cell function | Description | Phenotype\_coverage | Genes |
| --- | --- | --- | --- | --- |
| 14 | GO:0006936 | muscle contraction | 72.22222 | NEB, MYOT, CHRNE, TNNT1, ACTA1, TTN, DMD, CHRNA1, TPM2, CHRND, SCN4A, MYL2, MYH14, BIN1, CHRNB1, KLHL41, LMOD3, RYR1, MYH7, TPM3 |
| 14 | GO:0048747 | muscle fiber development | 72.22222 | NEB, SELENON, ACTA1, TTN, DMD, DYSF, MYL2, MYF6, CHRNB1, KLHL41, LMOD3, RYR1, FLNC, KLHL40 |

---


---


---

# Cluster #77

| HPO | Cluster | Description |
| --- | --- | --- |
| HP:0003236 | 77 | Elevated serum creatine kinase |
| HP:0003458 | 77 | EMG: myopathic abnormalities |
| HP:0030198 | 77 | Fatigable weakness of distal limb muscles |
| HP:0003555 | 77 | Muscle fiber splitting |
| HP:0003736 | 77 | Autophagic vacuoles |
| HP:0009077 | 77 | Weakness of long finger extensor muscles |
| HP:0012899 | 77 | Handgrip myotonia |

| Cluster | Cell function | Description | Phenotype\_coverage | Genes |
| --- | --- | --- | --- | --- |
| 77 | GO:0007517 | muscle organ development | 71.42857 | NEB, TTN, ACTA1, DMD, CFL2, KLHL41, LMOD3, LARGE1, MYH7, FKTN, CRYAB |
| 77 | GO:0006941 | striated muscle contraction | 71.42857 | TTN, CHRND, DMD, CHRNA1, SCN4A, CHRNB1, KLHL41, LMOD3, MYH7 |

---


---


---

# Cluster #86

| HPO | Cluster | Description |
| --- | --- | --- |
| HP:0003236 | 86 | Elevated serum creatine kinase |
| HP:0001315 | 86 | Reduced tendon reflexes |
| HP:0003307 | 86 | Hyperlordosis |
| HP:0003551 | 86 | Difficulty climbing stairs |
| HP:0012664 | 86 | Reduced ejection fraction |
| HP:0005085 | 86 | Limited knee flexion/extension |
| HP:0100748 | 86 | Muscular edema |

| Cluster | Cell function | Description | Phenotype\_coverage | Genes |
| --- | --- | --- | --- | --- |
| 86 | GO:0003012 | muscle system process | 71.42857 | TTN, ACTA1, DMD, TPM2, DYSF, SCN4A, LMNA, TPM3, MYH7, MYOT |
| 86 | GO:0048747 | muscle fiber development | 71.42857 | SELENON, TTN, DMD, ACTA1, DYSF |

---


---


---

# Cluster #100

| HPO | Cluster | Description |
| --- | --- | --- |
| HP:0003712 | 100 | Skeletal muscle hypertrophy |
| HP:0000597 | 100 | Ophthalmoparesis |
| HP:0003457 | 100 | EMG abnormality |
| HP:0002047 | 100 | Malignant hyperthermia |
| HP:0002153 | 100 | Hyperkalemia |
| HP:0003752 | 100 | Episodic flaccid weakness |
| HP:0002486 | 100 | Myotonia |

| Cluster | Cell function | Description | Phenotype\_coverage | Genes |
| --- | --- | --- | --- | --- |
| 100 | GO:0014819 | regulation of skeletal muscle contraction | 71.42857 | SCN4A, DMPK |

---


---


---

# Cluster #140

| HPO | Cluster | Description |
| --- | --- | --- |
| HP:0001270 | 140 | Motor delay |
| HP:0001638 | 140 | Cardiomyopathy |
| HP:0100295 | 140 | Muscle fiber atrophy |
| HP:0009025 | 140 | Increased connective tissue |
| HP:0009058 | 140 | Increased muscle lipid content |
| HP:0002111 | 140 | Restrictive deficit on pulmonary function testing |
| HP:0003741 | 140 | Congenital muscular dystrophy |

| Cluster | Cell function | Description | Phenotype\_coverage | Genes |
| --- | --- | --- | --- | --- |
| 140 | hsa05410 | Hypertrophic cardiomyopathy (HCM) | 71.42857 | LAMA2, TTN, TPM3, TPM2, MYH7 |
| 140 | hsa05414 | Dilated cardiomyopathy | 71.42857 | LAMA2, TTN, TPM3, TPM2, MYH7 |
| 140 | GO:0007517 | muscle organ development | 71.42857 | NEB, ACTA1, SELENON, TTN, LAMA2, KLHL41, LMOD3, RYR1, MYH7, FKTN, KLHL40 |
| 140 | GO:0030049 | muscle filament sliding | 71.42857 | NEB, ACTA1, TTN, TPM2, MYH7, TPM3 |
| 140 | GO:0014706 | striated muscle tissue development | 71.42857 | NEB, ACTA1, SELENON, TTN, LMOD3, KLHL41, RYR1, MYH7, KLHL40 |
| 140 | GO:0030240 | skeletal muscle thin filament assembly | 71.42857 | LMOD3, ACTA1, TTN |
| 140 | GO:0048747 | muscle fiber development | 71.42857 | NEB, ACTA1, SELENON, TTN, LMOD3, KLHL41, RYR1, KLHL40 |

---


---


---

# Cluster #65

| HPO | Cluster | Description |
| --- | --- | --- |
| HP:0001270 | 65 | Motor delay |
| HP:0040083 | 65 | Toe walking |
| HP:0001436 | 65 | Abnormality of the foot musculature |
| HP:0009027 | 65 | Foot dorsiflexor weakness |
| HP:0002515 | 65 | Waddling gait |
| HP:0001265 | 65 | Hyporeflexia |
| HP:0008956 | 65 | Proximal lower limb amyotrophy |
| HP:0008994 | 65 | Proximal muscle weakness in lower limbs |
| HP:0001284 | 65 | Areflexia |
| HP:0008981 | 65 | Calf muscle hypertrophy |
| HP:0003236 | 65 | Elevated serum creatine kinase |
| HP:0003391 | 65 | Gowers sign |
| HP:0003307 | 65 | Hyperlordosis |
| HP:0001558 | 65 | Decreased fetal movement |
| HP:0003306 | 65 | Spinal rigidity |
| HP:0001349 | 65 | Facial diplegia |
| HP:0003691 | 65 | Scapular winging |
| HP:0003803 | 65 | Type 1 muscle fiber predominance |
| HP:0003458 | 65 | EMG: myopathic abnormalities |
| HP:0008997 | 65 | Proximal muscle weakness in upper limbs |
| HP:0003798 | 65 | Nemaline bodies |
| HP:0002792 | 65 | Reduced vital capacity |
| HP:0008954 | 65 | Intrinsic hand muscle atrophy |
| HP:0008963 | 65 | Tibialis muscle weakness |
| HP:0009005 | 65 | Weakness of the intrinsic hand muscles |
| HP:0009073 | 65 | Progressive proximal muscle weakness |
| HP:0040081 | 65 | Abnormal circulating creatine kinase concentration |
| HP:0003555 | 65 | Muscle fiber splitting |
| HP:0003805 | 65 | Rimmed vacuoles |
| HP:0002987 | 65 | Elbow flexion contracture |
| HP:0011807 | 65 | Type 1 muscle fiber atrophy |
| HP:0003730 | 65 | EMG: myotonic runs |
| HP:0006785 | 65 | Limb-girdle muscular dystrophy |
| HP:0012548 | 65 | Fatty replacement of skeletal muscle |
| HP:0100297 | 65 | Increased endomysial connective tissue |
| HP:0008948 | 65 | Proximal upper limb amyotrophy |
| HP:0001771 | 65 | Achilles tendon contracture |
| HP:0004631 | 65 | Decreased cervical spine flexion due to contractures of posterior cervical muscles |
| HP:0003547 | 65 | Shoulder girdle muscle weakness |
| HP:0003731 | 65 | Quadriceps muscle weakness |
| HP:0001678 | 65 | Atrioventricular block |
| HP:0002486 | 65 | Myotonia |
| HP:0009077 | 65 | Weakness of long finger extensor muscles |
| HP:0030007 | 65 | EMG: positive sharp waves |
| HP:0100284 | 65 | EMG: myotonic discharges |
| HP:0009063 | 65 | Progressive distal muscle weakness |
| HP:0006467 | 65 | Limited shoulder movement |
| HP:0031108 | 65 | Triceps weakness |

| Cluster | Cell function | Description | Phenotype\_coverage | Genes |
| --- | --- | --- | --- | --- |
| 65 | GO:0007517 | muscle organ development | 70.83333 | MYF6, KLHL41, LMOD3, NEB, CAPN3, SGCD, TTN, SELENON, CHRNA1, EMD, SGCB, FKTN, KLHL40, CRYAB, SPEG, FHL1, DMD, CFL2, MYH14, CAV3, ITGA7, RYR1, COL6A3, MYH7, SGCA, FOS, ACTA1, CHRND, MYL2, SGCG, LARGE1 |

---


---


---

# Cluster #132

| HPO | Cluster | Description |
| --- | --- | --- |
| HP:0008800 | 132 | Limited hip movement |
| HP:0001436 | 132 | Abnormality of the foot musculature |
| HP:0008963 | 132 | Tibialis muscle weakness |
| HP:0003555 | 132 | Muscle fiber splitting |
| HP:0003730 | 132 | EMG: myotonic runs |
| HP:0012548 | 132 | Fatty replacement of skeletal muscle |
| HP:0003547 | 132 | Shoulder girdle muscle weakness |
| HP:0030007 | 132 | EMG: positive sharp waves |
| HP:0100284 | 132 | EMG: myotonic discharges |
| HP:0006467 | 132 | Limited shoulder movement |

| Cluster | Cell function | Description | Phenotype\_coverage | Genes |
| --- | --- | --- | --- | --- |
| 132 | GO:0007517 | muscle organ development | 70 | TTN, CHRND, CHRNA1, MYF6, SGCG, MYH7, SGCA |

---


---


---

# Cluster #4

| HPO | Cluster | Description |
| --- | --- | --- |
| HP:0001270 | 4 | Motor delay |
| HP:0001290 | 4 | Generalized hypotonia |
| HP:0002515 | 4 | Waddling gait |
| HP:0001265 | 4 | Hyporeflexia |
| HP:0001284 | 4 | Areflexia |
| HP:0003391 | 4 | Gowers sign |
| HP:0003324 | 4 | Generalized muscle weakness |
| HP:0003701 | 4 | Proximal muscle weakness |
| HP:0002421 | 4 | Poor head control |
| HP:0001558 | 4 | Decreased fetal movement |
| HP:0003306 | 4 | Spinal rigidity |
| HP:0000467 | 4 | Neck muscle weakness |
| HP:0003403 | 4 | EMG: decremental response of compound muscle action potential to repetitive nerve stimulation |
| HP:0003803 | 4 | Type 1 muscle fiber predominance |
| HP:0003325 | 4 | Limb-girdle muscle weakness |
| HP:0003388 | 4 | Easy fatigability |

---


---


---

# Cluster #6

| HPO | Cluster | Description |
| --- | --- | --- |
| HP:0002355 | 6 | Difficulty walking |
| HP:0009027 | 6 | Foot dorsiflexor weakness |
| HP:0002515 | 6 | Waddling gait |
| HP:0001284 | 6 | Areflexia |
| HP:0008981 | 6 | Calf muscle hypertrophy |
| HP:0003236 | 6 | Elevated serum creatine kinase |
| HP:0001638 | 6 | Cardiomyopathy |
| HP:0003391 | 6 | Gowers sign |
| HP:0003557 | 6 | Increased variability in muscle fiber diameter |
| HP:0003701 | 6 | Proximal muscle weakness |
| HP:0000467 | 6 | Neck muscle weakness |
| HP:0003551 | 6 | Difficulty climbing stairs |
| HP:0003691 | 6 | Scapular winging |
| HP:0003458 | 6 | EMG: myopathic abnormalities |
| HP:0008997 | 6 | Proximal muscle weakness in upper limbs |
| HP:0003805 | 6 | Rimmed vacuoles |
| HP:0012548 | 6 | Fatty replacement of skeletal muscle |
| HP:0003547 | 6 | Shoulder girdle muscle weakness |

---


---


---

# Cluster #9

| HPO | Cluster | Description |
| --- | --- | --- |
| HP:0002355 | 9 | Difficulty walking |
| HP:0001265 | 9 | Hyporeflexia |
| HP:0002460 | 9 | Distal muscle weakness |
| HP:0000508 | 9 | Ptosis |
| HP:0003324 | 9 | Generalized muscle weakness |
| HP:0003701 | 9 | Proximal muscle weakness |
| HP:0002747 | 9 | Respiratory insufficiency due to muscle weakness |
| HP:0001283 | 9 | Bulbar palsy |

---


---


---

# Cluster #10

| HPO | Cluster | Description |
| --- | --- | --- |
| HP:0002359 | 10 | Frequent falls |
| HP:0002515 | 10 | Waddling gait |
| HP:0009046 | 10 | Difficulty running |
| HP:0001284 | 10 | Areflexia |
| HP:0002460 | 10 | Distal muscle weakness |
| HP:0003236 | 10 | Elevated serum creatine kinase |
| HP:0003391 | 10 | Gowers sign |
| HP:0003403 | 10 | EMG: decremental response of compound muscle action potential to repetitive nerve stimulation |
| HP:0003551 | 10 | Difficulty climbing stairs |
| HP:0003691 | 10 | Scapular winging |
| HP:0003803 | 10 | Type 1 muscle fiber predominance |
| HP:0003388 | 10 | Easy fatigability |

---


---


---

# Cluster #12

| HPO | Cluster | Description |
| --- | --- | --- |
| HP:0003198 | 12 | Myopathy |
| HP:0001270 | 12 | Motor delay |
| HP:0002359 | 12 | Frequent falls |
| HP:0002355 | 12 | Difficulty walking |
| HP:0009027 | 12 | Foot dorsiflexor weakness |
| HP:0002515 | 12 | Waddling gait |
| HP:0009046 | 12 | Difficulty running |
| HP:0008981 | 12 | Calf muscle hypertrophy |
| HP:0003236 | 12 | Elevated serum creatine kinase |
| HP:0003391 | 12 | Gowers sign |
| HP:0003749 | 12 | Pelvic girdle muscle weakness |
| HP:0003551 | 12 | Difficulty climbing stairs |
| HP:0003388 | 12 | Easy fatigability |
| HP:0003805 | 12 | Rimmed vacuoles |
| HP:0001771 | 12 | Achilles tendon contracture |
| HP:0003547 | 12 | Shoulder girdle muscle weakness |

---


---


---

# Cluster #18

| HPO | Cluster | Description |
| --- | --- | --- |
| HP:0001270 | 18 | Motor delay |
| HP:0002359 | 18 | Frequent falls |
| HP:0002515 | 18 | Waddling gait |
| HP:0008981 | 18 | Calf muscle hypertrophy |
| HP:0003236 | 18 | Elevated serum creatine kinase |
| HP:0001644 | 18 | Dilated cardiomyopathy |
| HP:0003701 | 18 | Proximal muscle weakness |
| HP:0002938 | 18 | Lumbar hyperlordosis |
| HP:0003551 | 18 | Difficulty climbing stairs |
| HP:0006785 | 18 | Limb-girdle muscular dystrophy |
| HP:0025169 | 18 | Left ventricular systolic dysfunction |
| HP:0030099 | 18 | Reduced muscle fiber alpha dystroglycan |
| HP:0003707 | 18 | Calf muscle pseudohypertrophy |

---


---


---

# Cluster #19

| HPO | Cluster | Description |
| --- | --- | --- |
| HP:0001270 | 19 | Motor delay |
| HP:0002355 | 19 | Difficulty walking |
| HP:0009027 | 19 | Foot dorsiflexor weakness |
| HP:0030237 | 19 | Hand muscle weakness |
| HP:0002515 | 19 | Waddling gait |
| HP:0001265 | 19 | Hyporeflexia |
| HP:0001284 | 19 | Areflexia |
| HP:0002460 | 19 | Distal muscle weakness |
| HP:0010628 | 19 | Facial palsy |
| HP:0003391 | 19 | Gowers sign |
| HP:0003701 | 19 | Proximal muscle weakness |
| HP:0000467 | 19 | Neck muscle weakness |
| HP:0003551 | 19 | Difficulty climbing stairs |
| HP:0003691 | 19 | Scapular winging |
| HP:0003803 | 19 | Type 1 muscle fiber predominance |
| HP:0003458 | 19 | EMG: myopathic abnormalities |
| HP:0008180 | 19 | Mildly elevated creatine kinase |
| HP:0002792 | 19 | Reduced vital capacity |
| HP:0003388 | 19 | Easy fatigability |
| HP:0003805 | 19 | Rimmed vacuoles |
| HP:0012548 | 19 | Fatty replacement of skeletal muscle |
| HP:0003547 | 19 | Shoulder girdle muscle weakness |

---


---


---

# Cluster #20

| HPO | Cluster | Description |
| --- | --- | --- |
| HP:0003198 | 20 | Myopathy |
| HP:0002355 | 20 | Difficulty walking |
| HP:0001436 | 20 | Abnormality of the foot musculature |
| HP:0009027 | 20 | Foot dorsiflexor weakness |
| HP:0030237 | 20 | Hand muscle weakness |
| HP:0002515 | 20 | Waddling gait |
| HP:0001265 | 20 | Hyporeflexia |
| HP:0008956 | 20 | Proximal lower limb amyotrophy |
| HP:0009046 | 20 | Difficulty running |
| HP:0010628 | 20 | Facial palsy |
| HP:0003391 | 20 | Gowers sign |
| HP:0003701 | 20 | Proximal muscle weakness |
| HP:0000467 | 20 | Neck muscle weakness |
| HP:0003691 | 20 | Scapular winging |
| HP:0003388 | 20 | Easy fatigability |
| HP:0003547 | 20 | Shoulder girdle muscle weakness |
| HP:0031108 | 20 | Triceps weakness |

---


---


---

# Cluster #26

| HPO | Cluster | Description |
| --- | --- | --- |
| HP:0002355 | 26 | Difficulty walking |
| HP:0003394 | 26 | Muscle spasm |
| HP:0001290 | 26 | Generalized hypotonia |
| HP:0003326 | 26 | Myalgia |
| HP:0001771 | 26 | Achilles tendon contracture |
| HP:0003797 | 26 | Limb-girdle muscle atrophy |

---


---


---

# Cluster #27

| HPO | Cluster | Description |
| --- | --- | --- |
| HP:0003198 | 27 | Myopathy |
| HP:0002355 | 27 | Difficulty walking |
| HP:0001265 | 27 | Hyporeflexia |
| HP:0003236 | 27 | Elevated serum creatine kinase |
| HP:0001638 | 27 | Cardiomyopathy |
| HP:0003546 | 27 | Exercise intolerance |
| HP:0001349 | 27 | Facial diplegia |
| HP:0002747 | 27 | Respiratory insufficiency due to muscle weakness |
| HP:0003458 | 27 | EMG: myopathic abnormalities |
| HP:0003798 | 27 | Nemaline bodies |
| HP:0003552 | 27 | Muscle stiffness |
| HP:0009073 | 27 | Progressive proximal muscle weakness |
| HP:0040081 | 27 | Abnormal circulating creatine kinase concentration |
| HP:0009063 | 27 | Progressive distal muscle weakness |

---


---


---

# Cluster #28

| HPO | Cluster | Description |
| --- | --- | --- |
| HP:0009027 | 28 | Foot dorsiflexor weakness |
| HP:0010628 | 28 | Facial palsy |
| HP:0001638 | 28 | Cardiomyopathy |
| HP:0009073 | 28 | Progressive proximal muscle weakness |
| HP:0012548 | 28 | Fatty replacement of skeletal muscle |
| HP:0003547 | 28 | Shoulder girdle muscle weakness |
| HP:0009077 | 28 | Weakness of long finger extensor muscles |
| HP:0030007 | 28 | EMG: positive sharp waves |
| HP:0100284 | 28 | EMG: myotonic discharges |
| HP:0006467 | 28 | Limited shoulder movement |

---


---


---

# Cluster #30

| HPO | Cluster | Description |
| --- | --- | --- |
| HP:0009053 | 30 | Distal lower limb muscle weakness |
| HP:0003693 | 30 | Distal amyotrophy |
| HP:0002355 | 30 | Difficulty walking |
| HP:0009027 | 30 | Foot dorsiflexor weakness |
| HP:0001265 | 30 | Hyporeflexia |
| HP:0001284 | 30 | Areflexia |

---


---


---

# Cluster #31

| HPO | Cluster | Description |
| --- | --- | --- |
| HP:0002359 | 31 | Frequent falls |
| HP:0001290 | 31 | Generalized hypotonia |
| HP:0003557 | 31 | Increased variability in muscle fiber diameter |
| HP:0008081 | 31 | Pes valgus |
| HP:0002421 | 31 | Poor head control |
| HP:0003700 | 31 | Generalized amyotrophy |
| HP:0003306 | 31 | Spinal rigidity |
| HP:0003458 | 31 | EMG: myopathic abnormalities |

---


---


---

# Cluster #32

| HPO | Cluster | Description |
| --- | --- | --- |
| HP:0009027 | 32 | Foot dorsiflexor weakness |
| HP:0002460 | 32 | Distal muscle weakness |
| HP:0003236 | 32 | Elevated serum creatine kinase |
| HP:0003557 | 32 | Increased variability in muscle fiber diameter |
| HP:0003327 | 32 | Axial muscle weakness |
| HP:0000467 | 32 | Neck muscle weakness |
| HP:0003551 | 32 | Difficulty climbing stairs |
| HP:0003458 | 32 | EMG: myopathic abnormalities |
| HP:0003555 | 32 | Muscle fiber splitting |
| HP:0100297 | 32 | Increased endomysial connective tissue |
| HP:0003736 | 32 | Autophagic vacuoles |
| HP:0012515 | 32 | Hip flexor weakness |

---


---


---

# Cluster #34

| HPO | Cluster | Description |
| --- | --- | --- |
| HP:0003198 | 34 | Myopathy |
| HP:0001265 | 34 | Hyporeflexia |
| HP:0001284 | 34 | Areflexia |
| HP:0003307 | 34 | Hyperlordosis |
| HP:0001618 | 34 | Dysphonia |
| HP:0003700 | 34 | Generalized amyotrophy |
| HP:0003306 | 34 | Spinal rigidity |
| HP:0000467 | 34 | Neck muscle weakness |
| HP:0001349 | 34 | Facial diplegia |
| HP:0003273 | 34 | Hip contracture |
| HP:0003325 | 34 | Limb-girdle muscle weakness |

---


---


---

# Cluster #35

| HPO | Cluster | Description |
| --- | --- | --- |
| HP:0002355 | 35 | Difficulty walking |
| HP:0001284 | 35 | Areflexia |
| HP:0001638 | 35 | Cardiomyopathy |
| HP:0003391 | 35 | Gowers sign |
| HP:0003458 | 35 | EMG: myopathic abnormalities |
| HP:0009073 | 35 | Progressive proximal muscle weakness |
| HP:0040081 | 35 | Abnormal circulating creatine kinase concentration |
| HP:0030319 | 35 | Weakness of facial musculature |
| HP:0003547 | 35 | Shoulder girdle muscle weakness |
| HP:0012240 | 35 | Increased intramyocellular lipid droplets |

---


---


---

# Cluster #36

| HPO | Cluster | Description |
| --- | --- | --- |
| HP:0000298 | 36 | Mask-like facies |
| HP:0004326 | 36 | Cachexia |
| HP:0000600 | 36 | Abnormality of the pharynx |
| HP:0001621 | 36 | Weak voice |

---


---


---

# Cluster #37

| HPO | Cluster | Description |
| --- | --- | --- |
| HP:0002015 | 37 | Dysphagia |
| HP:0001290 | 37 | Generalized hypotonia |
| HP:0000508 | 37 | Ptosis |
| HP:0003200 | 37 | Ragged-red muscle fibers |
| HP:0003546 | 37 | Exercise intolerance |
| HP:0003701 | 37 | Proximal muscle weakness |
| HP:0001558 | 37 | Decreased fetal movement |
| HP:0001349 | 37 | Facial diplegia |
| HP:0001712 | 37 | Left ventricular hypertrophy |
| HP:0003803 | 37 | Type 1 muscle fiber predominance |
| HP:0000544 | 37 | External ophthalmoplegia |
| HP:0003388 | 37 | Easy fatigability |
| HP:0003737 | 37 | Mitochondrial myopathy |
| HP:0003547 | 37 | Shoulder girdle muscle weakness |

---


---


---

# Cluster #39

| HPO | Cluster | Description |
| --- | --- | --- |
| HP:0001436 | 39 | Abnormality of the foot musculature |
| HP:0002515 | 39 | Waddling gait |
| HP:0008956 | 39 | Proximal lower limb amyotrophy |
| HP:0009046 | 39 | Difficulty running |
| HP:0003236 | 39 | Elevated serum creatine kinase |
| HP:0000508 | 39 | Ptosis |
| HP:0010628 | 39 | Facial palsy |
| HP:0001315 | 39 | Reduced tendon reflexes |
| HP:0001638 | 39 | Cardiomyopathy |
| HP:0003391 | 39 | Gowers sign |
| HP:0003557 | 39 | Increased variability in muscle fiber diameter |
| HP:0003701 | 39 | Proximal muscle weakness |
| HP:0003327 | 39 | Axial muscle weakness |
| HP:0003403 | 39 | EMG: decremental response of compound muscle action potential to repetitive nerve stimulation |
| HP:0003691 | 39 | Scapular winging |
| HP:0003803 | 39 | Type 1 muscle fiber predominance |
| HP:0003458 | 39 | EMG: myopathic abnormalities |
| HP:0003388 | 39 | Easy fatigability |
| HP:0008963 | 39 | Tibialis muscle weakness |
| HP:0003555 | 39 | Muscle fiber splitting |
| HP:0001612 | 39 | Weak cry |
| HP:0100297 | 39 | Increased endomysial connective tissue |
| HP:0003547 | 39 | Shoulder girdle muscle weakness |
| HP:0012515 | 39 | Hip flexor weakness |
| HP:0006467 | 39 | Limited shoulder movement |

---


---


---

# Cluster #40

| HPO | Cluster | Description |
| --- | --- | --- |
| HP:0002359 | 40 | Frequent falls |
| HP:0040083 | 40 | Toe walking |
| HP:0002355 | 40 | Difficulty walking |
| HP:0008981 | 40 | Calf muscle hypertrophy |
| HP:0003749 | 40 | Pelvic girdle muscle weakness |
| HP:0002540 | 40 | Inability to walk |
| HP:0003551 | 40 | Difficulty climbing stairs |
| HP:0003458 | 40 | EMG: myopathic abnormalities |
| HP:0008180 | 40 | Mildly elevated creatine kinase |
| HP:0009073 | 40 | Progressive proximal muscle weakness |
| HP:0007126 | 40 | Proximal amyotrophy |
| HP:0025169 | 40 | Left ventricular systolic dysfunction |
| HP:0030099 | 40 | Reduced muscle fiber alpha dystroglycan |
| HP:0001771 | 40 | Achilles tendon contracture |
| HP:0003547 | 40 | Shoulder girdle muscle weakness |

---


---


---

# Cluster #42

| HPO | Cluster | Description |
| --- | --- | --- |
| HP:0040083 | 42 | Toe walking |
| HP:0002355 | 42 | Difficulty walking |
| HP:0009027 | 42 | Foot dorsiflexor weakness |
| HP:0001290 | 42 | Generalized hypotonia |
| HP:0002460 | 42 | Distal muscle weakness |
| HP:0002878 | 42 | Respiratory failure |
| HP:0003551 | 42 | Difficulty climbing stairs |
| HP:0003698 | 42 | Difficulty standing |
| HP:0003547 | 42 | Shoulder girdle muscle weakness |

---


---


---

# Cluster #44

| HPO | Cluster | Description |
| --- | --- | --- |
| HP:0003551 | 44 | Difficulty climbing stairs |
| HP:0003555 | 44 | Muscle fiber splitting |
| HP:0003805 | 44 | Rimmed vacuoles |
| HP:0003736 | 44 | Autophagic vacuoles |
| HP:0012899 | 44 | Handgrip myotonia |
| HP:0003715 | 44 | Myofibrillar myopathy |

---


---


---

# Cluster #46

| HPO | Cluster | Description |
| --- | --- | --- |
| HP:0001290 | 46 | Generalized hypotonia |
| HP:0001644 | 46 | Dilated cardiomyopathy |
| HP:0003326 | 46 | Myalgia |
| HP:0003324 | 46 | Generalized muscle weakness |
| HP:0008331 | 46 | Elevated creatine kinase after exercise |
| HP:0003458 | 46 | EMG: myopathic abnormalities |

---


---


---

# Cluster #49

| HPO | Cluster | Description |
| --- | --- | --- |
| HP:0002015 | 49 | Dysphagia |
| HP:0002359 | 49 | Frequent falls |
| HP:0008981 | 49 | Calf muscle hypertrophy |
| HP:0003749 | 49 | Pelvic girdle muscle weakness |
| HP:0003324 | 49 | Generalized muscle weakness |
| HP:0003551 | 49 | Difficulty climbing stairs |
| HP:0002522 | 49 | Areflexia of lower limbs |
| HP:0002747 | 49 | Respiratory insufficiency due to muscle weakness |
| HP:0003722 | 49 | Neck flexor weakness |
| HP:0030196 | 49 | Fatigable weakness of respiratory muscles |
| HP:0006957 | 49 | Loss of ability to walk |
| HP:0030319 | 49 | Weakness of facial musculature |

---


---


---

# Cluster #50

| HPO | Cluster | Description |
| --- | --- | --- |
| HP:0001270 | 50 | Motor delay |
| HP:0002355 | 50 | Difficulty walking |
| HP:0002515 | 50 | Waddling gait |
| HP:0008994 | 50 | Proximal muscle weakness in lower limbs |
| HP:0009046 | 50 | Difficulty running |
| HP:0008981 | 50 | Calf muscle hypertrophy |
| HP:0003236 | 50 | Elevated serum creatine kinase |
| HP:0001644 | 50 | Dilated cardiomyopathy |
| HP:0001315 | 50 | Reduced tendon reflexes |
| HP:0003391 | 50 | Gowers sign |
| HP:0003557 | 50 | Increased variability in muscle fiber diameter |
| HP:0003306 | 50 | Spinal rigidity |
| HP:0003551 | 50 | Difficulty climbing stairs |
| HP:0003691 | 50 | Scapular winging |
| HP:0003803 | 50 | Type 1 muscle fiber predominance |
| HP:0002747 | 50 | Respiratory insufficiency due to muscle weakness |
| HP:0008997 | 50 | Proximal muscle weakness in upper limbs |
| HP:0008963 | 50 | Tibialis muscle weakness |
| HP:0003555 | 50 | Muscle fiber splitting |
| HP:0002987 | 50 | Elbow flexion contracture |
| HP:0006466 | 50 | Ankle flexion contracture |
| HP:0012785 | 50 | Flexion contracture of finger |
| HP:0001239 | 50 | Wrist flexion contracture |
| HP:0003089 | 50 | Hamstring contractures |
| HP:0003730 | 50 | EMG: myotonic runs |
| HP:0006785 | 50 | Limb-girdle muscular dystrophy |
| HP:0100297 | 50 | Increased endomysial connective tissue |
| HP:0025169 | 50 | Left ventricular systolic dysfunction |
| HP:0001771 | 50 | Achilles tendon contracture |

---


---


---

# Cluster #52

| HPO | Cluster | Description |
| --- | --- | --- |
| HP:0001270 | 52 | Motor delay |
| HP:0002355 | 52 | Difficulty walking |
| HP:0002515 | 52 | Waddling gait |
| HP:0001265 | 52 | Hyporeflexia |
| HP:0008981 | 52 | Calf muscle hypertrophy |
| HP:0003236 | 52 | Elevated serum creatine kinase |
| HP:0001638 | 52 | Cardiomyopathy |
| HP:0003391 | 52 | Gowers sign |
| HP:0003557 | 52 | Increased variability in muscle fiber diameter |
| HP:0003324 | 52 | Generalized muscle weakness |
| HP:0001558 | 52 | Decreased fetal movement |
| HP:0003306 | 52 | Spinal rigidity |
| HP:0000467 | 52 | Neck muscle weakness |
| HP:0001349 | 52 | Facial diplegia |
| HP:0003551 | 52 | Difficulty climbing stairs |
| HP:0003691 | 52 | Scapular winging |
| HP:0003803 | 52 | Type 1 muscle fiber predominance |
| HP:0008997 | 52 | Proximal muscle weakness in upper limbs |
| HP:0003325 | 52 | Limb-girdle muscle weakness |
| HP:0030198 | 52 | Fatigable weakness of distal limb muscles |
| HP:0003388 | 52 | Easy fatigability |
| HP:0008954 | 52 | Intrinsic hand muscle atrophy |
| HP:0009005 | 52 | Weakness of the intrinsic hand muscles |
| HP:0009073 | 52 | Progressive proximal muscle weakness |
| HP:0040081 | 52 | Abnormal circulating creatine kinase concentration |

---


---


---

# Cluster #53

| HPO | Cluster | Description |
| --- | --- | --- |
| HP:0001270 | 53 | Motor delay |
| HP:0002359 | 53 | Frequent falls |
| HP:0040083 | 53 | Toe walking |
| HP:0002355 | 53 | Difficulty walking |
| HP:0001290 | 53 | Generalized hypotonia |
| HP:0002515 | 53 | Waddling gait |
| HP:0001265 | 53 | Hyporeflexia |
| HP:0001284 | 53 | Areflexia |
| HP:0008981 | 53 | Calf muscle hypertrophy |
| HP:0000602 | 53 | Ophthalmoplegia |
| HP:0002878 | 53 | Respiratory failure |
| HP:0003557 | 53 | Increased variability in muscle fiber diameter |
| HP:0003324 | 53 | Generalized muscle weakness |
| HP:0003701 | 53 | Proximal muscle weakness |
| HP:0001558 | 53 | Decreased fetal movement |
| HP:0003306 | 53 | Spinal rigidity |
| HP:0003551 | 53 | Difficulty climbing stairs |
| HP:0003691 | 53 | Scapular winging |
| HP:0003803 | 53 | Type 1 muscle fiber predominance |
| HP:0002747 | 53 | Respiratory insufficiency due to muscle weakness |
| HP:0003458 | 53 | EMG: myopathic abnormalities |
| HP:0003388 | 53 | Easy fatigability |
| HP:0008954 | 53 | Intrinsic hand muscle atrophy |
| HP:0009073 | 53 | Progressive proximal muscle weakness |
| HP:0002987 | 53 | Elbow flexion contracture |
| HP:0012548 | 53 | Fatty replacement of skeletal muscle |
| HP:0001771 | 53 | Achilles tendon contracture |

---


---


---

# Cluster #54

| HPO | Cluster | Description |
| --- | --- | --- |
| HP:0009053 | 54 | Distal lower limb muscle weakness |
| HP:0002355 | 54 | Difficulty walking |
| HP:0009027 | 54 | Foot dorsiflexor weakness |
| HP:0001265 | 54 | Hyporeflexia |
| HP:0008994 | 54 | Proximal muscle weakness in lower limbs |
| HP:0009046 | 54 | Difficulty running |
| HP:0001638 | 54 | Cardiomyopathy |
| HP:0003700 | 54 | Generalized amyotrophy |
| HP:0003306 | 54 | Spinal rigidity |
| HP:0002540 | 54 | Inability to walk |
| HP:0003551 | 54 | Difficulty climbing stairs |
| HP:0002747 | 54 | Respiratory insufficiency due to muscle weakness |
| HP:0008180 | 54 | Mildly elevated creatine kinase |
| HP:0008959 | 54 | Distal upper limb muscle weakness |
| HP:0009073 | 54 | Progressive proximal muscle weakness |
| HP:0030319 | 54 | Weakness of facial musculature |

---


---


---

# Cluster #55

| HPO | Cluster | Description |
| --- | --- | --- |
| HP:0040083 | 55 | Toe walking |
| HP:0002355 | 55 | Difficulty walking |
| HP:0003560 | 55 | Muscular dystrophy |
| HP:0003691 | 55 | Scapular winging |
| HP:0003458 | 55 | EMG: myopathic abnormalities |
| HP:0009005 | 55 | Weakness of the intrinsic hand muscles |
| HP:0009073 | 55 | Progressive proximal muscle weakness |
| HP:0002987 | 55 | Elbow flexion contracture |
| HP:0006466 | 55 | Ankle flexion contracture |
| HP:0012785 | 55 | Flexion contracture of finger |
| HP:0006785 | 55 | Limb-girdle muscular dystrophy |
| HP:0012548 | 55 | Fatty replacement of skeletal muscle |
| HP:0009063 | 55 | Progressive distal muscle weakness |

---


---


---

# Cluster #58

| HPO | Cluster | Description |
| --- | --- | --- |
| HP:0001270 | 58 | Motor delay |
| HP:0002355 | 58 | Difficulty walking |
| HP:0009027 | 58 | Foot dorsiflexor weakness |
| HP:0001265 | 58 | Hyporeflexia |
| HP:0001284 | 58 | Areflexia |
| HP:0009055 | 58 | Generalized limb muscle atrophy |
| HP:0000467 | 58 | Neck muscle weakness |
| HP:0001349 | 58 | Facial diplegia |
| HP:0003691 | 58 | Scapular winging |
| HP:0002380 | 58 | Fasciculations |

---


---


---

# Cluster #59

| HPO | Cluster | Description |
| --- | --- | --- |
| HP:0002015 | 59 | Dysphagia |
| HP:0001265 | 59 | Hyporeflexia |
| HP:0002804 | 59 | Arthrogryposis multiplex congenita |
| HP:0003749 | 59 | Pelvic girdle muscle weakness |
| HP:0000590 | 59 | Progressive external ophthalmoplegia |
| HP:0003546 | 59 | Exercise intolerance |
| HP:0003324 | 59 | Generalized muscle weakness |
| HP:0001488 | 59 | Bilateral ptosis |
| HP:0002540 | 59 | Inability to walk |
| HP:0006957 | 59 | Loss of ability to walk |
| HP:0001771 | 59 | Achilles tendon contracture |
| HP:0030319 | 59 | Weakness of facial musculature |

---


---


---

# Cluster #62

| HPO | Cluster | Description |
| --- | --- | --- |
| HP:0003198 | 62 | Myopathy |
| HP:0002359 | 62 | Frequent falls |
| HP:0040083 | 62 | Toe walking |
| HP:0002355 | 62 | Difficulty walking |
| HP:0003394 | 62 | Muscle spasm |
| HP:0001290 | 62 | Generalized hypotonia |
| HP:0001265 | 62 | Hyporeflexia |
| HP:0009046 | 62 | Difficulty running |
| HP:0003236 | 62 | Elevated serum creatine kinase |
| HP:0001276 | 62 | Hypertonia |
| HP:0003326 | 62 | Myalgia |
| HP:0000590 | 62 | Progressive external ophthalmoplegia |
| HP:0003457 | 62 | EMG abnormality |
| HP:0003546 | 62 | Exercise intolerance |
| HP:0100749 | 62 | Chest pain |
| HP:0001349 | 62 | Facial diplegia |
| HP:0003403 | 62 | EMG: decremental response of compound muscle action potential to repetitive nerve stimulation |
| HP:0003551 | 62 | Difficulty climbing stairs |
| HP:0003458 | 62 | EMG: myopathic abnormalities |
| HP:0003738 | 62 | Exercise-induced myalgia |
| HP:0003388 | 62 | Easy fatigability |
| HP:0003731 | 62 | Quadriceps muscle weakness |
| HP:0002486 | 62 | Myotonia |
| HP:0031108 | 62 | Triceps weakness |

---


---


---

# Cluster #64

| HPO | Cluster | Description |
| --- | --- | --- |
| HP:0003202 | 64 | Skeletal muscle atrophy |
| HP:0001265 | 64 | Hyporeflexia |
| HP:0001284 | 64 | Areflexia |
| HP:0010508 | 64 | Metatarsus valgus |
| HP:0001618 | 64 | Dysphonia |
| HP:0040081 | 64 | Abnormal circulating creatine kinase concentration |

---


---


---

# Cluster #66

| HPO | Cluster | Description |
| --- | --- | --- |
| HP:0003198 | 66 | Myopathy |
| HP:0001288 | 66 | Gait disturbance |
| HP:0002355 | 66 | Difficulty walking |
| HP:0002515 | 66 | Waddling gait |
| HP:0001265 | 66 | Hyporeflexia |
| HP:0001284 | 66 | Areflexia |
| HP:0003236 | 66 | Elevated serum creatine kinase |
| HP:0003712 | 66 | Skeletal muscle hypertrophy |
| HP:0000597 | 66 | Ophthalmoparesis |
| HP:0000298 | 66 | Mask-like facies |
| HP:0003326 | 66 | Myalgia |
| HP:0001638 | 66 | Cardiomyopathy |
| HP:0003391 | 66 | Gowers sign |
| HP:0003557 | 66 | Increased variability in muscle fiber diameter |
| HP:0003307 | 66 | Hyperlordosis |
| HP:0000590 | 66 | Progressive external ophthalmoplegia |
| HP:0003200 | 66 | Ragged-red muscle fibers |
| HP:0003457 | 66 | EMG abnormality |
| HP:0003546 | 66 | Exercise intolerance |
| HP:0003701 | 66 | Proximal muscle weakness |
| HP:0002421 | 66 | Poor head control |
| HP:0003306 | 66 | Spinal rigidity |
| HP:0000467 | 66 | Neck muscle weakness |
| HP:0003403 | 66 | EMG: decremental response of compound muscle action potential to repetitive nerve stimulation |
| HP:0003691 | 66 | Scapular winging |
| HP:0003803 | 66 | Type 1 muscle fiber predominance |
| HP:0003325 | 66 | Limb-girdle muscle weakness |
| HP:0003388 | 66 | Easy fatigability |
| HP:0009073 | 66 | Progressive proximal muscle weakness |
| HP:0040081 | 66 | Abnormal circulating creatine kinase concentration |
| HP:0030319 | 66 | Weakness of facial musculature |
| HP:0003737 | 66 | Mitochondrial myopathy |
| HP:0002486 | 66 | Myotonia |

---


---


---

# Cluster #68

| HPO | Cluster | Description |
| --- | --- | --- |
| HP:0003394 | 68 | Muscle spasm |
| HP:0008981 | 68 | Calf muscle hypertrophy |
| HP:0003236 | 68 | Elevated serum creatine kinase |
| HP:0001638 | 68 | Cardiomyopathy |
| HP:0002913 | 68 | Myoglobinuria |
| HP:0003557 | 68 | Increased variability in muscle fiber diameter |
| HP:0003738 | 68 | Exercise-induced myalgia |
| HP:0003089 | 68 | Hamstring contractures |
| HP:0003730 | 68 | EMG: myotonic runs |

---


---


---

# Cluster #69

| HPO | Cluster | Description |
| --- | --- | --- |
| HP:0003326 | 69 | Myalgia |
| HP:0003749 | 69 | Pelvic girdle muscle weakness |
| HP:0007126 | 69 | Proximal amyotrophy |
| HP:0003756 | 69 | Skeletal myopathy |
| HP:0012507 | 69 | Weakness of orbicularis oculi muscle |
| HP:0030051 | 69 | Tip-toe gait |

---


---


---

# Cluster #70

| HPO | Cluster | Description |
| --- | --- | --- |
| HP:0002015 | 70 | Dysphagia |
| HP:0003198 | 70 | Myopathy |
| HP:0001288 | 70 | Gait disturbance |
| HP:0003202 | 70 | Skeletal muscle atrophy |
| HP:0001265 | 70 | Hyporeflexia |
| HP:0001324 | 70 | Muscle weakness |
| HP:0001284 | 70 | Areflexia |
| HP:0002460 | 70 | Distal muscle weakness |
| HP:0008981 | 70 | Calf muscle hypertrophy |
| HP:0003236 | 70 | Elevated serum creatine kinase |
| HP:0000508 | 70 | Ptosis |
| HP:0000597 | 70 | Ophthalmoparesis |
| HP:0003560 | 70 | Muscular dystrophy |
| HP:0000298 | 70 | Mask-like facies |
| HP:0010628 | 70 | Facial palsy |
| HP:0004326 | 70 | Cachexia |
| HP:0001315 | 70 | Reduced tendon reflexes |
| HP:0002804 | 70 | Arthrogryposis multiplex congenita |
| HP:0002878 | 70 | Respiratory failure |
| HP:0003457 | 70 | EMG abnormality |
| HP:0003324 | 70 | Generalized muscle weakness |
| HP:0003701 | 70 | Proximal muscle weakness |
| HP:0002421 | 70 | Poor head control |
| HP:0003803 | 70 | Type 1 muscle fiber predominance |
| HP:0005855 | 70 | Multiple prenatal fractures |
| HP:0003722 | 70 | Neck flexor weakness |
| HP:0030196 | 70 | Fatigable weakness of respiratory muscles |
| HP:0002792 | 70 | Reduced vital capacity |
| HP:0002486 | 70 | Myotonia |

---


---


---

# Cluster #72

| HPO | Cluster | Description |
| --- | --- | --- |
| HP:0003198 | 72 | Myopathy |
| HP:0002355 | 72 | Difficulty walking |
| HP:0008994 | 72 | Proximal muscle weakness in lower limbs |
| HP:0008981 | 72 | Calf muscle hypertrophy |
| HP:0001638 | 72 | Cardiomyopathy |
| HP:0003551 | 72 | Difficulty climbing stairs |
| HP:0002522 | 72 | Areflexia of lower limbs |
| HP:0002747 | 72 | Respiratory insufficiency due to muscle weakness |
| HP:0003738 | 72 | Exercise-induced myalgia |
| HP:0008180 | 72 | Mildly elevated creatine kinase |
| HP:0008997 | 72 | Proximal muscle weakness in upper limbs |
| HP:0003690 | 72 | Limb muscle weakness |
| HP:0008954 | 72 | Intrinsic hand muscle atrophy |
| HP:0008963 | 72 | Tibialis muscle weakness |
| HP:0009073 | 72 | Progressive proximal muscle weakness |
| HP:0040081 | 72 | Abnormal circulating creatine kinase concentration |
| HP:0006957 | 72 | Loss of ability to walk |
| HP:0030319 | 72 | Weakness of facial musculature |
| HP:0003547 | 72 | Shoulder girdle muscle weakness |

---


---


---

# Cluster #73

| HPO | Cluster | Description |
| --- | --- | --- |
| HP:0002359 | 73 | Frequent falls |
| HP:0009046 | 73 | Difficulty running |
| HP:0002058 | 73 | Myopathic facies |
| HP:0011712 | 73 | Right bundle branch block |
| HP:0002540 | 73 | Inability to walk |
| HP:0003698 | 73 | Difficulty standing |
| HP:0003551 | 73 | Difficulty climbing stairs |
| HP:0001771 | 73 | Achilles tendon contracture |
| HP:0012664 | 73 | Reduced ejection fraction |
| HP:0030051 | 73 | Tip-toe gait |
| HP:0005085 | 73 | Limited knee flexion/extension |

---


---


---

# Cluster #75

| HPO | Cluster | Description |
| --- | --- | --- |
| HP:0003198 | 75 | Myopathy |
| HP:0009053 | 75 | Distal lower limb muscle weakness |
| HP:0002515 | 75 | Waddling gait |
| HP:0003236 | 75 | Elevated serum creatine kinase |
| HP:0002375 | 75 | Hypokinesia |
| HP:0001561 | 75 | Polyhydramnios |
| HP:0002878 | 75 | Respiratory failure |
| HP:0003557 | 75 | Increased variability in muscle fiber diameter |
| HP:0002421 | 75 | Poor head control |
| HP:0001558 | 75 | Decreased fetal movement |
| HP:0003306 | 75 | Spinal rigidity |
| HP:0003327 | 75 | Axial muscle weakness |
| HP:0000467 | 75 | Neck muscle weakness |
| HP:0001349 | 75 | Facial diplegia |
| HP:0003403 | 75 | EMG: decremental response of compound muscle action potential to repetitive nerve stimulation |
| HP:0003803 | 75 | Type 1 muscle fiber predominance |
| HP:0002747 | 75 | Respiratory insufficiency due to muscle weakness |
| HP:0003798 | 75 | Nemaline bodies |
| HP:0009025 | 75 | Increased connective tissue |
| HP:0003325 | 75 | Limb-girdle muscle weakness |
| HP:0003388 | 75 | Easy fatigability |
| HP:0008959 | 75 | Distal upper limb muscle weakness |
| HP:0009073 | 75 | Progressive proximal muscle weakness |
| HP:0006957 | 75 | Loss of ability to walk |
| HP:0030319 | 75 | Weakness of facial musculature |

---


---


---

# Cluster #76

| HPO | Cluster | Description |
| --- | --- | --- |
| HP:0040083 | 76 | Toe walking |
| HP:0007210 | 76 | Lower limb amyotrophy |
| HP:0009046 | 76 | Difficulty running |
| HP:0010628 | 76 | Facial palsy |
| HP:0003557 | 76 | Increased variability in muscle fiber diameter |
| HP:0002421 | 76 | Poor head control |
| HP:0003691 | 76 | Scapular winging |
| HP:0008997 | 76 | Proximal muscle weakness in upper limbs |
| HP:0003730 | 76 | EMG: myotonic runs |
| HP:0012548 | 76 | Fatty replacement of skeletal muscle |
| HP:0030007 | 76 | EMG: positive sharp waves |
| HP:0100284 | 76 | EMG: myotonic discharges |

---


---


---

# Cluster #79

| HPO | Cluster | Description |
| --- | --- | --- |
| HP:0001270 | 79 | Motor delay |
| HP:0002359 | 79 | Frequent falls |
| HP:0003202 | 79 | Skeletal muscle atrophy |
| HP:0009027 | 79 | Foot dorsiflexor weakness |
| HP:0001290 | 79 | Generalized hypotonia |
| HP:0001265 | 79 | Hyporeflexia |
| HP:0001284 | 79 | Areflexia |
| HP:0002460 | 79 | Distal muscle weakness |
| HP:0000597 | 79 | Ophthalmoparesis |
| HP:0003457 | 79 | EMG abnormality |
| HP:0003700 | 79 | Generalized amyotrophy |
| HP:0001349 | 79 | Facial diplegia |
| HP:0003805 | 79 | Rimmed vacuoles |
| HP:0012548 | 79 | Fatty replacement of skeletal muscle |
| HP:0002380 | 79 | Fasciculations |

---


---


---

# Cluster #80

| HPO | Cluster | Description |
| --- | --- | --- |
| HP:0009053 | 80 | Distal lower limb muscle weakness |
| HP:0008800 | 80 | Limited hip movement |
| HP:0001436 | 80 | Abnormality of the foot musculature |
| HP:0009027 | 80 | Foot dorsiflexor weakness |
| HP:0003236 | 80 | Elevated serum creatine kinase |
| HP:0003691 | 80 | Scapular winging |
| HP:0002792 | 80 | Reduced vital capacity |
| HP:0012548 | 80 | Fatty replacement of skeletal muscle |
| HP:0009046 | 80 | Difficulty running |
| HP:0000467 | 80 | Neck muscle weakness |
| HP:0002540 | 80 | Inability to walk |
| HP:0001349 | 80 | Facial diplegia |
| HP:0003687 | 80 | Centrally nucleated skeletal muscle fibers |
| HP:0003803 | 80 | Type 1 muscle fiber predominance |
| HP:0003798 | 80 | Nemaline bodies |
| HP:0003325 | 80 | Limb-girdle muscle weakness |
| HP:0008959 | 80 | Distal upper limb muscle weakness |
| HP:0008963 | 80 | Tibialis muscle weakness |
| HP:0003555 | 80 | Muscle fiber splitting |
| HP:0100297 | 80 | Increased endomysial connective tissue |
| HP:0003547 | 80 | Shoulder girdle muscle weakness |
| HP:0006467 | 80 | Limited shoulder movement |

---


---


---

# Cluster #81

| HPO | Cluster | Description |
| --- | --- | --- |
| HP:0008800 | 81 | Limited hip movement |
| HP:0009027 | 81 | Foot dorsiflexor weakness |
| HP:0008994 | 81 | Proximal muscle weakness in lower limbs |
| HP:0009046 | 81 | Difficulty running |
| HP:0008981 | 81 | Calf muscle hypertrophy |
| HP:0003236 | 81 | Elevated serum creatine kinase |
| HP:0001638 | 81 | Cardiomyopathy |
| HP:0003557 | 81 | Increased variability in muscle fiber diameter |
| HP:0011712 | 81 | Right bundle branch block |
| HP:0003306 | 81 | Spinal rigidity |
| HP:0003327 | 81 | Axial muscle weakness |
| HP:0003551 | 81 | Difficulty climbing stairs |
| HP:0003691 | 81 | Scapular winging |
| HP:0003803 | 81 | Type 1 muscle fiber predominance |
| HP:0002747 | 81 | Respiratory insufficiency due to muscle weakness |
| HP:0003458 | 81 | EMG: myopathic abnormalities |
| HP:0008997 | 81 | Proximal muscle weakness in upper limbs |
| HP:0003798 | 81 | Nemaline bodies |
| HP:0003722 | 81 | Neck flexor weakness |
| HP:0002792 | 81 | Reduced vital capacity |
| HP:0009005 | 81 | Weakness of the intrinsic hand muscles |
| HP:0003730 | 81 | EMG: myotonic runs |
| HP:0025169 | 81 | Left ventricular systolic dysfunction |
| HP:0003547 | 81 | Shoulder girdle muscle weakness |
| HP:0030051 | 81 | Tip-toe gait |
| HP:0005085 | 81 | Limited knee flexion/extension |
| HP:0100748 | 81 | Muscular edema |
| HP:0009077 | 81 | Weakness of long finger extensor muscles |
| HP:0030007 | 81 | EMG: positive sharp waves |
| HP:0100284 | 81 | EMG: myotonic discharges |

---


---


---

# Cluster #83

| HPO | Cluster | Description |
| --- | --- | --- |
| HP:0003198 | 83 | Myopathy |
| HP:0001288 | 83 | Gait disturbance |
| HP:0003236 | 83 | Elevated serum creatine kinase |
| HP:0003712 | 83 | Skeletal muscle hypertrophy |
| HP:0001276 | 83 | Hypertonia |
| HP:0001561 | 83 | Polyhydramnios |
| HP:0002058 | 83 | Myopathic facies |
| HP:0003457 | 83 | EMG abnormality |
| HP:0002047 | 83 | Malignant hyperthermia |
| HP:0003803 | 83 | Type 1 muscle fiber predominance |
| HP:0002747 | 83 | Respiratory insufficiency due to muscle weakness |
| HP:0003388 | 83 | Easy fatigability |
| HP:0002486 | 83 | Myotonia |

---


---


---

# Cluster #84

| HPO | Cluster | Description |
| --- | --- | --- |
| HP:0001270 | 84 | Motor delay |
| HP:0002359 | 84 | Frequent falls |
| HP:0002515 | 84 | Waddling gait |
| HP:0009046 | 84 | Difficulty running |
| HP:0008981 | 84 | Calf muscle hypertrophy |
| HP:0003236 | 84 | Elevated serum creatine kinase |
| HP:0003391 | 84 | Gowers sign |
| HP:0002938 | 84 | Lumbar hyperlordosis |
| HP:0003700 | 84 | Generalized amyotrophy |
| HP:0003551 | 84 | Difficulty climbing stairs |
| HP:0003691 | 84 | Scapular winging |
| HP:0008997 | 84 | Proximal muscle weakness in upper limbs |
| HP:0001771 | 84 | Achilles tendon contracture |
| HP:0003707 | 84 | Calf muscle pseudohypertrophy |
| HP:0003803 | 84 | Type 1 muscle fiber predominance |
| HP:0003325 | 84 | Limb-girdle muscle weakness |
| HP:0003388 | 84 | Easy fatigability |

---


---


---

# Cluster #85

| HPO | Cluster | Description |
| --- | --- | --- |
| HP:0002359 | 85 | Frequent falls |
| HP:0002355 | 85 | Difficulty walking |
| HP:0009027 | 85 | Foot dorsiflexor weakness |
| HP:0009046 | 85 | Difficulty running |
| HP:0008981 | 85 | Calf muscle hypertrophy |
| HP:0010628 | 85 | Facial palsy |
| HP:0003557 | 85 | Increased variability in muscle fiber diameter |
| HP:0003551 | 85 | Difficulty climbing stairs |
| HP:0003803 | 85 | Type 1 muscle fiber predominance |
| HP:0003388 | 85 | Easy fatigability |
| HP:0008963 | 85 | Tibialis muscle weakness |
| HP:0003547 | 85 | Shoulder girdle muscle weakness |
| HP:0003731 | 85 | Quadriceps muscle weakness |
| HP:0006467 | 85 | Limited shoulder movement |
| HP:0031108 | 85 | Triceps weakness |

---


---


---

# Cluster #87

| HPO | Cluster | Description |
| --- | --- | --- |
| HP:0002359 | 87 | Frequent falls |
| HP:0009046 | 87 | Difficulty running |
| HP:0008981 | 87 | Calf muscle hypertrophy |
| HP:0011712 | 87 | Right bundle branch block |
| HP:0003551 | 87 | Difficulty climbing stairs |
| HP:0003691 | 87 | Scapular winging |
| HP:0003730 | 87 | EMG: myotonic runs |
| HP:0025169 | 87 | Left ventricular systolic dysfunction |
| HP:0001771 | 87 | Achilles tendon contracture |
| HP:0030051 | 87 | Tip-toe gait |
| HP:0030007 | 87 | EMG: positive sharp waves |
| HP:0100284 | 87 | EMG: myotonic discharges |

---


---


---

# Cluster #88

| HPO | Cluster | Description |
| --- | --- | --- |
| HP:0002093 | 88 | Respiratory insufficiency |
| HP:0003202 | 88 | Skeletal muscle atrophy |
| HP:0003236 | 88 | Elevated serum creatine kinase |
| HP:0000508 | 88 | Ptosis |
| HP:0002375 | 88 | Hypokinesia |
| HP:0001561 | 88 | Polyhydramnios |
| HP:0002804 | 88 | Arthrogryposis multiplex congenita |
| HP:0003323 | 88 | Progressive muscle weakness |
| HP:0001638 | 88 | Cardiomyopathy |
| HP:0002047 | 88 | Malignant hyperthermia |
| HP:0003306 | 88 | Spinal rigidity |
| HP:0003273 | 88 | Hip contracture |
| HP:0003737 | 88 | Mitochondrial myopathy |

---


---


---

# Cluster #90

| HPO | Cluster | Description |
| --- | --- | --- |
| HP:0001288 | 90 | Gait disturbance |
| HP:0001324 | 90 | Muscle weakness |
| HP:0000298 | 90 | Mask-like facies |
| HP:0001284 | 90 | Areflexia |
| HP:0000602 | 90 | Ophthalmoplegia |

---


---


---

# Cluster #91

| HPO | Cluster | Description |
| --- | --- | --- |
| HP:0001290 | 91 | Generalized hypotonia |
| HP:0003236 | 91 | Elevated serum creatine kinase |
| HP:0003712 | 91 | Skeletal muscle hypertrophy |
| HP:0003391 | 91 | Gowers sign |
| HP:0003737 | 91 | Mitochondrial myopathy |
| HP:0000597 | 91 | Ophthalmoparesis |
| HP:0000298 | 91 | Mask-like facies |
| HP:0003200 | 91 | Ragged-red muscle fibers |
| HP:0003306 | 91 | Spinal rigidity |

---


---


---

# Cluster #92

| HPO | Cluster | Description |
| --- | --- | --- |
| HP:0002355 | 92 | Difficulty walking |
| HP:0009027 | 92 | Foot dorsiflexor weakness |
| HP:0001265 | 92 | Hyporeflexia |
| HP:0009046 | 92 | Difficulty running |
| HP:0001284 | 92 | Areflexia |
| HP:0001638 | 92 | Cardiomyopathy |
| HP:0003391 | 92 | Gowers sign |
| HP:0003749 | 92 | Pelvic girdle muscle weakness |
| HP:0000467 | 92 | Neck muscle weakness |
| HP:0002747 | 92 | Respiratory insufficiency due to muscle weakness |
| HP:0008997 | 92 | Proximal muscle weakness in upper limbs |
| HP:0009073 | 92 | Progressive proximal muscle weakness |
| HP:0040081 | 92 | Abnormal circulating creatine kinase concentration |
| HP:0012548 | 92 | Fatty replacement of skeletal muscle |
| HP:0009063 | 92 | Progressive distal muscle weakness |
| HP:0001771 | 92 | Achilles tendon contracture |
| HP:0003547 | 92 | Shoulder girdle muscle weakness |

---


---


---

# Cluster #94

| HPO | Cluster | Description |
| --- | --- | --- |
| HP:0031374 | 94 | Ankle weakness |
| HP:0007149 | 94 | Distal upper limb amyotrophy |
| HP:0002835 | 94 | Aspiration |
| HP:0008049 | 94 | Abnormality of the extraocular muscles |
| HP:0007354 | 94 | Amyotrophic lateral sclerosis |
| HP:0001430 | 94 | Abnormality of the calf musculature |

---


---


---

# Cluster #95

| HPO | Cluster | Description |
| --- | --- | --- |
| HP:0008800 | 95 | Limited hip movement |
| HP:0003394 | 95 | Muscle spasm |
| HP:0001436 | 95 | Abnormality of the foot musculature |
| HP:0009027 | 95 | Foot dorsiflexor weakness |
| HP:0031374 | 95 | Ankle weakness |
| HP:0008956 | 95 | Proximal lower limb amyotrophy |
| HP:0001638 | 95 | Cardiomyopathy |
| HP:0003324 | 95 | Generalized muscle weakness |
| HP:0030198 | 95 | Fatigable weakness of distal limb muscles |
| HP:0008963 | 95 | Tibialis muscle weakness |
| HP:0003555 | 95 | Muscle fiber splitting |
| HP:0100297 | 95 | Increased endomysial connective tissue |
| HP:0003756 | 95 | Skeletal myopathy |
| HP:0009077 | 95 | Weakness of long finger extensor muscles |
| HP:0006467 | 95 | Limited shoulder movement |
| HP:0011808 | 95 | Decreased patellar reflex |
| HP:0031108 | 95 | Triceps weakness |

---


---


---

# Cluster #99

| HPO | Cluster | Description |
| --- | --- | --- |
| HP:0001270 | 99 | Motor delay |
| HP:0002359 | 99 | Frequent falls |
| HP:0000580 | 99 | Pigmentary retinopathy |
| HP:0001712 | 99 | Left ventricular hypertrophy |
| HP:0003546 | 99 | Exercise intolerance |
| HP:0003128 | 99 | Lactic acidosis |
| HP:0003324 | 99 | Generalized muscle weakness |
| HP:0006957 | 99 | Loss of ability to walk |
| HP:0030319 | 99 | Weakness of facial musculature |
| HP:0003737 | 99 | Mitochondrial myopathy |
| HP:0003756 | 99 | Skeletal myopathy |

---


---


---

# Cluster #102

| HPO | Cluster | Description |
| --- | --- | --- |
| HP:0003198 | 102 | Myopathy |
| HP:0001270 | 102 | Motor delay |
| HP:0009053 | 102 | Distal lower limb muscle weakness |
| HP:0002515 | 102 | Waddling gait |
| HP:0001265 | 102 | Hyporeflexia |
| HP:0003236 | 102 | Elevated serum creatine kinase |
| HP:0010628 | 102 | Facial palsy |
| HP:0001315 | 102 | Reduced tendon reflexes |
| HP:0002804 | 102 | Arthrogryposis multiplex congenita |
| HP:0001638 | 102 | Cardiomyopathy |
| HP:0002058 | 102 | Myopathic facies |
| HP:0003701 | 102 | Proximal muscle weakness |
| HP:0002421 | 102 | Poor head control |
| HP:0003306 | 102 | Spinal rigidity |
| HP:0002540 | 102 | Inability to walk |
| HP:0100295 | 102 | Muscle fiber atrophy |
| HP:0003691 | 102 | Scapular winging |
| HP:0001612 | 102 | Weak cry |
| HP:0003730 | 102 | EMG: myotonic runs |
| HP:0006785 | 102 | Limb-girdle muscular dystrophy |

---


---


---

# Cluster #104

| HPO | Cluster | Description |
| --- | --- | --- |
| HP:0008800 | 104 | Limited hip movement |
| HP:0001436 | 104 | Abnormality of the foot musculature |
| HP:0030237 | 104 | Hand muscle weakness |
| HP:0030838 | 104 | Hip pain |
| HP:0031374 | 104 | Ankle weakness |
| HP:0040131 | 104 | Abnormal motor nerve conduction velocity |
| HP:0007354 | 104 | Amyotrophic lateral sclerosis |

---


---


---

# Cluster #105

| HPO | Cluster | Description |
| --- | --- | --- |
| HP:0009053 | 105 | Distal lower limb muscle weakness |
| HP:0002355 | 105 | Difficulty walking |
| HP:0009027 | 105 | Foot dorsiflexor weakness |
| HP:0009046 | 105 | Difficulty running |
| HP:0030198 | 105 | Fatigable weakness of distal limb muscles |
| HP:0002792 | 105 | Reduced vital capacity |
| HP:0008954 | 105 | Intrinsic hand muscle atrophy |
| HP:0009005 | 105 | Weakness of the intrinsic hand muscles |
| HP:0009077 | 105 | Weakness of long finger extensor muscles |
| HP:0008959 | 105 | Distal upper limb muscle weakness |
| HP:0009073 | 105 | Progressive proximal muscle weakness |
| HP:0040081 | 105 | Abnormal circulating creatine kinase concentration |
| HP:0009063 | 105 | Progressive distal muscle weakness |
| HP:0012548 | 105 | Fatty replacement of skeletal muscle |

---


---


---

# Cluster #106

| HPO | Cluster | Description |
| --- | --- | --- |
| HP:0002910 | 106 | Elevated hepatic transaminase |
| HP:0003394 | 106 | Muscle spasm |
| HP:0001644 | 106 | Dilated cardiomyopathy |
| HP:0001638 | 106 | Cardiomyopathy |
| HP:0003128 | 106 | Lactic acidosis |
| HP:0008331 | 106 | Elevated creatine kinase after exercise |
| HP:0040081 | 106 | Abnormal circulating creatine kinase concentration |

---


---


---

# Cluster #107

| HPO | Cluster | Description |
| --- | --- | --- |
| HP:0002359 | 107 | Frequent falls |
| HP:0040083 | 107 | Toe walking |
| HP:0007340 | 107 | Lower limb muscle weakness |
| HP:0007210 | 107 | Lower limb amyotrophy |
| HP:0002355 | 107 | Difficulty walking |
| HP:0003394 | 107 | Muscle spasm |
| HP:0003236 | 107 | Elevated serum creatine kinase |
| HP:0008081 | 107 | Pes valgus |
| HP:0003551 | 107 | Difficulty climbing stairs |
| HP:0009063 | 107 | Progressive distal muscle weakness |

---


---


---

# Cluster #108

| HPO | Cluster | Description |
| --- | --- | --- |
| HP:0003198 | 108 | Myopathy |
| HP:0007149 | 108 | Distal upper limb amyotrophy |
| HP:0003457 | 108 | EMG abnormality |
| HP:0009743 | 108 | Distichiasis |
| HP:0001621 | 108 | Weak voice |
| HP:0002835 | 108 | Aspiration |
| HP:0007354 | 108 | Amyotrophic lateral sclerosis |

---


---


---

# Cluster #109

| HPO | Cluster | Description |
| --- | --- | --- |
| HP:0002359 | 109 | Frequent falls |
| HP:0001288 | 109 | Gait disturbance |
| HP:0002355 | 109 | Difficulty walking |
| HP:0001290 | 109 | Generalized hypotonia |
| HP:0001324 | 109 | Muscle weakness |
| HP:0003236 | 109 | Elevated serum creatine kinase |
| HP:0003712 | 109 | Skeletal muscle hypertrophy |
| HP:0000597 | 109 | Ophthalmoparesis |
| HP:0001276 | 109 | Hypertonia |
| HP:0003326 | 109 | Myalgia |
| HP:0000590 | 109 | Progressive external ophthalmoplegia |
| HP:0003391 | 109 | Gowers sign |
| HP:0003749 | 109 | Pelvic girdle muscle weakness |
| HP:0003457 | 109 | EMG abnormality |
| HP:0003546 | 109 | Exercise intolerance |
| HP:0100749 | 109 | Chest pain |
| HP:0003324 | 109 | Generalized muscle weakness |
| HP:0000467 | 109 | Neck muscle weakness |
| HP:0001349 | 109 | Facial diplegia |
| HP:0003551 | 109 | Difficulty climbing stairs |
| HP:0003458 | 109 | EMG: myopathic abnormalities |
| HP:0008180 | 109 | Mildly elevated creatine kinase |
| HP:0009073 | 109 | Progressive proximal muscle weakness |
| HP:0040081 | 109 | Abnormal circulating creatine kinase concentration |
| HP:0012548 | 109 | Fatty replacement of skeletal muscle |
| HP:0030319 | 109 | Weakness of facial musculature |
| HP:0003737 | 109 | Mitochondrial myopathy |
| HP:0003547 | 109 | Shoulder girdle muscle weakness |
| HP:0002486 | 109 | Myotonia |
| HP:0003707 | 109 | Calf muscle pseudohypertrophy |
| HP:0009063 | 109 | Progressive distal muscle weakness |

---


---


---

# Cluster #110

| HPO | Cluster | Description |
| --- | --- | --- |
| HP:0002015 | 110 | Dysphagia |
| HP:0001270 | 110 | Motor delay |
| HP:0002359 | 110 | Frequent falls |
| HP:0003693 | 110 | Distal amyotrophy |
| HP:0007340 | 110 | Lower limb muscle weakness |
| HP:0003484 | 110 | Upper limb muscle weakness |
| HP:0002355 | 110 | Difficulty walking |
| HP:0003394 | 110 | Muscle spasm |
| HP:0007141 | 110 | Sensorimotor neuropathy |
| HP:0001265 | 110 | Hyporeflexia |
| HP:0001324 | 110 | Muscle weakness |
| HP:0001284 | 110 | Areflexia |
| HP:0002460 | 110 | Distal muscle weakness |
| HP:0000597 | 110 | Ophthalmoparesis |
| HP:0002375 | 110 | Hypokinesia |
| HP:0000602 | 110 | Ophthalmoplegia |
| HP:0003326 | 110 | Myalgia |
| HP:0002878 | 110 | Respiratory failure |
| HP:0000590 | 110 | Progressive external ophthalmoplegia |
| HP:0003200 | 110 | Ragged-red muscle fibers |
| HP:0003546 | 110 | Exercise intolerance |
| HP:0003324 | 110 | Generalized muscle weakness |
| HP:0001618 | 110 | Dysphonia |
| HP:0002540 | 110 | Inability to walk |
| HP:0100295 | 110 | Muscle fiber atrophy |
| HP:0001349 | 110 | Facial diplegia |
| HP:0003551 | 110 | Difficulty climbing stairs |
| HP:0002747 | 110 | Respiratory insufficiency due to muscle weakness |
| HP:0005855 | 110 | Multiple prenatal fractures |
| HP:0003388 | 110 | Easy fatigability |
| HP:0001612 | 110 | Weak cry |
| HP:0002578 | 110 | Gastroparesis |
| HP:0002127 | 110 | Abnormal upper motor neuron morphology |
| HP:0001283 | 110 | Bulbar palsy |
| HP:0030319 | 110 | Weakness of facial musculature |
| HP:0003737 | 110 | Mitochondrial myopathy |
| HP:0012664 | 110 | Reduced ejection fraction |
| HP:0002835 | 110 | Aspiration |
| HP:0005085 | 110 | Limited knee flexion/extension |
| HP:0100748 | 110 | Muscular edema |
| HP:0008049 | 110 | Abnormality of the extraocular muscles |
| HP:0010548 | 110 | Percussion myotonia |

---


---


---

# Cluster #116

| HPO | Cluster | Description |
| --- | --- | --- |
| HP:0002515 | 116 | Waddling gait |
| HP:0009046 | 116 | Difficulty running |
| HP:0008981 | 116 | Calf muscle hypertrophy |
| HP:0003236 | 116 | Elevated serum creatine kinase |
| HP:0003560 | 116 | Muscular dystrophy |
| HP:0001644 | 116 | Dilated cardiomyopathy |
| HP:0003391 | 116 | Gowers sign |
| HP:0003749 | 116 | Pelvic girdle muscle weakness |
| HP:0010508 | 116 | Metatarsus valgus |
| HP:0003701 | 116 | Proximal muscle weakness |
| HP:0003551 | 116 | Difficulty climbing stairs |
| HP:0040081 | 116 | Abnormal circulating creatine kinase concentration |
| HP:0002987 | 116 | Elbow flexion contracture |
| HP:0001771 | 116 | Achilles tendon contracture |
| HP:0003547 | 116 | Shoulder girdle muscle weakness |

---


---


---

# Cluster #119

| HPO | Cluster | Description |
| --- | --- | --- |
| HP:0001270 | 119 | Motor delay |
| HP:0002515 | 119 | Waddling gait |
| HP:0001265 | 119 | Hyporeflexia |
| HP:0001284 | 119 | Areflexia |
| HP:0003236 | 119 | Elevated serum creatine kinase |
| HP:0000508 | 119 | Ptosis |
| HP:0000597 | 119 | Ophthalmoparesis |
| HP:0001561 | 119 | Polyhydramnios |
| HP:0000298 | 119 | Mask-like facies |
| HP:0004326 | 119 | Cachexia |
| HP:0000602 | 119 | Ophthalmoplegia |
| HP:0002804 | 119 | Arthrogryposis multiplex congenita |
| HP:0003200 | 119 | Ragged-red muscle fibers |
| HP:0003128 | 119 | Lactic acidosis |
| HP:0003701 | 119 | Proximal muscle weakness |
| HP:0002421 | 119 | Poor head control |
| HP:0001558 | 119 | Decreased fetal movement |
| HP:0003306 | 119 | Spinal rigidity |
| HP:0001349 | 119 | Facial diplegia |
| HP:0003691 | 119 | Scapular winging |
| HP:0003803 | 119 | Type 1 muscle fiber predominance |
| HP:0002747 | 119 | Respiratory insufficiency due to muscle weakness |
| HP:0003458 | 119 | EMG: myopathic abnormalities |
| HP:0003388 | 119 | Easy fatigability |
| HP:0000600 | 119 | Abnormality of the pharynx |
| HP:0002578 | 119 | Gastroparesis |
| HP:0011807 | 119 | Type 1 muscle fiber atrophy |
| HP:0003737 | 119 | Mitochondrial myopathy |
| HP:0008316 | 119 | Abnormal mitochondria in muscle tissue |

---


---


---

# Cluster #121

| HPO | Cluster | Description |
| --- | --- | --- |
| HP:0002375 | 121 | Hypokinesia |
| HP:0009025 | 121 | Increased connective tissue |
| HP:0012664 | 121 | Reduced ejection fraction |
| HP:0002835 | 121 | Aspiration |
| HP:0030234 | 121 | Highly elevated creatine kinase |
| HP:0003741 | 121 | Congenital muscular dystrophy |

---


---


---

# Cluster #123

| HPO | Cluster | Description |
| --- | --- | --- |
| HP:0002460 | 123 | Distal muscle weakness |
| HP:0003458 | 123 | EMG: myopathic abnormalities |
| HP:0008180 | 123 | Mildly elevated creatine kinase |
| HP:0008316 | 123 | Abnormal mitochondria in muscle tissue |
| HP:0008049 | 123 | Abnormality of the extraocular muscles |
| HP:0007354 | 123 | Amyotrophic lateral sclerosis |
| HP:0001430 | 123 | Abnormality of the calf musculature |

---


---


---

# Cluster #126

| HPO | Cluster | Description |
| --- | --- | --- |
| HP:0001270 | 126 | Motor delay |
| HP:0002359 | 126 | Frequent falls |
| HP:0001290 | 126 | Generalized hypotonia |
| HP:0003326 | 126 | Myalgia |
| HP:0003391 | 126 | Gowers sign |
| HP:0003546 | 126 | Exercise intolerance |
| HP:0003324 | 126 | Generalized muscle weakness |
| HP:0003700 | 126 | Generalized amyotrophy |
| HP:0001349 | 126 | Facial diplegia |
| HP:0001712 | 126 | Left ventricular hypertrophy |
| HP:0003551 | 126 | Difficulty climbing stairs |
| HP:0003756 | 126 | Skeletal myopathy |
| HP:0003687 | 126 | Centrally nucleated skeletal muscle fibers |
| HP:0003803 | 126 | Type 1 muscle fiber predominance |
| HP:0003388 | 126 | Easy fatigability |

---


---


---

# Cluster #129

| HPO | Cluster | Description |
| --- | --- | --- |
| HP:0001288 | 129 | Gait disturbance |
| HP:0001324 | 129 | Muscle weakness |
| HP:0003236 | 129 | Elevated serum creatine kinase |
| HP:0003712 | 129 | Skeletal muscle hypertrophy |
| HP:0000597 | 129 | Ophthalmoparesis |
| HP:0001276 | 129 | Hypertonia |
| HP:0000298 | 129 | Mask-like facies |
| HP:0004326 | 129 | Cachexia |
| HP:0001315 | 129 | Reduced tendon reflexes |
| HP:0003307 | 129 | Hyperlordosis |
| HP:0003457 | 129 | EMG abnormality |
| HP:0100749 | 129 | Chest pain |
| HP:0002486 | 129 | Myotonia |
| HP:0003200 | 129 | Ragged-red muscle fibers |

---


---


---

# Cluster #135

| HPO | Cluster | Description |
| --- | --- | --- |
| HP:0007340 | 135 | Lower limb muscle weakness |
| HP:0003394 | 135 | Muscle spasm |
| HP:0001324 | 135 | Muscle weakness |
| HP:0003201 | 135 | Rhabdomyolysis |
| HP:0001663 | 135 | Ventricular fibrillation |
| HP:0009020 | 135 | Exercise-induced muscle fatigue |
| HP:0003756 | 135 | Skeletal myopathy |
| HP:0011808 | 135 | Decreased patellar reflex |

---


---


---

# Cluster #136

| HPO | Cluster | Description |
| --- | --- | --- |
| HP:0009053 | 136 | Distal lower limb muscle weakness |
| HP:0003693 | 136 | Distal amyotrophy |
| HP:0007340 | 136 | Lower limb muscle weakness |
| HP:0003202 | 136 | Skeletal muscle atrophy |
| HP:0007141 | 136 | Sensorimotor neuropathy |
| HP:0003484 | 136 | Upper limb muscle weakness |
| HP:0000580 | 136 | Pigmentary retinopathy |
| HP:0009027 | 136 | Foot dorsiflexor weakness |
| HP:0001284 | 136 | Areflexia |
| HP:0002460 | 136 | Distal muscle weakness |
| HP:0001638 | 136 | Cardiomyopathy |
| HP:0002421 | 136 | Poor head control |
| HP:0002540 | 136 | Inability to walk |
| HP:0003551 | 136 | Difficulty climbing stairs |
| HP:0002522 | 136 | Areflexia of lower limbs |

---


---


---

# Cluster #139

| HPO | Cluster | Description |
| --- | --- | --- |
| HP:0000580 | 139 | Pigmentary retinopathy |
| HP:0001644 | 139 | Dilated cardiomyopathy |
| HP:0005150 | 139 | Abnormal atrioventricular conduction |
| HP:0006957 | 139 | Loss of ability to walk |
| HP:0003756 | 139 | Skeletal myopathy |
| HP:0003797 | 139 | Limb-girdle muscle atrophy |

---


---


---

# Cluster #142

| HPO | Cluster | Description |
| --- | --- | --- |
| HP:0001436 | 142 | Abnormality of the foot musculature |
| HP:0002878 | 142 | Respiratory failure |
| HP:0001638 | 142 | Cardiomyopathy |
| HP:0003546 | 142 | Exercise intolerance |
| HP:0003306 | 142 | Spinal rigidity |
| HP:0003327 | 142 | Axial muscle weakness |
| HP:0003803 | 142 | Type 1 muscle fiber predominance |
| HP:0002522 | 142 | Areflexia of lower limbs |
| HP:0002747 | 142 | Respiratory insufficiency due to muscle weakness |
| HP:0003722 | 142 | Neck flexor weakness |
| HP:0030196 | 142 | Fatigable weakness of respiratory muscles |
| HP:0030192 | 142 | Fatigable weakness of bulbar muscles |
| HP:0030319 | 142 | Weakness of facial musculature |

---


---


---

# Cluster #143

| HPO | Cluster | Description |
| --- | --- | --- |
| HP:0003394 | 143 | Muscle spasm |
| HP:0000597 | 143 | Ophthalmoparesis |
| HP:0000298 | 143 | Mask-like facies |
| HP:0003200 | 143 | Ragged-red muscle fibers |
| HP:0002578 | 143 | Gastroparesis |
| HP:0003737 | 143 | Mitochondrial myopathy |
| HP:0000544 | 143 | External ophthalmoplegia |
| HP:0003457 | 143 | EMG abnormality |
| HP:0003388 | 143 | Easy fatigability |
| HP:0012664 | 143 | Reduced ejection fraction |

---


---


---

# Cluster #145

| HPO | Cluster | Description |
| --- | --- | --- |
| HP:0002359 | 145 | Frequent falls |
| HP:0009053 | 145 | Distal lower limb muscle weakness |
| HP:0002355 | 145 | Difficulty walking |
| HP:0008994 | 145 | Proximal muscle weakness in lower limbs |
| HP:0009046 | 145 | Difficulty running |
| HP:0003236 | 145 | Elevated serum creatine kinase |
| HP:0002540 | 145 | Inability to walk |
| HP:0003551 | 145 | Difficulty climbing stairs |
| HP:0008180 | 145 | Mildly elevated creatine kinase |
| HP:0008997 | 145 | Proximal muscle weakness in upper limbs |
| HP:0008959 | 145 | Distal upper limb muscle weakness |
| HP:0030237 | 145 | Hand muscle weakness |
| HP:0001265 | 145 | Hyporeflexia |
| HP:0001284 | 145 | Areflexia |
| HP:0001638 | 145 | Cardiomyopathy |
| HP:0003546 | 145 | Exercise intolerance |
| HP:0003738 | 145 | Exercise-induced myalgia |
| HP:0003722 | 145 | Neck flexor weakness |
| HP:0030192 | 145 | Fatigable weakness of bulbar muscles |
| HP:0006957 | 145 | Loss of ability to walk |
| HP:0001771 | 145 | Achilles tendon contracture |
| HP:0003731 | 145 | Quadriceps muscle weakness |
| HP:0030051 | 145 | Tip-toe gait |
| HP:0031108 | 145 | Triceps weakness |
| HP:0030198 | 145 | Fatigable weakness of distal limb muscles |
| HP:0003805 | 145 | Rimmed vacuoles |
| HP:0005085 | 145 | Limited knee flexion/extension |
| HP:0100748 | 145 | Muscular edema |

---


---


---

# Cluster #146

| HPO | Cluster | Description |
| --- | --- | --- |
| HP:0003323 | 146 | Progressive muscle weakness |
| HP:0012507 | 146 | Weakness of orbicularis oculi muscle |
| HP:0008316 | 146 | Abnormal mitochondria in muscle tissue |
| HP:0001430 | 146 | Abnormality of the calf musculature |

---


---


---

# Cluster #147

| HPO | Cluster | Description |
| --- | --- | --- |
| HP:0002359 | 147 | Frequent falls |
| HP:0040083 | 147 | Toe walking |
| HP:0001265 | 147 | Hyporeflexia |
| HP:0003236 | 147 | Elevated serum creatine kinase |
| HP:0001644 | 147 | Dilated cardiomyopathy |
| HP:0001638 | 147 | Cardiomyopathy |
| HP:0000590 | 147 | Progressive external ophthalmoplegia |
| HP:0003200 | 147 | Ragged-red muscle fibers |
| HP:0003546 | 147 | Exercise intolerance |
| HP:0001618 | 147 | Dysphonia |
| HP:0002747 | 147 | Respiratory insufficiency due to muscle weakness |
| HP:0009073 | 147 | Progressive proximal muscle weakness |
| HP:0030319 | 147 | Weakness of facial musculature |
| HP:0003306 | 147 | Spinal rigidity |
| HP:0000467 | 147 | Neck muscle weakness |
| HP:0001349 | 147 | Facial diplegia |
| HP:0003551 | 147 | Difficulty climbing stairs |
| HP:0008180 | 147 | Mildly elevated creatine kinase |
| HP:0008997 | 147 | Proximal muscle weakness in upper limbs |
| HP:0003690 | 147 | Limb muscle weakness |
| HP:0030192 | 147 | Fatigable weakness of bulbar muscles |
| HP:0003388 | 147 | Easy fatigability |
| HP:0001771 | 147 | Achilles tendon contracture |
| HP:0003737 | 147 | Mitochondrial myopathy |
| HP:0003547 | 147 | Shoulder girdle muscle weakness |

---


---


---

# Cluster #149

| HPO | Cluster | Description |
| --- | --- | --- |
| HP:0008800 | 149 | Limited hip movement |
| HP:0011712 | 149 | Right bundle branch block |
| HP:0002540 | 149 | Inability to walk |
| HP:0002792 | 149 | Reduced vital capacity |
| HP:0012548 | 149 | Fatty replacement of skeletal muscle |
| HP:0100297 | 149 | Increased endomysial connective tissue |
| HP:0012664 | 149 | Reduced ejection fraction |
| HP:0030051 | 149 | Tip-toe gait |
| HP:0003736 | 149 | Autophagic vacuoles |
| HP:0005085 | 149 | Limited knee flexion/extension |
| HP:0100748 | 149 | Muscular edema |
| HP:0012515 | 149 | Hip flexor weakness |
