# Supplementary material for: Decoding Neuromuscular Disorders Using Phenotypic Clusters Obtained From Co-Occurrence Networks
Source: Front Mol Biosci. 2021 Apr 19;8:635074. doi: 10.3389/fmolb.2021.635074 (PMC8147726; doi:10.3389/fmolb.2021.635074)
Supplement: Supplementary file 1 [file DataSheet1.zip › Sup5-Top10_phenotypes.pdf]

**HPO code**	**Phenotype**	**Counts in NMDs**	**Counts in non-NMDs**	***P*-value**
From OMIM database				
HP:0003236	**Elevated serum creatine kinase**	124	59	1.841099e-96
HP:0001284	Areflexia	110	58	2.162695e-82
HP:0001265	Hyporeflexia	117	100	4.686633e-74
HP:0002460	Distal muscle weakness	81	18	3.014901e-73
HP:0003701	Proximal muscle weakness	73	20	2.369912e-63
HP:0002936	Distal sensory impairment	74	29	2.761166e-59
HP:0003560	Muscular dystrophy	60	7	5.018812e-59
HP:0003693	Distal amyotrophy	72	27	2.756548e-58
HP:0003677	Slow progression	90	85	8.981719e-54
HP:0010628	Facial palsy	63	34	4.818952e-46
From Orphanet database				
HP:0003236	**Elevated serum creatine kinase**	40	23	3.047233e-35
HP:0003551	Difficulty climbing stairs	20	5	7.287806e-21
HP:0003457	EMG abnormality	25	17	7.380621e-21
HP:0003458	EMG: myopathic abnormalities	25	18	1.676727e-20
HP:0008981	Calf muscle hypertrophy	17	5	2.290024e-17
HP:0002515	Waddling gait	19	13	5.854754e-16
HP:0003557	Increased variability in muscle fiber diameter	17	9	2.199215e-15
HP:0003198	Myopathy	36	24	7.730904e-15
HP:0003805	Rimmed vacuoles	13	2	1.117866e-14
HP:0003391	Gowers sign	13	2	1.117866e-14
