# Supplementary figures and images for: Decoding Neuromuscular Disorders Using Phenotypic Clusters Obtained From Co-Occurrence Networks
Source: Front Mol Biosci. 2021 Apr 19;8:635074. doi: 10.3389/fmolb.2021.635074 (PMC8147726; doi:10.3389/fmolb.2021.635074)

# GO

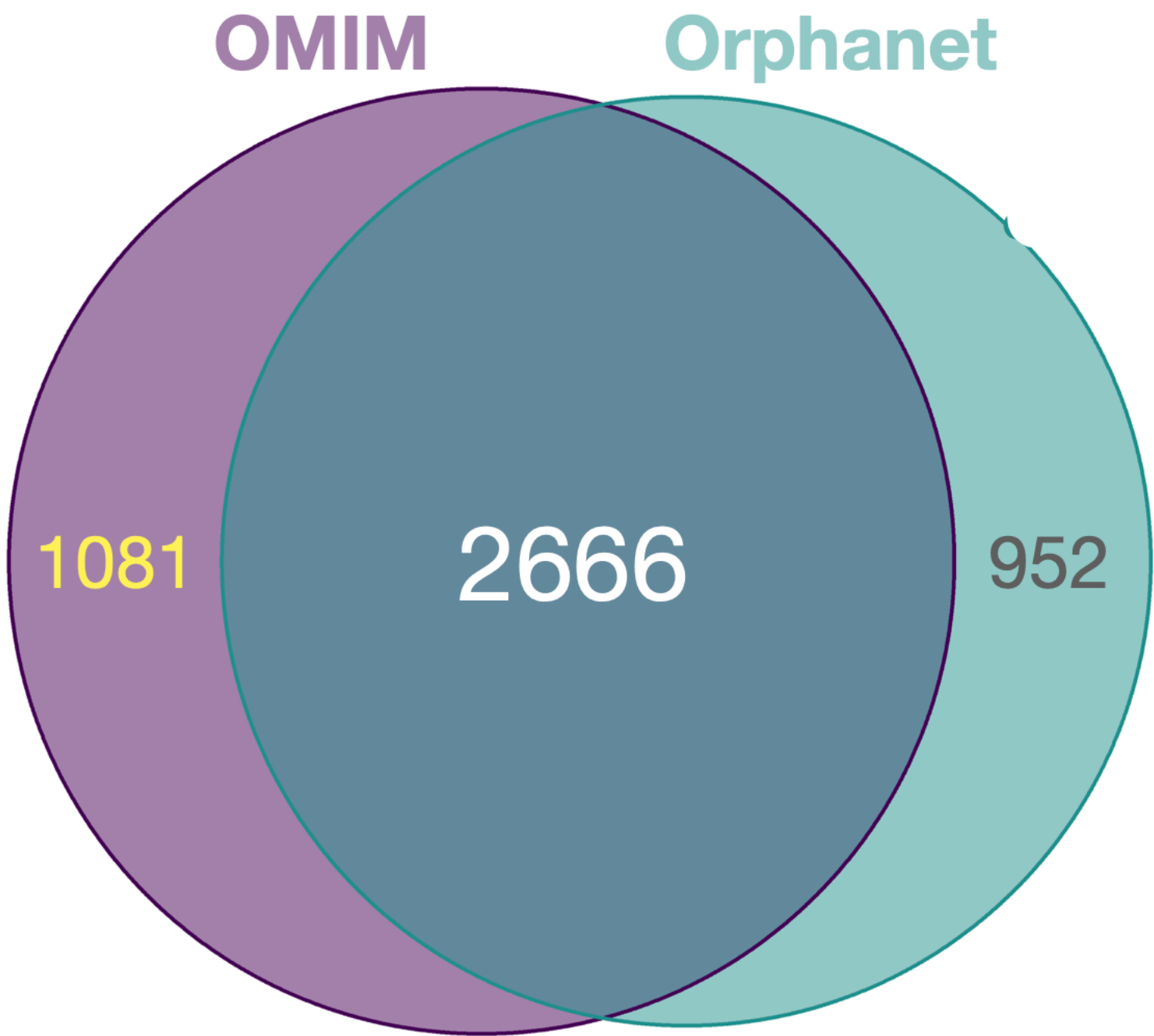

# KEGG

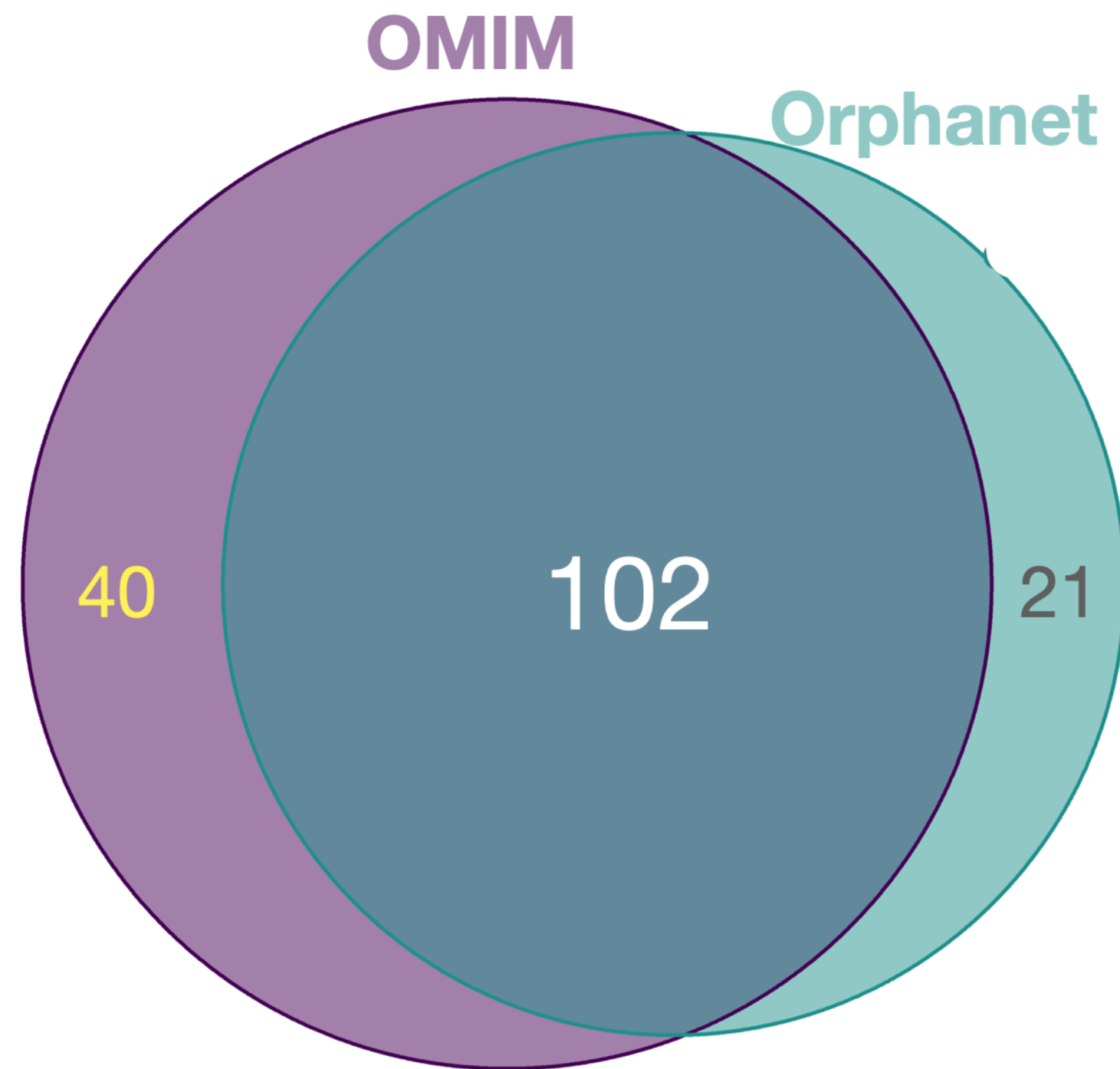

# Reactome

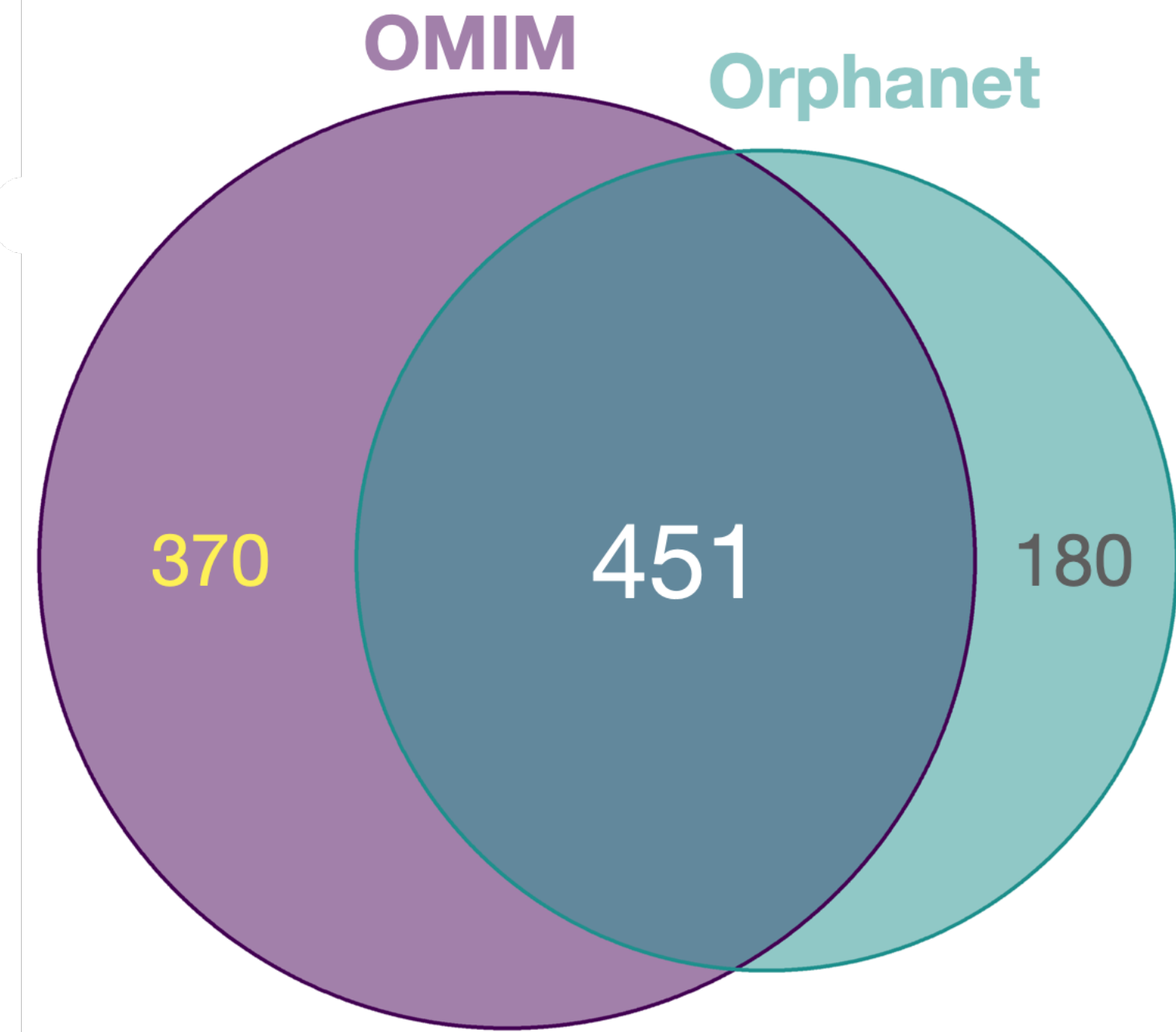

Supplement: Supplementary file 1 [file DataSheet1.zip › Sup6-Venn-functions.pdf]
